# Choice of Glucose-Lowering Drugs as Initial Monotherapy for Type 2 Diabetes Patients with Contraindications or Intolerance to Metformin: A Systematic Review and Meta-Analysis

**DOI:** 10.3390/jcm11237094

**Published:** 2022-11-30

**Authors:** Shuyan Gu, Xiaoqian Hu, Lizheng Shi, Xuemei Zhen, Xueshan Sun, Minzhuo Huang, Yuxuan Gu, Hengjin Dong

**Affiliations:** 1 Center for Health Policy and Management Studies, School of Government, Nanjing University, Nanjing 210023, China; 2 College of Politics and Public Administration, Qingdao University, Qingdao 266071, China; 3 Department of Global Health Management and Policy, School of Public Health and Tropical Medicine, Tulane University, New Orleans, LA 70112, USA; 4 Centre for Health Management and Policy Research, School of Public Health, Cheeloo College of Medicine (NHC Key Laboratory of Health Economics and Policy Research), Shandong University, Jinan 250012, China; 5 Center for Health Policy Studies, School of Public Health, Zhejiang University School of Medicine, Hangzhou 310058, China

**Keywords:** type 2 diabetes, initial monotherapy, metformin, sulfonylureas, thiazolidinediones, glinides, α-glucosidase inhibitors, dipeptidyl peptidase-4 inhibitors, sodium-glucose cotransporter-2 inhibitors, glucagon-like peptide-1 receptor agonists

## Abstract

Background: There are multiple glucose-lowering drugs available as alternative initial monotherapy for type 2 diabetes patients with contraindications or intolerance to metformin. However, little comparative and systematic data are available for them as initial monotherapy. This study estimated and compared the treatment effects of glucose-lowering drugs as initial monotherapy for type 2 diabetes. Methods: PubMed, Web of Science, Embase, CNKI, Chongqing VIP, and WanFang Data from 1 January 1990 until 31 December 2020 were searched for randomized controlled trials which compared a glucose-lowering drug with placebo/lifestyle-intervention for type 2 diabetes. Drug classes included metformin, sulfonylureas (SUs), thiazolidinediones (TZDs), glinides (NIDEs), α-glucosidase inhibitors (AGIs), dipeptidyl peptidase-4 inhibitors (DPP-4is), sodium-glucose cotransporter-2 inhibitors (SGLT2is), insulins (INSs), and glucagon-like peptide-1 receptor agonists (GLP-1RAs). Results: A total of 185 trials were included, identifying 38,376 patients from 56 countries across six continents. When choosing an initial drug monotherapy alternative to metformin, SUs were most efficacious in reducing HbA1c (−1.39%; 95% CI −1.63, −1.16) and FPG (−2.70 mmol/L; 95% CI −3.18, −2.23), but increased hypoglycemia risks (5.44; 95% CI 2.11, 14.02). GLP-1RAs were most efficacious in reducing BMI (−1.05 kg/m^2^; 95% CI −1.81, −0.29) and TC (−0.42 mmol/L; 95% CI −0.61, −0.22). TZDs were most efficacious in increasing HDL-C (0.12 mmol/L; 95% CI 0.07, 0.17). SGLT2is were most efficacious in lowering SBP (−4.18 mmHg; 95% CI −4.84, −3.53). While AGIs conferred higher risk of AE-induced discontinuations (2.57; 95% CI 1.64, 4.03). Overall, only GLP-1RAs showed an integrated beneficial effect on all outcomes. Our results also confirmed the intraclass differences in treatment effects across drugs. Most trials were short-term, and no significant differences in mortality, total vascular events, myocardial infarction, heart failure, stroke, or diabetic nephropathy were observed across drug classes. Conclusions: Our results suggest a potential treatment hierarchy for decision-makers, with GLP-1RAs being the preferred alternative therapy to metformin regarding their favorable efficacy and safety profiles.

## 1. Introduction

Diabetes is among the top ten causes of death, imposing serious threats to global health and economy [1]. Globally, one in eleven adults were living with diabetes (463 million), 4.2 million deaths were due to diabetes, and 10% of the health expenditure (USD 760 billion) was spent on diabetes in 2019 [1]. Type 2 diabetes accounts for around 90% of all diabetes, which is characterized by chronic hyperglycemia [2]. Hyperglycemia, if left poorly managed, can lead to severe diabetes-related vascular events and even death. Conversely, if appropriately managed, the events can be prevented or delayed [1,3].

Clinical guidelines recommend metformin as the preferred initial monotherapy added to lifestyle interventions (LIs) for type 2 diabetes patients with proven effectiveness and safety [3,4]. However, in patients with contraindications or intolerance to metformin, initial monotherapy is recommended to consider a glucose-lowering drug from another class based on patient factors and drug-specific effects [3,4]. There are eight alternative drug classes available worldwide, including sulfonylureas (SUs), thiazolidinediones (TZDs), glinides (NIDEs), α-glucosidase inhibitors (AGIs), dipeptidyl peptidase-4 inhibitors (DPP-4is), sodium-glucose cotransporter-2 inhibitors (SGLT2is), insulins (INSs), and glucagon-like peptide-1 receptor agonists (GLP-1RAs), which have increased the complexity of clinical drug use. A national survey in China reported 35.8% of type 2 diabetes patients used monotherapy; among which, insulin secretagogues (SUs or NIDEs) were most commonly used (45.6%), followed by metformin (30%) and AGIs (18%) [5]. However, 32% of patients changed their therapies within a year, attributable to insufficient efficacy and/or intolerant adverse events (AEs) [5].

Regarding the heavy disease burden and frequent therapy alteration, it is of value to choose a rational drug as alternative initial monotherapy when metformin is contraindicated or intolerant. Evidence shows that focusing solely on glucose control has a limited effect on reducing the risks of diabetes-related vascular events and death, while a comprehensive control of cardiovascular risk factors (e.g., hypertension, dyslipidemia) can bring greater benefits, particularly among patients with special characteristics and needs [3,6]. In addition, the diabetes associations recommend a patient-centered approach to guide the choice of drugs, considering efficacy, weight impact, hypoglycemia risk, effect on cardiovascular and renal comorbidities, and patient preferences [4,7]. It is essential to fully understand the advantages and disadvantages of each drug before making a choice. However, there is little systematic and comparative data available for all the alternative drugs as initial monotherapy of type 2 diabetes [4]. Therefore, this study systematically estimated and compared the treatment effects of all available glucose-lowering drugs when used as initial monotherapy for type 2 diabetes, so as to enable decision-makers to make informed choice when choosing an alternative drug as initial monotherapy for patients with contraindications or intolerance to metformin.

## 2. Methods

This study was carried out in accordance with the Preferred Reporting Items for Systematic Reviews and Meta-Analysis (PRISMA) statement [8] and was registered at PROSPERO (CRD42020170769). We estimated the drug-specific effects of each drug after removing the effects of placebo (PBO) and LI and compared the drug classes against each other.

### 2.1. Eligibility Criteria

Studies were eligible if (1) participants were type 2 diabetes patients ≥18 years; (2) intervention was a glucose-lowering drug monotherapy; (3) comparator was PBO or LI such as diet and/or exercise; (4) only LI was allowed as background therapy; (5) study duration was ≥12 weeks; (6) outcomes were hemoglobin Alc (HbA1c), fasting plasma glucose (FPG), body mass index (BMI), total cholesterol (TC), high-density lipoprotein-cholesterol (HDL-C), systolic blood pressure (SBP), hypoglycemia, mortality, vascular outcomes, or discontinuation; (7) study design was randomized controlled trial (RCT); and (8) study was published in English or Chinese. Detailed eligibility criteria are shown in Supplementary Table S1.

27 glucose-lowering drugs from nine drug classes were targeted:
BiguanideMetforminSUsGlyburide, glimepiride, gliclazide, glipizide, and gliquidoneTZDsRosiglitazone and pioglitazoneNIDEsRepaglinide, nateglinide, and mitiglinideAGIsAcarbose, voglibose, and miglitolDPP-4isSitagliptin, saxagliptin, vildagliptin, linagliptin, and alogliptinSGLT2isDapagliflozin, empagliflozin, and canagliflozinINSsInsulin and insulin analogsGLP-1RAsExenatide, liraglutide, lixisenatide, and beinaglutide

### 2.2. Information Sources and Searches

We systematically searched PubMed, Web of Science, Embase, China National Knowledge Infrastructure (CNKI), Chongqing VIP, and WanFang Data for RCTs published between 1 January 1990 and 31 December 2020, which compared a glucose-lowering drug against PBO (or LI) with or without a background of LI for type 2 diabetes. Search terms used were type 2 diabetes, 27 glucose-lowering drugs, nine drug classes, placebo, diet, exercise, and lifestyle. Detailed search strategies are shown in Supplementary Table S2. The reference lists of relevant reviews and retrieved studies were manually screened to supplement for database searching.

### 2.3. Study Selection

After removing the duplicates, two researchers (S.G. and X.H.) screened the title and abstract of each retrieved record independently, and then examined potentially eligible records by reading full texts. The results were cross-checked by two other researchers (X.S. and Y.G.). Any potential disagreements were resolved through consensus by the research team.

### 2.4. Data Extraction and Quality Assessment

Two researchers (S.G. and X.H.) independently extracted data using a standardized form and assessed the risk of bias of each study by using the Cochrane Collaboration’s risk of bias assessment tool [9]. The extracted data included study characteristics, patient characteristics, intervention, comparator, outcomes, and other relevant data. The publication bias across studies was estimated using Egger’s test or Harbord’s modified test. The results were cross-checked by two other researchers (X.S. and Y.G.). Any potential discrepancies were resolved by consensus.

### 2.5. Data Synthesis and Analysis

Firstly, we conducted a series of meta-analyses to estimate the treatment effects of different drugs on each outcome, after removing the effects of PBO/LI. Then, we conducted the adjusted indirect treatment comparisons on the basis of the Bucher method to compare the treatment effects of the drugs with each other by using PBO/LI as common comparator [10,11]. For continuous outcomes, we calculated the weighted mean difference (WMD) with 95% confidence intervals (CIs). For dichotomous outcomes, we calculated the relative risk (RR) with 95% CIs. We used a random-effects model conservatively by assuming a substantial variability in effect size across studies and drugs [12,13]. We used the *I*^2^ statistic to evaluate heterogeneity, which was considered important when it was above 50% [14]. The subgroup analyses were performed based on individual drugs within the same class. The sensitivity analyses were performed using fixed-effects meta-analyses. All analyses were performed in Stata/SE 15.1.

## 3. Results

**Figure 1:**
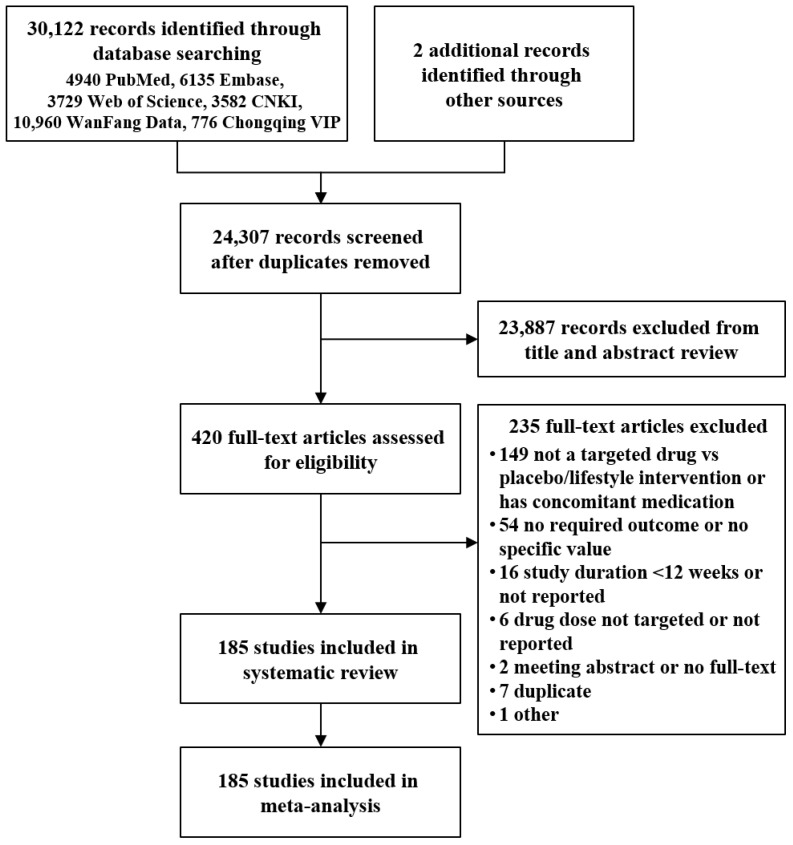
PRISMA flow diagram of study search and selection.

The initial search identified 30,124 records, of which 420 records were assessed in full texts. Finally, 185 trials were included [15,16,17,18,19,20,21,22,23,24,25,26,27,28,29,30,31,32,33,34,35,36,37,38,39,40,41,42,43,44,45,46,47,48,49,50,51,52,53,54,55,56,57,58,59,60,61,62,63,64,65,66,67,68,69,70,71,72,73,74,75,76,77,78,79,80,81,82,83,84,85,86,87,88,89,90,91,92,93,94,95,96,97,98,99,100,101,102,103,104,105,106,107,108,109,110,111,112,113,114,115,116,117,118,119,120,121,122,123,124,125,126,127,128,129,130,131,132,133,134,135,136,137,138,139,140,141,142,143,144,145,146,147,148,149,150,151,152,153,154,155,156,157,158,159,160,161,162,163,164,165,166,167,168,169,170,171,172,173,174,175,176,177,178,179,180,181,182,183,184,185,186,187,188,189,190,191,192,193,194,195,196,197,198,199], which identified 38,376 patients randomly assigned to 23 drugs from eight classes or PBO/LI (Figure 1). The sample size of the trials varied from 16 to 888. The study duration ranged from 12 weeks to 108 weeks. Metformin was studied in 40 trials, with pioglitazone (23 trials), acarbose (22 trials), and rosiglitazone (21 trials) being the three next most studied drugs. No eligible trial was detected for gliquidone, mitiglinide, beinaglutide, or insulin. The participants were from 56 countries across six continents. Mean age varied from 30.8 years to 74.5 years, with a diabetes duration of 0.23 year to 9.9 years. The characteristics with the risk of bias of the trials are presented in Supplementary Table S3. The publication bias is shown in Supplementary Figure S1.

### 3.1. Intermediate Outcomes

#### 3.1.1. Hemoglobin Alc

A total of 167 trials (34,133 patients) provided data on HbA1c. All drugs significantly reduced HbA1c after removing the PBO/LI effects. The largest PBO/LI-subtracted reductions were evident with SUs (1.39%; 95% CI 1.16, 1.63), followed by GLP-1RAs (0.99%; 95% CI 0.78, 1.20); while NIDEs (0.44%; 95% CI 0.20, 0.69) resulted in the smallest reductions. There were great differences in HbA1c reductions detected within GLP-1RAs, ranging from 0.60% (95% CI 0.37, 0.83) with lixisenatide to 1.17% (95% CI 0.87, 1.47) with liraglutide; and within AGIs, ranging from 0.20% (95% CI 0.07, 0.33) with voglibose to 0.74% (95% CI 0.52, 0.96) with acarbose (Table 1; Supplementary Figures S2–S9).

Across the drug classes, SUs produced significantly greater HbA1c reductions than all other drug classes, ranging from 0.40% (95% CI 0.09, 0.72) vs. GLP-1RAs to 0.95% (95% CI 0.61, 1.29) vs. NIDEs. In addition, GLP-1RAs, metformin, and TZDs were superior to DPP-4is, AGIs, and NIDEs. SGLT2is worked better than DPP-4is and NIDEs. The greatest contrast among all comparisons was detected between SUs and NIDEs (Table 2).

#### 3.1.2. Fasting Plasma Glucose

A total of 163 trials (33,963 patients) provided data on FPG. All drug classes significantly reduced FPG after removing the PBO/LI effects. The greatest PBO/LI-subtracted reductions were seen with SUs (2.70 mmol/L; 95% CI 2.23, 3.18), ranging from 2.22 mmol/L (95% CI 0.97, 3.47) with gliclazide to 3.02 mmol/L (95% CI 2.20, 3.85) with glipizide, followed by TZDs (1.91 mmol/L; 95% CI 1.60, 2.23), with reductions of 1.73 mmol/L (95% CI 1.14, 2.32) for rosiglitazone vs. 2.01 mmol/L (95% CI 1.67, 2.34) for pioglitazone. As a class, NIDEs conferred the smallest reductions (0.75 mmol/L; 95% CI 0.45, 1.04), which was inferior to AGIs (1.19 mmol/L; 95% CI 0.64, 1.73); but large differences within AGIs were noted, with a 1.17 mmol/L (95% CI 0.50, 1.83) reduction with acarbose vs. no significant reductions with other AGIs (Table 1; Supplementary Figures S10–S17).

Across the drug classes, SUs showed significantly greater FPG reductions than all other drug classes, ranging from 0.79 mmol/L (95% CI 0.22, 1.36) vs. TZDs to 1.95 mmol/L (95% CI 1.39, 2.51) vs. NIDEs. In addition, TZDs, metformin, GLP-1RAs, and SGLT2is performed better than DPP-4is and NIDEs. TZDs were superior to AGIs (Table 2).

#### 3.1.3. Body Mass Index

A total of 67 trials (4990 patients) provided data on BMI. After removing the PBO/LI effects, metformin and GLP-1RAs significantly reduced BMI by 1.28 kg/m^2^ (95% CI 0.31, 2.26) and 1.05 kg/m^2^ (95% CI 0.29, 1.81), while SUs and TZDs significantly increased BMI by 1.22 kg/m^2^ (95% CI 0.13, 2.31) and 0.63 kg/m^2^ (95% CI 0.26, 0.99), respectively. Across individual drugs, exenatide twice-daily produced a larger BMI loss (1.65 kg/m^2^; 95% CI 1.04, 2.26), while glimepiride yielded a larger BMI increase (1.79 kg/m^2^; 95% CI 0.46, 3.12). Smaller BMI increases were observed with rosiglitazone and alogliptin, in the order of 0.91 kg/m^2^ (95% CI 0.48, 1.35) and 0.81 kg/m^2^ (95% CI 0.27, 1.35). The other drugs had a neutral effect on BMI (Table 1; Supplementary Figures S18–S25).

Across the drug classes, metformin, GLP-1RAs, and AGIs produced significantly greater BMI reductions than DPP-4is, TZDs, and SUs, whilst SGLT2is were superior to SUs. The largest difference was seen between metformin and SUs (−2.50 kg/m^2^; 95% CI −3.96, −1.04), with the smallest difference seen between AGIs and DPP-4is (−0.96 kg/m^2^; 95% CI −1.87, −0.05) (Table 2).

#### 3.1.4. Total Cholesterol

A total of 71 trials (12,014 patients) provided data on TC. Only GLP-1RAs, metformin, and AGIs reported significant TC reductions after removing the PBO/LI effects. The largest reductions were evident with GLP-1RAs (0.42 mmol/L; 95% CI 0.22, 0.61), ranging from 0.28 mmol/L (95% CI 0.06, 0.50) with exenatide twice-daily to 0.56 mmol/L (95% CI 0.34, 0.78) with liraglutide. AGIs conferred the smallest reductions (0.29 mmol/L; 95% CI 0.00, 0.58), although there were large differences within AGIs, with a 0.34 mmol/L (95% CI 0.03, 0.65) reduction with acarbose vs. no significant reduction with voglibose. Conversely, SGLT2is reported significant TC increases (0.22 mmol/L; 95% CI 0.13, 0.31), by 0.20 mmol/L (95% CI 0.06, 0.34) with empagliflozin and 0.30 mmol/L (95% CI 0.19, 0.40) with canagliflozin. Within DPP-4is, although alogliptin yielded TC loss (0.19 mmol/L; 95% CI 0.07, 0.31), sitagliptin produced TC increase (0.06 mmol/L; 95% CI 0.01, 0.11) (Table 1; Supplementary Figures S26–S33).

Across the drug classes, GLP-1RAs and metformin showed significantly greater TC reductions than DPP-4is, TZDs, and SGLT2is, ranging from 0.26 mmol/L (95% CI 0.08, 0.44) with metformin vs. DPP-4is to 0.64 mmol/L (95% CI 0.43, 0.86) with GLP-1RAs vs. SGLT2is. In addition, AGIs and DPP-4is were superior to SGLT2is (Table 2).

#### 3.1.5. High Density Lipoprotein-Cholesterol

A total of 72 trials (12,578 patients) provided data on HDL-C. After removing the PBO/LI effects, the greatest HDL-C increases were seen with TZDs (0.12 mmol/L; 95% CI 0.07, 0.17); although certain intraclass differences were noted, with a 0.06 mmol/L (95% CI 0.03, 0.08) increase with rosiglitazone vs. 0.18 mmol/L (95% CI 0.09, 0.26) increase with pioglitazone. The smallest increase was evident with metformin (0.05 mmol/L; 95% CI 0.02, 0.09). With the exception of dapagliflozin, SGLT2is also increased HDL-C (0.09 mmol/L; 95% CI 0.07, 0.11), in the order of 0.08 mmol/L (95% CI 0.07, 0.10) with empagliflozin and 0.10 mmol/L (95% CI 0.07, 0.12) with canagliflozin. Although no significant HDL-C effect was seen with exenatide twice-daily, liraglutide showed a similar HDL-C increase to TZDs (Table 1; Supplementary Figures S34–S41).

Across the drug classes, TZDs produced significantly greater HDL-C increases than metformin, DPP-4is, and GLP-1RAs by 0.07~0.09 mmol/L. In addition, SGLT2is worked better than DPP-4is and GLP-1RAs (Table 2).

#### 3.1.6. Systolic Blood Pressure

A total of 35 trials (9553 patients) provided data on SBP. Only SGLT2is and GLP-1RAs showed significant SBP reductions after removing the PBO/LI effects. SGLT2is produced relatively larger reductions (4.18 mmHg; 95% CI 3.53, 4.84), ranging from 3.29 mmHg (95% CI 2.25, 4.34) with empagliflozin to 5.36 mmHg (95% CI 4.14, 6.59) with canagliflozin. GLP-1RAs yielded smaller reductions (2.98 mmHg; 95% CI 1.30, 4.67), with reductions of 2.74 mmHg (95% CI 0.25, 5.24) with liraglutide vs. 3.19 mmHg (95% CI 0.90, 5.47) with exenatide twice-daily (Table 1; Supplementary Figures S42–S49).

Across the drug classes, SGLT2is conferred significantly greater SBP reductions than metformin, DPP-4is, and TZDs, ranging from 2.68 mmHg (95% CI 0.30, 5.06) vs. metformin to 4.96 mmHg (95% CI 1.74, 8.18) vs. TZDs. In addition, GLP-1RAs were superior to DPP-4is and TZDs (Table 2).

### 3.2. Hypoglycemia

A total of 103 trials (28,678 patients) provided data on hypoglycemia. The relative risk increases vs. PBO/LI were highest with SUs (5.44; 95% CI 2.11, 14.02), although only glipizide (7.11; 95% CI 2.18, 23.24) reported a significantly increased hypoglycemia risk. In addition, alogliptin (2.97; 95% CI 1.00, 8.77) significantly increased hypoglycemia risk, while empagliflozin (0.31; 95% CI 0.14, 0.71) reduced hypoglycemia risk compared with PBO/LI. The risks for other drugs were generally low (Table 1; Supplementary Figures S50–S57).

Across the drug classes, SUs showed significantly higher hypoglycemia risks than all other drug classes except NIDEs, by a RR ranging from 3.45 (95% CI 1.09, 10.91) vs. GLP-1RAs to 11.08 (95% CI 3.33, 36.87) vs. TZDs. In addition, metformin had higher risks than DPP-4is and TZDs. GLP-1RAs had higher risks than TZDs (Table 2).

### 3.3. Mortality and Vascular Outcomes

A total of 116 trials (24,976 patients) provided data on mortality, while most of them (101/116) reported that no death occurred during the trial. For the fatal events, most were considered by the investigator as not related to the study drug. A total of 32 trials (11,397 patients) provided data on vascular outcomes, 11 of which reported that no vascular events occurred. The main events reported were myocardial infarction (17 trials), heart failure (10 trials), stroke (9 trials), and diabetic nephropathy (9 trials). Compared with PBO/LI, all drugs had a neutral effect on mortality, total vascular events, myocardial infarction, heart failure, stroke, and diabetic nephropathy (Table 1; Supplementary Figures S58–S95).

Across the drug classes, there was no significant difference observed regarding mortality, total vascular events, myocardial infarction, heart failure, stroke, or diabetic nephropathy (Table 2; Supplementary Table S4).

### 3.4. Discontinuation

A total of 152 trials (34,569 patients) provided data on AE-induced discontinuations, 77 of which reported that no AE-induced discontinuation occurred during the trial. Overall, compared with PBO/LI, AGIs (2.57; 95% CI 1.64, 4.03) yielded significantly higher risk of AE-induced discontinuations, although large differences existed within AGIs, with 5.37 (95% CI 2.11, 13.69) times higher risk with miglitol vs. no risk increase with voglibose (0.92; 95% CI 0.19, 4.46). Conversely, linagliptin (0.55; 95% CI 0.30, 0.99) and empagliflozin (0.56; 95% CI 0.36, 0.87) significantly reduced the risk (Table 1; Supplementary Figures S96–S103).

Across the drug classes, AGIs conferred significantly higher risk of AE-induced discontinuations than TZDs (2.05; 95% CI 1.09, 3.85), metformin (2.49; 95% CI 1.43, 4.35), DPP-4is (2.80; 95% CI 1.70, 4.61), or SGLT2is (2.89; 95% CI 1.65, 5.07). No significant difference was seen across other comparisons (Table 2).

### 3.5. Sensitivity Analysis

The sensitivity analyses results did not significantly change the overall results (Supplementary Figures S104–S110).

## 4. Discussion

Evidence-based healthcare decision-making needs comparisons of all relevant competing therapies, on a full range of intermediate outcomes (e.g., blood glucose, blood lipids, and blood pressure), important AEs (e.g., hypoglycemia), and long-term outcomes (e.g., vascular events and mortality). This study identified important interclass and intraclass differences in these outcomes across all available glucose-lowering drugs when used as initial monotherapy for type 2 diabetes, to help decision makers rationally choose an alternative initial drug when metformin is contraindicated or intolerant.

The core of managing type 2 diabetes is to control blood glucose [3,4]. Intensive glucose control plays an important role in reducing the risks of diabetes-related vascular events and death [200]. This study showed that SUs generated the greatest HbA1c reductions, followed by GLP-1RAs, metformin, TZDs, SGLT2is, DPP-4is, AGIs, and NIDEs; and the HbA1c reductions observed were around the recommendation of clinical guidelines [3]. This is partly consistent with the results of Sherifali’s (2010) study, which found SUs to have greater HbA1c effect than metformin, TZDs, DPP-4is, AGIs, and NIDEs by synthesizing 61 English-language studies that compared an oral drug with placebo in patients with or without a background of other drugs [201]. Regarding FPG effects, SUs were also shown to have the largest FPG reductions, followed by TZDs, metformin, GLP-1RAs, SGLT2is, AGIs, DPP-4is, and NIDEs. In addition, we found the HbA1c reduction with liraglutide (1.17%) was more similar to SUs (1.39%) than other GLP-1RAs, such as lixisenatide (0.60%). We also found large differences in FPG reductions within GLP-1RAs, with 1.97 mmol/L for liraglutide vs. 0.97 mmol/L for lixisenatide. These findings confirmed important intraclass differences in glucose effect across drugs. Thus, optimal therapeutic decisions should also consider the comparative effects across individual drugs, especially when there is a need to choose from drugs belonging to the same classes.

Overweight or obesity is a common comorbidity of type 2 diabetes (85%) [202]. Weight gain may increase medical costs, while weight loss can improve cardiovascular risk factors, and reduce mortality and costs in patients with both type 2 diabetes and overweight/obesity [203,204,205,206]. This study reported that metformin and GLP-1RAs reduced BMI; while NIDEs, AGIs, DPP-4is, and SGLT2is maintained BMI; and SUs and TZDs increased BMI. The results were roughly consistent with that of Maruthur’s (2016) study, which compared the weight effect across monotherapies and metformin-based combinations of six drug classes from 85 English-language studies, and found weight was maintained or reduced with metformin, GLP-1RAs, DPP-4is, and SGLT2is, but increased with SUs and TZDs [207].

Dyslipidemia and/or hypertension, as independent risk factors for cardiovascular diseases, are also common comorbidities of type 2 diabetes (72%) [3,6]. Patients with type 2 diabetes, dyslipidemia, and hypertension are six times more likely to have cardiovascular diseases compared to those with diabetes alone [6]. Their integrated controls play important roles in reducing vascular events and costs, especially for those with long diabetes duration, old age, a history of cardiovascular diseases, or multiple risk factors [3]. This study showed that GLP-1RAs, AGIs, and metformin reduced TC, but SGLT2is increased TC; GLP-1RAs were superior to other drug classes in reducing TC. We also noted that within DPP-4is, alogliptin and sitagliptin showed opposite TC effects, confirming important intraclass differences across drugs. Regarding HDL-C effects, only TZDs, SGLT2is, and metformin increased HDL-C, and TZDs were the most efficacious drugs. This result is partly consistent with that of Bolen’s (2007) study, which found TZDs to have beneficial effects on HDL-C compared with other drugs by synthesizing 53 English-language studies on comparing monotherapies or combination therapies, although it did not include DPP-4is, SGLT2is, or GLP-1RAs [208].

Blood pressure lowering is related to improved mortality and other clinical outcomes for type 2 diabetes patients, and even a small reduction of 2.4 mmHg in SBP has an effect in reducing cardiovascular events [209,210]. As this study showed that SGLT2is and GLP-1RAs conferred SBP reductions by 2.74~5.36 mmHg, they may have beneficial effects on cardiovascular events. Our findings roughly correspond to that of Tsapas’s (2021) study, which reported that treatment with SGLT2is or GLP-1RAs with or without a metformin-based background therapy were more efficacious than other drug classes in reducing SBP by estimating 204 English-language studies [211].

A patient-centered approach recommends minimizing AEs, vascular events, and mortality beyond optimizing efficacy. Hypoglycemia as a common AE in treating type 2 diabetes is associated with multiple complications and high economic burden, and fear of it may reduce treatment adherence and prevent optimal glucose control [212,213]. This study showed that SUs had higher hypoglycemia risks than other drug classes, which is consistent with the previous studies [207,208]. In addition, large intraclass differences were noted regarding hypoglycemia risk. For example, within DPP-4is, alogliptin was reported to increase the risk but linagliptin reduced the risk. Overall, amongst all monotherapies, only GLP-1RAs produced a comprehensive beneficial effect on HbA1c, FPG, BMI, TC, and SBP, while not increasing hypoglycemia risk. As GLP-1RAs did not have a significant beneficial effect on HDL-C, while TZDs conferred the best effect on HDL-C, GLP-1RAs and TZDs may be the best mixed therapy to control HbA1c, FPG and lipid profile all together for patients with contraindications or intolerance to metformin. In terms of long-term outcomes, no significant difference was observed regarding total vascular events, myocardial infarction, heart failure, stroke, diabetic nephropathy, or mortality across the drug classes. This may be because when patients start the initial drug monotherapy they are most likely in the early stages of diabetes and may not develop serious vascular events in the short term. However, most of the included studies were short-term trials, lasting less than one year, thus with limited ability to observe long-term vascular outcomes and deaths.

Only a few systematic reviews were detected to have compared several drug classes, however, they were not focused on monotherapy, not covered all available drug classes, and only included English-language studies [201,207,208,211]. Thus, our study has noteworthy strengths in that it is the first attempt to comprehensively and simultaneously estimate and compare the treatment effects of all available glucose-lowering drugs when used as initial monotherapy for type 2 diabetes across multiple clinically meaningful intermediate and long-term outcomes, by merging data from English- and Chinese-language studies. The inclusion of 62 Chinese-language studies allowed the inclusion of populations and drugs that had not been studied previously in other settings. Our observations expanded the knowledge by supplementing with evidence from China. Our results may enable decision makers to better understand the similarities and differences across drugs, as to make comparisons between drugs at a glance when choosing an alternative monotherapy to metformin for type 2 diabetes patients.

Certain limitations should be acknowledged. First, from the methodological point of view, a meta-analysis has some weakness, including high risk of bias within studies, potential publication bias, and clinical heterogeneity. Second, most of the included trials were short-term trials, lasting less than one year, with limited ability to observe long-term clinical outcomes, such as vascular events and mortality. Ideally, as therapeutic decision-making should depend on long-term effectiveness, the generalizability of our results may be limited due to paucity of long-term evidence. Third, we only searched PubMed, Web of Science, Embase, CNKI, Chongqing VIP, and WanFang Data for eligible RCTs, thus RCTs indexed only in other databases (e.g., Scopus) may not be included in our review, which may induce potential bias.

## 5. Conclusions

Rational choice of drugs should be individualized, based on unique patient characteristics and benefit-risk profile of each drug. When choosing an alternative drug as initial monotherapy for type 2 diabetes patients with contraindications or intolerance to metformin, our results suggest a potential treatment hierarchy, with GLP-1RAs being preferred in terms of their favorable efficacy and safety profiles. In addition, SUs are most suggested for patients whose HbA1c or FPG is far away from the target, but least suggested for those wishing to minimize hypoglycemia. GLP-1RAs are most suggested for patients for whom BMI management or TC management is an emphasis. TZDs are most suggested for patients for whom HDL-C control is a priority. SGLT2is are most suggested for patients having a need to reduce SBP. While AGIs are least suggested for patients wishing to avoid AE-induced discontinuations. Our observations also corroborated the intraclass differences in treatment effects among drugs. Future studies should pay more attention to long-term studies to obtain more precise data on vascular outcomes and mortality. Moreover, this study also provided comparative and systematic clinical data for international researchers to conduct cost-effectiveness modelling studies, which will add long-term costs and effectiveness evidence for the rational choice of initial monotherapies alternative to metformin and improving the diabetes management as well as healthcare resource allocation.

## Figures and Tables

**Table 1:** Treatment effects of glucose-lowering drugs after removing the effect of placebo/lifestyle intervention.

**Glucose-Lowering Drug**	**HbA1c, %**	**FPG, mmol/L**	**BMI, kg/m^2^**	**TC, mmol/L**	**HDL-C, mmol/L**	**SBP, mmHg**	**Hypoglycemia**
Metformin	−0.96 (−1.16, −0.76) *	−1.65 (−2.02, −1.27) *	−1.28 (−2.26, −0.31) *	−0.30 (−0.47, −0.14) *	0.05 (0.02, 0.09) *	−1.50 (−3.78, 0.79)	1.53 (0.98, 2.40)
SUs	−1.39 (−1.63, −1.16) *	−2.70 (−3.18, −2.23) *	1.22 (0.13, 2.31) *	−0.40 (−1.20, 0.40)	−0.00 (−0.18, 0.18)	1.84 (−4.57, 8.25)	5.44 (2.11, 14.02) *
Glyburide	−1.50 (−2.69, −0.30) *	−2.35 (−3.59, −1.12) *	0.27 (−1.48, 2.03)	−0.80 (−1.94, 0.34)	−0.20 (−0.63, 0.23)	—	—
Glimepiride	−1.36 (−1.57, −1.16) *	−2.41 (−3.09, −1.73) *	1.79 (0.46, 3.12) *	−0.22 (−1.60, 1.17)	0.12 (−0.04, 0.29)	1.84 (−4.57, 8.25)	2.88 (0.45, 18.58)
Gliclazide	−1.40 (−2.70, −0.10) *	−2.22 (−3.47, −0.97) *	—	−0.75 (−1.84, 0.34)	0.07 (−0.08, 0.22)	—	5.00 (0.25, 99.95)
Glipizide	−1.47 (−1.87, −1.06) *	−3.02 (−3.85, −2.20) *	—	0.20 (−3.19, 3.59)	0.41 (−1.64, 2.47)	—	7.11 (2.18, 23.24) *
TZDs	−0.89 (−1.04, −0.73) *	−1.91 (−2.23, −1.60) *	0.63 (0.26, 0.99) *	0.01 (−0.19, 0.22)	0.12 (0.07, 0.17) *	0.78 (−2.37, 3.93)	0.49 (0.23, 1.03)
Rosiglitazone	−0.68 (−0.98, −0.38) *	−1.73 (−2.32, −1.14) *	0.91 (0.48, 1.35) *	0.25 (0.03, 0.46) *	0.06 (0.03, 0.08) *	2.43 (−1.55, 6.42)	0.43 (0.15, 1.26)
Pioglitazone	−1.00 (−1.17, −0.82) *	−2.01 (−2.34, −1.67) *	0.38 (−0.07, 0.82)	−0.09 (−0.32, 0.14)	0.18 (0.09, 0.26) *	−1.79 (−6.33, 2.74)	0.55 (0.20, 1.53)
NIDEs	−0.44 (−0.69, −0.20) *	−0.75 (−1.04, −0.45) *	0.08 (−1.29, 1.44)	0.21 (−0.39, 0.81)	0.08 (−0.21, 0.37)	−5.98 (−12.33, 0.37)	1.37 (0.34, 5.59)
Repaglinide	−0.45 (−0.81, −0.09) *	−0.64 (−1.27, −0.01) *	—	—	—	—	0.97 (0.14, 6.77)
Nateglinide	−0.45 (−0.79, −0.10) *	−0.70 (−1.04, −0.36) *	0.08 (−1.29, 1.44)	0.21 (−0.39, 0.81)	0.08 (−0.21, 0.37)	−5.98 (−12.33, 0.37)	2.00 (0.26, 15.33)
AGIs	−0.62 (−0.79, −0.45) *	−1.19 (−1.73, −0.64) *	−0.49 (−1.26, 0.28)	−0.29 (−0.58, −0.00) *	0.03 (−0.12, 0.17)	−1.40 (−4.71, 1.90)	0.86 (0.51, 1.45)
Acarbose	−0.74 (−0.96, −0.52) *	−1.17 (−1.83, −0.50) *	−0.60 (−1.66, 0.46)	−0.34 (−0.65, −0.03) *	0.07 (−0.11, 0.25)	−1.40 (−4.71, 1.90)	1.19 (0.34, 4.23)
Voglibose	−0.20 (−0.33, −0.07) *	−1.78 (−3.58, 0.02)	0.10 (−0.13, 0.33)	−0.15 (−0.92, 0.62)	−0.17 (−0.48, 0.14)	—	0.93 (0.10, 8.79)
Miglitol	−0.53 (−0.85, −0.21) *	−0.01 (−0.88, 0.86)	—	—	—	—	0.79 (0.44, 1.44)
DPP−4is	−0.63 (−0.68, −0.58) *	−0.94 (−1.03, −0.85) *	0.47 (−0.01, 0.95)	−0.04 (−0.11, 0.02)	0.03 (−0.01, 0.06)	0.02 (−1.10, 1.14)	0.89 (0.67, 1.18)
Sitagliptin	−0.73 (−0.82, −0.65) *	−1.07 (−1.20, −0.95) *	0.10 (−1.24, 1.44)	0.06 (0.01, 0.11) *	0.06 (−0.02, 0.14)	0.18 (−1.27, 1.64)	0.79 (0.52, 1.20)
Saxagliptin	−0.52 (−0.61, −0.44) *	−0.83 (−1.00, −0.67) *	−0.46 (−2.04, 1.12)	—	—	—	1.21 (0.52, 2.81)
Vildagliptin	−0.48 (−0.57, −0.38) *	−0.56 (−0.82, −0.30) *	−0.58 (−2.09, 0.93)	−0.12 (−0.83, 0.59)	—	—	1.07 (0.54, 2.13)
Linagliptin	−0.68 (−0.79, −0.58) *	−0.94 (−1.16, −0.73) *	—	−0.06 (−0.21, 0.09)	0.03 (−0.06, 0.12)	−1.74 (−4.75, 1.26)	0.52 (0.26, 1.01)
Alogliptin	−0.68 (−0.76, −0.61) *	−1.07 (−1.27, −0.86) *	0.81 (0.27, 1.35) *	−0.19 (−0.31, −0.07) *	−0.00 (−0.04, 0.03)	0.59 (−1.59, 2.76)	2.97 (1.00, 8.77) *
SGLT2is	−0.80 (−0.87, −0.72) *	−1.58 (−1.81, −1.36) *	−0.60 (−1.89, 0.69)	0.22 (0.13, 0.31) *	0.09 (0.07, 0.11) *	−4.18 (−4.84, −3.53) *	0.86 (0.55, 1.37)
Dapagliflozin	−0.68 (−0.77, −0.59) *	−1.27 (−1.49, −1.06) *	−0.60 (−1.89, 0.69)	−0.02 (−0.24, 0.20)	0.03 (−0.09, 0.15)	−3.89 (−5.02, −2.75) *	1.29 (0.70, 2.36)
Empagliflozin	−0.79 (−0.86, −0.72) *	−1.84 (−1.97, −1.72) *	—	0.20 (0.06, 0.34) *	0.08 (0.07, 0.10) *	−3.29 (−4.34, −2.25) *	0.31 (0.14, 0.71) *
Canagliflozin	−0.99 (−1.06, −0.92) *	−2.09 (−2.31, −1.87) *	—	0.30 (0.19, 0.40) *	0.10 (0.07, 0.12) *	−5.36 (−6.59, −4.14) *	1.57 (0.79, 3.10)
GLP−1RAs	−0.99 (−1.20, −0.78) *	−1.64 (−2.00, −1.28) *	−1.05 (−1.81, −0.29) *	−0.42 (−0.61, −0.22) *	0.03 (−0.01, 0.06)	−2.98 (−4.67, −1.30) *	1.57 (0.82, 3.02)
Exenatide twice-daily	−0.64 (−0.82, −0.47) *	−1.13 (−1.49, −0.77) *	−1.65 (−2.26, −1.04) *	−0.28 (−0.50, −0.06) *	0.01 (−0.01, 0.03)	−3.19 (−5.47, −0.90) *	3.36 (0.84, 13.52)
Liraglutide	−1.17 (−1.47, −0.87) *	−1.97 (−2.47, −1.47) *	−0.80 (−1.66, 0.07)	−0.56 (−0.78, −0.34) *	0.13 (0.05, 0.21) *	−2.74 (−5.24, −0.25) *	1.37 (0.58, 3.25)
Lixisenatide	−0.60 (−0.83, −0.37) *	−0.97 (−1.47, −0.48) *	—	—	—	—	1.04 (0.25, 4.29)
**Glucose-Lowering Drug**	**Death**	**Total** **Vascular Events**	**Myocardial Infarction**	**Heart Failure**	**Stroke**	**Diabetic Nephropathy**	**AE-Induced Discontinuations**
Metformin	0.88 (0.46, 1.69)	0.91 (0.22, 3.73)	0.98 (0.10, 9.30)	0.98 (0.10, 9.30)	0.98 (0.10, 9.30)	1.59 (0.20, 12.85)	1.03 (0.74, 1.43)
SUs	1.10 (0.27, 4.52)	0.46 (0.03, 7.00)	1.41 (0.06, 33.26)	—	0.16 (0.01, 3.70)	—	2.25 (0.74, 6.81)
Glyburide	1.09 (0.07, 16.30)	—	—	—	—	—	2.24 (0.31, 16.50)
Glimepiride	1.16 (0.15, 9.23)	0.46 (0.03, 7.00)	1.41 (0.06, 33.26)	—	0.16 (0.01, 3.70)	—	0.99 (0.10, 9.40)
Gliclazide	1.00 (0.02, 48.82)	—	—	—	—	—	1.00 (0.02, 48.82)
Glipizide	1.02 (0.02, 50.81)	—	—	—	—	—	4.64 (0.74, 28.95)
TZDs	0.95 (0.48, 1.90)	1.44 (0.38, 5.39)	0.82 (0.13, 5.06)	0.97 (0.19, 4.92)	0.96 (0.13, 7.21)	0.97 (0.02, 48.35)	1.25 (0.81, 1.95)
Rosiglitazone	0.94 (0.29, 3.00)	1.00 (0.02, 46.40)	1.00 (0.02, 46.40)	1.00 (0.02, 46.40)	—	—	0.97 (0.43, 2.23)
Pioglitazone	0.96 (0.41, 2.26)	1.51 (0.37, 6.17)	0.78 (0.10, 6.12)	0.96 (0.16, 5.78)	0.96 (0.13, 7.21)	0.97 (0.02, 48.35)	1.38 (0.82, 2.33)
NIDEs	0.96 (0.20, 4.68)	—	—	—	—	—	0.97 (0.24, 3.81)
Repaglinide	0.97 (0.14, 6.77)	—	—	—	—	—	0.97 (0.14, 6.77)
Nateglinide	0.94 (0.06, 14.46)	—	—	—	—	—	0.96 (0.14, 6.67)
AGIs	1.07 (0.41, 2.78)	1.55 (0.19, 12.51)	1.92 (0.16, 22.74)	—	—	—	2.57 (1.64, 4.03) *
Acarbose	0.97 (0.28, 3.31)	—	—	—	—	—	2.15 (1.23, 3.75) *
Voglibose	0.93 (0.10, 8.78)	0.90 (0.02, 45.04)	—	—	—	—	0.92 (0.19, 4.46)
Miglitol	1.60 (0.20, 12.92)	1.92 (0.16, 22.74)	1.92 (0.16, 22.74)	—	—	—	5.37 (2.11, 13.69) *
DPP-4is	0.89 (0.51, 1.58)	0.82 (0.47, 1.41)	0.47 (0.19, 1.16)	1.00 (0.14, 7.05)	0.99 (0.14, 7.00)	0.98 (0.14, 6.94)	0.92 (0.74, 1.14)
Sitagliptin	0.82 (0.30, 2.20)	0.68 (0.28, 1.66)	0.56 (0.09, 3.53)	1.03 (0.06, 16.28)	0.34 (0.01, 8.36)	—	0.89 (0.62, 1.28)
Saxagliptin	1.44 (0.49, 4.22)	0.71 (0.29, 1.73)	0.33 (0.08, 1.30)	—	—	0.33 (0.01, 8.15)	1.28 (0.68, 2.42)
Vildagliptin	0.48 (0.10, 2.36)	—	—	—	—	—	1.08 (0.73, 1.60)
Linagliptin	0.85 (0.16, 4.50)	0.95 (0.21, 4.25)	0.31 (0.04, 2.52)	—	—	—	0.55 (0.30, 0.99) *
Alogliptin	0.79 (0.18, 3.45)	2.19 (0.39, 12.23)	1.88 (0.16, 22.23)	0.97 (0.06, 15.40)	1.87 (0.16, 22.19)	1.87 (0.16, 22.19)	0.82 (0.42, 1.60)
SGLT2is	0.81 (0.41, 1.60)	1.00 (0.45, 2.21)	0.79 (0.17, 3.64)	1.00 (0.10, 9.50)	0.58 (0.12, 2.88)	1.53 (0.56, 4.18)	0.89 (0.63, 1.24)
Dapagliflozin	1.07 (0.41, 2.80)	1.19 (0.38, 3.68)	—	—	—	1.19 (0.38, 3.68)	1.66 (0.84, 3.27)
Empagliflozin	0.53 (0.15, 1.81)	0.81 (0.21, 3.09)	0.64 (0.08, 5.18)	—	0.34 (0.04, 3.27)	3.95 (0.44, 35.38)	0.56 (0.36, 0.87) *
Canagliflozin	0.77 (0.17, 3.55)	1.00 (0.10, 9.50)	1.00 (0.10, 9.50)	1.00 (0.10, 9.50)	1.00 (0.10, 9.50)	—	1.78 (0.78, 4.07)
GLP-1RAs	0.89 (0.33, 2.43)	0.39 (0.10, 1.57)	0.67 (0.04, 10.13)	0.67 (0.04, 10.13)	0.67 (0.04, 10.13)	0.80 (0.05, 12.41)	1.23 (0.60, 2.54)
Exenatide twice-daily	0.96 (0.14, 6.73)	0.86 (0.02, 40.01)	0.86 (0.02, 40.01)	0.86 (0.02, 40.01)	0.86 (0.02, 40.01)	0.86 (0.02, 40.01)	2.63 (0.49, 14.04)
Liraglutide	0.86 (0.27, 2.79)	0.35 (0.08, 1.55)	0.51 (0.01, 25.66)	0.51 (0.01, 25.66)	0.51 (0.01, 25.66)	0.75 (0.02, 37.38)	0.63 (0.25, 1.61)
Lixisenatide	—	—	—	—	—	—	4.01 (0.85, 18.83)

For HbA1c, FPG, BMI, TC, HDL-C and SBP, data are WMD (95% CIs); for hypoglycemia, death, AE-induced discontinuations, total vascular events, myocardial infarction, heart failure, stroke, and diabetic nephropathy, data are RR (95% CIs). * Statistically significant differences. Table 1 represents a summary from Supplementary Figures S2–S103. Abbreviations: AE, adverse event; AGIs, α-glucosidase inhibitors; BMI, body mass index; CI, confidence interval; DPP-4is, dipeptidyl peptidase-4 inhibitors; FPG, fasting plasma glucose; GLP-1RAs, glucagon-like peptide-1 receptor agonists; HbA1c, hemoglobin Alc; HDL-C, high-density lipoprotein-cholesterol; LI, lifestyle intervention; NIDEs, glinides; PBO, placebo; RR, risk ratio; SBP, systolic blood pressure; SGLT2is, sodium-glucose cotransporter 2 inhibitors; SUs, sulfonylureas; TC, total cholesterol; TZDs, thiazolidinediones; WMD, weighted mean difference.

**Table 2:** Treatment effects of glucose-lowering drugs compared with each other.

**HbA1c, % (Left Lower Half)**							**FPG, mmol/L (Right Upper Half)**
Metformin	1.05 (0.45, 1.66) *	0.26 (−0.23, 0.75)	−0.90 (−1.38, −0.42) *	−0.46 (−1.12, 0.20)	−0.71 (−1.10, −0.32) *	−0.07 (−0.51, 0.37)	−0.01 (−0.53, 0.51)
0.43 (0.12, 0.74) *	SUs	−0.79 (−1.36, −0.22) *	−1.95 (−2.51, −1.39) *	−1.51 (−2.23, −0.79) *	−1.76 (−2.24, −1.28) *	−1.12 (−1.65, −0.59) *	−1.06 (−1.66, −0.46) *
−0.07 (−0.32, 0.18)	−0.50 (−0.78, −0.22) *	TZDs	−1.16 (−1.59, −0.73) *	−0.72 (−1.35, −0.09) *	−0.97 (−1.30, −0.64) *	−0.33 (−0.72, 0.06)	−0.27 (−0.75, 0.21)
−0.52 (−0.84, −0.20) *	−0.95 (−1.29, −0.61) *	−0.45 (−0.74, −0.16) *	NIDEs	0.44 (−0.18, 1.06)	0.19 (−0.12, 0.50)	0.83 (0.46, 1.20) *	0.89 (0.43, 1.36) *
−0.34 (−0.60, −0.08) *	−0.77 (−1.06, −0.48) *	−0.27 (−0.50, −0.04) *	0.18 (−0.12, 0.48)	AGIs	−0.25 (−0.80, 0.30)	0.39 (−0.20, 0.98)	0.45 (−0.20, 1.10)
−0.33 (−0.54, −0.12) *	−0.76 (−1.00, −0.52) *	−0.26 (−0.42, −0.10) *	0.19 (−0.06, 0.44)	0.01 (−0.17, 0.19)	DPP−4is	0.64 (0.40, 0.88) *	0.70 (0.33, 1.07) *
−0.16 (−0.37, 0.05)	−0.59 (−0.84, −0.34) *	−0.09 (−0.26, 0.08)	0.36 (0.10, 0.62) *	0.18 (−0.01, 0.37)	0.17 (0.08, 0.26) *	SGLT2is	0.06 (−0.37, 0.49)
0.03 (−0.26, 0.32)	−0.40 (−0.72, −0.09) *	0.10 (−0.16, 0.36)	0.55 (0.23, 0.87) *	0.37 (0.10, 0.64) *	0.36 (0.14, 0.58) *	0.19 (−0.03, 0.41)	GLP−1RAs
**BMI, kg/m^2^ (Left Lower Half)**							**TC, mmol/L (Right Upper Half)**
Metformin	0.10 (−0.72, 0.92)	−0.31 (−0.57, −0.05) *	−0.51 (−1.13, 0.11)	−0.01 (−0.34, 0.32)	−0.26 (−0.44, −0.08) *	−0.52 (−0.71, −0.33) *	0.12 (−0.14, 0.38)
−2.50 (−3.96, −1.04) *	SUs	−0.41 (−1.24, 0.42)	−0.61 (−1.61, 0.39)	−0.11 (−0.96, 0.74)	−0.36 (−1.16, 0.44)	−0.62 (−1.43, 0.19)	0.02 (−0.80, 0.84)
−1.91 (−2.95, −0.87) *	0.59 (−0.56, 1.74)	TZDs	−0.20 (−0.83, 0.43)	0.30 (−0.06, 0.66)	0.05 (−0.17, 0.27)	−0.21 (−0.43, 0.01)	0.43 (0.15, 0.71) *
−1.36 (−3.04, 0.32)	1.14 (−0.61, 2.89)	0.55 (−0.86, 1.96)	NIDEs	0.50 (−0.17, 1.17)	0.25 (−0.35, 0.85)	−0.01 (−0.62, 0.60)	0.63 (−0.00, 1.26)
−0.79 (−2.03, 0.45)	1.71 (0.38, 3.05) *	1.12 (0.27, 1.97) *	0.57 (−1.00, 2.14)	AGIs	−0.25 (−0.55, 0.05)	−0.51 (−0.81, −0.21) *	0.13 (−0.22, 0.48)
−1.75 (−2.84, −0.66) *	0.75 (−0.44, 1.94)	0.16 (−0.44, 0.76)	−0.39 (−1.84, 1.06)	−0.96 (−1.87, −0.05) *	DPP−4is	−0.26 (−0.37, −0.15) *	0.38 (0.17, 0.59) *
−0.68 (−2.30, 0.94)	1.82 (0.13, 3.51) *	1.23 (−0.11, 2.57)	0.68 (−1.20, 2.56)	0.11 (−1.39, 1.61)	1.07 (−0.31, 2.45)	SGLT2is	0.64 (0.43, 0.86) *
−0.23 (−1.47, 1.01)	2.27 (0.94, 3.60) *	1.68 (0.84, 2.52) *	1.13 (−0.43, 2.69)	0.56 (−0.52, 1.64)	1.52 (0.62, 2.42) *	0.45 (−1.05, 1.95)	GLP−1RAs
**HDL-C, mmol/L (Left Lower Half)**							**SBP, mmHg (Right Upper Half)**
Metformin	−3.34 (−10.15, 3.47)	−2.28 (−6.17, 1.61)	4.48 (−2.27, 11.23)	−0.10 (−4.12, 3.92)	−1.52 (−4.07, 1.03)	2.68 (0.30, 5.06) *	1.48 (−1.36, 4.32)
0.05 (−0.13, 0.23)	SUs	1.06 (−6.08, 8.20)	7.82 (−1.20, 16.84)	3.24 (−3.97, 10.45)	1.82 (−4.69, 8.33)	6.02 (−0.42, 12.46)	4.82 (−1.81, 11.45)
−0.07 (−0.13, −0.01) *	−0.12 (−0.31, 0.07)	TZDs	6.76 (−0.33, 13.85)	2.18 (−2.39, 6.75)	0.76 (−2.58, 4.10)	4.96 (1.74, 8.18) *	3.76 (0.19, 7.33) *
−0.03 (−0.32, 0.26)	−0.08 (−0.42, 0.26)	0.04 (−0.25, 0.33)	NIDEs	−4.58 (−11.74, 2.58)	−6.00 (−12.45, 0.45)	−1.80 (−8.18, 4.58)	−3.00 (−9.57, 3.57)
0.02 (−0.13, 0.17)	−0.03 (−0.26, 0.20)	0.09 (−0.06, 0.24)	0.05 (−0.27, 0.37)	AGIs	−1.42 (−4.91, 2.07)	2.78 (−0.59, 6.15)	1.58 (−2.13, 5.29)
0.02 (−0.03, 0.07)	−0.03 (−0.21, 0.15)	0.09 (0.03, 0.15) *	0.05 (−0.24, 0.34)	0.00 (−0.15, 0.15)	DPP−4is	4.20 (2.90, 5.50) *	3.00 (0.98, 5.02) *
−0.04 (−0.08, 0.00)	−0.09 (−0.27, 0.09)	0.03 (−0.02, 0.08)	−0.01 (−0.30, 0.28)	−0.06 (−0.21, 0.09)	−0.06 (−0.10, −0.02) *	SGLT2is	−1.20 (−3.01, 0.61)
0.02 (−0.03, 0.07)	−0.03 (−0.21, 0.15)	0.09 (0.03, 0.15) *	0.05 (−0.24, 0.34)	0.00 (−0.15, 0.15)	0.00 (−0.05, 0.05)	0.06 (0.02, 0.10) *	GLP-1RAs
**Hypoglycemia (Left Lower Half)**							**Death (Right Upper Half)**
Metformin	0.80 (0.17, 3.80)	0.93 (0.36, 2.39)	0.92 (0.17, 5.09)	0.83 (0.26, 2.63)	0.99 (0.42, 2.35)	1.09 (0.42, 2.79)	0.99 (0.30, 3.29)
0.28 (0.10, 0.81) *	SUs	1.15 (0.24, 5.55)	1.15 (0.14, 9.57)	1.03 (0.19, 5.67)	1.23 (0.27, 5.65)	1.36 (0.28, 6.51)	1.24 (0.22, 7.01)
3.13 (1.32, 7.42) *	11.08 (3.33, 36.87) *	TZDs	0.99 (0.18, 5.58)	0.89 (0.28, 2.90)	1.07 (0.44, 2.61)	1.18 (0.45, 3.09)	1.07 (0.32, 3.62)
1.12 (0.26, 4.90)	3.97 (0.73, 21.66)	0.36 (0.07, 1.76)	NIDEs	0.90 (0.14, 5.71)	1.08 (0.20, 5.78)	1.19 (0.21, 6.64)	1.08 (0.17, 7.05)
1.79 (0.89, 3.57)	6.33 (2.14, 18.73) *	0.57 (0.23, 1.42)	1.60 (0.36, 7.16)	AGIs	1.20 (0.39, 3.64)	1.32 (0.41, 4.27)	1.20 (0.30, 4.82)
1.73 (1.02, 2.95) *	6.14 (2.28, 16.51) *	0.55 (0.25, 1.22)	1.55 (0.37, 6.49)	0.97 (0.53, 1.76)	DPP-4is	1.10 (0.46, 2.67)	1.01 (0.32, 3.19)
1.78 (0.93, 3.38)	6.30 (2.20, 18.05) *	0.57 (0.24, 1.36)	1.59 (0.36, 6.96)	0.99 (0.49, 2.00)	1.03 (0.60, 1.76)	SGLT2is	0.91 (0.27, 3.07)
0.97 (0.44, 2.15)	3.45 (1.09, 10.91) *	0.31 (0.12, 0.84) *	0.87 (0.19, 4.10)	0.55 (0.24, 1.26)	0.56 (0.28, 1.15)	0.55 (0.25, 1.22)	GLP-1RAs
**Total Vascular Events (Left Lower Half)**							**AE-Induced Discontinuations (Right Upper Half)**
Metformin	0.46 (0.14, 1.46)	0.82 (0.48, 1.42)	1.07 (0.26, 4.37)	0.40 (0.23, 0.70) *	1.12 (0.76, 1.66)	1.16 (0.73, 1.85)	0.84 (0.38, 1.85)
1.99 (0.09, 42.75)	SUs	1.79 (0.54, 5.91)	2.33 (0.40, 13.59)	0.88 (0.27, 2.90)	2.45 (0.79, 7.58)	2.53 (0.80, 8.06)	1.82 (0.49, 6.85)
0.63 (0.09, 4.37)	0.32 (0.02, 6.59)	TZDs	1.30 (0.31, 5.48)	0.49 (0.26, 0.92) *	1.37 (0.84, 2.23)	1.41 (0.81, 2.46)	1.02 (0.44, 2.37)
—	—	—	NIDEs	0.38 (0.09, 1.59)	1.05 (0.26, 4.22)	1.09 (0.27, 4.46)	0.78 (0.17, 3.69)
0.59 (0.05, 7.30)	0.30 (0.01, 9.16)	0.93 (0.08, 10.98)	—	AGIs	2.80 (1.70, 4.61) *	2.89 (1.65, 5.07) *	2.08 (0.89, 4.88)
1.12 (0.25, 5.08)	0.56 (0.04, 9.07)	1.76 (0.42, 7.38)	—	1.90 (0.22, 16.48)	DPP-4is	1.03 (0.69, 1.54)	0.75 (0.35, 1.58)
0.91 (0.18, 4.61)	0.46 (0.03, 7.86)	1.44 (0.31, 6.74)	—	1.56 (0.17, 14.53)	0.82 (0.31, 2.15)	SGLT2is	0.72 (0.33, 1.60)
2.33 (0.32, 16.87)	1.17 (0.06, 24.97)	3.67 (0.54, 25.02)	—	3.96 (0.32, 48.69)	2.08 (0.47, 9.30)	2.55 (0.51, 12.66)	GLP-1RAs

Treatment estimates are WMD (95% CIs) (or RR (95% CIs)) of the column-defining treatment compared with the row-defining treatment for HbA1c, BMI (or hypoglycemia, total vascular events) (left lower half), and WMD > 0 (or RR > 1) favor the row-defining treatment. Treatment estimates are WMD (95% CIs) of the column-defining treatment compared with the row-defining treatment for HDL-C (left lower half), and WMD > 0 favor the column-defining treatment. Treatment estimates are WMD (95% CIs) (or RR (95% CIs)) of the row-defining treatment compared with the column-defining treatment for FPG, TC, SBP (or death, AE-induced discontinuations) (right upper half), and WMD > 0 (or RR > 1) favor the column-defining treatment. * Statistically significant differences. Abbreviations: AE, adverse event; AGIs, α-glucosidase inhibitors; BMI, body mass index; CI, confidence interval; DPP-4is, dipeptidyl peptidase-4 inhibitors; FPG, fasting plasma glucose; GLP-1RAs, glucagon-like peptide-1 receptor agonists; HbA1c, hemoglobin Alc; HDL-C, high-density lipoprotein-cholesterol; NIDEs, glinides; RR, risk ratio; SBP, systolic blood pressure; SGLT2is, sodium-glucose cotransporter 2 inhibitors; SUs, sulfonylureas; TC, total cholesterol; TZDs, thiazolidinediones; WMD, weighted mean difference.

## Data Availability

The datasets used and/or analyzed during the current study are available from the corresponding authors on reasonable request.

## Supplementary Materials

The following supporting information can be downloaded at: https://www.mdpi.com/article/10.3390/jcm11237094/s1.
