# Supplementary material for: Choice of Glucose-Lowering Drugs as Initial Monotherapy for Type 2 Diabetes Patients with Contraindications or Intolerance to Metformin: A Systematic Review and Meta-Analysis
Source: J Clin Med. 2022 Nov 30;11(23):7094. doi: 10.3390/jcm11237094 (PMC9740076; doi:10.3390/jcm11237094)
Supplement: Supplementary file 1 [file jcm-11-07094-s001.zip › jcm-2035854-supplementary.pdf]

Content

Tables

Table S1. Eligibility criteria 5
Table S2. Search terms and search strategies 6
Table S3. The characteristics and risk of bias of the included studies 8
Table S4. Treatment effects on vascular events of glucose-lowering drugs vs each other 24

Figures

Figure S1. Risk of publication bias across studies 25
Figure S2. Meta-analysis results for change in hemoglobin Alc (%) of metformin vs placebo/lifestyle intervention 26
Figure S3. Meta-analysis results for change in hemoglobin Alc (%) of sulfonylureas vs placebo/lifestyle intervention 27
Figure S4. Meta-analysis results for change in hemoglobin Alc (%) of thiazolidinediones vs placebo/lifestyle intervention 28
Figure S5. Meta-analysis results for change in hemoglobin Alc (%) of glinides vs placebo/lifestyle intervention 29
Figure S6. Meta-analysis results for change in hemoglobin Alc (%) of α-glucosidase inhibitors vs placebo/lifestyle intervention 30
Figure S7. Meta-analysis results for change in hemoglobin Alc (%) of dipeptidyl peptidase-4 inhibitors vs placebo/lifestyle intervention 32
Figure S8. Meta-analysis results for change in hemoglobin Alc (%) of sodium-glucose cotransporter-2 inhibitors vs placebo/lifestyle intervention 33
Figure S9. Meta-analysis results for change in hemoglobin Alc (%) of glucagon-like peptide-1 receptor agonists vs placebo/lifestyle intervention 34
Figure S10. Meta-analysis results for change in fasting plasma glucose (mmol/l) of metformin vs placebo/lifestyle intervention 35
Figure S11. Meta-analysis results for change in fasting plasma glucose (mmol/l) of sulfonylureas vs placebo/lifestyle intervention 36
Figure S12. Meta-analysis results for change in fasting plasma glucose (mmol/l) of thiazolidinediones vs placebo/lifestyle intervention 37
Figure S13. Meta-analysis results for change in fasting plasma glucose (mmol/l) of glinides vs placebo/lifestyle intervention 38
Figure S14. Meta-analysis results for change in fasting plasma glucose (mmol/l) of α-glucosidase inhibitors vs placebo/lifestyle intervention 39
Figure S15. Meta-analysis results for change in fasting plasma glucose (mmol/l) of dipeptidyl peptidase-4 inhibitors vs placebo/lifestyle intervention 41
Figure S16. Meta-analysis results for change in fasting plasma glucose (mmol/l) of sodium-glucose cotransporter-2 inhibitors vs placebo/lifestyle intervention 42
Figure S17. Meta-analysis results for change in fasting plasma glucose (mmol/l) of glucagon-like peptide-1 receptor agonists vs placebo/lifestyle intervention 43
Figure S18. Meta-analysis results for change in body mass index (kg/m^2) of metformin vs placebo/lifestyle intervention 44
Figure S19. Meta-analysis results for change in body mass index (kg/m^2) of sulfonylureas vs placebo/lifestyle intervention 45
Figure S20. Meta-analysis results for change in body mass index (kg/m^2) of thiazolidinediones vs placebo/lifestyle intervention 46
Figure S21. Meta-analysis results for change in body mass index (kg/m^2) of glinides vs placebo/lifestyle intervention 47
Figure S22. Meta-analysis results for change in body mass index (kg/m^2) of α-glucosidase inhibitors vs placebo/lifestyle intervention 48
Figure S23. Meta-analysis results for change in body mass index (kg/m^2) of dipeptidyl peptidase-4 inhibitors vs placebo/lifestyle intervention 49
Figure S24. Meta-analysis results for change in body mass index (kg/m^2) of sodium-glucose cotransporter-2 inhibitors vs placebo/lifestyle intervention 50
Figure S25. Meta-analysis results for change in body mass index (kg/m^2) of glucagon-like peptide-1 receptor agonists vs placebo/lifestyle intervention 51
Figure S26. Meta-analysis results for change in total cholesterol (mmol/l) of metformin vs placebo/lifestyle intervention 52
Figure S27. Meta-analysis results for change in total cholesterol (mmol/l) of sulfonylureas vs placebo/lifestyle intervention 53
Figure S28. Meta-analysis results for change in total cholesterol (mmol/l) of thiazolidinediones vs placebo/lifestyle intervention 54
Figure S29. Meta-analysis results for change in total cholesterol (mmol/l) of glinides vs placebo/lifestyle intervention 55
Figure S30. Meta-analysis results for change in total cholesterol (mmol/l) of α-glucosidase inhibitors vs placebo/lifestyle intervention 56
Figure S31. Meta-analysis results for change in total cholesterol (mmol/l) of dipeptidyl peptidase-4 inhibitors vs placebo/lifestyle intervention 57

Figure S32. Meta-analysis results for change in total cholesterol (mmol/l) of sodium-glucose cotransporter-2 inhibitors vs placebo/lifestyle intervention 58
Figure S33. Meta-analysis results for change in total cholesterol (mmol/l) of glucagon-like peptide-1 receptor agonists vs placebo/lifestyle intervention 59
Figure S34. Meta-analysis results for change in high density lipoprotein-cholesterol (mmol/l) of metformin vs placebo/lifestyle intervention 60
Figure S35. Meta-analysis results for change in high density lipoprotein-cholesterol (mmol/l) of sulfonylureas vs placebo/lifestyle intervention 61
Figure S36. Meta-analysis results for change in high density lipoprotein-cholesterol (mmol/l) of thiazolidinediones vs placebo/lifestyle intervention 62
Figure S37. Meta-analysis results for change in high density lipoprotein-cholesterol (mmol/l) of glinides vs placebo/lifestyle intervention 63
Figure S38. Meta-analysis results for change in high density lipoprotein-cholesterol (mmol/l) of α-glucosidase inhibitors vs placebo/lifestyle intervention 64
Figure S39. Meta-analysis results for change in high density lipoprotein-cholesterol (mmol/l) of dipeptidyl peptidase-4 inhibitors vs placebo/lifestyle intervention 65
Figure S40. Meta-analysis results for change in high density lipoprotein-cholesterol (mmol/l) of sodium-glucose cotransporter-2 inhibitors vs placebo/lifestyle intervention 66
Figure S41. Meta-analysis results for change in high density lipoprotein-cholesterol (mmol/l) of glucagon-like peptide-1 receptor agonists vs placebo/lifestyle intervention 67
Figure S42. Meta-analysis results for change in systolic blood pressure (mmHg) of metformin vs placebo/lifestyle intervention 68
Figure S43. Meta-analysis results for change in systolic blood pressure (mmHg) of sulfonylureas vs placebo/lifestyle intervention 69
Figure S44. Meta-analysis results for change in systolic blood pressure (mmHg) of thiazolidinediones vs placebo/lifestyle intervention 70
Figure S45. Meta-analysis results for change in systolic blood pressure (mmHg) of glinides vs placebo/lifestyle intervention 71
Figure S46. Meta-analysis results for change in systolic blood pressure (mmHg) of α-glucosidase inhibitors vs placebo/lifestyle intervention 72
Figure S47. Meta-analysis results for change in systolic blood pressure (mmHg) of dipeptidyl peptidase-4 inhibitors vs placebo/lifestyle intervention 73
Figure S48. Meta-analysis results for change in systolic blood pressure (mmHg) of sodium-glucose cotransporter-2 inhibitors vs placebo/lifestyle intervention 74
Figure S49. Meta-analysis results for change in systolic blood pressure (mmHg) of glucagon-like peptide-1 receptor agonists vs placebo/lifestyle intervention 75
Figure S50. Meta-analysis results for incidence of hypoglycemia of metformin vs placebo/lifestyle intervention 76
Figure S51. Meta-analysis results for incidence of hypoglycemia of sulfonylureas vs placebo/lifestyle intervention 77
Figure S52. Meta-analysis results for incidence of hypoglycemia of thiazolidinediones vs placebo/lifestyle intervention 78
Figure S53. Meta-analysis results for incidence of hypoglycemia of glinides vs placebo/lifestyle intervention 79
Figure S54. Meta-analysis results for incidence of hypoglycemia of α-glucosidase inhibitors vs placebo/lifestyle intervention 80
Figure S55. Meta-analysis results for incidence of hypoglycemia of dipeptidyl peptidase-4 inhibitors vs placebo/lifestyle intervention 82
Figure S56. Meta-analysis results for incidence of hypoglycemia of sodium-glucose cotransporter-2 inhibitors vs placebo/lifestyle intervention 83
Figure S57. Meta-analysis results for incidence of hypoglycemia of glucagon-like peptide-1 receptor agonists vs placebo/lifestyle intervention 84
Figure S58. Meta-analysis results for incidence of death of metformin vs placebo/lifestyle intervention 85
Figure S59. Meta-analysis results for incidence of death of sulfonylureas vs placebo/lifestyle intervention 86
Figure S60. Meta-analysis results for incidence of death of thiazolidinediones vs placebo/lifestyle intervention 87
Figure S61. Meta-analysis results for incidence of death of glinides vs placebo/lifestyle intervention 88
Figure S62. Meta-analysis results for incidence of death of α-glucosidase inhibitors vs placebo/lifestyle intervention 89
Figure S63. Meta-analysis results for incidence of death of dipeptidyl peptidase-4 inhibitors vs placebo/lifestyle intervention 90
Figure S64. Meta-analysis results for incidence of death of sodium-glucose cotransporter-2 inhibitors vs placebo/lifestyle intervention 91
Figure S65. Meta-analysis results for incidence of death of glucagon-like peptide-1 receptor agonists vs placebo/lifestyle intervention 92
Figure S66. Meta-analysis results for incidence of total vascular events of metformin vs placebo/lifestyle intervention.. 93
Figure S67. Meta-analysis results for incidence of total vascular events of sulfonylureas vs placebo/lifestyle intervention 94
Figure S68. Meta-analysis results for incidence of total vascular events of thiazolidinediones vs placebo/lifestyle intervention 95

Figure S69. Meta-analysis results for incidence of total vascular events of α-glucosidase inhibitors vs placebo/lifestyle intervention 96
Figure S70. Meta-analysis results for incidence of total vascular events of dipeptidyl peptidase-4 inhibitors vs placebo/lifestyle intervention 97
Figure S71. Meta-analysis results for incidence of total vascular events of sodium-glucose cotransporter-2 inhibitors vs placebo/lifestyle intervention 98
Figure S72. Meta-analysis results for incidence of total vascular events of glucagon-like peptide-1 receptor agonists vs placebo/lifestyle intervention 99
Figure S73. Meta-analysis results for incidence of myocardial infarction of metformin vs placebo/lifestyle intervention 100
Figure S74. Meta-analysis results for incidence of myocardial infarction of sulfonylureas vs placebo/lifestyle intervention 101
Figure S75. Meta-analysis results for incidence of myocardial infarction of thiazolidinediones vs placebo/lifestyle intervention 102
Figure S76. Meta-analysis results for incidence of myocardial infarction of α-glucosidase inhibitors vs placebo/lifestyle intervention 103
Figure S77. Meta-analysis results for incidence of myocardial infarction of dipeptidyl peptidase-4 inhibitors vs placebo/lifestyle intervention 104
Figure S78. Meta-analysis results for incidence of myocardial infarction of sodium-glucose cotransporter-2 inhibitors vs placebo/lifestyle intervention 105
Figure S79. Meta-analysis results for incidence of myocardial infarction of glucagon-like peptide-1 receptor agonists vs placebo/lifestyle intervention 106
Figure S80. Meta-analysis results for incidence of heart failure of metformin vs placebo/lifestyle intervention 107
Figure S81. Meta-analysis results for incidence of heart failure of thiazolidinediones vs placebo/lifestyle intervention 108
Figure S82. Meta-analysis results for incidence of heart failure of dipeptidyl peptidase-4 inhibitors vs placebo/lifestyle intervention 109
Figure S83. Meta-analysis results for incidence of heart failure of sodium-glucose cotransporter-2 inhibitors vs placebo/lifestyle intervention 110
Figure S84. Meta-analysis results for incidence of heart failure of glucagon-like peptide-1 receptor agonists vs placebo/lifestyle intervention 111
Figure S85. Meta-analysis results for incidence of stroke of metformin vs placebo/lifestyle intervention 112
Figure S86. Meta-analysis results for incidence of stroke of sulfonylureas vs placebo/lifestyle intervention 113
Figure S87. Meta-analysis results for incidence of stroke of thiazolidinediones vs placebo/lifestyle intervention 114
Figure S88. Meta-analysis results for incidence of stroke of dipeptidyl peptidase-4 inhibitors vs placebo/lifestyle intervention 115
Figure S89. Meta-analysis results for incidence of stroke of sodium-glucose cotransporter-2 inhibitors vs placebo/lifestyle intervention 116
Figure S90. Meta-analysis results for incidence of stroke of glucagon-like peptide-1 receptor agonists vs placebo/lifestyle intervention 117
Figure S91. Meta-analysis results for incidence of diabetic nephropathy of metformin vs placebo/lifestyle intervention 118
Figure S92. Meta-analysis results for incidence of diabetic nephropathy of thiazolidinediones vs placebo/lifestyle intervention 119
Figure S93. Meta-analysis results for incidence of diabetic nephropathy of dipeptidyl peptidase-4 inhibitors vs placebo/lifestyle intervention 120
Figure S94. Meta-analysis results for incidence of diabetic nephropathy of sodium-glucose cotransporter-2 inhibitors vs placebo/lifestyle intervention 121
Figure S95. Meta-analysis results for incidence of diabetic nephropathy of glucagon-like peptide-1 receptor agonists vs placebo/lifestyle intervention 122
Figure S96. Meta-analysis results for incidence of AE-induced discontinuations of metformin vs placebo/lifestyle intervention 123
Figure S97. Meta-analysis results for incidence of AE-induced discontinuations of sulfonylureas vs placebo/lifestyle intervention 124
Figure S98. Meta-analysis results for incidence of AE-induced discontinuations of thiazolidinediones vs placebo/lifestyle intervention 125
Figure S99. Meta-analysis results for incidence of AE-induced discontinuations of glinides vs placebo/lifestyle intervention 126
Figure S100. Meta-analysis results for incidence of AE-induced discontinuations of α-glucosidase inhibitors vs placebo/lifestyle intervention 127
Figure S101. Meta-analysis results for incidence of AE-induced discontinuations of dipeptidyl peptidase-4 inhibitors vs placebo/lifestyle intervention 129
Figure S102. Meta-analysis results for incidence of AE-induced discontinuations of sodium-glucose cotransporter-2 inhibitors vs placebo/lifestyle intervention 130
Figure S103. Meta-analysis results for incidence of AE-induced discontinuations of glucagon-like peptide-1 receptor agonists vs placebo/lifestyle intervention 131

Figure S104. Sensitivity analysis results for change in hemoglobin Alc (%) of glucose-lowering drugs vs placebo/lifestyle intervention 133
Figure S105. Sensitivity analysis results for change in fasting plasma glucose (mmol/l) of glucose-lowering drugs vs placebo/lifestyle intervention 135
Figure S106. Sensitivity analysis results for change in body mass index (kg/m^2) of glucose-lowering drugs vs placebo/lifestyle intervention 136
Figure S107. Sensitivity analysis results for change in total cholesterol (mmol/l) of glucose-lowering drugs vs placebo/lifestyle intervention 138
Figure S108. Sensitivity analysis results for change in high density lipoprotein-cholesterol (mmol/l) of glucose-lowering drugs vs placebo/lifestyle intervention 140
Figure S109. Sensitivity analysis results for change in systolic blood pressure (mmHg) of glucose-lowering drugs vs placebo/lifestyle intervention 141
Figure S110. Sensitivity analysis results for incidence of hypoglycemia of glucose-lowering drugs vs placebo/lifestyle intervention 143
Reference 144

Table S1. Eligibility criteria

**Inclusion criteria**	
1. Participants were patients with type 2 diabetes (≥18 years).	
2. Intervention was a glucose-lowering drug in the regimen of monotherapy, including 27 glucose-lowering drugs from nine drug classes:	
Biguanide:	metformin (500~2550 mg/d)
Sulfonylureas:	glyburide (1.25~20 mg/d), glimepiride (1~8 mg/d), gliclazide (80~320 mg/d), glipizide (2.5~40 mg/d), gliquidone (30~180 mg/d)
Thiazolidinediones:	rosiglitazone (4~8 mg/d), pioglitazone (15~45 mg/d)
Glinides:	repaglinide (1~16 mg/d), nateglinide (120~360 mg/d), mitiglinide (30~60 mg/d)
α-Glucosidase inhibitors:	acarbose (100~300 mg/d), voglibose (0.2~1.2 mg/d), miglitol (100~300 mg/d)
DPP-4 inhibitors:	sitagliptin (50,100 mg/d), saxagliptin (2.5,5 mg/d), vildagliptin (50,100 mg/d), linagliptin (5 mg/d), alogliptin (12.5,25 mg/d)
SGLT2 inhibitors:	dapagliflozin (5,10 mg/d), empagliflozin (10~25 mg/d), canagliflozin (100~300 mg/d)
GLP-1 receptor agonists:	exenatide (0.01~0.02 mg/d twice-daily; 2 mg once-weekly), liraglutide (0.6~1.8 mg/d), lixisenatide (0.01~0.02 mg/d), beinaglutide (0.3~0.6 mg/d)
Insulins:	insulin and insulin analogs
3. Comparator was placebo or lifestyle intervention like diet and/or physical exercise therapy.	
4. Background therapy was limited to lifestyle intervention.	
5. Study duration was ≥12 weeks.	
6. Outcomes were hemoglobin Alc (HbA1c), fasting plasma glucose (FPG), body mass index (BMI), total cholesterol (TC), high density lipoprotein-cholesterol (HDL-C), systolic blood pressure (SBP), hypoglycemia, mortality, macrovascular and microvascular outcomes, or discontinuation. The change-from-baseline value of continuous outcomes with related standard deviation or standard error or 95% confidence interval (or the mean values of the outcomes in the baseline and in the endpoint with related standard deviation or standard error or 95% confidence interval) should be reported.	
7. Study design was randomized controlled trial.	
8. Study was published in English or Chinese.	
**Exclusion criteria**	
1. Participants were not patients with type 2 diabetes, such as those with impaired glucose tolerance, impaired fasting glucose, type 1 diabetes, gestational diabetes, or latent autoimmune diabetes in adults.	
2. Participants were <18 years.	
3. Participants were comorbid with pulmonary tuberculosis, tumor, cancer, diabetic ketoacidosis, or mental diseases; or participants with severe heart, liver or kidney dysfunction; or participants during pregnancy or lactation; or participants in the perioperative period.	
4. Intervention was not monotherapy of a targeted glucose-lowering drug; or in addition to the targeted drug, there were concomitant medications that affect the outcomes, such as other glucose-lowering drugs, antihypertensive drugs and antihyperlipidemic drugs; or the dose of the drug was not targeted or not reported.	
5. Comparator was not placebo or lifestyle intervention; or in addition to placebo or lifestyle intervention, there were concomitant medications that affect the outcomes, such as other glucose-lowering drugs, antihypertensive drugs and antihyperlipidemic drugs.	
6. Study duration was <12 weeks or not reported.	
7. There was no required clinical outcome, or has required outcome but no required value.	
8. Study was not randomized controlled trial, such as observational study, economic evaluation, review, meeting abstract, commentary or letter;	
9. Duplicate.	

Table S2. Search terms and search strategies

**Database 1: PubMed**		
Advanced Search	Query	Results
Search strategy	(Type 2 diabetes OR Non insulin dependent diabetes mellitus) AND (Metformin OR Sulfonylurea OR Glyburide OR Glibenclamide OR Glimepiride OR Gliclazide OR Glipizide OR Gliquidone OR Thiazolidinedione OR Rosiglitazone OR Pioglitazone OR Glinide OR Repaglinide OR Nateglinide OR Mitiglinide OR α-Glucosidase inhibitor OR Acarbose OR Voglibose OR Miglitol OR Dipeptidyl peptidase 4 inhibitor OR Sitagliptin OR Saxagliptin OR Vildagliptin OR Linagliptin OR Alogliptin OR Sodium-glucose cotransporter 2 inhibitor OR Dapagliflozin OR Empagliflozin OR Canagliflozin OR Glucagon-like peptide-1 receptor agonist OR Exenatide OR Liraglutide OR Lixisenatide OR Beinaglutide OR Insulin) AND (Placebo OR Diet OR Dietary OR Exercise OR Lifestyle) AND ("1990/01/01"[Date - Publication] : "2020/12/31"[Date - Publication]) Filters: Clinical Study, Clinical Trial, Humans, Chinese, English, Adult: 19+ years	4940
**Database 2: Embase**		
Advanced Search	Query	Results
Search strategy	('type 2 diabetes' OR 'non insulin dependent diabetes mellitus') AND (metformin OR sulfonylurea OR glyburide OR glibenclamide OR glimepiride OR gliclazide OR glipizide OR gliquidone OR thiazolidinedione OR rosiglitazone OR pioglitazone OR glinide OR repaglinide OR nateglinide OR mitiglinide OR 'α-glucosidase inhibitor' OR acarbose OR voglibose OR miglitol OR 'dipeptidyl peptidase 4 inhibitor' OR sitagliptin OR saxagliptin OR vildagliptin OR linagliptin OR alogliptin OR 'sodium-glucose cotransporter 2 inhibitor' OR dapagliflozin OR empagliflozin OR canagliflozin OR 'glucagon-like peptide-1 receptor agonist' OR exenatide OR liraglutide OR lixisenatide OR beinaglutide OR insulin) AND (placebo OR diet OR dietary OR exercise OR lifestyle) AND [randomized controlled trial]/lim AND [article]/lim AND ([chinese]/lim OR [english]/lim) AND ([adult]/lim OR [aged]/lim) AND [humans]/lim AND [clinical study]/lim AND [embase]/lim AND [1990-2020]/py	6135
**Database 3: Web of Science**		
Advanced Search	Query	Results
Search strategy	TS=('Type 2 diabetes' OR 'Non insulin dependent diabetes mellitus') AND TS=(Metformin OR Sulfonylurea OR Glyburide OR Glibenclamide OR Glimepiride OR Gliclazide OR Glipizide OR Gliquidone OR Thiazolidinedione OR Rosiglitazone OR Pioglitazone OR Glinide OR Repaglinide OR Nateglinide OR Mitiglinide OR 'α-Glucosidase inhibitor' OR Acarbose OR Voglibose OR Miglitol OR 'Dipeptidyl peptidase 4 inhibitor' OR Sitagliptin OR Saxagliptin OR Vildagliptin OR Linagliptin OR Alogliptin OR 'Sodium-glucose cotransporter 2 inhibitor' OR Dapagliflozin OR Empagliflozin OR Canagliflozin OR 'Glucagon-like peptide-1 receptor agonist' OR Exenatide OR Liraglutide OR Lixisenatide OR Beinaglutide OR Insulin) AND TS=(Placebo OR Diet OR Dietary OR Exercise OR Lifestyle) AND TS=('Clinical Study' OR 'Clinical Trial')	3729
Publication date	1990 to 2020	
**Database 4: China National Knowledge Infrastructure (CNKI)**		
Expert Search	Query	Results

Search strategy	(SU=2 型糖尿病 OR SU=II型糖尿病 OR SU=非胰岛素依赖型糖尿病) AND (SU=二甲双胍 OR SU=磺脲类药物 OR SU=格列本脲 OR SU=格列美脲 OR SU=格列齐特 OR SU=格列吡嗪 OR SU=格列喹酮 OR SU=噻唑烷二酮类药物 OR SU=罗格列酮 OR SU=吡格列酮 OR SU=格列奈类药物 OR SU=瑞格列奈 OR SU=那格列奈 OR SU=米格列奈 OR SU=α-糖苷酶抑制剂 OR SU=阿卡波糖 OR SU=伏格列波糖 OR SU=米格列醇 OR SU=DPP-4 抑制剂 OR SU=西格列汀 OR SU=沙格列汀 OR SU=维格列汀 OR SU=利格列汀 OR SU=阿格列汀 OR SU=SGLT2 抑制剂 OR SU=达格列净 OR SU=恩格列净 OR SU=卡格列净 OR SU=GLP-1 受体激动剂 OR SU=艾塞那肽 OR SU=利拉鲁肽 OR SU=利司那肽 OR SU=贝那鲁肽 OR SU=胰岛素) AND (SU=安慰剂 OR SU=饮食 OR SU=运动 OR SU=生活方式干预 OR SU=生活方式 OR SU=生活干预)	3582
Publication date	1990-01-01 to 2020-12-31	
Article type	Journal; Educational Journal; Featured journal; Doctoral dissertation; Master's dissertation	
**Database 5: WanFang Data**		
Expert Search	Query	Results
Search strategy	(主题:(2 型糖尿病) or 主题:(II型糖尿病) or 主题:(非胰岛素依赖型糖尿病)) and (主题:(二甲双胍) or 主题:(磺脲类药物) or 主题:(格列本脲) or 主题:(格列美脲) or 主题:(格列齐特) or 主题:(格列吡嗪) or 主题:(格列喹酮) or 主题:(噻唑烷二酮类药物) or 主题:(罗格列酮) or 主题:(吡格列酮) or 主题:(格列奈类药物) or 主题:(瑞格列奈) or 主题:(那格列奈) or 主题:(米格列奈) or 主题:(α-糖苷酶抑制剂) or 主题:(阿卡波糖) or 主题:(伏格列波糖) or 主题:(米格列醇) or 主题:(DPP-4 抑制剂) or 主题:(西格列汀) or 主题:(沙格列汀) or 主题:(维格列汀) or 主题:(利格列汀) or 主题:(阿格列汀) or 主题:(SGLT2 抑制剂) or 主题:(达格列净) or 主题:(恩格列净) or 主题:(卡格列净) or 主题:(GLP-1 受体激动剂) or 主题:(艾塞那肽) or 主题:(利拉鲁肽) or 主题:(利司那肽) or 主题:(贝那鲁肽) or 主题:(胰岛素)) and (主题:(安慰剂) or 主题:(饮食) or 主题:(运动) or 主题:(生活方式干预) or 主题:(生活方式) or 主题:(生活干预))	10960
Publication date	1990 to 2020	
Article type	Journal; Dissertation	
**Database 6: The Chongqing VIP (CQVIP)**		
Expert Search	Query	Results
Search strategy	(M=2 型糖尿病 OR M=II型糖尿病 OR M=非胰岛素依赖型糖尿病) AND (M=二甲双胍 OR M=磺脲类药物 OR M=格列本脲 OR M=格列美脲 OR M=格列齐特 OR M=格列吡嗪 OR M=格列喹酮 OR M=噻唑烷二酮类药物 OR M=罗格列酮 OR M=吡格列酮 OR M=格列奈类药物 OR M=瑞格列奈 OR M=那格列奈 OR M=米格列奈 OR M=α-糖苷酶抑制剂 OR M=阿卡波糖 OR M=伏格列波糖 OR M=米格列醇 OR M=DPP-4 抑制剂 OR M=西格列汀 OR M=沙格列汀 OR M=维格列汀 OR M=利格列汀 OR M=阿格列汀 OR M=SGLT2 抑制剂 OR M=达格列净 OR M=恩格列净 OR M=卡格列净 OR M=GLP-1 受体激动剂 OR M=艾塞那肽 OR M=利拉鲁肽 OR M=利司那肽 OR M=贝那鲁肽 OR M=胰岛素) AND (M=安慰剂 OR M=饮食 OR M=运动 OR M=生活方式干预 OR M=生活方式 OR M=生活干预)	776
Publication date	1990 to 2020	

Table S3. The characteristics and risk of bias of the included studies

Study	Characteristics of study																Risk of bias assessments						
	Location	Background therapy	Study duration, w	Treatment		Sample size, n		Female, n		Age, y		Diabetes duration, y		Baseline HbA1c, %		Drug dose, mg/d	Random sequence generation	Allocation concealment	Blinding: participants & personnel	Blinding: outcome assessment	Incomplete outcome data	Selective reporting	Other bias
				I	C	I	C	I	C	I	C	I	C	I	C								
Ji L 2016a[1]	China	Diet, exercise	24	Metformin	Placebo	126	127	57	40	52.6(9.5)	53.6(9.7)	1(0.2)	1.1(0.2)	8.7(1)	9(1.1)	1000	Low risk	Unclear risk	Low risk	Low risk	Low risk	Low risk	Unclear risk
Ji L 2016b[1]	China	Diet, exercise	24	Metformin	Placebo	124	127	49	40	53(10.3)	53.6(9.7)	1.1(0.2)	1.1(0.2)	8.7(1.1)	9(1.1)	1700	Low risk	Unclear risk	Low risk	Low risk	Low risk	Low risk	Unclear risk
Hassan MH 2015a[2]	Egypt	Diet, exercise	12	Metformin	Placebo	30	30	NR	NR	30~75	30~75	NR	NR	8.9(2.19)	9(2.74)	1000	Unclear risk	Unclear risk	Low risk	Low risk	Low risk	Unclear risk	Unclear risk
Pratley RE 2014a[3]	USA, Czech Republic, Hungary, Israel, Lithuania, Mexico, Poland, Romania, Russia, Slovakia, South Africa, Ukraine	Diet, exercise	26	Metformin	Placebo	114	109	67	54	54.6(10.2)	53.1(9.6)	3.8(3.9)	4.3(4.78)	NR	NR	1000	Unclear risk	Unclear risk	Low risk	Low risk	High risk	Low risk	Unclear risk
Pratley RE 2014b[3]	USA, Czech Republic, Hungary, Israel, Lithuania, Mexico, Poland, Romania, Russia, Slovakia, South Africa, Ukraine	Diet, exercise	26	Metformin	Placebo	111	109	60	54	52.6(11.3)	53.1(9.6)	4.1(4.59)	4.3(4.78)	NR	NR	2000	Unclear risk	Unclear risk	Low risk	Low risk	High risk	Low risk	Unclear risk
Ferrannini E 2013a[4]	Argentina, Croatia, Estonia, Germany, Italy, Korea, Lithuania, Romania, Russia, Sweden, Slovakia, Taiwan, Ukraine	None	12	Metformin	Placebo	80	82	41	37	58	58	NR	NR	8.1(0.9)	7.8(0.8)	1000~2000	Low risk	Low risk	High risk	Low risk	Low risk	Low risk	Unclear risk
Haak T 2012a[5]	Germany, UK	None	24	Metformin	Placebo	144	72	62	36	52.9(10.4)	55.7(11)	NR	NR	8.7(0.9)	8.7(1)	1000	Unclear risk	Unclear risk	Low risk	Low risk	Low risk	Low risk	Unclear risk
Haak T 2012b[5]	Germany, UK	None	24	Metformin	Placebo	147	72	69	36	55.2(10.6)	55.7(11)	NR	NR	8.5(0.9)	8.7(1)	2000	Unclear risk	Unclear risk	Low risk	Low risk	Low risk	Low risk	Unclear risk
Chakraborty A 2011[6]	India	Diet, exercise	24	Metformin	Placebo	110	98	49	53	30~55	30~55	1~5	1~5	8.7(1.4)	8.7(1.5)	850~2000	Unclear risk	Unclear risk	Low risk	Low risk	High risk	Low risk	Unclear risk
List JF 2009a[7]	USA, Canada, Mexico	Diet, exercise	12	Metformin	Placebo	51	44	27	19	54(9)	53(11)	NR	NR	7.6(0.8)	7.9(0.9)	Initial:750; Max:1500	Unclear risk	Unclear risk	Low risk	Low risk	High risk	Low risk	Unclear risk
Goldstein BJ 2007a[8]	Australia, Chile, Colombia, Costa Rica, Guatemala, Hungary, Lithuania, Malaysia, Mexico, New Zealand, Norway, Peru, Philippines, Russia, South Africa, UK, USA	Diet, exercise	24	Metformin	Placebo	178	165	NR	NR	18~78	18~78	4.5	4.5	8.9(1)	8.68(1)	1000	Unclear risk	Unclear risk	Low risk	Low risk	Low risk	Low risk	Unclear risk
Goldstein BJ 2007b[8]	Australia, Chile, Colombia, Costa Rica, Guatemala, Hungary, Lithuania, Malaysia, Mexico, New Zealand, Norway, Peru, Philippines, Russia, South Africa, UK, USA	Diet, exercise	24	Metformin	Placebo	177	165	NR	NR	18~78	18~78	4.5	4.5	8.68(0.91)	8.68(1)	2000	Unclear risk	Unclear risk	Low risk	Low risk	Low risk	Low risk	Unclear risk
Wang L 2006[9]	China	Diet, exercise	24	Metformin	Placebo	26	16	10	8	47.8(1.8)	46.6(2.4)	NR	NR	NR	NR	1500	Unclear risk	Unclear risk	Low risk	Low risk	Low risk	Unclear risk	Unclear risk
Fujioka K 2005a[10]	USA, Israel, Belgium	Diet, exercise	12	Metformin	Placebo	161	79	60	37	55(11)	58(11)	3.3(2.8)	3.2(2.6)	8.1(0.9)	7.9(0.9)	1000	Low risk	Unclear risk	Low risk	Low risk	High risk	Unclear risk	Unclear risk

Fujioka K 2005b[10]	USA, Israel, Belgium	Diet, exercise	16	Metformin	Placebo	128	117	72	60	55(11)	54(10)	3.3(2.9)	2.7(2.7)	8.2(0.9)	8.3(1.1)	500	Low risk	Unclear risk	Low risk	Low risk	High risk	Unclear risk	Unclear risk
Fujioka K 2005c[10]	USA, Israel, Belgium	Diet, exercise	16	Metformin	Placebo	120	117	50	60	56(10)	54(10)	3(2.7)	2.7(2.7)	8.4(1.1)	8.3(1.1)	1000	Low risk	Unclear risk	Low risk	Low risk	High risk	Unclear risk	Unclear risk
Fujioka K 2005d[10]	USA, Israel, Belgium	Diet, exercise	16	Metformin	Placebo	120	117	62	60	56(11)	54(10)	2.9(2.7)	2.7(2.7)	8.4(1.0)	8.3(1.1)	1500	Low risk	Unclear risk	Low risk	Low risk	High risk	Unclear risk	Unclear risk
Fujioka K 2005e[10]	USA, Israel, Belgium	Diet, exercise	16	Metformin	Placebo	134	117	79	60	55(11)	54(10)	2.7(2.5)	2.7(2.7)	8.4(1.1)	8.3(1.1)	2000	Low risk	Unclear risk	Low risk	Low risk	High risk	Unclear risk	Unclear risk
Karlsson HK 2005a[11]	Finland	Diet, exercise	26	Metformin	Placebo	9	11	3	2	57.7(8.7)	58.6(8.29)	Newly diagnosed	Newly diagnosed	7.1(0.9)	6.3(0.33)	Initial:1000; 3w:2000	Unclear risk	Unclear risk	Low risk	Low risk	Low risk	Low risk	Unclear risk
Viljanen AP 2005a[12]	Finland	Diet	26	Metformin	Placebo	12	11	5	2	57.8(8.7)	58.7(8.3)	NR	NR	6.9(0.9)	6.2(0.7)	Initial:1000; 3w:2000	Unclear risk	Unclear risk	Low risk	Low risk	Low risk	Unclear risk	Unclear risk
Horton ES 2004a[13]	USA	Diet, exercise	24	Metformin	Placebo	104	104	34	37	55.4(11.22)	59(11.22)	3.7(4.08)	4.2(4.08)	8.3(0.99)	8.2(0.99)	1500	Unclear risk	Unclear risk	Low risk	Low risk	Low risk	Unclear risk	Unclear risk
Manzella D 2004[14]	Italy	Diet	17	Metformin	Placebo	60	60	29	27	57(11)	57(11)	NR	NR	8(0.2)	8.1(0.2)	1700	Unclear risk	Unclear risk	Low risk	Low risk	Low risk	Unclear risk	Unclear risk
Del Prato S 2003[15]	France, Italy, Netherlands	Diet	29	Metformin	Placebo	284	144	116	53	56(9)	56(9)	NR	NR	7.79(1.61)	7.43(1.48)	Initial:850; Max:2550	Unclear risk	Unclear risk	Low risk	Low risk	High risk	Low risk	Unclear risk
Chiasson JL 2001a[16]	Canada, France	Diet, exercise	36	Metformin	Placebo	83	83	22	27	57.9(8.6)	57.7(9.9)	7.5(7.4)	5.1(4.9)	8.2(0.9)	8.1(0.7)	1500	Unclear risk	Unclear risk	Low risk	Low risk	Low risk	Unclear risk	Unclear risk
Mather KJ 2001[17]	USA, Canada	Diet	12	Metformin	Placebo	28	15	14	4	50.7(9.52)	54.8(10.07)	NR	NR	6.8(1.06)	7.2(1.94)	1000	High risk	Unclear risk	Low risk	Low risk	Low risk	Low risk	Unclear risk
Wu GT 2001[18]	China	Diet, exercise	24	Metformin	Placebo	60	60	25	23	49.8(6.7)	50.4(6.4)	4.2(2.1)	4.3(2.4)	9.75(0.37)	9.56(0.32)	1000	Unclear risk	Low risk	Low risk	Low risk	Low risk	Unclear risk	Unclear risk
Lee A 1998[19]	USA	Diet	24	Metformin	Placebo	24	24	NR	NR	59(14.7)	61(9.8)	4(4.90)	3(9.80)	8.4(1.47)	8.4(0.98)	850 or 1700	Low risk	Unclear risk	Low risk	Low risk	High risk	Unclear risk	Unclear risk
Hoffmann J 1997a[20]	Germany	Diet	24	Metformin	Placebo	31	32	17	20	55.9(7.8)	60.2(8.6)	2.08(1.45)	3.6(2.83)	9.7(0.9)	9.4(0.9)	1700	Low risk	Unclear risk	Low risk	Low risk	Low risk	Unclear risk	Unclear risk
Wang DL 2020a[21]	China	Diet, exercise	52	Metformin	Diet, exercise	60	60	22	24	46.7(4.6)	46.8(4.7)	NR	NR	8.64(1.06)	8.79(1.21)	1500	Low risk	Unclear risk	High risk	Low risk	Low risk	Unclear risk	Unclear risk
Yang Y 2019[22]	China	Diet, exercise	12	Metformin	Diet, exercise	37	33	15	13	70.5(4.2)	70.2(4.1)	8.2(2.6)	8.5(2.7)	NR	NR	Initial:1000; Max:2500	Unclear risk	Unclear risk	High risk	Low risk	Low risk	Unclear risk	Unclear risk
Han SQ 2018[23]	China	Diet, exercise	12	Metformin	Diet, exercise	35	35	15	11	59.68(12.35)	60.12(13.62)	7.15(2.35)	7.54(2.21)	9.12(2.23)	9.08(2.31)	1500	Low risk	Unclear risk	High risk	Low risk	Low risk	Low risk	Unclear risk
Zhou RF 2017a[24]	China	None	12	Metformin	Diet, exercise	30	30	17	15	49.1(12)	47.7(13.1)	Newly diagnosed	Newly diagnosed	7.6(0.6)	7.6(0.6)	1000	Unclear risk	Unclear risk	High risk	Low risk	Low risk	Low risk	Unclear risk
Bai N 2016[25]	China	Diet, exercise	12	Metformin	Diet, exercise	45	45	17	19	54(8.28)	54(8.12)	NR	NR	8.9(2.4)	8.8(2.2)	1500	Unclear risk	Unclear risk	High risk	Low risk	Low risk	Low risk	Unclear risk
Geng N 2016[26]	China	Diet, exercise	12	Metformin	Diet, exercise	30	30	0	0	40.9(5.4)	42.6(6.2)	NR	NR	8.1(1.2)	7.9(1.2)	Initial:500; Max:1500	Unclear risk	Unclear risk	High risk	Low risk	Low risk	Low risk	Unclear risk

Guo DL 2014[27]	China	Diet, exercise	26	Metformin	Diet, exercise	60	60	20	28	44.9	44.9	NR	NR	9.51(1.23)	9.49(1.18)	Initial:500; Max:2000	Unclear risk	Unclear risk	High risk	Low risk	Low risk	Unclear risk	Unclear risk
Guo W 2014[28]	China	Diet, exercise	12	Metformin	Diet, exercise	30	30	13	12	49.7(6.8)	50.4(6.4)	Newly diagnosed	Newly diagnosed	7.71(1.52)	7.12(1.24)	1500	Unclear risk	Unclear risk	High risk	Low risk	Low risk	Unclear risk	Unclear risk
Li L 2014a[29]	China	Diet, exercise	12	Metformin	Diet, exercise	16	12	6	4	49.5(11.7)	49.5(11.7)	1.2(0.9)	1.2(0.9)	8.09(1.23)	8.01(1.07)	1500	Unclear risk	Unclear risk	High risk	Low risk	Low risk	Unclear risk	Unclear risk
Esteghamati A 2013[30]	Iran	Diet, exercise	12	Metformin	Diet, exercise	50	49	31	30	49.74(8.23)	52.38(8.38)	Newly diagnosed	Newly diagnosed	7.55(1.55)	7.09(0.75)	1000	Unclear risk	Unclear risk	High risk	Low risk	Low risk	Low risk	Unclear risk
Liu YT 2013[31]	China	None	14	Metformin	Diet, exercise	38	35	21	13	53.12(8.29)	58.64(7.71)	NR	NR	7.86(1.58)	7.07(0.92)	Initial:500; Max:1500	Low risk	Unclear risk	High risk	Low risk	Low risk	Low risk	Unclear risk
Xi Y 2013a[32]	China	Diet, exercise	16	Metformin	Diet, exercise	20	20	10	9	51(4)	51(7)	Newly diagnosed	Newly diagnosed	8.6(0.9)	8.3(0.9)	2000	Low risk	Unclear risk	High risk	Low risk	Low risk	Unclear risk	Unclear risk
Yang WC 2013[33]	China	Exercise	12	Metformin	Exercise	19	19	9	11	NR	NR	2(1.2)	2(1.2)	8.42(0.71)	8.33(0.59)	1000	High risk	Unclear risk	High risk	Low risk	Unclear risk	Unclear risk	Unclear risk
Qu JC 2011a[34]	China	Diet, exercise	12	Metformin	Diet, exercise	30	30	NR	NR	45(9)	45(9)	Newly diagnosed	Newly diagnosed	NR	NR	1000	High risk	Unclear risk	High risk	Low risk	Low risk	Unclear risk	Unclear risk
Nar A 2009[35]	Turkey	Diet, exercise	24	Metformin	Diet, exercise	19	15	15	10	49.4(8.6)	44.5(5.9)	Newly diagnosed	Newly diagnosed	6.9(1.4)	6.1(1.1)	Initial:850; 2w:1700	Unclear risk	Unclear risk	High risk	Low risk	Low risk	Low risk	Unclear risk
Chen Y 2008a[36]	China	Diet, exercise	16	Metformin	Diet, exercise	17	17	8	7	42(4.7)	44(5.2)	Newly diagnosed	Newly diagnosed	8.2(2.6)	8.3(2.5)	1000	Unclear risk	Unclear risk	High risk	Low risk	Low risk	Unclear risk	Unclear risk
Cao Y 2007[37]	China	Diet, exercise	12	Metformin	Diet, exercise	30	30	19	18	53.6(12.9)	55.4(10.7)	9.7(3.6)	9.9(3.3)	7.45(1.59)	7.5(1.55)	1500	Unclear risk	Unclear risk	High risk	Low risk	Low risk	Low risk	Unclear risk
Deng HO 2007a[38]	China	None	12	Metformin	Diet	20	20	0	0	67(2.3)	68(2.1)	Newly diagnosed	Newly diagnosed	7.3(0.3)	7.3(0.2)	1000	Unclear risk	Unclear risk	High risk	Low risk	Low risk	Unclear risk	Unclear risk
Deng HO 2007b[38]	China	None	12	Metformin	Diet	20	20	0	0	66(2.2)	68(2.1)	Newly diagnosed	Newly diagnosed	7.4(0.2)	7.3(0.2)	1500	Unclear risk	Unclear risk	High risk	Low risk	Low risk	Unclear risk	Unclear risk
Li HZ 2007a[39]	China	Diet, exercise	12	Metformin	Diet, exercise	41	38	17	18	47.5(12.6)	46.2(10.4)	0.3(0.1)	0.32(0.17)	NR	NR	1500	Unclear risk	Unclear risk	High risk	Low risk	Low risk	Unclear risk	Unclear risk
Mei Q 2006[40]	China	Diet, exercise	16	Metformin	Diet, exercise	46	40	21	18	46.3	46.3	Newly diagnosed	Newly diagnosed	NR	NR	1500	Unclear risk	Unclear risk	High risk	Low risk	Low risk	Unclear risk	Unclear risk
Hanefeld M 2002a[41]	USA, Germany	Diet	16	Glyburide	Placebo	8	8	3	2	60.6(7.07)	59(4.53)	7.12(3.46)	6.77(4.53)	8.4(1.13)	8.7(1.70)	3	Unclear risk	Unclear risk	Low risk	Low risk	Low risk	Unclear risk	Unclear risk
Birkeland KI 1994a[42]	Norway, Sweden	Diet	60	Glyburide	Placebo	15	16	NR	NR	59(7)	59(7)	3.5(3.1)	3.5(3.1)	NR	NR	5.5	Unclear risk	Unclear risk	Low risk	Low risk	Low risk	Unclear risk	Unclear risk
Takami K 2002a[43]	Japan	Diet	12	Glyburide	Diet	9	11	3	4	50.8(8.6)	50.8(8.6)	Newly diagnosed	Newly diagnosed	9.1(2.5)	8.3(1.3)	1.25	Unclear risk	Unclear risk	High risk	Low risk	Low risk	Low risk	Unclear risk
Hassan MH 2015b[2]	Egypt	Diet, exercise	12	Glimepiride	Placebo	30	30	NR	NR	30~75	30~75	NR	NR	8.8(2.19)	9(2.74)	3	Unclear risk	Unclear risk	Low risk	Low risk	Low risk	Unclear risk	Unclear risk
Kaku K 2013A[44]	Japan	Diet, exercise	12	Glimepiride	Placebo	49	48	17	17	54.5(9.8)	58.8(8.3)	5.46(5.07)	6.41(6.09)	8.55(0.83)	8.5(0.92)	1	Unclear risk	Unclear risk	High risk	Low risk	Low risk	Unclear risk	Unclear risk

Bautista JL 2003[45]	USA	Diet, exercise	14	Glimepiride	Placebo	48	22	21	11	48.4(11.7)	50.7(10)	4.2(5.8)	5.7(8.4)	10.1(1.94)	10.6(2.55)	Initial:1; Max:4	Unclear risk	Unclear risk	Low risk	Low risk	High risk	Unclear risk	Unclear risk
Yu JX 2014[46]	China	None	14	Glimepiride	Diet, exercise	30	30	15	14	53.5(5.1)	54.7(5.3)	NR	NR	6.74(0.55)	7.06(0.93)	Initial:1, adjust dose based on glucose level	Unclear risk	Unclear risk	High risk	Low risk	Low risk	Low risk	Unclear risk
Hassan MH 2015c[2]	Egypt	Diet, exercise	12	Gliclazide	Placebo	30	30	NR	NR	30~75	30~75	NR	NR	8.9(2.74)	9(2.74)	80	Unclear risk	Unclear risk	Low risk	Low risk	Low risk	Unclear risk	Unclear risk
Wei JC 2010[47]	China	None	16	Glipizide	Placebo	60	60	36	39	55.4(7.6)	58.7(6.9)	7.1(2.4)	6.5(2.2)	7.8(1.1)	7.2(1.6)	30	Unclear risk	Unclear risk	High risk	Low risk	Low risk	Unclear risk	Unclear risk
Scott R 2007a[48]	USA, New Zealand	Diet, exercise	12	Glipizide	Placebo	123	125	53	47	54.7(10.7)	55.3(9.7)	4.7(4.2)	4.8(4.7)	7.82(0.95)	7.88(0.96)	Initial:5; Max:20	Low risk	Unclear risk	Low risk	Low risk	Low risk	Low risk	Unclear risk
Simonson DC 1997a[49]	USA	None	16	Glipizide	Placebo	68	69	28	16	57.4	60.2	6.90	7.5	8.5(1.62)	8.3(1.65)	5	Unclear risk	Unclear risk	Low risk	Low risk	Low risk	Unclear risk	Unclear risk
Simonson DC 1997b[49]	USA	None	16	Glipizide	Placebo	42	69	17	16	58.7	60.2	8.80	7.5	8.8(1.30)	8.3(1.65)	10	Unclear risk	Unclear risk	Low risk	Low risk	Low risk	Unclear risk	Unclear risk
Simonson DC 1997c[49]	USA	None	16	Glipizide	Placebo	42	69	16	16	55.5	60.2	6.50	7.5	8.6(1.28)	8.3(1.65)	15	Unclear risk	Unclear risk	Low risk	Low risk	Low risk	Unclear risk	Unclear risk
Simonson DC 1997d[49]	USA	None	16	Glipizide	Placebo	69	69	22	16	59.3	60.2	7.80	7.5	8.7(1.65)	8.3(1.65)	20	Unclear risk	Unclear risk	Low risk	Low risk	Low risk	Unclear risk	Unclear risk
Simonson DC 1997e[49]	USA	None	16	Glipizide	Placebo	28	69	11	16	61.7	60.2	6.60	7.5	8.4(1.53)	8.3(1.65)	40	Unclear risk	Unclear risk	Low risk	Low risk	Low risk	Unclear risk	Unclear risk
Birkeland KI 1994b[42]	Norway, Sweden	Diet	60	Glipizide	Placebo	15	16	NR	NR	59(7)	59(7)	3.5(3.1)	3.5(3.1)	NR	NR	9.4	Unclear risk	Unclear risk	Low risk	Low risk	Low risk	Unclear risk	Unclear risk
Oz GO 2010a[50]	Turkey	None	12	Rosiglitazone	Placebo	20	21	12	12	56.4(7.9)	56.4(7.9)	<0.5	<0.5	7.3(1.3)	7.3(0.9)	4	Unclear risk	Unclear risk	Low risk	Low risk	Low risk	Low risk	Unclear risk
Rahman S 2010[51]	Malaysia	None	52	Rosiglitazone	Placebo	11	11	NR	NR	30~65	30~65	Newly diagnosed	Newly diagnosed	7.5(1.7)	8.3(2.3)	4	Unclear risk	Unclear risk	Low risk	Low risk	Low risk	Low risk	Unclear risk
Oz O 2008a[52]	Turkey	None	12	Rosiglitazone	Placebo	11	10	5	5	55.2(7.7)	55.2(7.7)	<0.5	<0.5	7(1.07)	6.39(1.1)	4	Unclear risk	Unclear risk	Low risk	Low risk	Low risk	Low risk	Unclear risk
Albertini JP 2007[53]	Austria, France, Germany, Ireland, UK	Diet, exercise	12	Rosiglitazone	Placebo	64	71	22	26	55.5(8)	56.4(6.9)	4.3(5.2)	4.1(4.8)	NR	NR	8	Unclear risk	Unclear risk	Low risk	Low risk	High risk	Unclear risk	Unclear risk
Gastaldelli A 2006[54]	USA	Diet	12	Rosiglitazone	Placebo	13	13	6	5	53(2)	56(2)	4(2)	3(1)	8.6(1.80)	8.2(1.44)	8	Unclear risk	Unclear risk	Low risk	Low risk	Low risk	Unclear risk	Unclear risk
Karlsson HK 2005b[11]	Finland	Diet, exercise	26	Rosiglitazone	Placebo	10	11	2	2	58.2(6.64)	58.6(8.29)	Newly diagnosed	Newly diagnosed	6.8(0.95)	6.3(0.33)	Initial:4; 3w:8	Unclear risk	Unclear risk	Low risk	Low risk	Low risk	Low risk	Unclear risk
Viljanen AP 2005b[12]	Finland	Diet	26	Rosiglitazone	Placebo	14	11	5	2	58.6(7.7)	58.7(8.3)	NR	NR	6.8(0.9)	6.2(0.7)	Initial:4; 3w:8	Unclear risk	Unclear risk	Low risk	Low risk	Low risk	Unclear risk	Unclear risk
Chen HY 2004[55]	China	Diet	12	Rosiglitazone	Placebo	16	16	7	5	57(6.86)	58(9.72)	NR	NR	NR	NR	Initial:4; Max:8	Unclear risk	Unclear risk	Low risk	Low risk	Low risk	Unclear risk	Unclear risk
Juhl CB 2003[56]	Denmark	Diet, exercise	13	Rosiglitazone	Placebo	10	10	1	4	54(9)	54(9)	NR	NR	7(1.4)	6.8(1)	8	Unclear risk	Unclear risk	Low risk	Low risk	Low risk	Unclear risk	Unclear risk

Carey DG 2002[57]	Australia	Diet	16	Rosiglitazone	Placebo	16	17	2	4	54.2(11.1)	57.9(10.7)	3.3(4.5)	3.1(3.3)	7.8(1.3)	7.1(1.4)	8	Unclear risk	Unclear risk	Low risk	Low risk	Low risk	Unclear risk	Unclear risk
Haffner SM 2002a[58]	USA	Diet	26	Rosiglitazone	Placebo	126	95	33	42	60.7(9.3)	59.8(10.5)	4.7(6.1)	4.5(4.8)	8.8(1.4)	8.7(1.5)	4	Unclear risk	Unclear risk	Low risk	Low risk	Low risk	Unclear risk	Unclear risk
Haffner SM 2002b[58]	USA	Diet	26	Rosiglitazone	Placebo	136	95	35	42	60.4(9.3)	59.8(10.5)	4.9(5.2)	4.5(4.8)	8.6(1.5)	8.7(1.5)	8	Unclear risk	Unclear risk	Low risk	Low risk	Low risk	Unclear risk	Unclear risk
Lebovitz HE 2001a[59]	USA	Diet	26	Rosiglitazone	Placebo	166	158	59	54	60(9.8)	59(10.9)	4.8(5.8)	4.6(4.8)	9.0(1.5)	9.0(1.7)	4	Unclear risk	Unclear risk	Low risk	Low risk	Unclear risk	Unclear risk	Unclear risk
Lebovitz HE 2001b[59]	USA	Diet	26	Rosiglitazone	Placebo	169	158	56	54	61(9.5)	59(10.9)	5.4(6)	4.6(4.8)	8.8(1.6)	9.0(1.7)	8	Unclear risk	Unclear risk	Low risk	Low risk	Unclear risk	Unclear risk	Unclear risk
Miyazaki Y 2001[60]	USA	Diet	12	Rosiglitazone	Placebo	15	14	8	5	54(7.75)	56(7.48)	6(11.62)	4(3.74)	8.7(1.55)	8.3(1.50)	8	Unclear risk	Unclear risk	Low risk	Low risk	Low risk	Low risk	Unclear risk
Patel J 1999[61]	USA	Diet	12	Rosiglitazone	Placebo	80	75	25	23	59.7(10)	56.8(11.5)	5.80	4.2	9	9.1	4	Unclear risk	Unclear risk	Low risk	Low risk	Low risk	Unclear risk	Unclear risk
Huang D 2016[62]	China	None	16	Rosiglitazone	Diet, exercise	38	35	NR	NR	57.8(7.5)	58.4(6.8)	Newly diagnosed	Newly diagnosed	7.4(0.5)	7.8(0.6)	4	Unclear risk	Unclear risk	High risk	Low risk	Low risk	Low risk	Unclear risk
Qu JC 2011b[34]	China	Diet, exercise	12	Rosiglitazone	Diet, exercise	30	30	NR	NR	45(9)	45(9)	Newly diagnosed	Newly diagnosed	NR	NR	4	High risk	Unclear risk	High risk	Low risk	Low risk	Unclear risk	Unclear risk
Berberoglu Z 2010[63]	Turkey	Diet	104	Rosiglitazone	Diet	26	23	26	23	60.7(6.4)	59.8(7.6)	Newly diagnosed	Newly diagnosed	6.3(0.5)	6(0.5)	4	Unclear risk	Low risk	High risk	Low risk	Low risk	Unclear risk	Unclear risk
Lu XH 2009[64]	China	Diet	12	Rosiglitazone	Exercise	30	30	10	8	44.7(9.1)	44.2(8)	Newly diagnosed	Newly diagnosed	8.06(0.71)	8.03(0.69)	8	Unclear risk	Unclear risk	High risk	Low risk	Low risk	Low risk	Unclear risk
Zhu KS 2009[65]	China	Diet, exercise	12	Rosiglitazone	Diet, exercise	24	12	NR	NR	47.1(4.9)	46.2(4.4)	NR	NR	6.8(0.6)	6.7(0.5)	4	Unclear risk	Unclear risk	High risk	Low risk	Low risk	Unclear risk	Unclear risk
Berberoglu Z 2007[66]	Turkey	Diet	12	Rosiglitazone	Diet	28	28	28	28	59.7(6.35)	59.8(8.47)	Newly diagnosed	Newly diagnosed	6.34(0.53)	5.96(0.53)	4	Unclear risk	Low risk	High risk	Low risk	Low risk	Unclear risk	Unclear risk
Li HZ 2007b[39]	China	Diet, exercise	12	Rosiglitazone	Diet, exercise	38	38	17	18	47.1(9.8)	46.2(10.4)	0.35(0.15)	0.32(0.17)	NR	NR	8	Unclear risk	Unclear risk	High risk	Low risk	Low risk	Unclear risk	Unclear risk
Wang J 2020[67]	China	None	12	Pioglitazone	Placebo	53	53	23	20	66.26(3.33)	66.39(3.59)	Newly diagnosed	Newly diagnosed	NR	NR	30	High risk	Unclear risk	Low risk	Low risk	Low risk	Unclear risk	Unclear risk
Bajpeyi S 2017[68]	USA	None	12	Pioglitazone	Placebo	15	8	7	3	53(2.7)	55(3.2)	NR	NR	7.2(2.13)	6.9(1.02)	Initial:30; Max:45	Unclear risk	Unclear risk	Low risk	Low risk	Low risk	Low risk	Unclear risk
Tang XQ 2017[69]	China	Diet, exercise	12	Pioglitazone	Placebo	26	25	12	11	70.3(5.4)	71.5(5.3)	NR	NR	8.81(1.32)	8.83(1.26)	30	High risk	Unclear risk	Low risk	Low risk	Low risk	Low risk	Unclear risk
Sykes AP 2015A[70]	USA	None	12	Pioglitazone	Placebo	34	33	NR	NR	18~70	18~70	Newly diagnosed	Newly diagnosed	8.11(0.85)	8.19(0.81)	30	Unclear risk	Unclear risk	Low risk	Low risk	High risk	Unclear risk	Unclear risk
Sykes AP 2015B[71]	USA	None	12	Pioglitazone	Placebo	47	43	NR	NR	18~70	18~70	Newly diagnosed	Newly diagnosed	8.08(0.56)	8.12(0.76)	30	Unclear risk	Unclear risk	Low risk	Low risk	High risk	Unclear risk	Unclear risk
Chou HS 2012[72]	USA, Europe, India, South America, South Africa	None	26	Pioglitazone	Placebo	751	137	353	70	55(10.84)	55.4(12.32)	4.4(4.99)	4.9(6.13)	7.7(0.58)	7.7(0.55)	45	Unclear risk	Unclear risk	Low risk	Low risk	High risk	Unclear risk	Unclear risk

Kong AP 2011[73]	China	None	12	Pioglitazone	Placebo	37	32	16	13	53.6(7.6)	54(8.5)	5.59(4.6)	5.85(3.89)	7.49(0.82)	7.35(0.62)	30	Unclear risk	Unclear risk	Low risk	Low risk	Low risk	Low risk	Unclear risk
Oz GO 2010b[50]	Turkey	None	12	Pioglitazone	Placebo	19	21	11	12	56.4(7.9)	56.4(7.9)	<0.5	<0.5	7.6(1.5)	7.3(0.9)	30	Unclear risk	Unclear risk	Low risk	Low risk	Low risk	Low risk	Unclear risk
Wang B 2009[74]	China	Diet, exercise	12	Pioglitazone	Placebo	68	68	NR	NR	NR	NR	Newly diagnosed	Newly diagnosed	NR	NR	30	Unclear risk	Unclear risk	Low risk	Low risk	Unclear risk	Unclear risk	Unclear risk
Oz O 2008b[52]	Turkey	None	12	Pioglitazone	Placebo	14	10	7	5	55.2(7.7)	55.2(7.7)	<0.5	<0.5	7.82(1.7)	6.39(1.1)	30	Unclear risk	Unclear risk	Low risk	Low risk	Low risk	Low risk	Unclear risk
Gastaldelli A 2007[75]	Italy	Diet	16	Pioglitazone	Placebo	17	10	7	4	61(5.2)	61(5.2)	NR	NR	7(1.04)	7(1.04)	45	Unclear risk	Unclear risk	Low risk	Low risk	Low risk	Unclear risk	Unclear risk
Goldstein BJ 2006[76]	USA	None	12	Pioglitazone	Placebo	72	70	25	27	58.9(11.7)	56.1(10.6)	0.2~0.21	0.2~0.21	7(0.9)	7(0.8)	45	Low risk	Unclear risk	Low risk	Low risk	Low risk	Unclear risk	Unclear risk
Hao YR 2006a[77]	China	None	12	Pioglitazone	Placebo	8	8	NR	NR	30~60	30~60	NR	NR	NR	NR	15	Low risk	Unclear risk	Low risk	Low risk	Low risk	Unclear risk	Unclear risk
Hao YR 2006b[77]	China	None	12	Pioglitazone	Placebo	8	8	NR	NR	60~75	60~75	NR	NR	NR	NR	15	Low risk	Unclear risk	Low risk	Low risk	Low risk	Unclear risk	Unclear risk
Khan M 2006a[78]	USA	None	26	Pioglitazone	Placebo	22	21	8	15	56.4(9.95)	54.8(8.65)	NR	NR	8.46(1.45)	8.62(1.48)	15	Unclear risk	Unclear risk	Low risk	Low risk	Low risk	Unclear risk	Unclear risk
Khan M 2006b[78]	USA	None	26	Pioglitazone	Placebo	22	21	7	15	52.7(8.95)	54.8(8.65)	NR	NR	8.45(1.46)	8.62(1.48)	30	Unclear risk	Unclear risk	Low risk	Low risk	Low risk	Unclear risk	Unclear risk
Khan M 2006c[78]	USA	None	26	Pioglitazone	Placebo	23	21	9	15	56.7(7.17)	54.8(8.65)	NR	NR	9.54(1.56)	8.62(1.48)	45	Unclear risk	Unclear risk	Low risk	Low risk	Low risk	Unclear risk	Unclear risk
Pang WY 2006[79]	China	Diet, exercise	12	Pioglitazone	Placebo	28	28	14	15	68(5)	68(5)	Newly diagnosed	Newly diagnosed	8.8(1.6)	8.8(1.4)	30	Unclear risk	Unclear risk	Low risk	Low risk	Low risk	Unclear risk	Unclear risk
Wallace TM 2004[80]	UK	Diet	12	Pioglitazone	Placebo	19	11	5	3	61.4(27.46)	62.6(33.17)	2.60	2.5	6.7(3.92)	6.7(2.98)	Initial:30; 2w:45	Unclear risk	Unclear risk	Low risk	Low risk	Low risk	Low risk	Unclear risk
Liu CM 2003[81]	China	None	12	Pioglitazone	Placebo	40	40	18	18	53.4	53.4	0.33~10	0.33~10	9.6(2.5)	9.5(2.35)	Initial:15; Max:30	Unclear risk	Unclear risk	Low risk	Low risk	Low risk	Unclear risk	Unclear risk
Miyazaki Y 2002a[82]	USA	Diet	26	Pioglitazone	Placebo	12	11	4	8	57(4)	58(3)	NR	NR	8(1.04)	8.6(1.66)	15	Unclear risk	Unclear risk	Low risk	Low risk	Low risk	Low risk	Unclear risk
Miyazaki Y 2002b[82]	USA	Diet	26	Pioglitazone	Placebo	11	11	3	8	51(2)	58(3)	NR	NR	8.5(1.66)	8.6(1.66)	30	Unclear risk	Unclear risk	Low risk	Low risk	Low risk	Low risk	Unclear risk
Miyazaki Y 2002c[82]	USA	Diet	26	Pioglitazone	Placebo	11	11	6	8	55(2)	58(3)	NR	NR	9.1(0.99)	8.6(1.66)	45	Unclear risk	Unclear risk	Low risk	Low risk	Low risk	Low risk	Unclear risk
Scherbaum WA 2002a[83]	Germany	Diet	26	Pioglitazone	Placebo	89	84	33	37	58	59.1	5.40	5.6	9.33(1.18)	8.75(1.06)	15	Unclear risk	Unclear risk	Low risk	Low risk	High risk	Unclear risk	Unclear risk
Scherbaum WA 2002b[83]	Germany	Diet	26	Pioglitazone	Placebo	78	84	46	37	59.6	59.1	4.60	5.6	9.06(1.2)	8.75(1.06)	30	Unclear risk	Unclear risk	Low risk	Low risk	High risk	Unclear risk	Unclear risk
Aronoff S 2000a[84]	USA	Diet	26	Pioglitazone	Placebo	81	79	34	33	53.7	53.7	NR	NR	10.2(1.96)	10.4(1.96)	15	Unclear risk	Unclear risk	Low risk	Low risk	High risk	Unclear risk	Unclear risk

Aronoff S 2000b[84]	USA	Diet	26	Pioglitazone	Placebo	87	79	37	33	53.7	53.7	NR	NR	10.2(1.94)	10.4(1.96)	30	Unclear risk	Unclear risk	Low risk	Low risk	High risk	Unclear risk	Unclear risk
Aronoff S 2000c[84]	USA	Diet	26	Pioglitazone	Placebo	80	79	34	33	53.7	53.7	NR	NR	10.3(1.92)	10.4(1.96)	45	Unclear risk	Unclear risk	Low risk	Low risk	High risk	Unclear risk	Unclear risk
Wang DL 2020b[21]	China	Diet, exercise	52	Pioglitazone	Diet, exercise	60	60	21	24	48.2(5)	46.8(4.7)	NR	NR	8.59(1.09)	8.79(1.21)	15	Low risk	Unclear risk	High risk	Low risk	Low risk	Unclear risk	Unclear risk
Mirmiranpour H 2013[85]	Iran	Diet, exercise	12	Pioglitazone	Diet, exercise	30	49	19	29	51.16(1.47)	52.38(1.22)	Newly diagnosed	Newly diagnosed	7.5(1.31)	7.09(0.7)	30	Unclear risk	Unclear risk	High risk	Low risk	Low risk	Low risk	Unclear risk
Chen Y 2008b[36]	China	Diet, exercise	16	Pioglitazone	Diet, exercise	18	17	9	7	45(5.7)	44(5.2)	Newly diagnosed	Newly diagnosed	8.4(2.9)	8.3(2.5)	30	Unclear risk	Unclear risk	High risk	Low risk	Low risk	Unclear risk	Unclear risk
Bech P 2003[86]	Australia, Croatia, Czech Republic, France, Greece, Israel, Macedonia, Poland, Russia, Slovenia, Spain	Diet	16	Repaglinide	Placebo	164	89	70	37	56.9(8.6)	57.3(8.2)	2.77(4.2)	2.81(4.96)	7.8(1.7)	7.6(1.6)	1~2	Unclear risk	Unclear risk	Low risk	Low risk	Low risk	Unclear risk	Unclear risk
Xu L 2011[87]	China	Diet, exercise	12	Repaglinide	Diet, exercise	31	30	16	13	41.8(9.6)	39.5(4.9)	7.2(3.8)	8.1(2.3)	7.58(1.43)	7.49(1.15)	3	Unclear risk	Unclear risk	High risk	Low risk	Low risk	Unclear risk	Unclear risk
Zhong XY 2010[88]	China	Diet, exercise	12	Repaglinide	Diet, exercise	31	30	16	13	41.8(9.6)	39.5(4.9)	7.2(3.8)	8.1(2.3)	7.58(1.43)	7.49(1.15)	3	Unclear risk	Unclear risk	High risk	Low risk	Low risk	Unclear risk	Unclear risk
Guo XD 2009[89]	China	Diet, exercise	12	Repaglinide	Diet, exercise	31	30	16	13	41.8(9.6)	39.5(4.9)	7.2(3.8)	8.1(2.3)	7.58(1.43)	7.49(1.15)	3	Unclear risk	Unclear risk	High risk	Low risk	Low risk	Unclear risk	Unclear risk
Zhao W 2008[90]	China	Diet, exercise	12	Repaglinide	Diet, exercise	31	30	16	13	41.8(9.6)	39.5(4.9)	7.2(3.8)	8.1(2.3)	7.58(1.43)	7.49(1.15)	3	Unclear risk	Unclear risk	High risk	Low risk	Low risk	Unclear risk	Unclear risk
Gonzalez-Clemente JM 2008[91]	Spain	Diet, exercise	12	Nateglinide	Placebo	55	54	24	20	59.9(10.6)	57.2(10.7)	NR	NR	7.2(0.6)	7.1(0.7)	360	Unclear risk	Low risk	Low risk	Low risk	Low risk	Low risk	Unclear risk
Schwarz SL 2008[92]	USA	None	12	Nateglinide	Placebo	30	24	10	13	74.4(5.1)	74.5(6.5)	3(5.2)	2.5(3.2)	7.6(0.55)	7.7(0.98)	360	Unclear risk	Unclear risk	Low risk	Low risk	High risk	Unclear risk	Unclear risk
Mari A 2005a[93]	Argentina, Australia, Belgium, Canada, Finland, France, Germany, Italy, Netherlands, New Zealand, Sweden, USA	Diet, exercise	24	Nateglinide	Placebo	27	30	8	11	61(8)	60(11)	4.3(3.6)	4.5(5)	6.6(0.6)	6.5(0.6)	180	Unclear risk	Unclear risk	Low risk	Low risk	High risk	Unclear risk	Unclear risk
Mari A 2005b[93]	Argentina, Australia, Belgium, Canada, Finland, France, Germany, Italy, Netherlands, New Zealand, Sweden, USA	Diet, exercise	24	Nateglinide	Placebo	25	30	10	11	62(9)	60(11)	5.2(6.7)	4.5(5)	6.6(0.7)	6.5(0.6)	360	Unclear risk	Unclear risk	Low risk	Low risk	High risk	Unclear risk	Unclear risk
Horton ES 2004b[13]	USA	Diet, exercise	24	Nateglinide	Placebo	104	104	45	37	57.9(10.2)	59(11.22)	4.7(6.12)	4.2(4.08)	8.1(0.99)	8.2(0.99)	120	Unclear risk	Unclear risk	Low risk	Low risk	Low risk	Unclear risk	Unclear risk
Kato T 2010a[94]	Japan	Diet	12	Nateglinide	Diet	10	10	3	5	67.8(8.6)	68(7.7)	NR	NR	6.1(0.6)	5.8(0.6)	270	Unclear risk	Unclear risk	High risk	Low risk	Low risk	Low risk	Unclear risk
Kirkman MS 2006[95]	USA	Diet	52	Acarbose	Placebo	109	110	73	72	53.7(11)	53.7(11.7)	NR	NR	6.35(0.64)	6.32(0.62)	Initial:25; Max:300	Unclear risk	Unclear risk	Low risk	Low risk	High risk	Unclear risk	Unclear risk
Josse RG 2003[96]	Canada	Diet	52	Acarbose	Placebo	93	99	28	39	69.7(4.82)	70.3(4.97)	5.8(6.75)	4.8(4.97)	7.4(0.96)	7.3(0.99)	Initial:50; Max:300	Unclear risk	Unclear risk	Low risk	Low risk	Low risk	Unclear risk	Unclear risk

Wu GT 2003[97]	China	Diet, exercise	24	Acarbose	Placebo	80	80	35	33	50(7)	50(6)	4.2(2.1)	4.3(2.4)	9.8(2.1)	9.6(2.7)	Initial:150; 3w:300	Low risk	Low risk	Low risk	Low risk	Low risk	Unclear risk	Unclear risk
Hanefeld M 2002b[41]	USA, Germany	Diet	16	Acarbose	Placebo	11	8	1	2	60.4(4.31)	59(4.53)	7.7(4.48)	6.77(4.53)	8.2(0.99)	8.7(1.70)	300	Unclear risk	Unclear risk	Low risk	Low risk	Low risk	Unclear risk	Unclear risk
Meneilly GS 2000[98]	Canada, USA	Diet	52	Acarbose	Placebo	22	23	NR	NR	68(4.69)	70(4.8)	NR	NR	7.3(0.47)	7(0.96)	Initial:150; Max:300	Unclear risk	Unclear risk	Low risk	Low risk	Low risk	Unclear risk	Unclear risk
Scott R 1999[99]	Australia, New Zealand	Diet	16	Acarbose	Placebo	53	52	20	18	56(9)	57(8)	1.75(1.25)	2.17(1.42)	7(0.87)	6.89(0.85)	Initial:150; Max:300	Unclear risk	Unclear risk	Low risk	Low risk	Low risk	Unclear risk	Unclear risk
Chan JCN 1998[100]	Taiwan, Hong Kong, Philippines, Korea, Singapore, Malaysia	Diet	24	Acarbose	Placebo	63	63	31	31	52.8(10.2)	54(10)	2.7(3.5)	2.1(3.4)	8.2(1)	8.6(1.1)	Initial:150; 4w:300	Unclear risk	Unclear risk	Low risk	Low risk	Low risk	Low risk	Unclear risk
Fischer S 1998a[101]	Austria, Croatia, Germany, Hungary, Italy	Diet	24	Acarbose	Placebo	88	81	45	38	55.5(9.6)	52.7(8.7)	1.67	2.00	7.52(1.09)	7.46(1.09)	150	Unclear risk	Unclear risk	Low risk	Low risk	Low risk	Unclear risk	Unclear risk
Fischer S 1998b[101]	Austria, Croatia, Germany, Hungary, Italy	Diet	24	Acarbose	Placebo	78	81	32	38	56.8(9.4)	52.7(8.7)	1.42	2.00	7.43(1.1)	7.46(1.09)	300	Unclear risk	Unclear risk	Low risk	Low risk	Low risk	Unclear risk	Unclear risk
Hoffmann J 1997b[20]	Germany	Diet	24	Acarbose	Placebo	31	32	25	20	58.9(9.4)	60.2(8.6)	3.08(2.27)	3.6(2.83)	9.6(0.9)	9.4(0.9)	300	Low risk	Unclear risk	Low risk	Low risk	Low risk	Unclear risk	Unclear risk
Braun D 1996[102]	Germany	Diet	24	Acarbose	Placebo	42	44	16	20	60	61	1.33	1.42	10(1.5)	9.9(1.5)	Initial:150; 3w:300	Low risk	Unclear risk	Low risk	Low risk	Low risk	Unclear risk	Unclear risk
Hou WK 1996[103]	China	Diet	12	Acarbose	Placebo	50	50	NR	NR	57.8(6.7)	58.6(5.8)	0.65(0.1)	0.63(0.13)	NR	NR	Initial:150; 5w:300	Unclear risk	Unclear risk	Low risk	Low risk	Low risk	Unclear risk	Unclear risk
Zheng GF 1995[104]	China	Diet, exercise	24	Acarbose	Placebo	39	38	19	18	49.6(6.9)	49(6.6)	4.1(2.8)	4.2(3.6)	9.85(2.37)	9.82(2.16)	Initial:150; 4w:300	Unclear risk	Low risk	Low risk	Low risk	Low risk	Unclear risk	Unclear risk
Hotta N 1993[105]	Japan	Diet	24	Acarbose	Placebo	19	18	5	4	49.8	47.9	4.60	4.80	11.1(2.18)	10.3(1.70)	300	Unclear risk	Unclear risk	Low risk	Low risk	Low risk	Low risk	Unclear risk
Santeusanio F 1993a[106]	Italy	Diet	16	Acarbose	Placebo	18	23	8	7	58.9(9.76)	55.5(11.51)	3.85(2.97)	3.87(4.32)	7.07(0.72)	7.22(0.91)	150	Low risk	Unclear risk	Low risk	Low risk	High risk	Unclear risk	Unclear risk
Santeusanio F 1993b[106]	Italy	Diet	16	Acarbose	Placebo	23	23	8	7	53.8(11.03)	55.5(11.51)	5.05(4.8)	3.87(4.32)	7.15(0.86)	7.22(0.91)	300	Low risk	Unclear risk	Low risk	Low risk	High risk	Unclear risk	Unclear risk
Hanefeld M 1991[107]	Germany	Diet	24	Acarbose	Placebo	47	47	24	22	60	59	5.83	4.08	9.3(1.38)	9.4(1.14)	300	Unclear risk	Unclear risk	Low risk	Low risk	Low risk	Low risk	Unclear risk
Chen DL 2017[108]	China	Diet, exercise	12	Acarbose	Diet, exercise	40	40	21	18	57.1(4.4)	58.3(4.1)	0.5~5	0.33~5	NR	NR	150	Low risk	Unclear risk	High risk	Low risk	Low risk	Unclear risk	Unclear risk
Yang LH 2015[109]	China	Diet, exercise	12	Acarbose	Diet, exercise	50	50	21	22	58.39(4.3)	56.42(4.67)	4.29(0.29)	3.97(0.33)	NR	NR	150	Low risk	Unclear risk	High risk	Low risk	Low risk	Unclear risk	Unclear risk
Wu HX 2014[110]	China	Diet, exercise	12	Acarbose	Diet, exercise	31	31	7	6	58.1(5.2)	57.8(4.8)	5.7(2.4)	5.6(2.6)	8.9(1.3)	8.6(1.5)	150	Unclear risk	Unclear risk	High risk	Low risk	Low risk	Unclear risk	Unclear risk
Yang Y 2014[111]	China	Diet, exercise	12	Acarbose	Diet, exercise	60	60	28	27	44.35(12.86)	45.28(12.63)	6.62(2.93)	6.72(2.95)	9.94(1.36)	9.88(1.27)	150	Low risk	Unclear risk	High risk	Low risk	Low risk	Unclear risk	Unclear risk
Hao HR 2012[112]	China	Diet, exercise	16	Acarbose	Diet, exercise	48	35	20	16	44.9(6.9)	47.8(5.3)	0.5~1	0.5~1	NR	NR	150	Low risk	Unclear risk	High risk	Low risk	Low risk	Unclear risk	Unclear risk

Kato T 2010b[94]	Japan	Diet	12	Acarbose	Diet	10	10	3	5	67.6(6.2)	68(7.7)	NR	NR	6(0.3)	5.8(0.6)	300	Unclear risk	Unclear risk	High risk	Low risk	Low risk	Low risk	Unclear risk
Ye XH 2009[113]	China	Diet, exercise	12	Acarbose	Diet, exercise	29	29	16	15	55.9(13.5)	56.2(15.2)	Newly diagnosed	Newly diagnosed	7.9(1.5)	7.8(1.6)	150	Low risk	Unclear risk	High risk	Low risk	Low risk	Unclear risk	Unclear risk
Seino Y 2011a[114]	Japan	Diet, exercise	12	Voglibose	Placebo	83	75	27	19	59.1(10.31)	59.1(10.47)	5.85(5.95)	6.83(6.07)	7.99(0.82)	7.85(0.89)	0.6	Low risk	Unclear risk	Low risk	Low risk	Low risk	Low risk	Unclear risk
Guo HG 2001[115]	China	Diet, exercise	24	Voglibose	Placebo	42	41	20	18	51.2(7.1)	47.6(6.1)	4.5(2.9)	5.3(3.9)	10.31(0.41)	10.13(0.36)	Initial:0.6; 4w:1.2	Unclear risk	Low risk	Low risk	Low risk	Low risk	Unclear risk	Unclear risk
Takami K 2002b[43]	Japan	Diet	12	Voglibose	Diet	12	11	3	4	50.8(8.6)	50.8(8.6)	Newly diagnosed	Newly diagnosed	8(1.7)	8.3(1.3)	0.9	Unclear risk	Unclear risk	High risk	Low risk	Low risk	Low risk	Unclear risk
Drent ML 2002a[116]	Netherlands	Diet	24	Miglitol	Placebo	84	87	37	38	63(9)	63(11)	4.00	2.50	8.09(1.23)	7.94(1.15)	150	Low risk	Unclear risk	Low risk	Low risk	Low risk	Unclear risk	Unclear risk
Drent ML 2002b[116]	Netherlands	Diet	24	Miglitol	Placebo	71	87	34	38	63(11)	63(11)	3.00	2.50	8(1.21)	7.94(1.15)	Initial:150; 3w:300	Low risk	Unclear risk	Low risk	Low risk	Low risk	Unclear risk	Unclear risk
Chiasson JL 2001b[16]	Canada, France	Diet, exercise	36	Miglitol	Placebo	82	83	18	27	57.3(9)	57.7(9.9)	5.2(4.7)	5.1(4.9)	8.2(0.9)	8.1(0.7)	Initial:75; Max:300	Unclear risk	Unclear risk	Low risk	Low risk	Low risk	Unclear risk	Unclear risk
Gantz I 2017[117]	Japan	None	24	Sitagliptin	Placebo	165	83	50	26	60(9)	61(9)	7.4(5.3)	8.6(5.1)	8(0.8)	8.1(0.7)	50	Low risk	Low risk	Low risk	Low risk	Low risk	Low risk	Unclear risk
Ji L 2016c[1]	China	Diet, exercise	24	Sitagliptin	Placebo	120	127	46	40	51.7(10.2)	53.6(9.7)	1.1(0.2)	1.1(0.2)	8.7(1.1)	9(1.1)	100	Low risk	Unclear risk	Low risk	Low risk	Low risk	Low risk	Unclear risk
Tian M 2016[118]	China	Diet, exercise	12	Sitagliptin	Placebo	94	47	46	23	54(8.1)	54(8.1)	Newly diagnosed	Newly diagnosed	8.08(0.6)	8.16(0.48)	100	Unclear risk	Unclear risk	Low risk	Low risk	High risk	Low risk	Unclear risk
Roden M 2015a[119]	USA, Belgium, Canada, China, France, Germany, Greece, India, Ireland, Japan, Korea, Mexico, Philippines, Slovakia, Slovenia, Switzerland, Taiwan, Turkey, Ukraine	Diet, exercise	76	Sitagliptin	Placebo	223	228	82	105	55.1(9.9)	54.9(10.9)	NR	NR	7.85(0.79)	7.91(0.78)	100	Low risk	Low risk	Low risk	Low risk	Low risk	Low risk	Unclear risk
Roden M 2013a[120]	Belgium, Canada, China, Germany, India, Ireland, Japan, Switzerland, USA	Diet, exercise	24	Sitagliptin	Placebo	223	228	82	105	55.1(9.9)	54.9(10.9)	NR	NR	7.85(0.79)	7.91(0.78)	100	Low risk	Low risk	Low risk	Low risk	Low risk	Low risk	Unclear risk
Sun J 2013[121]	China	Diet, exercise	12	Sitagliptin	Placebo	23	23	12	12	48.2(6.2)	48.2(6.2)	NR	NR	8.22(0.78)	8.13(0.86)	100	Unclear risk	Unclear risk	Low risk	Low risk	Low risk	Unclear risk	Unclear risk
Barzilai N 2011[122]	USA	Diet, exercise	24	Sitagliptin	Placebo	102	104	54	55	71.6(6.1)	72.1(6)	7.2(7.3)	7(7.5)	7.8(0.8)	7.7(0.7)	50 or 100	Low risk	Unclear risk	Low risk	Low risk	High risk	Low risk	Unclear risk
Iwamoto Y 2010a[123]	Japan	Diet, exercise	12	Sitagliptin	Placebo	72	73	25	23	60.2(9.4)	60.2(8)	5.6(6.4)	6.4(5.5)	7.57(0.84)	7.74(0.93)	50	Low risk	Low risk	Low risk	Low risk	Low risk	Low risk	Unclear risk
Iwamoto Y 2010b[123]	Japan	Diet, exercise	12	Sitagliptin	Placebo	70	73	34	23	58.3(9.5)	60.2(8)	5.4(5.4)	6.4(5.5)	7.56(0.8)	7.74(0.93)	100	Low risk	Low risk	Low risk	Low risk	Low risk	Low risk	Unclear risk
Mohan V 2009[124]	China, India, Korea	Diet, exercise	18	Sitagliptin	Placebo	352	178	152	72	50.9(9.3)	50.9(9.3)	2.1(1.7)	1.9(1.6)	8.7(1)	8.7(1)	100	Low risk	Low risk	Low risk	Low risk	Low risk	Low risk	Unclear risk
Nonaka K 2008[125]	Japan	Diet, exercise	12	Sitagliptin	Placebo	75	76	30	26	55.6(8.6)	55(8)	4(4.1)	4.1(4.6)	7.54(0.85)	7.69(0.86)	100	Low risk	Unclear risk	Low risk	Low risk	Low risk	Low risk	Unclear risk

Goldstein BJ 2007c[8]	Australia, Chile, Colombia, Costa Rica, Guatemala, Hungary, Lithuania, Malaysia, Mexico, New Zealand, Norway, Peru, Philippines, Russia, South Africa, UK, USA	Diet, exercise	24	Sitagliptin	Placebo	175	165	NR	NR	18~78	18~78	4.50	4.50	8.87(0.99)	8.68(1)	100	Unclear risk	Unclear risk	Low risk	Low risk	Low risk	Low risk	Unclear risk
Hanefeld M 2007a[126]	USA, Belgium	Diet, exercise	12	Sitagliptin	Placebo	112	111	61	41	55.3(10.3)	55.9(9.3)	3.3(3.9)	3.3(3.4)	7.6(0.94)	7.59(0.89)	50	Unclear risk	Unclear risk	Low risk	Low risk	High risk	Unclear risk	Unclear risk
Hanefeld M 2007b[126]	USA, Belgium	Diet, exercise	12	Sitagliptin	Placebo	110	111	49	41	56(7.9)	55.9(9.3)	3.6(3.9)	3.3(3.4)	7.78(0.9)	7.59(0.89)	100	Unclear risk	Unclear risk	Low risk	Low risk	High risk	Unclear risk	Unclear risk
Hanefeld M 2007c[126]	USA, Belgium	Diet, exercise	12	Sitagliptin	Placebo	111	111	62	41	55.2(9.5)	55.9(9.3)	4.5(5.9)	3.3(3.4)	7.79(0.85)	7.59(0.89)	100	Unclear risk	Unclear risk	Low risk	Low risk	High risk	Unclear risk	Unclear risk
Scott R 2007b[48]	USA, New Zealand	Diet, exercise	12	Sitagliptin	Placebo	123	125	52	47	55.6(9)	55.3(9.7)	5(5.2)	4.8(4.7)	7.89(0.94)	7.88(0.96)	50	Low risk	Unclear risk	Low risk	Low risk	Low risk	Low risk	Unclear risk
Scott R 2007c[48]	USA, New Zealand	Diet, exercise	12	Sitagliptin	Placebo	124	125	59	47	55.1(9.8)	55.3(9.7)	4.2(4)	4.8(4.7)	7.83(0.95)	7.88(0.96)	100	Low risk	Unclear risk	Low risk	Low risk	Low risk	Low risk	Unclear risk
Aschner P 2006[127]	USA, Belgium	Diet, exercise	24	Sitagliptin	Placebo	238	253	102	123	53.4(9.5)	54.3(10.1)	4.40	4.40	8.01(0.88)	8.03(0.82)	100	Unclear risk	Unclear risk	Low risk	Low risk	Low risk	Low risk	Unclear risk
Raz I 2006[128]	NR	Diet, exercise	18	Sitagliptin	Placebo	205	110	95	41	54.5(10)	55.5(10.1)	4.5(4.3)	4.7(5)	8.04(0.82)	8.05(0.9)	100	Unclear risk	Unclear risk	Low risk	Low risk	Low risk	Unclear risk	Unclear risk
Deng X 2017[129]	China	None	52	Sitagliptin	Diet, exercise	36	36	8	10	63.7(10.7)	64.1(11.2)	<2	<2	7.3(0.7)	7.4(0.8)	Initial:50; Max:100	Low risk	Low risk	High risk	Low risk	Low risk	Unclear risk	Unclear risk
Zhang JB 2016[130]	China	None	52	Saxagliptin	Placebo	24	23	12	11	44(3.1)	45(2.1)	Newly diagnosed	Newly diagnosed	8.31(0.83)	8.33(0.89)	5	Unclear risk	Unclear risk	Low risk	Low risk	Low risk	Unclear risk	Unclear risk
Xiu SL 2015[131]	China	Diet, exercise	16	Saxagliptin	Placebo	30	30	11	13	49(8)	49(8)	0.26(0.05)	0.23(0.06)	8.5(0.5)	8.5(0.4)	5	Low risk	Unclear risk	Low risk	Low risk	Low risk	Unclear risk	Unclear risk
Han BY 2014[132]	China	Diet, exercise	12	Saxagliptin	Placebo	40	40	17	18	46.9(8)	45.7(9.7)	Newly diagnosed	Newly diagnosed	8.8(0.97)	8.9(0.82)	5	Low risk	Unclear risk	Low risk	Low risk	Low risk	Unclear risk	Unclear risk
Prasanna Kumar KM 2014[133]	India	Diet, exercise	24	Saxagliptin	Placebo	107	106	50	43	49.1(8.8)	48.3(9.6)	0.8(1.2)	1(1.4)	8.33(0.82)	8.33(0.72)	5	Low risk	Low risk	Low risk	Low risk	Low risk	Low risk	Unclear risk
Wu YF 2013[134]	China	Diet, exercise	24	Saxagliptin	Placebo	11	10	6	3	49.55(9.44)	51.6(14.92)	Newly diagnosed	Newly diagnosed	8.31(0.84)	8.33(0.9)	5	Unclear risk	Unclear risk	Low risk	Low risk	Low risk	Unclear risk	Unclear risk
Frederich R 2012a[135]	USA, Russia, India, Taiwan	Diet, exercise	24	Saxagliptin	Placebo	74	74	49	39	55.2(10.44)	55.6(10.32)	1.2(1.6)	1.7(2.8)	8(0.90)	7.8(0.91)	2.5	Unclear risk	Unclear risk	Low risk	Low risk	Low risk	Low risk	Unclear risk
Frederich R 2012b[135]	USA, Russia, India, Taiwan	Diet, exercise	24	Saxagliptin	Placebo	74	74	36	39	54.7(9.71)	55.6(10.32)	1.7(2.4)	1.7(2.8)	7.9(0.91)	7.8(0.91)	5	Unclear risk	Unclear risk	Low risk	Low risk	Low risk	Low risk	Unclear risk
Frederich R 2012c[135]	USA, Russia, India, Taiwan	Diet, exercise	24	Saxagliptin	Placebo	71	74	34	39	54.3(10.93)	55.6(10.32)	2(2.9)	1.7(2.8)	8(1.08)	7.8(0.91)	Initial:2.5; Max:5	Unclear risk	Unclear risk	Low risk	Low risk	Low risk	Low risk	Unclear risk
Frederich R 2012d[135]	USA, Russia, India, Taiwan	Diet, exercise	24	Saxagliptin	Placebo	72	74	39	39	55.1(10.35)	55.6(10.32)	2(5.2)	1.7(2.8)	7.9(0.92)	7.8(0.91)	5	Unclear risk	Unclear risk	Low risk	Low risk	Low risk	Low risk	Unclear risk
Pan CY 2012[136]	China, India, Philippines, South Korea	Diet, exercise	24	Saxagliptin	Placebo	284	284	124	129	51.2(10)	51.6(10.3)	0.8(1.4)	1.2(2.6)	8.15(0.83)	8.14(0.83)	5	Low risk	Unclear risk	Low risk	Low risk	Low risk	Low risk	Unclear risk

Rosenstock J 2009a[137]	USA, Australia, Canada, Mexico, Taiwan	Diet, exercise	24	Saxagliptin	Placebo	102	95	44	48	53.27(10.06)	53.91(12.32)	3.1(3.5)	2.3(2.7)	7.9(0.9)	7.9(0.9)	2.5	Unclear risk	Unclear risk	Low risk	Low risk	High risk	Low risk	Unclear risk
Rosenstock J 2009b[137]	USA, Australia, Canada, Mexico, Taiwan	Diet, exercise	24	Saxagliptin	Placebo	106	95	52	48	53.91(11.57)	53.91(12.32)	2.5(3.3)	2.3(2.7)	8(1.1)	7.9(0.9)	5	Unclear risk	Unclear risk	Low risk	Low risk	High risk	Low risk	Unclear risk
Rosenstock J 2008a[138]	USA	Diet	12	Saxagliptin	Placebo	55	67	33	25	52.5(10.53)	55.2(9.8)	1.00	1.80	7.7(0.97)	8(0.98)	2.5	Unclear risk	Unclear risk	Low risk	Low risk	Low risk	Unclear risk	Unclear risk
Rosenstock J 2008b[138]	USA	Diet	12	Saxagliptin	Placebo	47	67	22	25	53.7(10.14)	55.2(9.8)	0.80	1.80	7.9(1.09)	8(0.98)	5	Unclear risk	Unclear risk	Low risk	Low risk	Low risk	Unclear risk	Unclear risk
Mari A 2008[139]	Finland, France, Germany, Romania, Spain, Sweden	Diet, exercise	52	Vildagliptin	Placebo	156	150	63	61	63.3(10.2)	62.8(11)	2.5(2.9)	2.7(3.2)	6.7(0.4)	6.8(0.4)	50	Unclear risk	Unclear risk	Low risk	Low risk	Low risk	Low risk	Unclear risk
Scherbaum WA 2008A[140]	Finland, France, Germany, Romania, Spain, Sweden	Diet, exercise	52	Vildagliptin	Placebo	156	150	63	61	63.3(10.2)	62.8(11)	2.5(2.9)	2.7(3.2)	6.7(0.4)	6.8(0.4)	50	Unclear risk	Unclear risk	Low risk	Low risk	Low risk	Low risk	Unclear risk
Scherbaum WA 2008B[141]	Finland, France, Germany, Romania, Spain, Sweden	Diet, exercise	108	Vildagliptin	Placebo	68	63	27	26	63.1(9.6)	63.2(10)	2.1(2.1)	2.5(2.6)	6.6(0.4)	6.7(0.4)	50	Unclear risk	Unclear risk	Low risk	Low risk	High risk	Low risk	Unclear risk
Dejager S 2007a[142]	USA, Russia, Tunisia	None	24	Vildagliptin	Placebo	104	94	61	49	55.3(11.4)	52.2(11.2)	2.1(3.6)	1.6(2.5)	8.2(1.02)	8.4(0.97)	50	Unclear risk	Unclear risk	Low risk	Low risk	Low risk	Low risk	Unclear risk
Dejager S 2007b[142]	USA, Russia, Tunisia	None	24	Vildagliptin	Placebo	90	94	48	49	52.8(9.6)	52.2(11.2)	2.1(3.3)	1.6(2.5)	8.6(0.95)	8.4(0.97)	100	Unclear risk	Unclear risk	Low risk	Low risk	Low risk	Low risk	Unclear risk
Dejager S 2007c[142]	USA, Russia, Tunisia	None	24	Vildagliptin	Placebo	92	94	43	49	53.6(10.8)	52.2(11.2)	2.4(4.2)	1.6(2.5)	8.4(0.96)	8.4(0.97)	100	Unclear risk	Unclear risk	Low risk	Low risk	Low risk	Low risk	Unclear risk
Pi-Sunyer FX 2007a[143]	USA, India, Slovakia	None	24	Vildagliptin	Placebo	88	92	39	42	50.6(10.4)	52(12)	1.8(2.7)	2.5(3.7)	8.3(0.92)	8.5(0.94)	50	Unclear risk	Unclear risk	Low risk	Low risk	Low risk	Low risk	Unclear risk
Pi-Sunyer FX 2007b[143]	USA, India, Slovakia	None	24	Vildagliptin	Placebo	83	92	36	42	50.2(12.7)	52(12)	2.4(3.2)	2.5(3.7)	8.4(0.89)	8.5(0.94)	100	Unclear risk	Unclear risk	Low risk	Low risk	Low risk	Low risk	Unclear risk
Pi-Sunyer FX 2007c[143]	USA, India, Slovakia	None	24	Vildagliptin	Placebo	91	92	42	42	52(11.7)	52(12)	2.1(2.9)	2.5(3.7)	8.3(0.94)	8.5(0.94)	100	Unclear risk	Unclear risk	Low risk	Low risk	Low risk	Low risk	Unclear risk
Pratley RE 2006[144]	South America, Mexico	Diet, exercise	12	Vildagliptin	Placebo	70	28	42	14	56.9(9.4)	52.8(10)	4.6(5.6)	3.5(5.7)	8(0.9)	8.1(1.2)	50	Unclear risk	Unclear risk	Low risk	Low risk	Low risk	Unclear risk	Unclear risk
Ristic S 2005a[145]	USA, Russia	None	12	Vildagliptin	Placebo	51	58	27	25	55.6(10.9)	54.6(10.6)	3.28(3.81)	2.28(2.99)	7.64(0.69)	7.76(0.83)	50	Unclear risk	Unclear risk	Low risk	Low risk	Low risk	Unclear risk	Unclear risk
Ristic S 2005b[145]	USA, Russia	None	12	Vildagliptin	Placebo	53	58	27	25	57(10.2)	54.6(10.6)	2.71(3.24)	2.28(2.99)	7.7(0.82)	7.76(0.83)	50	Unclear risk	Unclear risk	Low risk	Low risk	Low risk	Unclear risk	Unclear risk
Ristic S 2005c[145]	USA, Russia	None	12	Vildagliptin	Placebo	63	58	28	25	56.2(10.1)	54.6(10.6)	3.03(4.22)	2.28(2.99)	7.64(0.75)	7.76(0.83)	100	Unclear risk	Unclear risk	Low risk	Low risk	Low risk	Unclear risk	Unclear risk
Chen J 2016[146]	China	Diet, exercise	26	Vildagliptin	Diet, exercise	29	29	14	12	56.4(4.8)	54.7(6.2)	Newly diagnosed	Newly diagnosed	8.7(3.4)	8.9(4.1)	100	Low risk	Unclear risk	High risk	Low risk	Low risk	Unclear risk	Unclear risk
Lin N 2017[147]	China	Diet, exercise	24	Linagliptin	Placebo	45	45	25	23	54.1(8.22)	53.83(7.71)	NR	NR	NR	NR	5	Low risk	Unclear risk	Low risk	Low risk	Low risk	Unclear risk	Unclear risk
Chen Y 2015[148]	China, Malaysia, Philippines	None	24	Linagliptin	Placebo	200	99	84	40	54.6(10.1)	54.1(9.3)	NR	NR	7.95(0.89)	8.09(0.91)	5	Low risk	Low risk	Low risk	Low risk	Low risk	Low risk	Unclear risk

Wu W 2015[149]	China	Health education	24	Linagliptin	Placebo	34	23	12	12	52.5(11)	51.2(7.5)	Newly diagnosed	Newly diagnosed	7.97(0.68)	8(0.69)	5	Low risk	Low risk	Low risk	Low risk	Low risk	Low risk	Unclear risk
Wu WJ 2014[150]	China	Diet, exercise	24	Linagliptin	Placebo	27	23	18	13	52.3(12.7)	50.7(9.5)	Newly diagnosed	Newly diagnosed	7.93(0.86)	8.03(0.96)	5	Low risk	Unclear risk	Low risk	Low risk	Low risk	Low risk	Unclear risk
Barnett AH 2012[151]	Canada, Mexico, Philippines, Romania, Russia, Ukraine, USA	None	18	Linagliptin	Placebo	151	76	96	43	56.4(10.6)	56.7(9.7)	NR	NR	8.1(1)	8.1(0.9)	5	Low risk	Low risk	Low risk	Low risk	Low risk	Low risk	Unclear risk
Haak T 2012c[5]	Germany, UK	None	24	Linagliptin	Placebo	142	72	62	36	56.2(10.8)	55.7(11)	NR	NR	8.7(1)	8.7(1)	5	Unclear risk	Unclear risk	Low risk	Low risk	Low risk	Low risk	Unclear risk
Kawamori R 2012[152]	Japan	None	12	Linagliptin	Placebo	159	80	48	23	60.3(9.4)	59.7(8.9)	NR	NR	8.07(0.63)	7.95(0.63)	5	Unclear risk	Unclear risk	Low risk	Low risk	Low risk	Low risk	Unclear risk
Del Prato S 2011[153]	Croatia, India, Italy, Israel, Malaysia, Poland, Romania, Slovakia, Ukraine, Thailand, Netherlands	None	24	Linagliptin	Placebo	336	167	172	88	56.4(10.1)	54.4(10.3)	NR	NR	8(0.87)	8(0.86)	5	Low risk	Low risk	Low risk	Low risk	Low risk	Low risk	Unclear risk
Pan C 2017[154]	China, Taiwan, Hong Kong	Diet, exercise	16	Alogliptin	Placebo	92	92	37	38	51.6(10.4)	53.1(8.9)	1.9(2.4)	2.1(2.8)	8.04(0.92)	7.86(0.78)	25	Low risk	Low risk	Low risk	Low risk	Low risk	Low risk	Unclear risk
Inagaki N 2015[155]	Japan	Diet, exercise	24	Alogliptin	Placebo	92	50	23	7	60	62	7.07(5.93)	7.54(5.5)	7.87(0.86)	7.72(0.77)	25	Low risk	Low risk	Low risk	Low risk	Low risk	Low risk	Unclear risk
Pratley RE 2014c[3]	USA, Czech Republic, Hungary, Israel, Lithuania, Mexico, Poland, Romania, Russia, Slovakia, South Africa, Ukraine	Diet, exercise	26	Alogliptin	Placebo	112	109	48	55	52.6(9.38)	53.1(9.6)	3.6(4.12)	4.3(4.78)	NR	NR	25	Unclear risk	Unclear risk	Low risk	Low risk	High risk	Low risk	Unclear risk
Pratley RE 2014d[3]	USA, Czech Republic, Hungary, Israel, Lithuania, Mexico, Poland, Romania, Russia, Slovakia, South Africa, Ukraine	Diet, exercise	26	Alogliptin	Placebo	113	109	63	55	53.7(9.7)	53.1(9.6)	4(4.8)	4.3(4.78)	NR	NR	25	Unclear risk	Unclear risk	Low risk	Low risk	High risk	Low risk	Unclear risk
Seino Y 2011b[114]	Japan	Diet, exercise	12	Alogliptin	Placebo	84	75	25	19	58.7(9.54)	59.1(10.47)	5.94(5.32)	6.83(6.07)	7.99(0.88)	7.85(0.89)	12.5	Low risk	Unclear risk	Low risk	Low risk	Low risk	Low risk	Unclear risk
Seino Y 2011c[114]	Japan	Diet, exercise	12	Alogliptin	Placebo	80	75	17	19	59.5(11.16)	59.1(10.47)	6.98(6.99)	6.83(6.07)	7.88(0.99)	7.85(0.89)	25	Low risk	Unclear risk	Low risk	Low risk	Low risk	Low risk	Unclear risk
DeFronzo RA 2008a[156]	USA, Argentina, Australia, Brazil, Chile, Czech Republic, Dominican Republic, Germany, Guatemala, Hungary, India, Mexico, Netherlands, New Zealand, Peru, Poland, South Africa, UK	Diet, exercise	26	Alogliptin	Placebo	133	64	68	32	53.4(11.1)	53.4(11.1)	NR	NR	7.9(0.08)	7.9(0.08)	12.5	Unclear risk	Unclear risk	Low risk	Low risk	Low risk	Low risk	Unclear risk
DeFronzo RA 2008b[156]	USA, Argentina, Australia, Brazil, Chile, Czech Republic, Dominican Republic, Germany, Guatemala, Hungary, India, Mexico, Netherlands, New Zealand, Peru, Poland, South Africa, UK	Diet, exercise	26	Alogliptin	Placebo	131	64	54	32	53.4(11.1)	53.4(11.1)	NR	NR	7.9(0.08)	7.9(0.08)	25	Unclear risk	Unclear risk	Low risk	Low risk	Low risk	Low risk	Unclear risk
Kutoh E 2012[157]	Japan	Exercise	12	Alogliptin	Diet	25	26	5	8	47.9(13.4)	50.5(11.4)	NR	NR	10.51(1.78)	10.01(1.12)	12.5~25	Low risk	Unclear risk	High risk	Low risk	Low risk	Low risk	Unclear risk
Wang WY 2020a[158]	China	Diet, exercise	12	Dapagliflozin	Placebo	30	30	17	15	41.3(1.9)	40.3(2.8)	Newly diagnosed	Newly diagnosed	9.2(0.7)	8.7(0.9)	10	Unclear risk	Unclear risk	Low risk	Low risk	Low risk	Unclear risk	Unclear risk

Wang WY 2020b[158]	China	Diet, exercise	12	Dapagliflozin	Placebo	30	30	14	15	40.6(3.1)	40.3(2.8)	Newly diagnosed	Newly diagnosed	8.3(0.7)	8.7(0.9)	5	Unclear risk	Unclear risk	Low risk	Low risk	Low risk	Unclear risk	Unclear risk
Liao X 2016[159]	China	Diet, exercise	12	Dapagliflozin	Placebo	117	45	60	23	55(7)	54(6)	NR	NR	8.04(0.5)	8.16(0.48)	10	Low risk	Unclear risk	Low risk	Low risk	High risk	Low risk	Unclear risk
Ji L 2014a[160]	China, Korea, Taiwan, India	Diet, exercise	24	Dapagliflozin	Placebo	128	132	44	45	53(11.07)	49.9(10.87)	1.15(2.3)	1.3(2)	8.14(0.74)	8.35(0.95)	5	Low risk	Low risk	Low risk	Low risk	Low risk	Low risk	Unclear risk
Ji L 2014b[160]	China, Korea, Taiwan, India	Diet, exercise	24	Dapagliflozin	Placebo	133	132	47	45	51.2(9.89)	49.9(10.87)	1.67(2.8)	1.3(2)	8.28(0.95)	8.35(0.95)	10	Low risk	Low risk	Low risk	Low risk	Low risk	Low risk	Unclear risk
Kaku K 2014a[161]	Japan	Diet, exercise	24	Dapagliflozin	Placebo	86	87	50	35	58.6(10.4)	60.4(9.7)	4.59(5.56)	5.29(6.17)	7.5(0.72)	7.5(0.63)	5	Unclear risk	Unclear risk	Low risk	Low risk	Low risk	Low risk	Unclear risk
Kaku K 2014b[161]	Japan	Diet, exercise	24	Dapagliflozin	Placebo	88	87	53	35	57.5(9.3)	60.4(9.7)	4.93(4.52)	5.29(6.17)	7.46(0.61)	7.5(0.63)	10	Unclear risk	Unclear risk	Low risk	Low risk	Low risk	Low risk	Unclear risk
Liu LM 2014a[162]	China	Diet, exercise	24	Dapagliflozin	Placebo	8	8	3	3	18~79	18~79	NR	NR	8.26(0.68)	8.69(0.86)	5	Unclear risk	Unclear risk	Low risk	Low risk	Low risk	Unclear risk	Unclear risk
Liu LM 2014b[162]	China	Diet, exercise	24	Dapagliflozin	Placebo	9	8	3	3	18~79	18~79	NR	NR	9.12(0.83)	8.69(0.86)	10	Unclear risk	Unclear risk	Low risk	Low risk	Low risk	Unclear risk	Unclear risk
Kaku K 2013B-a[163]	Japan	Diet, exercise	12	Dapagliflozin	Placebo	58	54	11	11	58(9.5)	58.4(10)	5.34(4.51)	4.74(3.82)	8.05(0.66)	8.12(0.71)	5	Low risk	Low risk	Low risk	Low risk	Low risk	Low risk	Unclear risk
Kaku K 2013B-b[163]	Japan	Diet, exercise	12	Dapagliflozin	Placebo	52	54	13	11	56.5(11.5)	58.4(10)	4.73(4.73)	4.74(3.82)	8.18(0.69)	8.12(0.71)	10	Low risk	Low risk	Low risk	Low risk	Low risk	Low risk	Unclear risk
Bailey CJ 2012[164]	USA, Canada, Mexico, Russia, India, South Africa	Diet, exercise	24	Dapagliflozin	Placebo	68	68	36	31	51.3(11.51)	53.5(11.08)	1.4(3.24)	1.1(1.95)	7.92(1.04)	7.8(1.12)	5	Low risk	Low risk	Low risk	Low risk	High risk	Low risk	Unclear risk
Ferrannini E 2010a[165]	USA, Canada, Mexico, Russia	Diet, exercise	24	Dapagliflozin	Placebo	64	75	33	44	52.6(10.9)	52.7(10.3)	0.25	0.50	7.86(0.94)	7.84(0.87)	5	Unclear risk	Unclear risk	Low risk	Low risk	Low risk	Low risk	Unclear risk
Ferrannini E 2010b[165]	USA, Canada, Mexico, Russia	Diet, exercise	24	Dapagliflozin	Placebo	70	75	36	44	50.6(9.97)	52.7(10.3)	0.45	0.50	8.01(0.96)	7.84(0.87)	10	Unclear risk	Unclear risk	Low risk	Low risk	Low risk	Low risk	Unclear risk
List JF 2009b[7]	USA, Canada, Mexico	Diet, exercise	12	Dapagliflozin	Placebo	55	44	29	19	55(12)	53(11)	NR	NR	8(0.9)	7.9(0.9)	5	Unclear risk	Unclear risk	Low risk	Low risk	High risk	Low risk	Unclear risk
List JF 2009c[7]	USA, Canada, Mexico	Diet, exercise	12	Dapagliflozin	Placebo	40	44	19	19	54(9)	53(11)	NR	NR	8(0.8)	7.9(0.9)	10	Unclear risk	Unclear risk	Low risk	Low risk	High risk	Low risk	Unclear risk
Roden M 2015b[119]	USA, Belgium, Canada, China, France, Germany, Greece, India, Ireland, Japan, Korea, Mexico, Philippines, Slovakia, Slovenia, Switzerland, Taiwan, Turkey, Ukraine	Diet, exercise	76	Empagliflozin	Placebo	224	228	82	105	56.2(11.6)	54.9(10.9)	NR	NR	7.87(0.88)	7.91(0.78)	10	Low risk	Low risk	Low risk	Low risk	Low risk	Low risk	Unclear risk
Roden M 2015c[119]	USA, Belgium, Canada, China, France, Germany, Greece, India, Ireland, Japan, Korea, Mexico, Philippines, Slovakia, Slovenia, Switzerland, Taiwan, Turkey, Ukraine	Diet, exercise	76	Empagliflozin	Placebo	224	228	79	105	53.8(11.6)	54.9(10.9)	NR	NR	7.86(0.85)	7.91(0.78)	25	Low risk	Low risk	Low risk	Low risk	Low risk	Low risk	Unclear risk
Kadowaki T 2014a[166]	Japan	Diet, exercise	12	Empagliflozin	Placebo	109	109	32	29	57.9(9.4)	58.7(8.7)	NR	NR	7.93(0.71)	7.94(0.74)	10	Low risk	Low risk	Low risk	Low risk	Low risk	Low risk	Unclear risk

Kadowaki T 2014b[166]	Japan	Diet, exercise	12	Empagliflozin	Placebo	109	109	25	29	57.2(9.7)	58.7(8.7)	NR	NR	7.93(0.78)	7.94(0.74)	25	Low risk	Low risk	Low risk	Low risk	Low risk	Low risk	Unclear risk
Ferrannini E 2013b[4]	Argentina, Croatia, Estonia, Germany, Italy, Korea, Lithuania, Romania, Russia, Sweden, Slovakia, Taiwan, Ukraine	None	12	Empagliflozin	Placebo	81	82	41	37	58	58	NR	NR	8(0.8)	7.8(0.8)	10	Low risk	Low risk	Low risk	Low risk	Low risk	Low risk	Unclear risk
Ferrannini E 2013c[4]	Argentina, Croatia, Estonia, Germany, Italy, Korea, Lithuania, Romania, Russia, Sweden, Slovakia, Taiwan, Ukraine	None	12	Empagliflozin	Placebo	82	82	41	37	57	58	NR	NR	7.8(0.8)	7.8(0.8)	25	Low risk	Low risk	Low risk	Low risk	Low risk	Low risk	Unclear risk
Roden M 2013b[120]	Belgium, Canada, China, Germany, India, Ireland, Japan, Switzerland, USA	Diet, exercise	24	Empagliflozin	Placebo	224	228	82	105	56.2(11.6)	54.9(10.9)	NR	NR	7.87(0.88)	7.91(0.78)	10	Low risk	Low risk	Low risk	Low risk	Low risk	Low risk	Unclear risk
Roden M 2013c[120]	Belgium, Canada, China, Germany, India, Ireland, Japan, Switzerland, USA	Diet, exercise	24	Empagliflozin	Placebo	224	228	79	105	53.8(11.6)	54.9(10.9)	NR	NR	7.86(0.85)	7.91(0.78)	25	Low risk	Low risk	Low risk	Low risk	Low risk	Low risk	Unclear risk
Inagaki N 2014a[167]	Japan	Diet, exercise	24	Canagliflozin	Placebo	90	93	31	33	58.4(10.4)	58.2(11)	4.72(4.59)	5.63(5.76)	7.98(0.73)	8.04(0.7)	100	Unclear risk	Unclear risk	Low risk	Low risk	Low risk	Low risk	Unclear risk
Inagaki N 2014b[167]	Japan	Diet, exercise	24	Canagliflozin	Placebo	88	93	16	33	57.4(11.1)	58.2(11)	5.88(5.93)	5.63(5.76)	8.04(0.77)	8.04(0.7)	200	Unclear risk	Unclear risk	Low risk	Low risk	Low risk	Low risk	Unclear risk
Inagaki N 2013a[168]	Japan	Diet, exercise	12	Canagliflozin	Placebo	74	75	22	21	57.7(10.5)	57.7(11)	NR	NR	8.05(0.86)	7.99(0.77)	100	Low risk	Low risk	Low risk	Low risk	Low risk	Low risk	Unclear risk
Inagaki N 2013b[168]	Japan	Diet, exercise	12	Canagliflozin	Placebo	76	75	27	21	57(10.7)	57.7(11)	NR	NR	8.11(0.88)	7.99(0.77)	200	Low risk	Low risk	Low risk	Low risk	Low risk	Low risk	Unclear risk
Inagaki N 2013c[168]	Japan	Diet, exercise	12	Canagliflozin	Placebo	75	75	20	21	57.1(10.1)	57.7(11)	NR	NR	8.17(0.81)	7.99(0.77)	300	Low risk	Low risk	Low risk	Low risk	Low risk	Low risk	Unclear risk
Stenlof K 2013a[169]	USA, Austria, Colombia, Estonia, Guatemala, Iceland, India, Korea, Lithuania, Malaysia, Mexico, Philippines, Poland, Romania, South Africa, Spain, Sweden	Diet, exercise	26	Canagliflozin	Placebo	195	192	114	104	55.1(10.8)	55.7(10.9)	4.5(4.4)	4.2(4.1)	8.1(1)	8(1)	100	Unclear risk	Unclear risk	Low risk	Low risk	High risk	Low risk	Unclear risk
Stenlof K 2013b[169]	USA, Austria, Colombia, Estonia, Guatemala, Iceland, India, Korea, Lithuania, Malaysia, Mexico, Philippines, Poland, Romania, South Africa, Spain, Sweden	Diet, exercise	26	Canagliflozin	Placebo	197	192	108	104	55.3(10.2)	55.7(10.9)	4.3(4.7)	4.2(4.1)	8(1)	8(1)	300	Unclear risk	Unclear risk	Low risk	Low risk	High risk	Low risk	Unclear risk
Hong LH 2016[170]	China	Diet, exercise	12	Exenatide twice-daily	Placebo	11	11	2	3	44.73(10.97)	38.45(8.65)	NR	NR	9.82(2.6)	9.2(1.64)	Initial:0.01; 5w:0.02	High risk	Unclear risk	Low risk	Low risk	Low risk	Low risk	Unclear risk
Gastaldelli A 2014a[171]	USA, Romania, Russia, India	Diet, exercise	24	Exenatide twice-daily	Placebo	26	24	16	12	57(2)	54(2)	NR	NR	7.78(0.92)	7.77(0.78)	0.01	Low risk	Low risk	Low risk	Low risk	Low risk	Low risk	Unclear risk
Gastaldelli A 2014b[171]	USA, Romania, Russia, India	Diet, exercise	24	Exenatide twice-daily	Placebo	29	24	18	12	59(2)	54(2)	NR	NR	7.73(0.92)	7.77(0.78)	0.02	Low risk	Low risk	Low risk	Low risk	Low risk	Low risk	Unclear risk
Moretto TJ 2008a[172]	USA, Romania, Russia, India	Diet, exercise	24	Exenatide twice-daily	Placebo	77	77	48	45	54(10)	53(9)	2(3)	1(2)	7.9(1)	7.8(0.9)	0.01	Low risk	Low risk	Low risk	Low risk	Low risk	Low risk	Unclear risk

Moretto TJ 2008b[172]	USA, Romania, Russia, India	Diet, exercise	24	Exenatide twice-daily	Placebo	78	77	38	45	55(10)	53(9)	2(3)	1(2)	7.8(1)	7.8(0.9)	Initial:0.01; 5w:0.02	Low risk	Low risk	Low risk	Low risk	Low risk	Low risk	Unclear risk
Zhou RF 2017b[24]	China	None	12	Exenatide twice-daily	Diet, exercise	30	30	20	15	49(12.9)	47.7(13.1)	Newly diagnosed	Newly diagnosed	7.6(0.6)	7.6(0.6)	0.01	Unclear risk	Unclear risk	High risk	Low risk	Low risk	Low risk	Unclear risk
Zhu HQ 2017[173]	China	Diet, exercise	12	Exenatide twice-daily	Diet, exercise	9	11	3	4	50.8(3.8)	50.7(4.2)	Newly diagnosed	Newly diagnosed	7.28(0.43)	7.21(0.71)	Initial:0.01; Max:0.02	Unclear risk	Unclear risk	High risk	Low risk	High risk	Low risk	Unclear risk
Yamada Y 2020[174]	Japan	Diet, exercise	52	Liraglutide	Placebo	48	49	9	9	59(10)	59(9)	6.7(5.2)	8.4(6)	8.3(0.8)	8.3(1.1)	0.9	Low risk	Low risk	Low risk	Low risk	Low risk	Low risk	Unclear risk
Nino A 2018[175]	Japan	Diet, exercise	24	Liraglutide	Placebo	103	77	22	25	58.4(9.72)	57.3(11.27)	5.8(4.37)	6.7(5.38)	8.07(0.79)	8.16(0.88)	Initial:0.3; 2w:0.6;3w:0.9	Low risk	Low risk	Low risk	Low risk	Low risk	Low risk	Unclear risk
Tran S 2018[176]	Canada	None	24	Liraglutide	Placebo	26	25	10	9	58.9(8.7)	57.4(7.4)	3.00	1.50	6.4(0.5)	6.2(0.4)	Initial:0.6; 2w:1.2;3w:1.8	Low risk	Unclear risk	Low risk	Low risk	Low risk	Low risk	Unclear risk
Miyagawa J 2015[177]	Japan	None	26	Liraglutide	Placebo	137	70	24	15	57.9(10.4)	57.7(8.3)	6.3(6)	6.3(5.1)	8.08(0.89)	8.2(0.83)	Initial:0.3; 2w:0.6;3w:0.9	Low risk	Low risk	High risk	Low risk	Low risk	Low risk	Unclear risk
Tan XR 2015[178]	China	Diet, exercise	12	Liraglutide	Placebo	45	30	20	15	56.1(9.1)	55.6(10.2)	Newly diagnosed	Newly diagnosed	8.72(0.62)	8.16(0.48)	Initial:0.6; 2w:1.2;3w:1.8	Unclear risk	Unclear risk	Low risk	Low risk	Low risk	Low risk	Unclear risk
Retnakaran R 2014[179]	Canada	None	48	Liraglutide	Placebo	26	25	10	9	58.9(8.7)	57.4(7.4)	3.00	1.50	6.4(0.5)	6.2(0.4)	Initial: 0.6; 2w:1.2;3w:1.8	Low risk	Unclear risk	Low risk	Low risk	Low risk	Low risk	Unclear risk
Seino Y 2008a[180]	Japan	Diet	14	Liraglutide	Placebo	45	46	17	17	60(7)	57.5(8.7)	8.87(6.77)	7.48(5.65)	8.21(0.83)	8.43(1.02)	0.6	Low risk	Low risk	Low risk	Low risk	High risk	Low risk	Unclear risk
Seino Y 2008b[180]	Japan	Diet	14	Liraglutide	Placebo	44	46	13	17	55.5(7.6)	57.5(8.7)	7.62(4.92)	7.48(5.65)	8.12(0.98)	8.43(1.02)	0.9	Low risk	Low risk	Low risk	Low risk	High risk	Low risk	Unclear risk
Vilsboll T 2008a[181]	Denmark	None	14	Liraglutide	Placebo	8	10	0	2	61.1(7.6)	55.4(6.7)	7.1(2.5)	1.8(0.8)	8.7(0.7)	8.1(0.3)	0.65	Unclear risk	Unclear risk	Low risk	Low risk	High risk	Low risk	Unclear risk
Vilsboll T 2008b[181]	Denmark	None	14	Liraglutide	Placebo	10	10	0	2	56.9(10.1)	55.4(6.7)	7.9(2.7)	1.8(0.8)	8.4(0.6)	8.1(0.3)	1.25	Unclear risk	Unclear risk	Low risk	Low risk	High risk	Low risk	Unclear risk
Vilsboll T 2008c[181]	Denmark	None	14	Liraglutide	Placebo	11	10	2	2	58.6(10.3)	55.4(6.7)	4.6(3.2)	1.8(0.8)	8.2(0.6)	8.1(0.3)	1.9	Unclear risk	Unclear risk	Low risk	Low risk	High risk	Low risk	Unclear risk
Fan P 2020[182]	China	Diet	12	Liraglutide	Diet	52	52	23	24	57.85(5.11)	58.47(5.48)	5.32(2.1)	5.74(2.21)	9.01(1.21)	9.27(1.32)	Initial:0.6; Max:1.2	Unclear risk	Unclear risk	High risk	Low risk	Low risk	Unclear risk	Unclear risk
Li QY 2018[183]	China	Diet, exercise	24	Liraglutide	Diet, exercise	30	30	NR	NR	30.8(1.9)	31.1(2.1)	Newly diagnosed	Newly diagnosed	5.6(0.9)	5.9(1.1)	Initial:0.6; Max:1.8	Low risk	Unclear risk	High risk	Low risk	Low risk	Low risk	Unclear risk
Liu MR 2017a[184]	China	Diet	12	Liraglutide	Diet	30	30	14	15	39.68(11.48)	40.05(10.26)	0.7(0.17)	0.68(0.18)	7.16(0.7)	7.19(0.65)	Initial:0.6; 2w:1.2	Low risk	Unclear risk	High risk	Low risk	Low risk	Low risk	Unclear risk
Liu MR 2017b[184]	China	Diet	12	Liraglutide	Diet	30	30	14	18	39.86(9.98)	40.62(11.03)	0.68(0.25)	0.69(0.17)	7.03(0.64)	6.96(0.63)	Initial:0.6; 2w:1.2	Low risk	Unclear risk	High risk	Low risk	Low risk	Low risk	Unclear risk
Li L 2014b[29]	China	Diet, exercise	12	Liraglutide	Diet, exercise	16	12	6	4	49.5(11.7)	49.5(11.7)	1.2(0.9)	1.2(0.9)	8.12(1.36)	8.01(1.07)	Initial:0.6; 3w:1.2/1.8	Unclear risk	Unclear risk	High risk	Low risk	Low risk	Unclear risk	Unclear risk
Xi Y 2013b[32]	China	Diet, exercise	16	Liraglutide	Diet, exercise	20	20	8	9	50(5)	51(7)	Newly diagnosed	Newly diagnosed	8.8(0.8)	8.3(0.9)	Initial:0.6; 2w:1.2	Low risk	Unclear risk	High risk	Low risk	Low risk	Unclear risk	Unclear risk

Fonseca VA 2012a[185]	Belgium, India, Israel, Japan, South Korea, Mexico, Poland, Romania, Russia, Tunisia, Ukraine, USA	Diet, exercise	12	Lixisenatide	Placebo	120	122	57	62	53.3(9.7)	54.1(11)	1.40	1.40	7.98(0.9)	8.07(0.9)	Initial:0.01; 2w:0.015;3w:0.02	Low risk	Low risk	Low risk	Low risk	Low risk	Low risk	Unclear risk
Fonseca VA 2012b[185]	Belgium, India, Israel, Japan, South Korea, Mexico, Poland, Romania, Russia, Tunisia, Ukraine, USA	Diet, exercise	12	Lixisenatide	Placebo	119	122	56	62	53.8(10.9)	54.1(11)	1.10	1.40	8.07(0.9)	8.07(0.9)	Initial:0.01; 3w:0.02	Low risk	Low risk	Low risk	Low risk	Low risk	Low risk	Unclear risk

C, comparator. I, intervention. NR, not reported. w, week.

Table S4. Treatment effects on vascular events of glucose-lowering drugs vs each other

**Myocardial infarction** (left lower half)							**Heart failure** (right upper half)
**Metformin**	—	1.01 [0.06, 16.16]	—	—	0.98 [0.05, 19.33]	0.98 [0.04, 23.80]	1.47 [0.04, 50.93]
0.70 [0.01, 33.72]	**SUs**	—	—	—	—	—	—
1.19 [0.07, 21.38]	1.71 [0.05, 65.41]	**TZDs**	—	—	0.97 [0.08, 12.33]	0.98 [0.06, 15.69]	1.46 [0.06, 35.18]
—	—	—	**NIDEs**	—	—	—	—
0.51 [0.02, 14.41]	0.73 [0.01, 40.51]	0.43 [0.02, 9.20]	—	**AGIs**	—	—	—
2.09 [0.19, 23.58]	3.00 [0.11, 80.41]	1.76 [0.23, 13.34]	—	4.09 [0.30, 56.85]	**DPP-4is**	1.00 [0.05, 19.83]	1.50 [0.05, 43.35]
1.25 [0.08, 19.00]	1.79 [0.05, 60.25]	1.05 [0.10, 11.31]	—	2.45 [0.13, 44.85]	0.60 [0.10, 3.55]	**SGLT2is**	1.49 [0.04, 51.96]
1.47 [0.04, 50.93]	2.11 [0.03, 138.64]	1.24 [0.05, 33.10]	—	2.88 [0.07, 115.41]	0.70 [0.04, 12.61]	1.18 [0.05, 27.22]	**GLP-1RAs**
**Stroke** (left lower half)							**Diabetic nephropathy** (right upper half)
**Metformin**	—	1.64 [0.02, 137.30]	—	—	1.62 [0.09, 28.34]	1.04 [0.10, 10.58]	1.98 [0.06, 62.19]
6.26 [0.13, 303.48]	**SUs**	—	—	—	—	—	—
1.02 [0.05, 20.84]	0.16 [0.00, 6.90]	**TZDs**	—	—	0.99 [0.01, 78.19]	0.64 [0.01, 35.96]	1.21 [0.01, 143.15]
—	—	—	**NIDEs**	—	—	—	—
—	—	—	—	**AGIs**	—	—	—
0.99 [0.05, 19.48]	0.16 [0.00, 6.50]	0.97 [0.06, 16.08]	—	—	**DPP-4is**	0.64 [0.07, 5.80]	1.22 [0.04, 35.45]
1.68 [0.11, 26.49]	0.27 [0.01, 9.26]	1.65 [0.13, 21.54]	—	—	1.70 [0.14, 21.20]	**SGLT2is**	1.90 [0.10, 35.27]
1.47 [0.04, 50.93]	0.24 [0.00, 15.40]	1.44 [0.05, 43.29]	—	—	1.49 [0.05, 43.07]	0.88 [0.04, 20.87]	**GLP-1RAs**

Treatment estimates are RR [95% CIs] of the column-defining treatment compared with the row-defining treatment for myocardial infarction, stroke (left lower half), and RR > 1 favor the row-defining treatment. Treatment estimates are RR [95% CIs] of the row-defining treatment compared with the column-defining treatment for heart failure, diabetic nephropathy (right upper half), and RR > 1 favor the column-defining treatment. * Statistically significant differences. **Abbreviations**: AGIs, α-glucosidase inhibitors. CI, confidence interval. DPP-4is, dipeptidyl peptidase-4 inhibitors. GLP-1RAs, glucagon-like peptide-1 receptor agonists. NIDEs, glinides. RR, risk ratio. SGLT2is, sodium-glucose cotransporter 2 inhibitors. SUs, sulfonylureas. TZDs, thiazolidinediones.

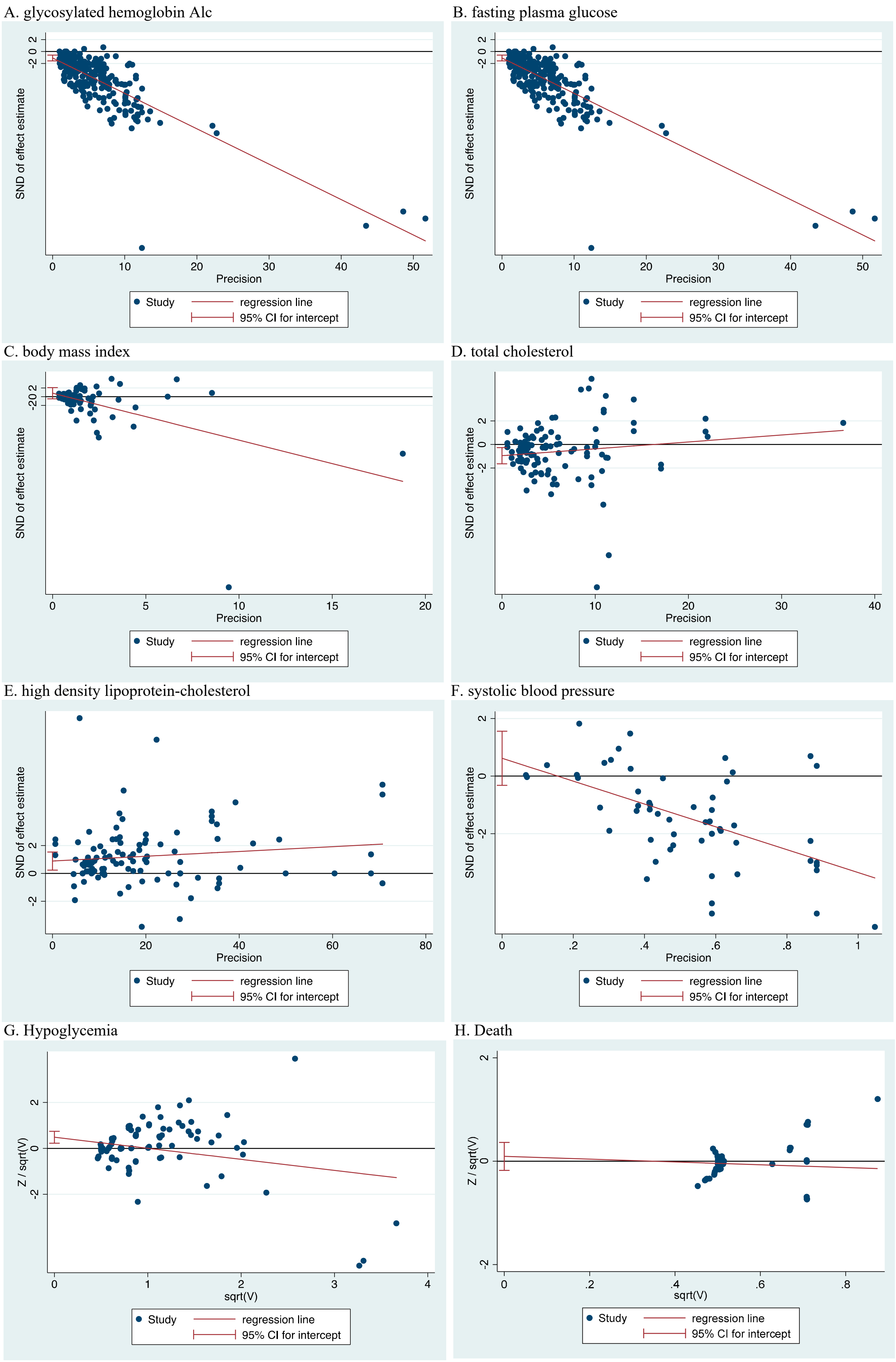

Figure S1. Risk of publication bias across studies

【Hemoglobin Alc, %】

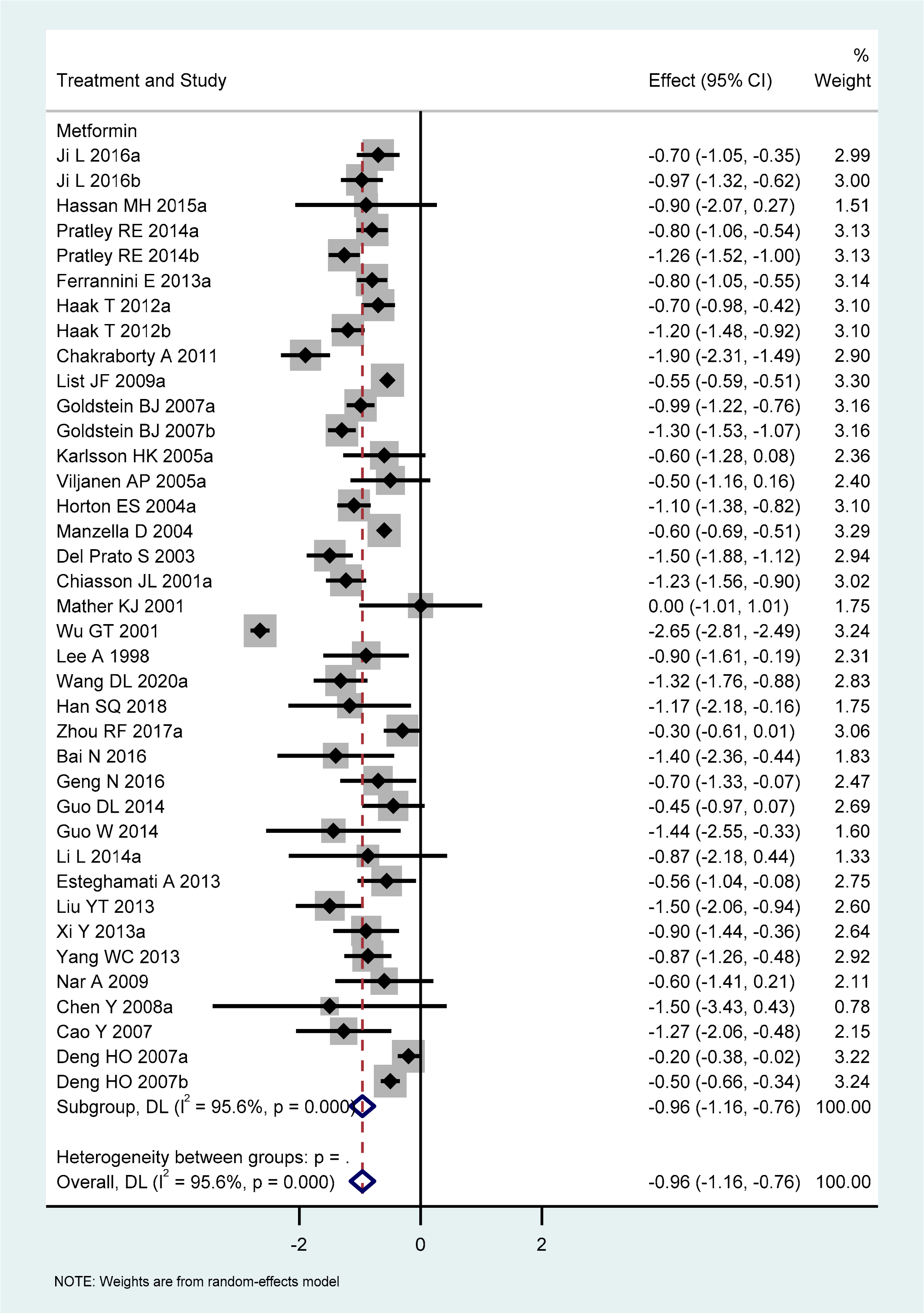

Figure S2. Meta-analysis results for change in hemoglobin Alc (%) of metformin vs placebo/lifestyle intervention

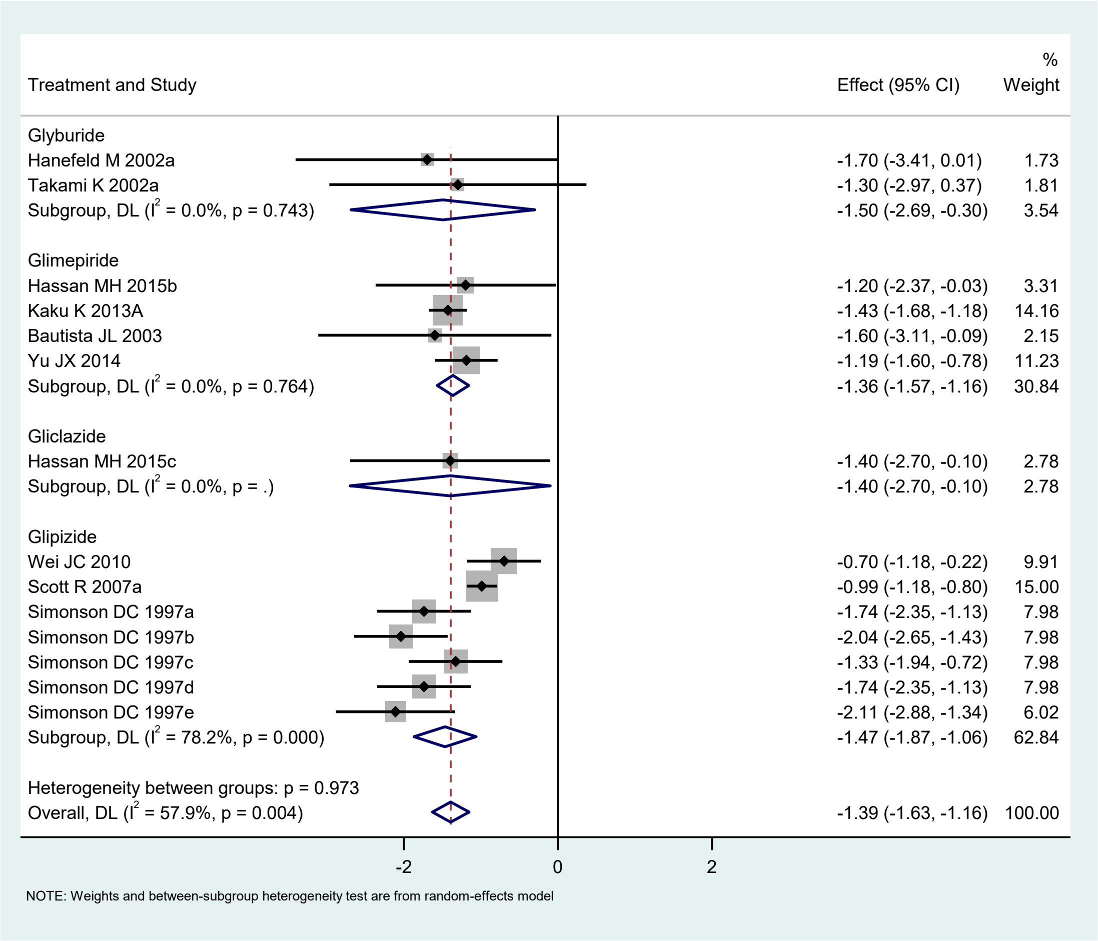

Figure S3. Meta-analysis results for change in hemoglobin Alc (%) of sulfonylureas vs placebo/lifestyle intervention

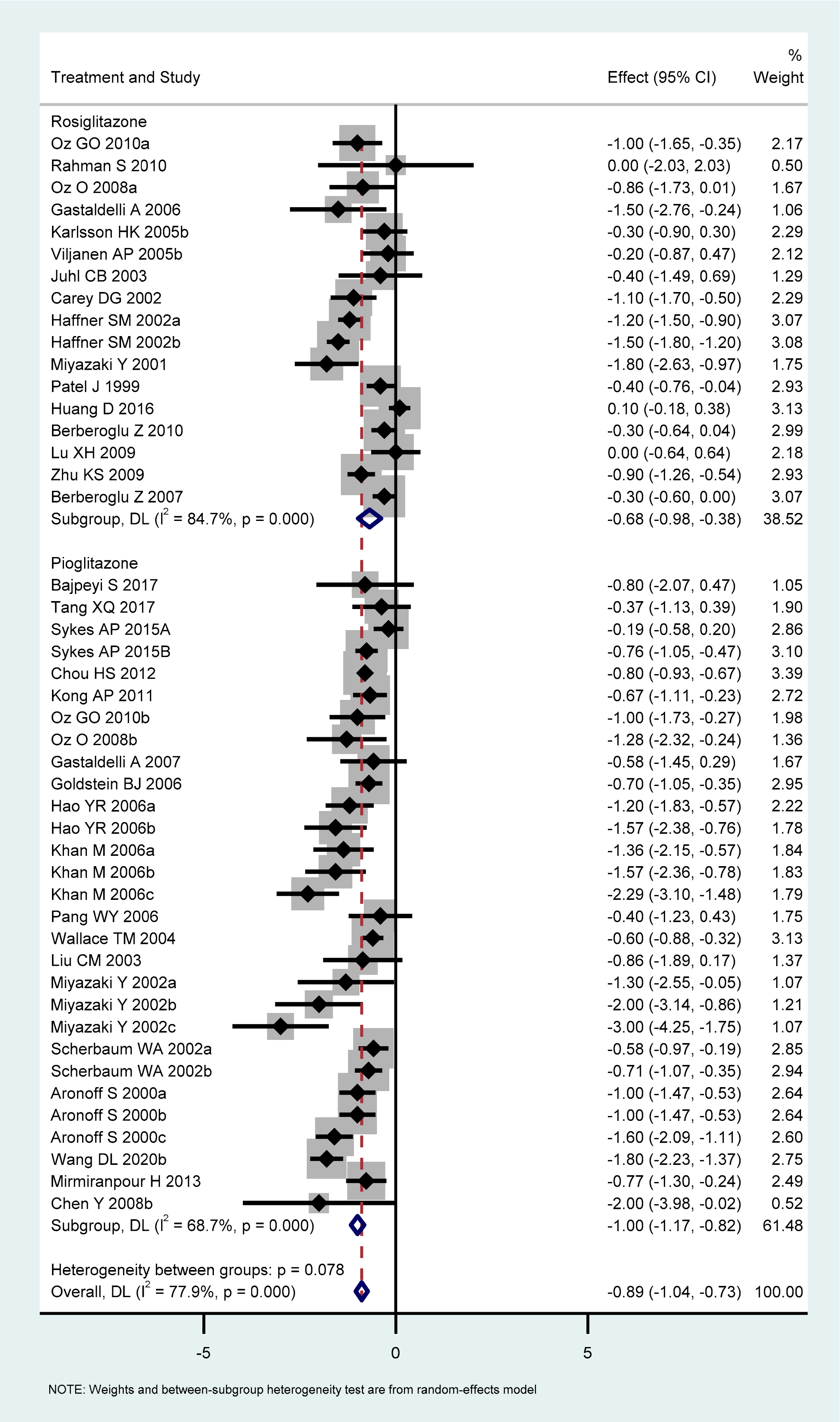

Figure S4. Meta-analysis results for change in hemoglobin Alc (%) of thiazolidinediones vs placebo/lifestyle intervention

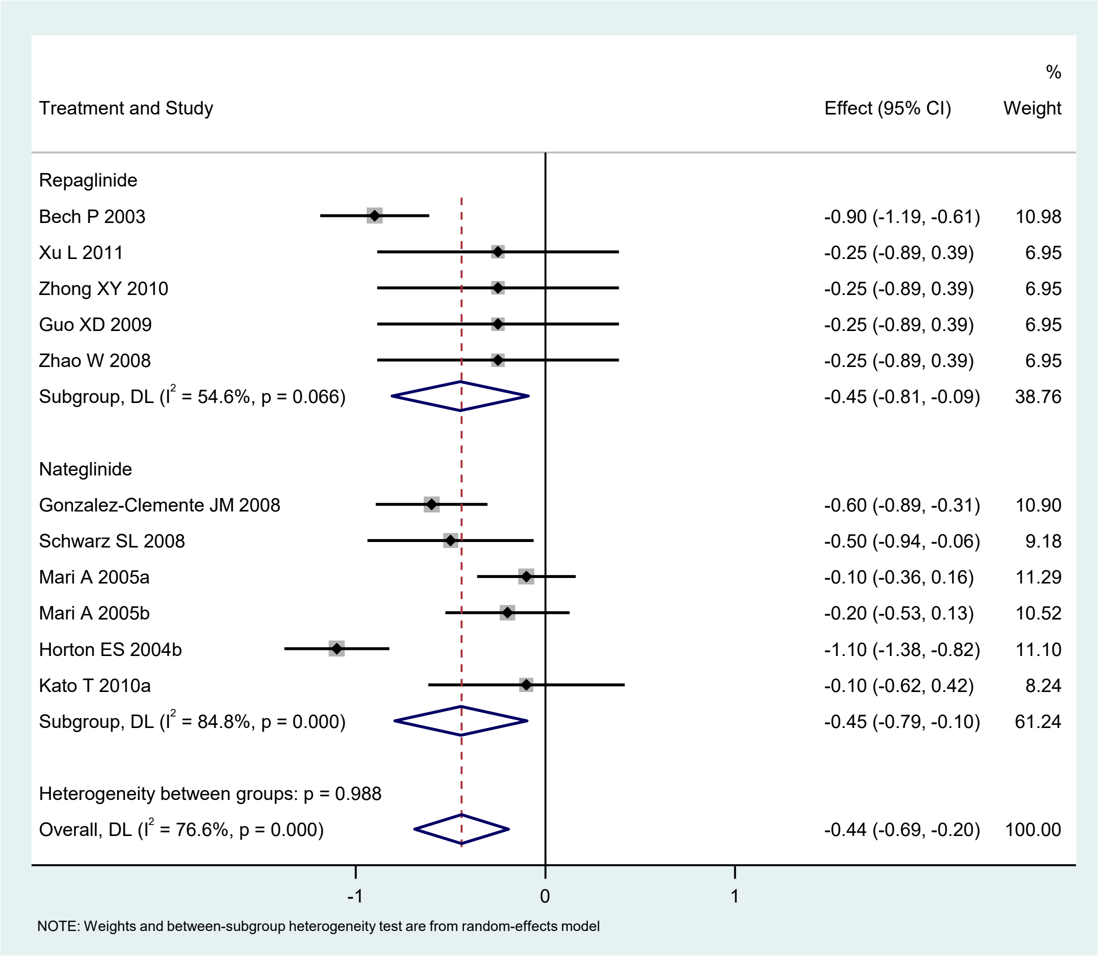

Figure S5. Meta-analysis results for change in hemoglobin Alc (%) of glinides vs placebo/lifestyle intervention

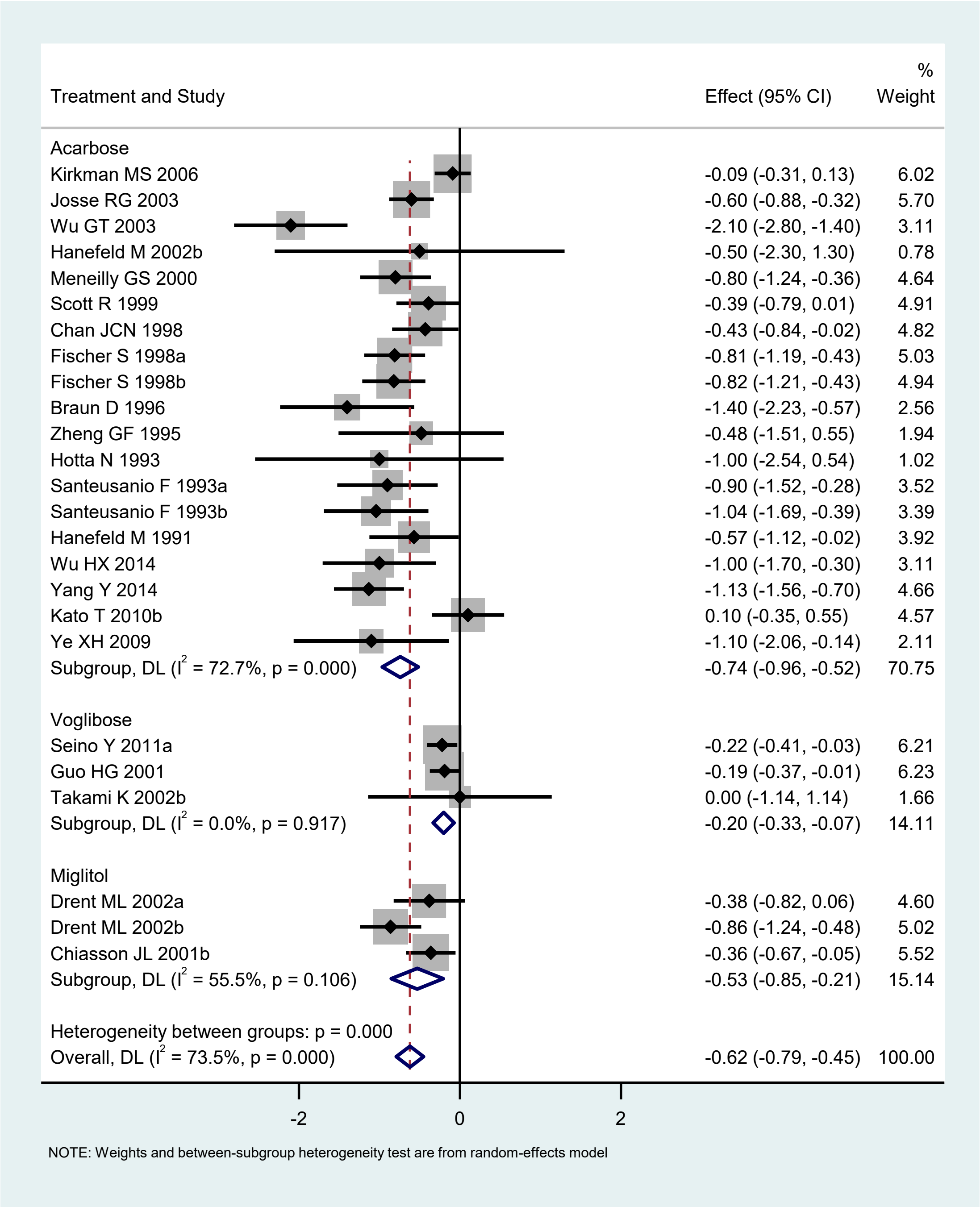

Figure S6. Meta-analysis results for change in hemoglobin Alc (%) of α-glucosidase inhibitors vs placebo/lifestyle intervention

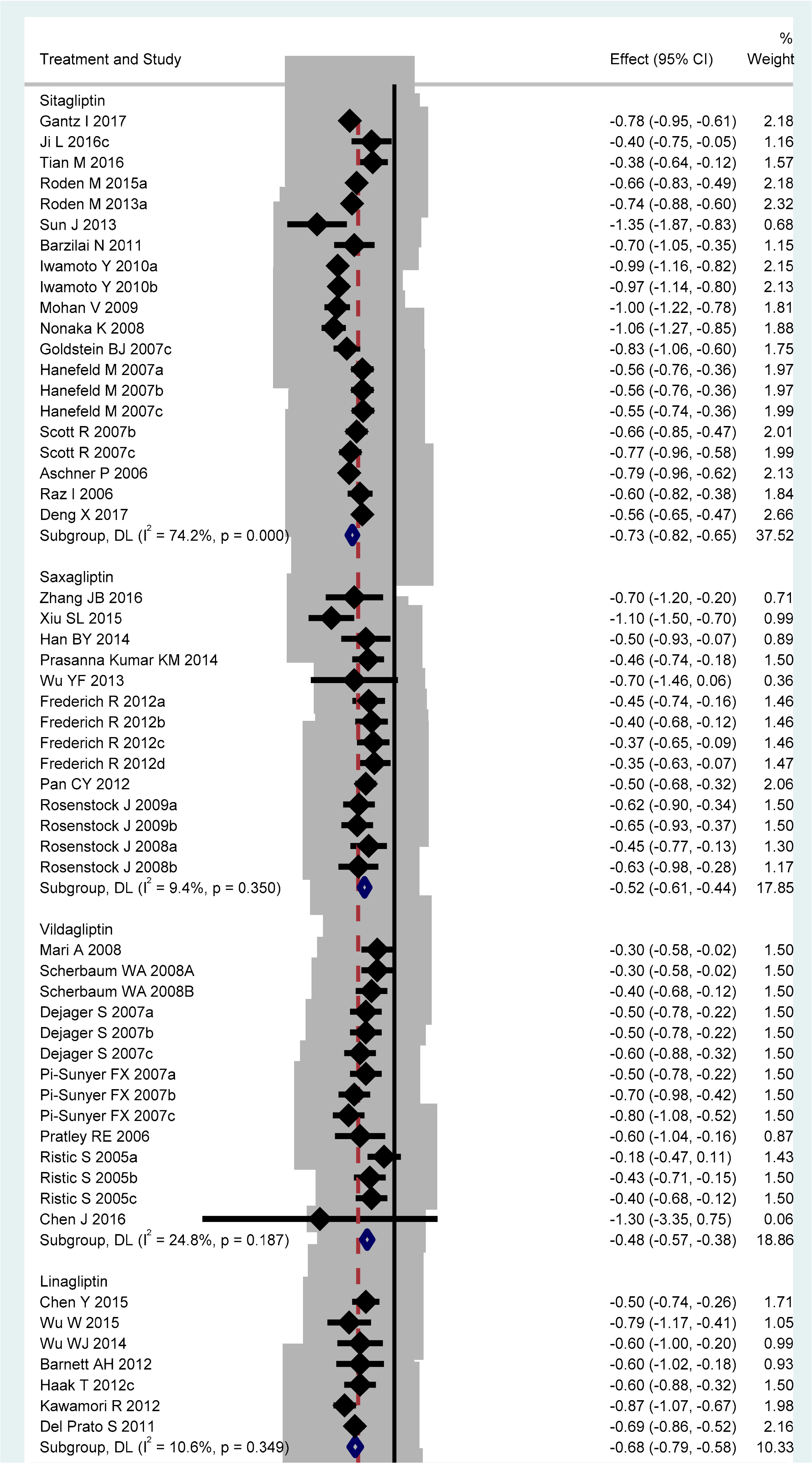
Treatment and Study
Effect (95% CI)
% Weight
Sitagliptin
Gantz I 2017 -0.78 (-0.95, -0.61) 2.18
Ji L 2016c -0.40 (-0.75, -0.05) 1.16
Tian M 2016 -0.38 (-0.64, -0.12) 1.57
Roden M 2015a -0.66 (-0.83, -0.49) 2.18
Roden M 2013a -0.74 (-0.88, -0.60) 2.32
Sun J 2013 -1.35 (-1.87, -0.83) 0.68
Barzilai N 2011 -0.70 (-1.05, -0.35) 1.15
Iwamoto Y 2010a -0.99 (-1.16, -0.82) 2.15
Iwamoto Y 2010b -0.97 (-1.14, -0.80) 2.13
Mohan V 2009 -1.00 (-1.22, -0.78) 1.81
Nonaka K 2008 -1.06 (-1.27, -0.85) 1.88
Goldstein BJ 2007c -0.83 (-1.06, -0.60) 1.75
Hanefeld M 2007a -0.56 (-0.76, -0.36) 1.97
Hanefeld M 2007b -0.56 (-0.76, -0.36) 1.97
Hanefeld M 2007c -0.55 (-0.74, -0.36) 1.99
Scott R 2007b -0.66 (-0.85, -0.47) 2.01
Scott R 2007c -0.77 (-0.96, -0.58) 1.99
Aschner P 2006 -0.79 (-0.96, -0.62) 2.13
Raz I 2006 -0.60 (-0.82, -0.38) 1.84
Deng X 2017 -0.56 (-0.65, -0.47) 2.66
Subgroup, DL (I2 = 74.2%, p = 0.000) -0.73 (-0.82, -0.65) 37.52
Saxagliptin
Zhang JB 2016 -0.70 (-1.20, -0.20) 0.71
Xiu SL 2015 -1.10 (-1.50, -0.70) 0.99
Han BY 2014 -0.50 (-0.93, -0.07) 0.89
Prasanna Kumar KM 2014 -0.46 (-0.74, -0.18) 1.50
Wu YF 2013 -0.70 (-1.46, 0.06) 0.36
Frederich R 2012a -0.45 (-0.74, -0.16) 1.46
Frederich R 2012b -0.40 (-0.68, -0.12) 1.46
Frederich R 2012c -0.37 (-0.65, -0.09) 1.46
Frederich R 2012d -0.35 (-0.63, -0.07) 1.47
Pan CY 2012 -0.50 (-0.68, -0.32) 2.06
Rosenstock J 2009a -0.62 (-0.90, -0.34) 1.50
Rosenstock J 2009b -0.65 (-0.93, -0.37) 1.50
Rosenstock J 2008a -0.45 (-0.77, -0.13) 1.30
Rosenstock J 2008b -0.63 (-0.98, -0.28) 1.17
Subgroup, DL (I2 = 9.4%, p = 0.350) -0.52 (-0.61, -0.44) 17.85
Vildagliptin
Mari A 2008 -0.30 (-0.58, -0.02) 1.50
Scherbaum WA 2008A -0.30 (-0.58, -0.02) 1.50
Scherbaum WA 2008B -0.40 (-0.68, -0.12) 1.50
Dejager S 2007a -0.50 (-0.78, -0.22) 1.50
Dejager S 2007b -0.50 (-0.78, -0.22) 1.50
Dejager S 2007c -0.60 (-0.88, -0.32) 1.50
Pi-Sunyer FX 2007a -0.50 (-0.78, -0.22) 1.50
Pi-Sunyer FX 2007b -0.70 (-0.98, -0.42) 1.50
Pi-Sunyer FX 2007c -0.80 (-1.08, -0.52) 1.50
Pratley RE 2006 -0.60 (-1.04, -0.16) 0.87
Ristic S 2005a -0.18 (-0.47, 0.11) 1.43
Ristic S 2005b -0.43 (-0.71, -0.15) 1.50
Ristic S 2005c -0.40 (-0.68, -0.12) 1.50
Chen J 2016 -1.30 (-3.35, 0.75) 0.06
Subgroup, DL (I2 = 24.8%, p = 0.187) -0.48 (-0.57, -0.38) 18.86
Linagliptin
Chen Y 2015 -0.50 (-0.74, -0.26) 1.71
Wu W 2015 -0.79 (-1.17, -0.41) 1.05
Wu WJ 2014 -0.60 (-1.00, -0.20) 0.99
Barnett AH 2012 -0.60 (-1.02, -0.18) 0.93
Haak T 2012c -0.60 (-0.88, -0.32) 1.50
Kawamori R 2012 -0.87 (-1.07, -0.67) 1.98
Del Prato S 2011 -0.69 (-0.86, -0.52) 2.16
Subgroup, DL (I2 = 10.6%, p = 0.349) -0.68 (-0.79, -0.58) 10.33

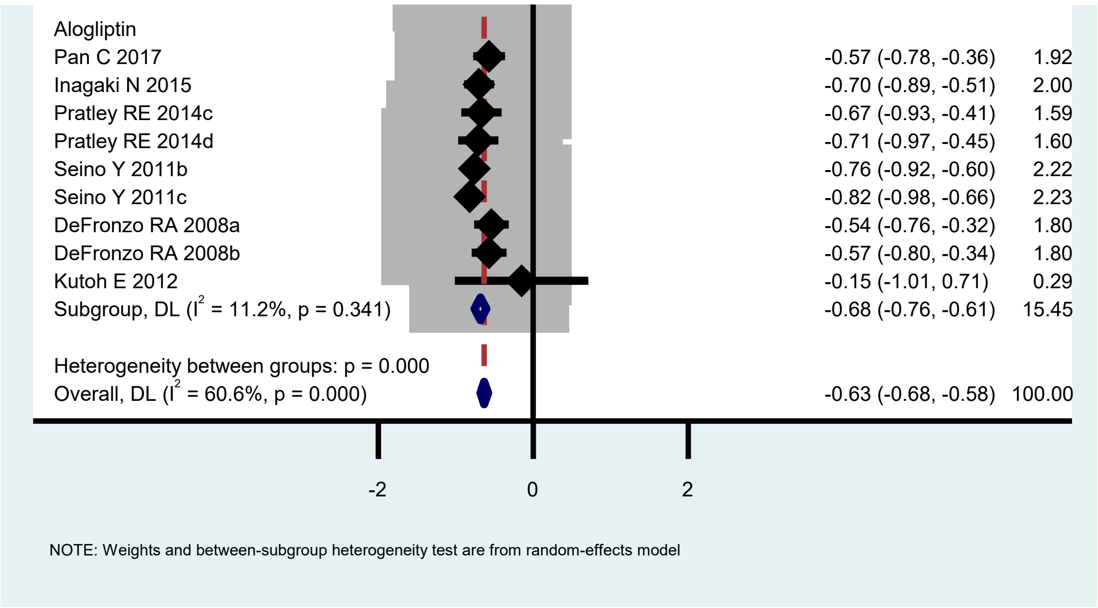

Figure S7. Meta-analysis results for change in hemoglobin Alc (%) of dipeptidyl peptidase-4 inhibitors vs placebo/lifestyle intervention

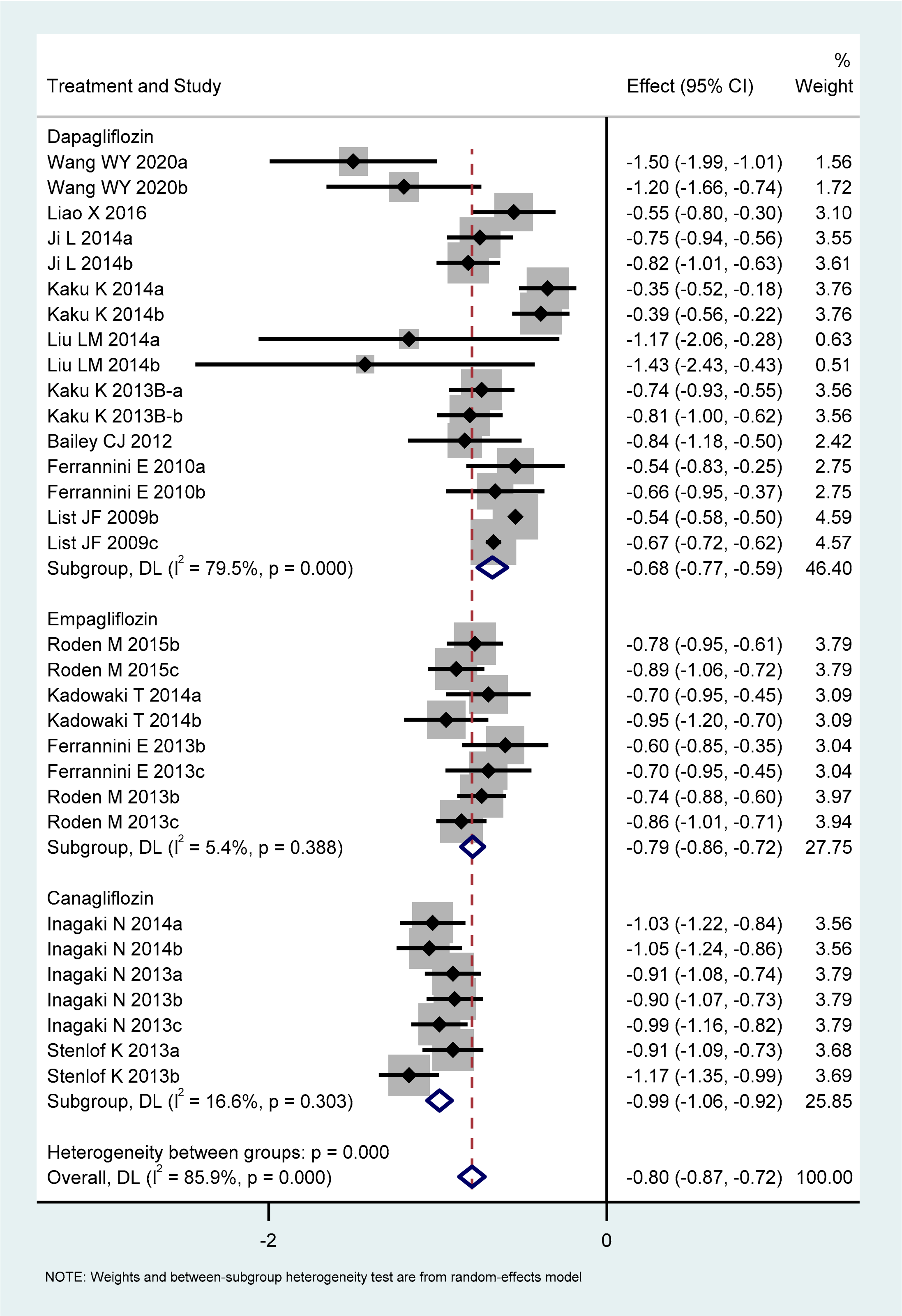

Figure S8. Meta-analysis results for change in hemoglobin Alc (%) of sodium-glucose cotransporter-2 inhibitors vs placebo/lifestyle intervention

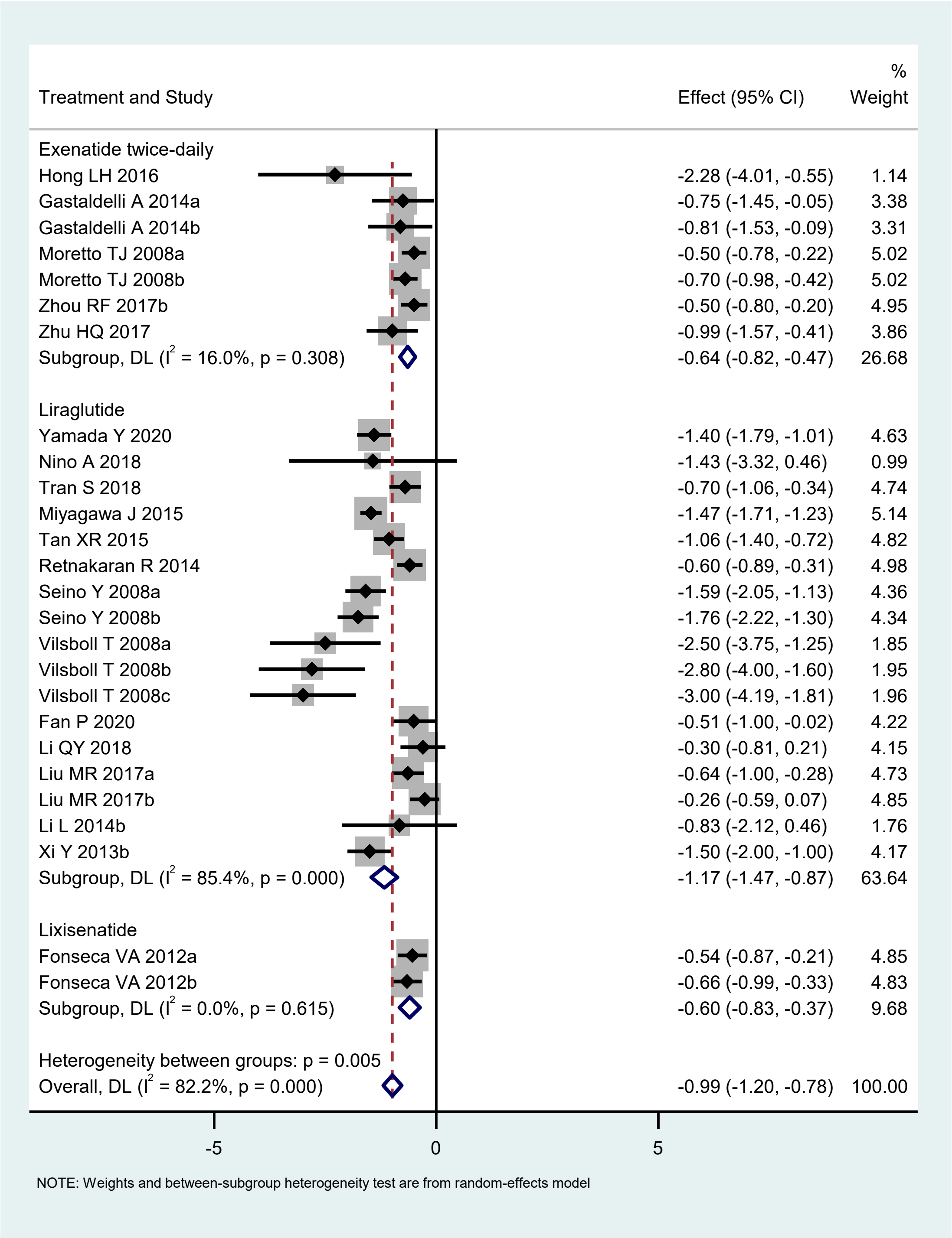

Figure S9. Meta-analysis results for change in hemoglobin Alc (%) of glucagon-like peptide-1 receptor agonists vs placebo/lifestyle intervention

【Fasting plasma glucose, mmol/l】

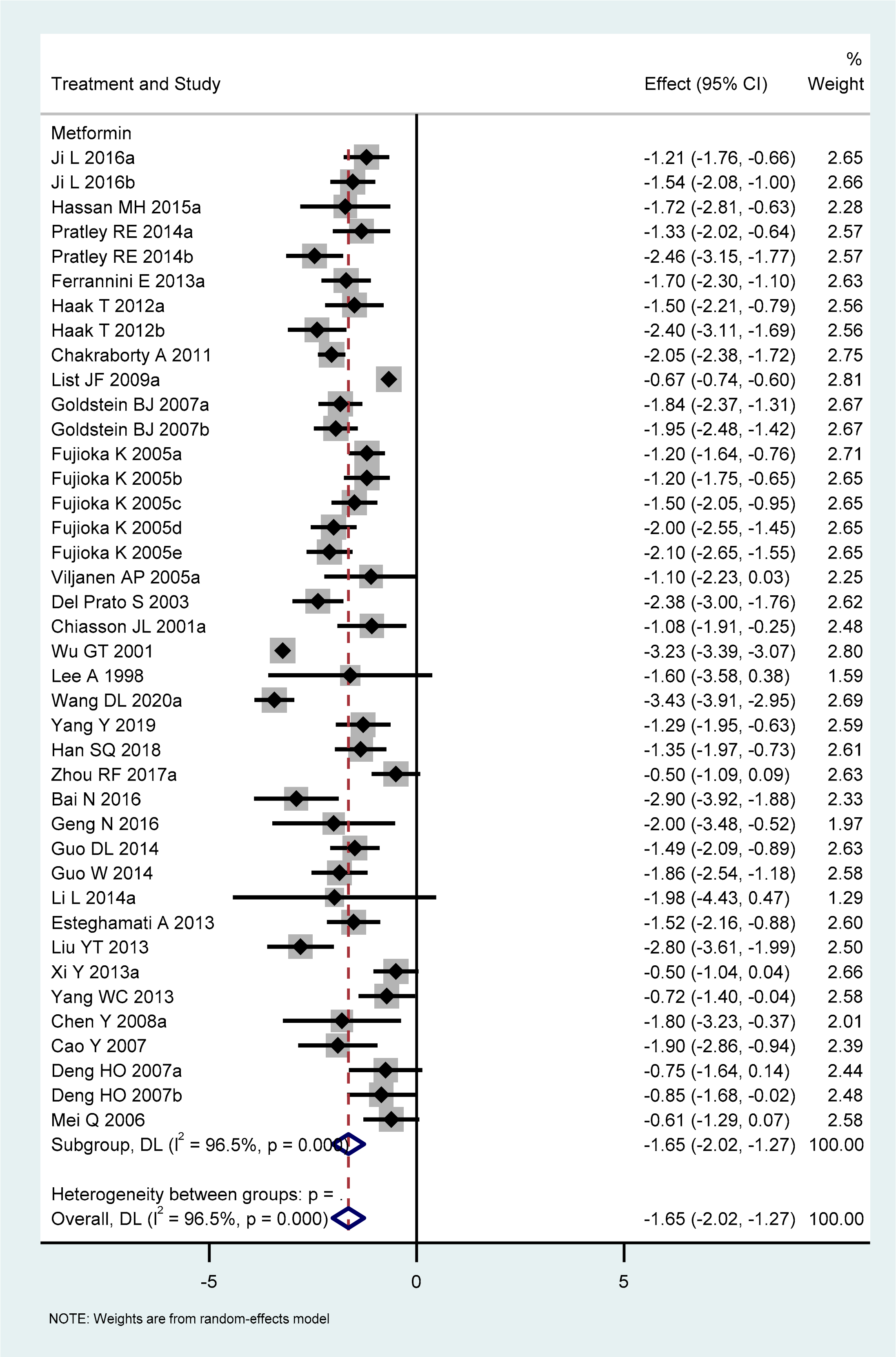

Figure S10. Meta-analysis results for change in fasting plasma glucose (mmol/l) of metformin vs placebo/lifestyle intervention

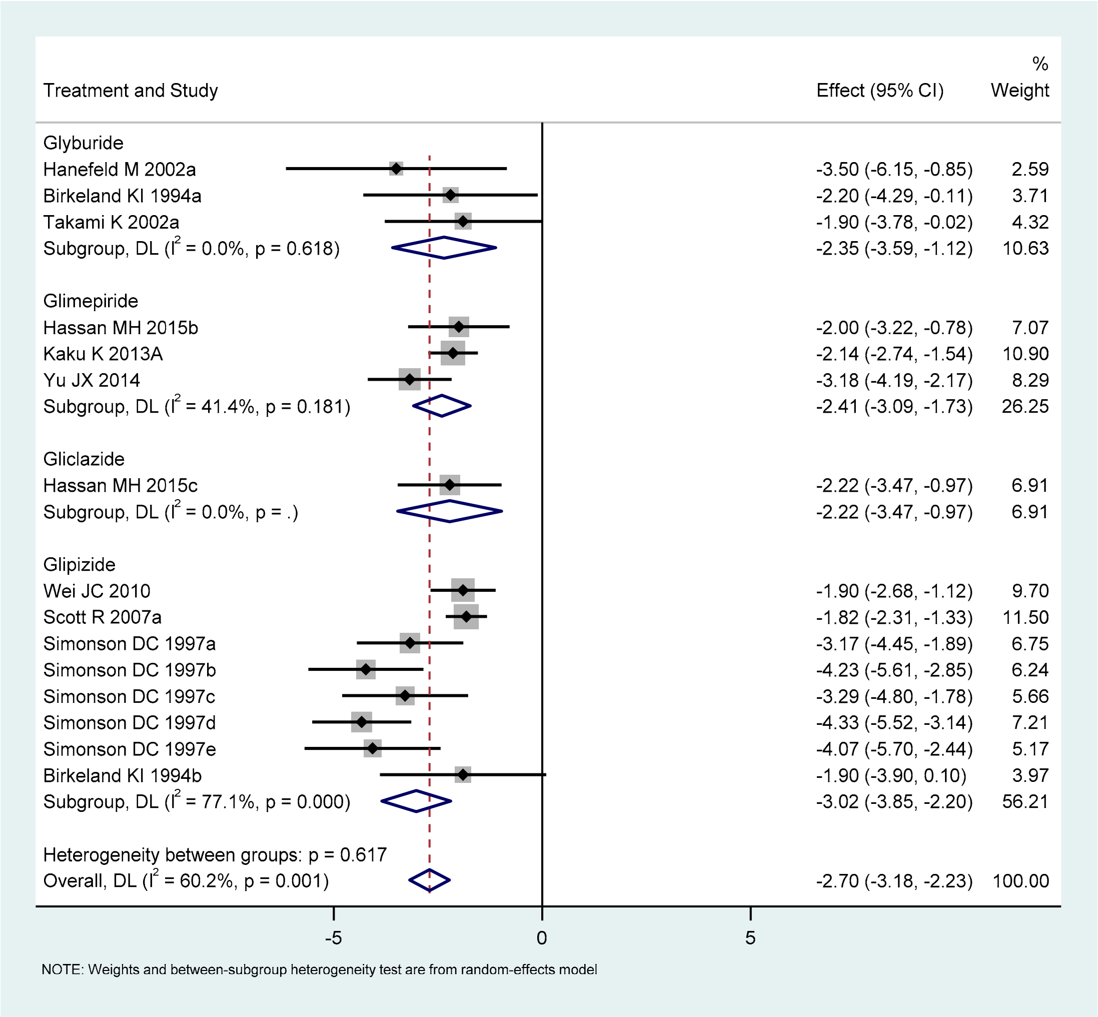

Figure S11. Meta-analysis results for change in fasting plasma glucose (mmol/l) of sulfonylureas vs placebo/lifestyle intervention

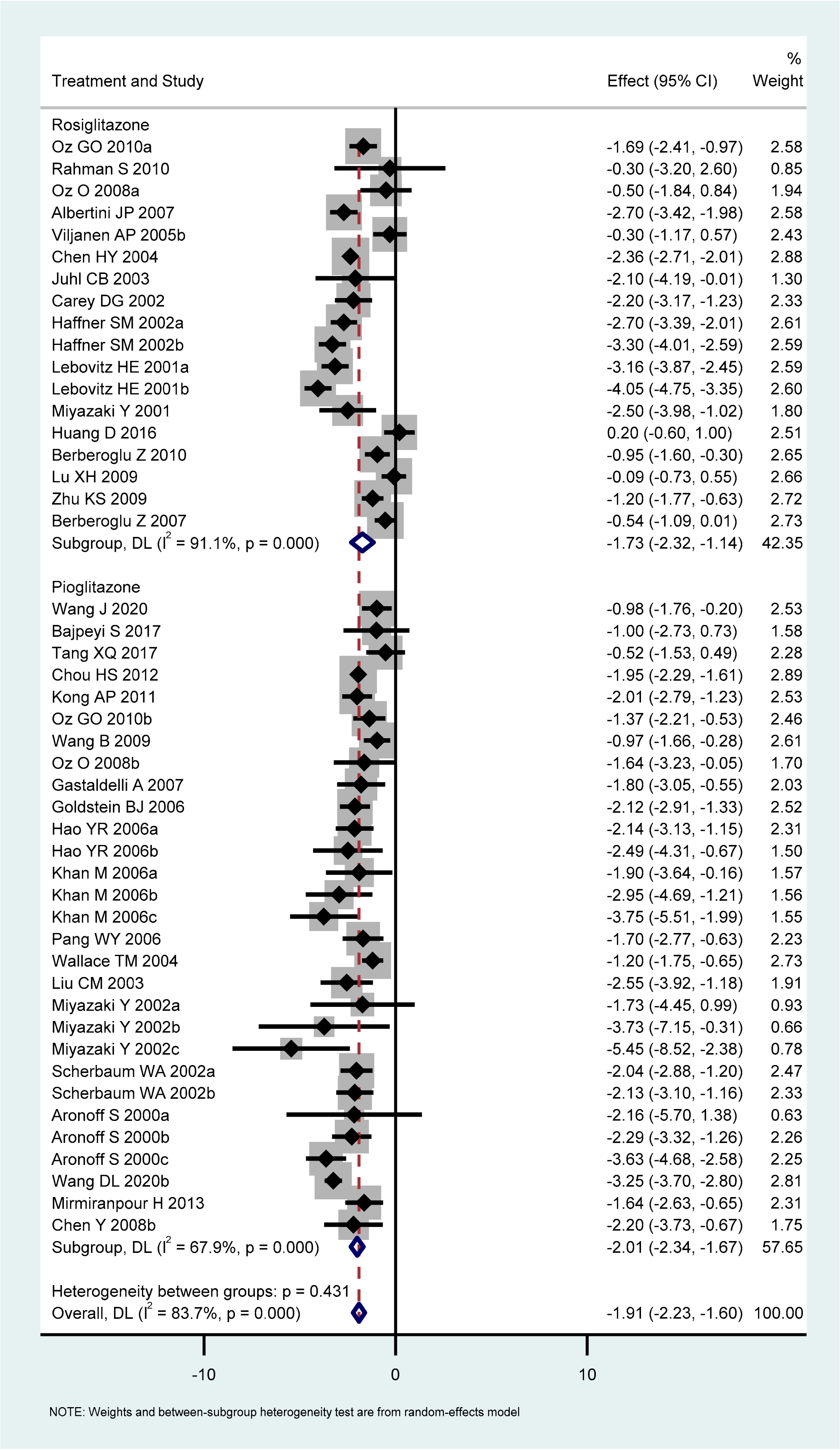

Figure S12. Meta-analysis results for change in fasting plasma glucose (mmol/l) of thiazolidinediones vs placebo/lifestyle intervention

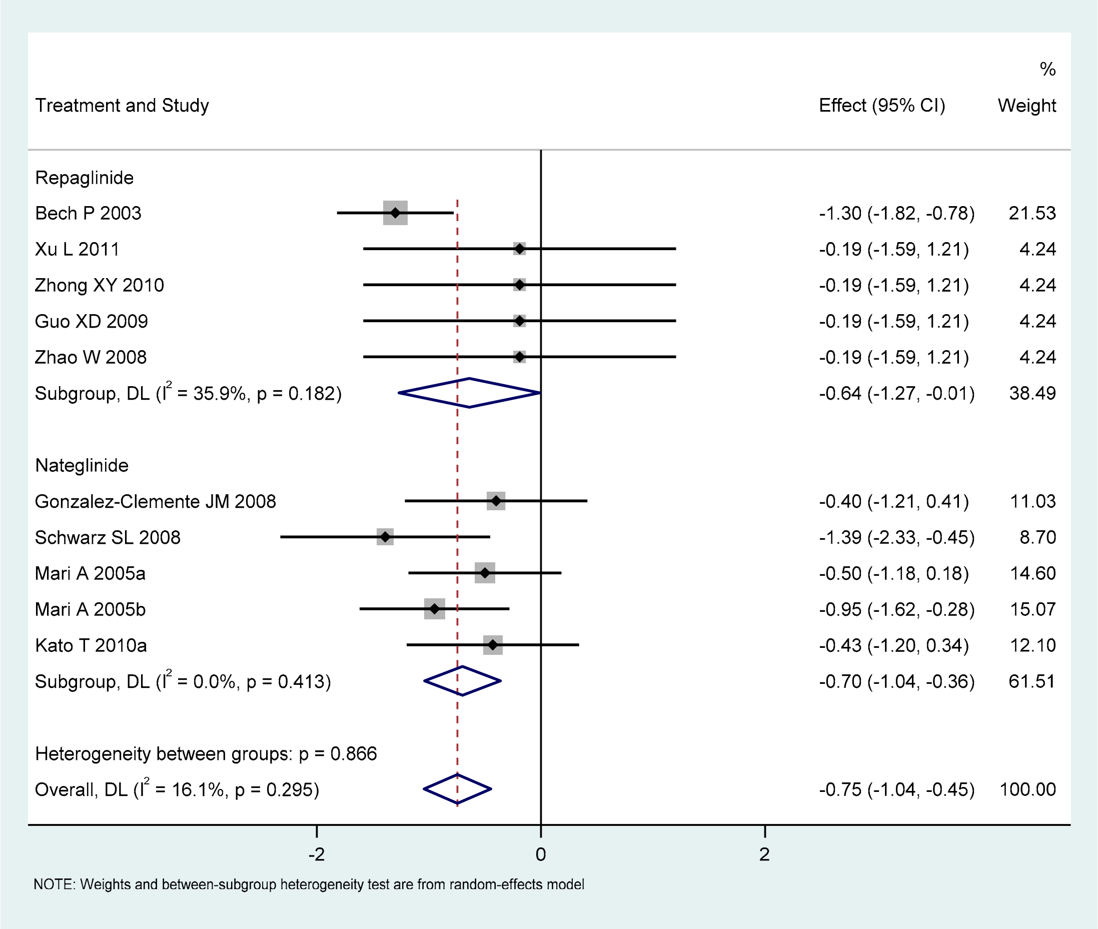

Figure S13. Meta-analysis results for change in fasting plasma glucose (mmol/l) of glinides vs placebo/lifestyle intervention

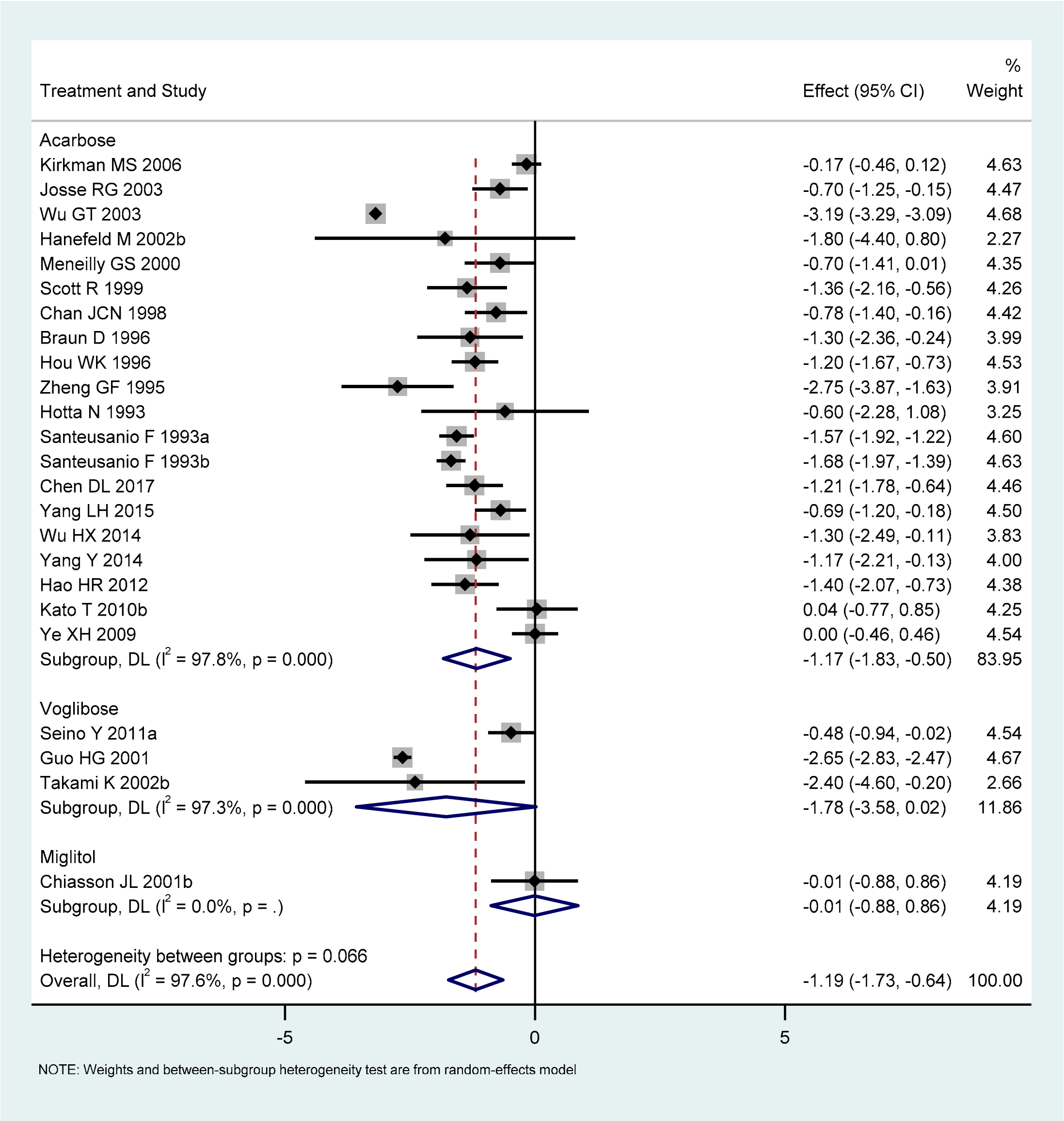

Figure S14. Meta-analysis results for change in fasting plasma glucose (mmol/l) of α-glucosidase inhibitors vs placebo/lifestyle intervention

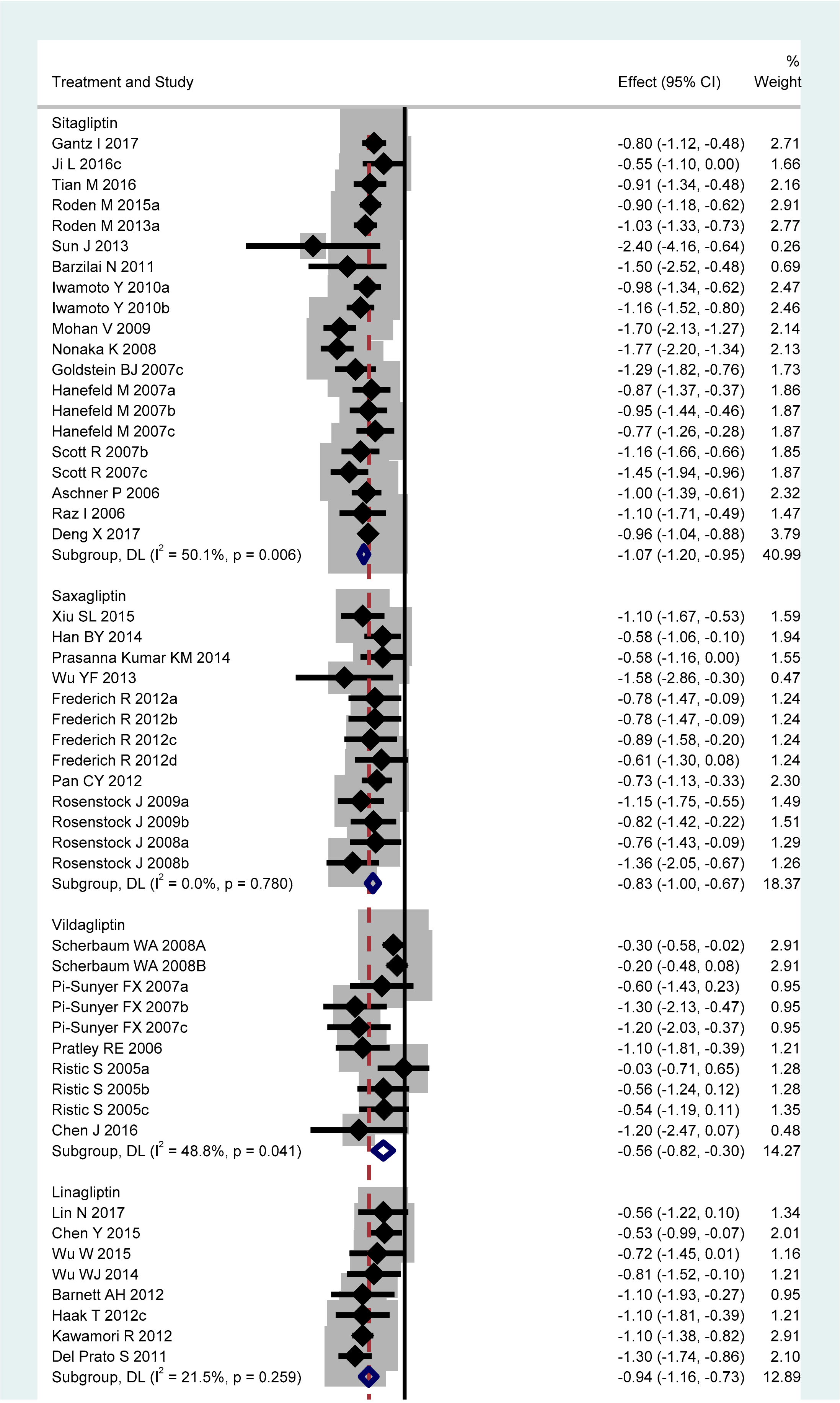
%
Treatment and Study
Effect (95% CI)
Weight
Sitagliptin
Gantz I 2017 -0.80 (-1.12, -0.48) 2.71
Ji L 2016c -0.55 (-1.10, 0.00) 1.66
Tian M 2016 -0.91 (-1.34, -0.48) 2.16
Roden M 2015a -0.90 (-1.18, -0.62) 2.91
Roden M 2013a -1.03 (-1.33, -0.73) 2.77
Sun J 2013 -2.40 (-4.16, -0.64) 0.26
Barzilai N 2011 -1.50 (-2.52, -0.48) 0.69
Iwamoto Y 2010a -0.98 (-1.34, -0.62) 2.47
Iwamoto Y 2010b -1.16 (-1.52, -0.80) 2.46
Mohan V 2009 -1.70 (-2.13, -1.27) 2.14
Nonaka K 2008 -1.77 (-2.20, -1.34) 2.13
Goldstein BJ 2007c -1.29 (-1.82, -0.76) 1.73
Hanefeld M 2007a -0.87 (-1.37, -0.37) 1.86
Hanefeld M 2007b -0.95 (-1.44, -0.46) 1.87
Hanefeld M 2007c -0.77 (-1.26, -0.28) 1.87
Scott R 2007b -1.16 (-1.66, -0.66) 1.85
Scott R 2007c -1.45 (-1.94, -0.96) 1.87
Aschner P 2006 -1.00 (-1.39, -0.61) 2.32
Raz I 2006 -1.10 (-1.71, -0.49) 1.47
Deng X 2017 -0.96 (-1.04, -0.88) 3.79
Subgroup, DL (I^2 = 50.1%, p = 0.006) -1.07 (-1.20, -0.95) 40.99
Saxagliptin
Xiu SL 2015 -1.10 (-1.67, -0.53) 1.59
Han BY 2014 -0.58 (-1.06, -0.10) 1.94
Prasanna Kumar KM 2014 -0.58 (-1.16, 0.00) 1.55
Wu YF 2013 -1.58 (-2.86, -0.30) 0.47
Frederich R 2012a -0.78 (-1.47, -0.09) 1.24
Frederich R 2012b -0.78 (-1.47, -0.09) 1.24
Frederich R 2012c -0.89 (-1.58, -0.20) 1.24
Frederich R 2012d -0.61 (-1.30, 0.08) 1.24
Pan CY 2012 -0.73 (-1.13, -0.33) 2.30
Rosenstock J 2009a -1.15 (-1.75, -0.55) 1.49
Rosenstock J 2009b -0.82 (-1.42, -0.22) 1.51
Rosenstock J 2008a -0.76 (-1.43, -0.09) 1.29
Rosenstock J 2008b -1.36 (-2.05, -0.67) 1.26
Subgroup, DL (I^2 = 0.0%, p = 0.780) -0.83 (-1.00, -0.67) 18.37
Vildagliptin
Scherbaum WA 2008A -0.30 (-0.58, -0.02) 2.91
Scherbaum WA 2008B -0.20 (-0.48, 0.08) 2.91
Pi-Sunyer FX 2007a -0.60 (-1.43, 0.23) 0.95
Pi-Sunyer FX 2007b -1.30 (-2.13, -0.47) 0.95
Pi-Sunyer FX 2007c -1.20 (-2.03, -0.37) 0.95
Pratley RE 2006 -1.10 (-1.81, -0.39) 1.21
Ristic S 2005a -0.03 (-0.71, 0.65) 1.28
Ristic S 2005b -0.56 (-1.24, 0.12) 1.28
Ristic S 2005c -0.54 (-1.19, 0.11) 1.35
Chen J 2016 -1.20 (-2.47, 0.07) 0.48
Subgroup, DL (I^2 = 48.8%, p = 0.041) -0.56 (-0.82, -0.30) 14.27
Linagliptin
Lin N 2017 -0.56 (-1.22, 0.10) 1.34
Chen Y 2015 -0.53 (-0.99, -0.07) 2.01
Wu W 2015 -0.72 (-1.45, 0.01) 1.16
Wu WJ 2014 -0.81 (-1.52, -0.10) 1.21
Barnett AH 2012 -1.10 (-1.93, -0.27) 0.95
Haak T 2012c -1.10 (-1.81, -0.39) 1.21
Kawamori R 2012 -1.10 (-1.38, -0.82) 2.91
Del Prato S 2011 -1.30 (-1.74, -0.86) 2.10
Subgroup, DL (I^2 = 21.5%, p = 0.259) -0.94 (-1.16, -0.73) 12.89

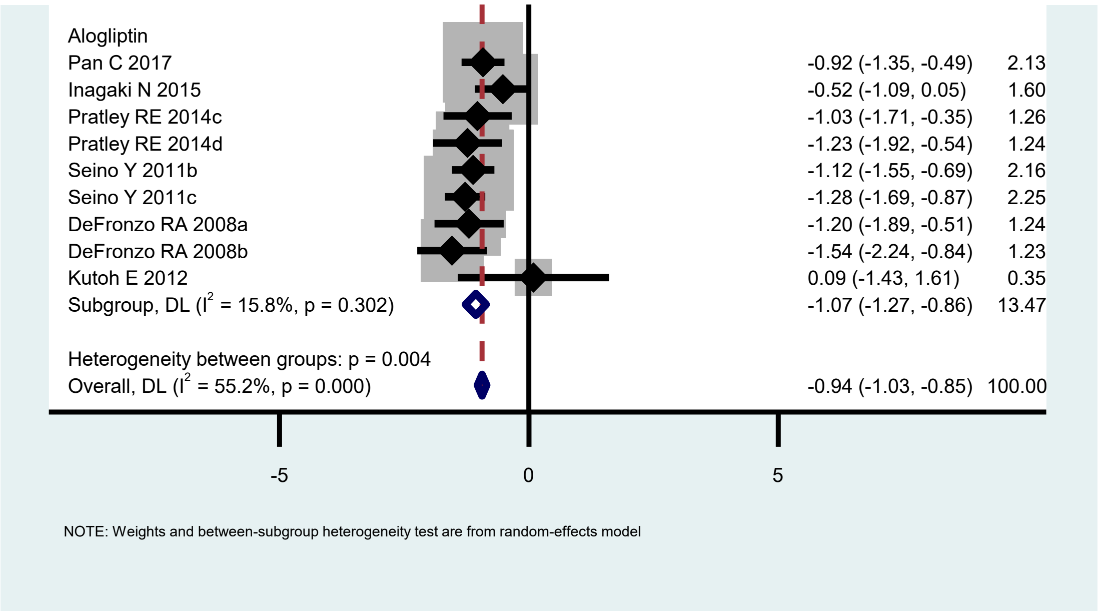

Figure S15. Meta-analysis results for change in fasting plasma glucose (mmol/l) of dipeptidyl peptidase-4 inhibitors vs placebo/lifestyle intervention

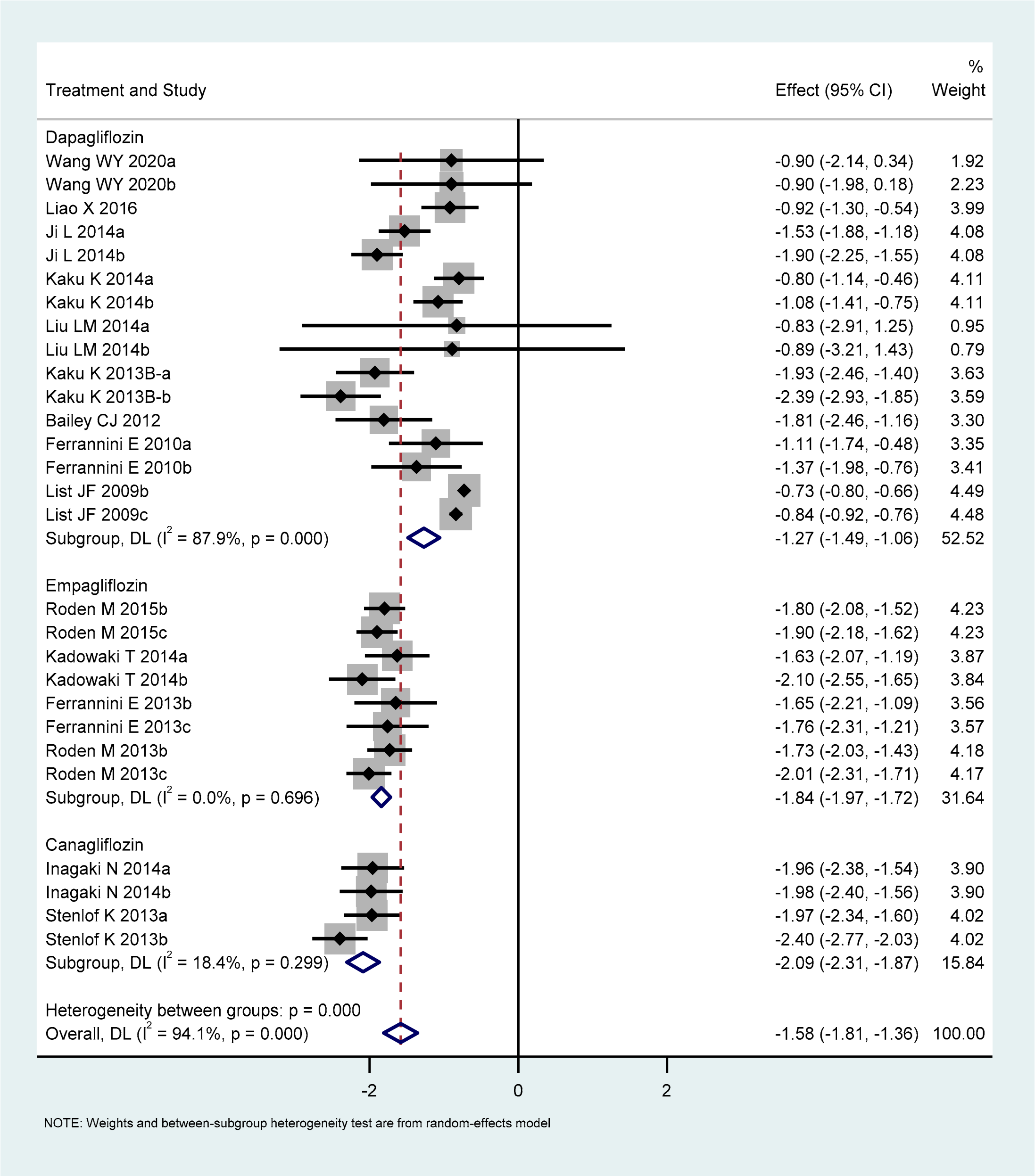

Figure S16. Meta-analysis results for change in fasting plasma glucose (mmol/l) of sodium-glucose cotransporter-2 inhibitors vs placebo/lifestyle intervention

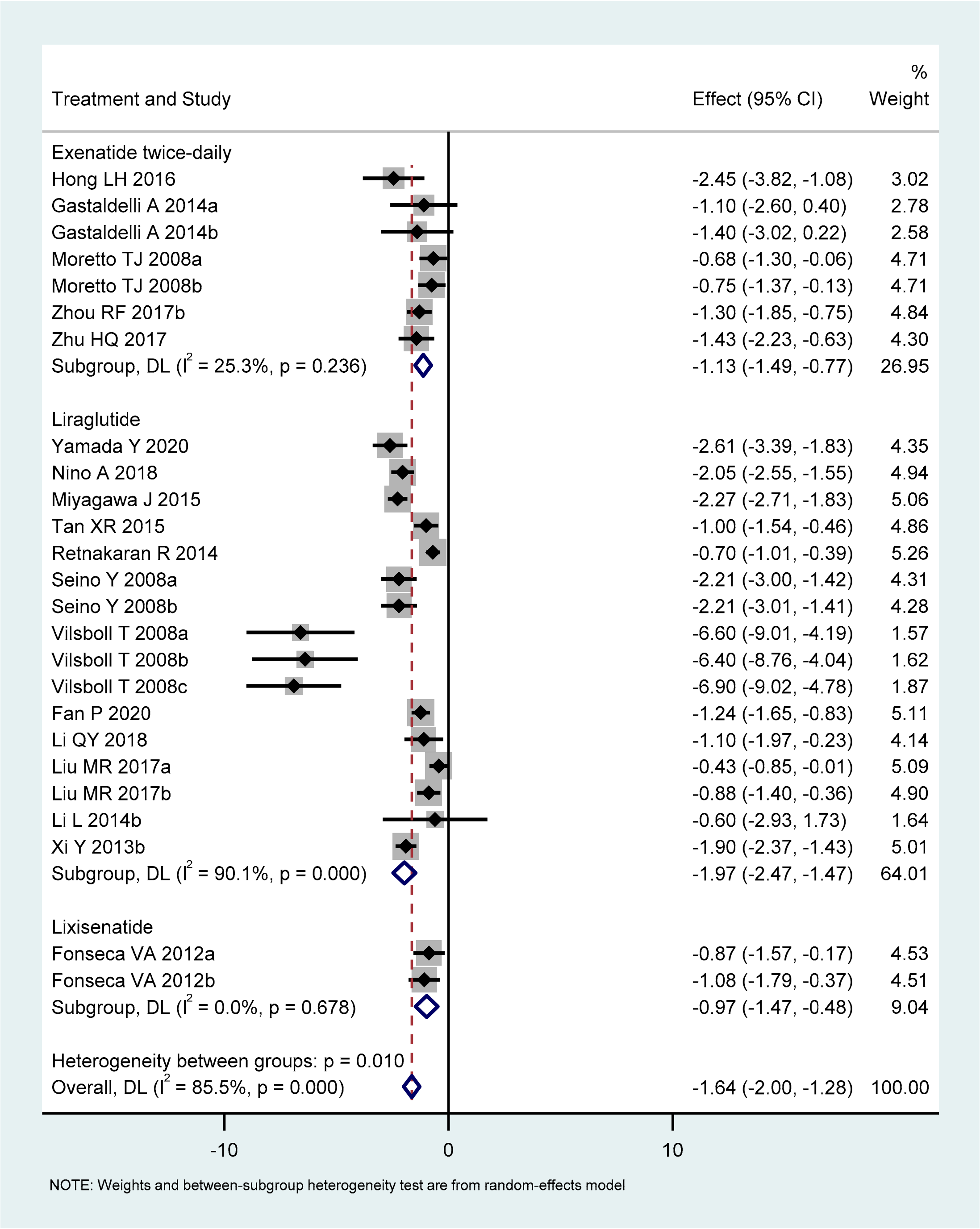

Figure S17. Meta-analysis results for change in fasting plasma glucose (mmol/l) of glucagon-like peptide-1 receptor agonists vs placebo/lifestyle intervention

【Body mass index, kg/m²】

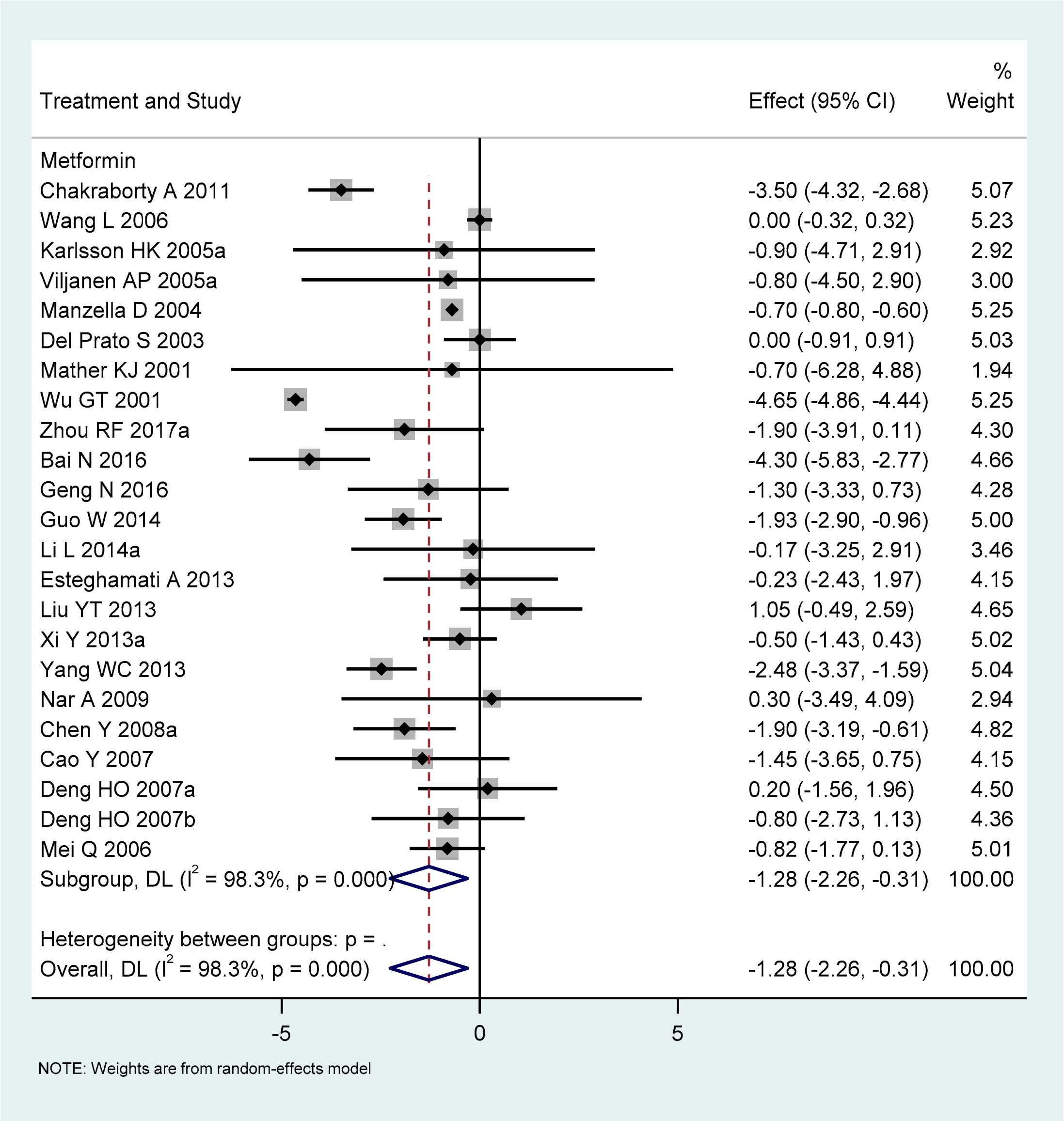

Figure S18. Meta-analysis results for change in body mass index (kg/m²) of metformin vs placebo/lifestyle intervention

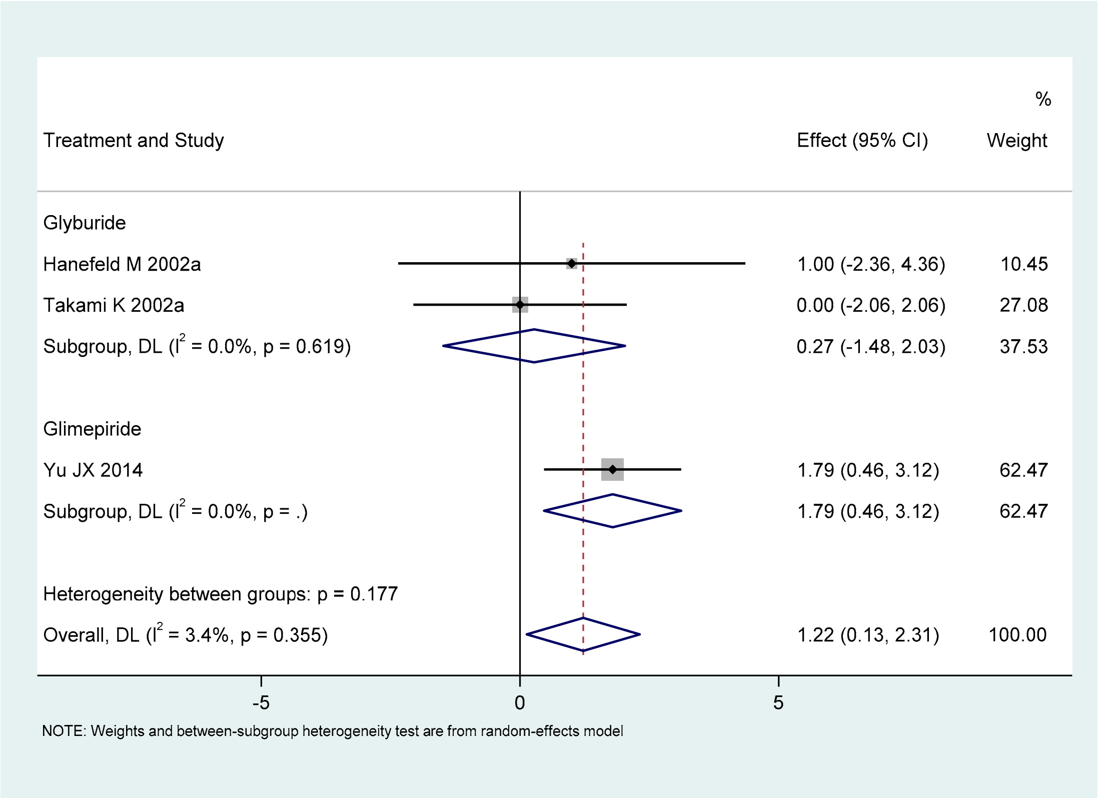

Figure S19. Meta-analysis results for change in body mass index (kg/m^2) of sulfonylureas vs placebo/lifestyle intervention

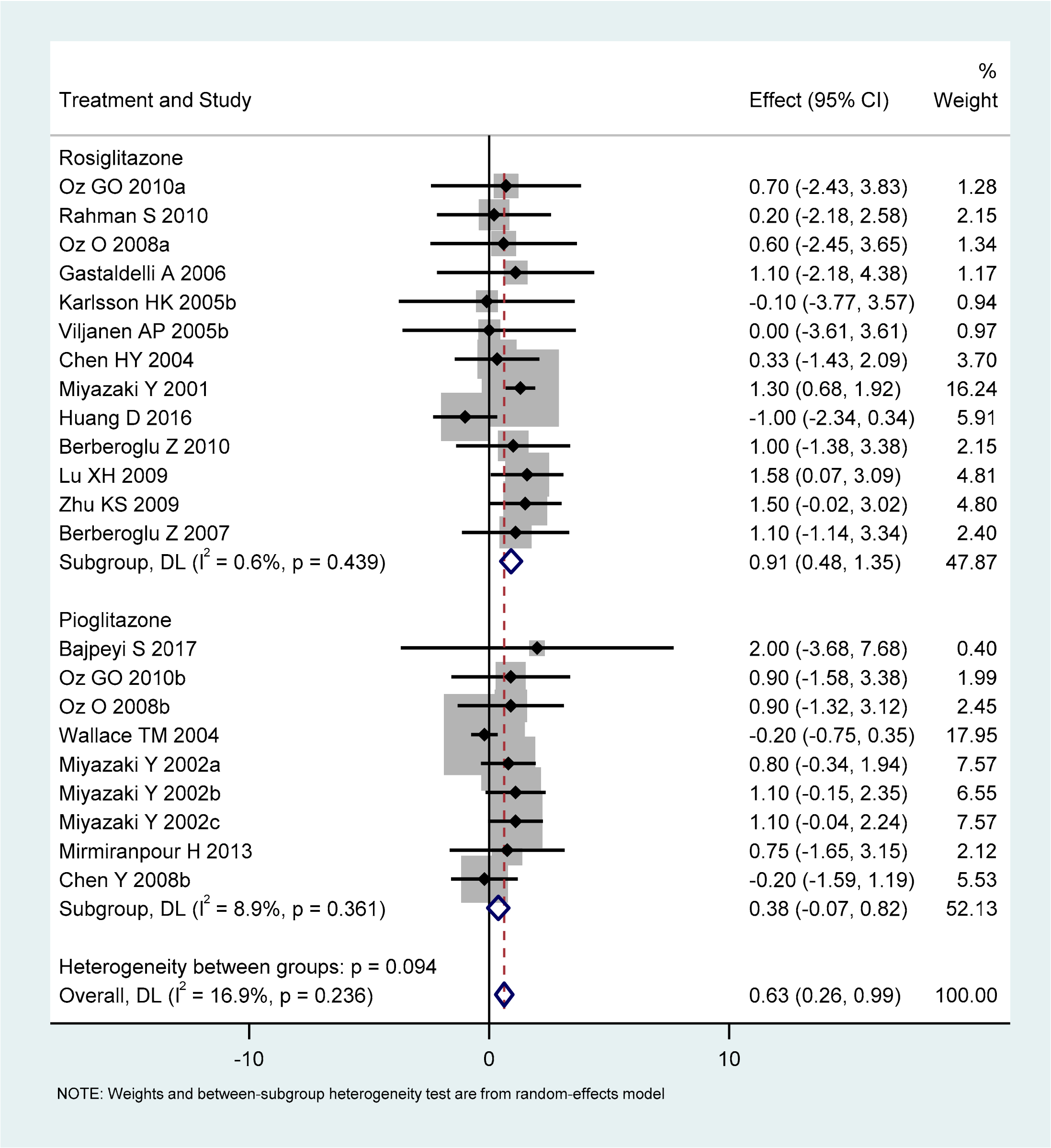

Figure S20. Meta-analysis results for change in body mass index (kg/m^2) of thiazolidinediones vs placebo/lifestyle intervention

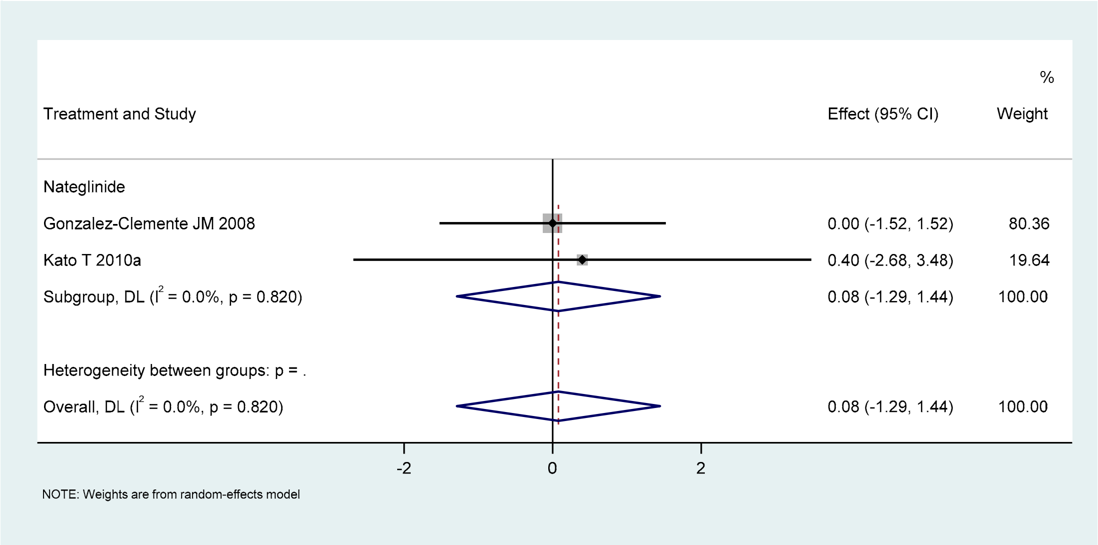

Figure S21. Meta-analysis results for change in body mass index (kg/m^2) of glinides vs placebo/lifestyle intervention

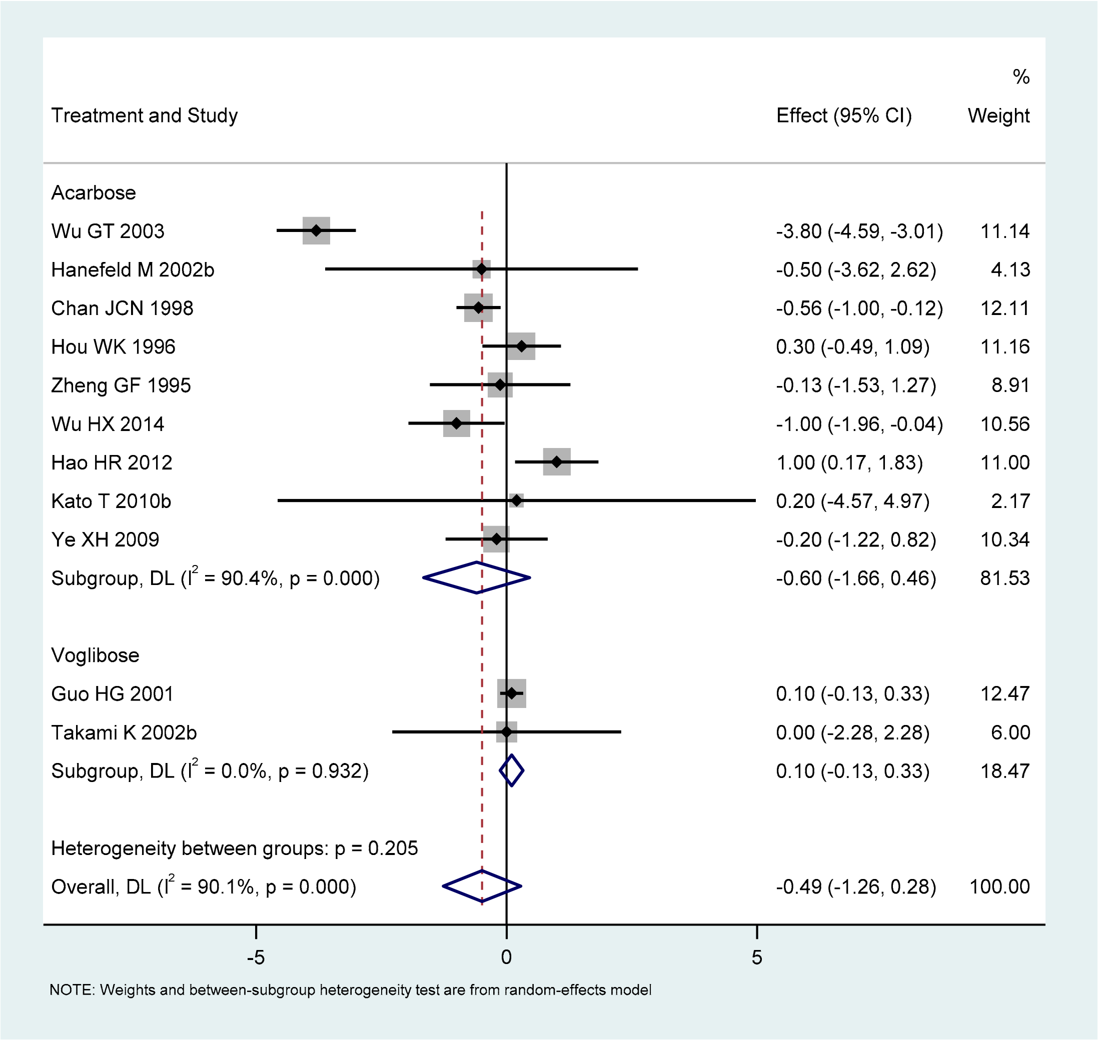

Figure S22. Meta-analysis results for change in body mass index (kg/m^2) of α-glucosidase inhibitors vs placebo/lifestyle intervention

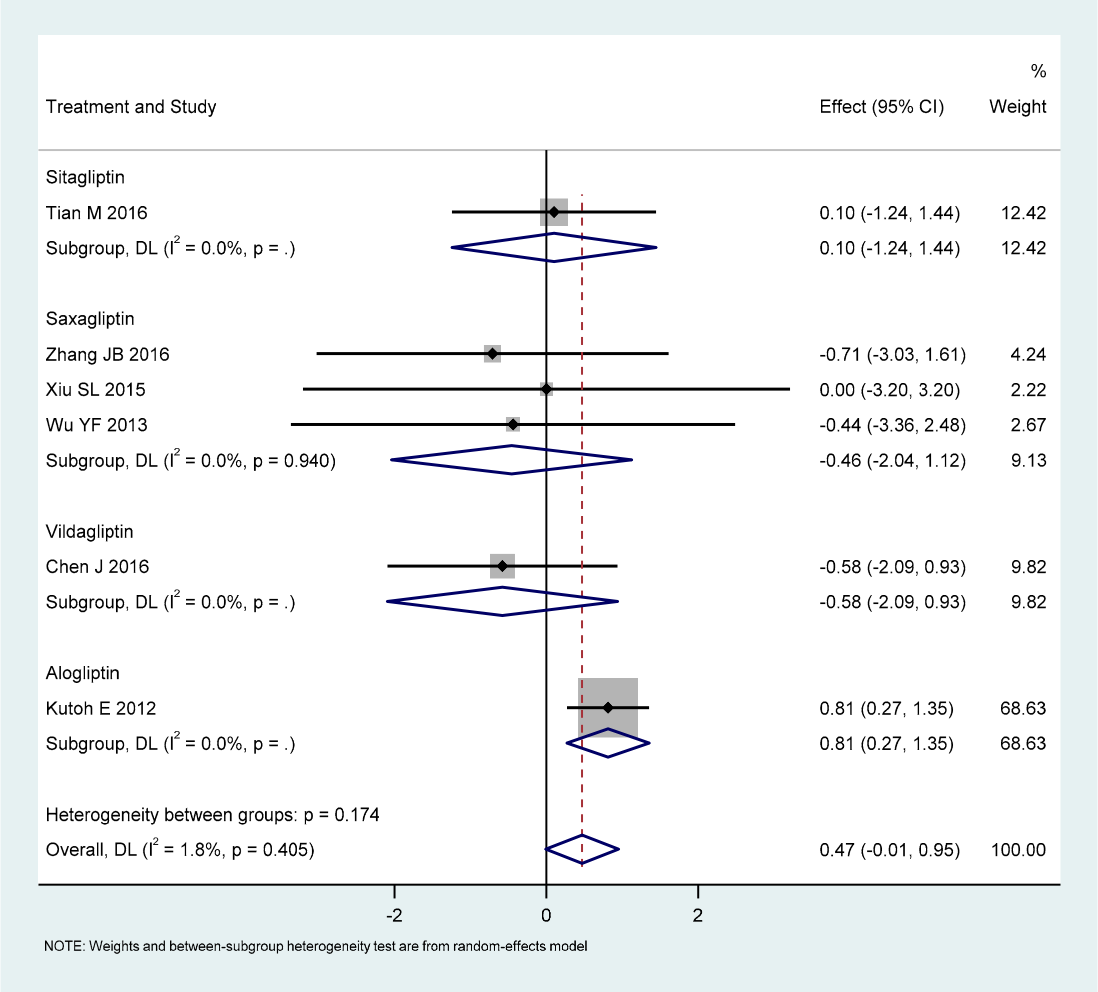

Figure S23. Meta-analysis results for change in body mass index (kg/m^2) of dipeptidyl peptidase-4 inhibitors vs placebo/lifestyle intervention

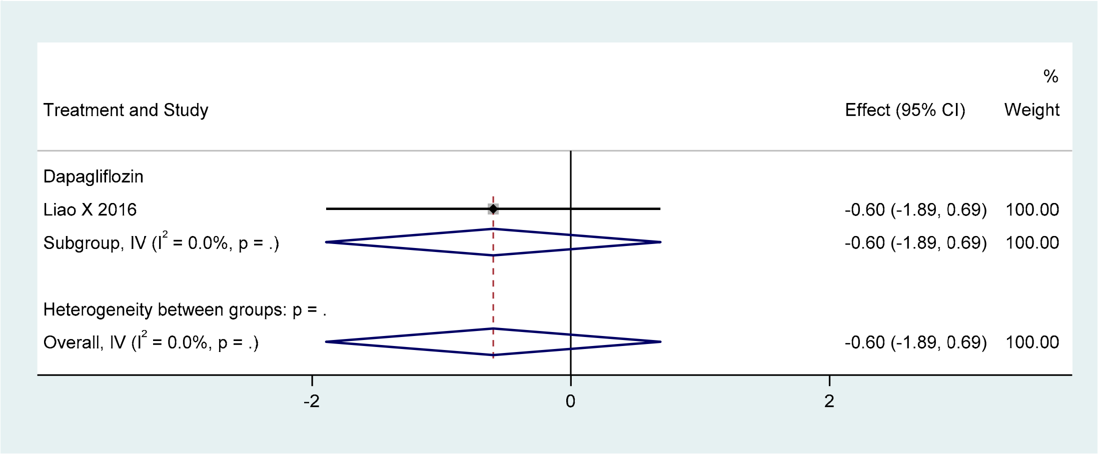

Figure S24. Meta-analysis results for change in body mass index (kg/m^2) of sodium-glucose cotransporter-2 inhibitors vs placebo/lifestyle intervention

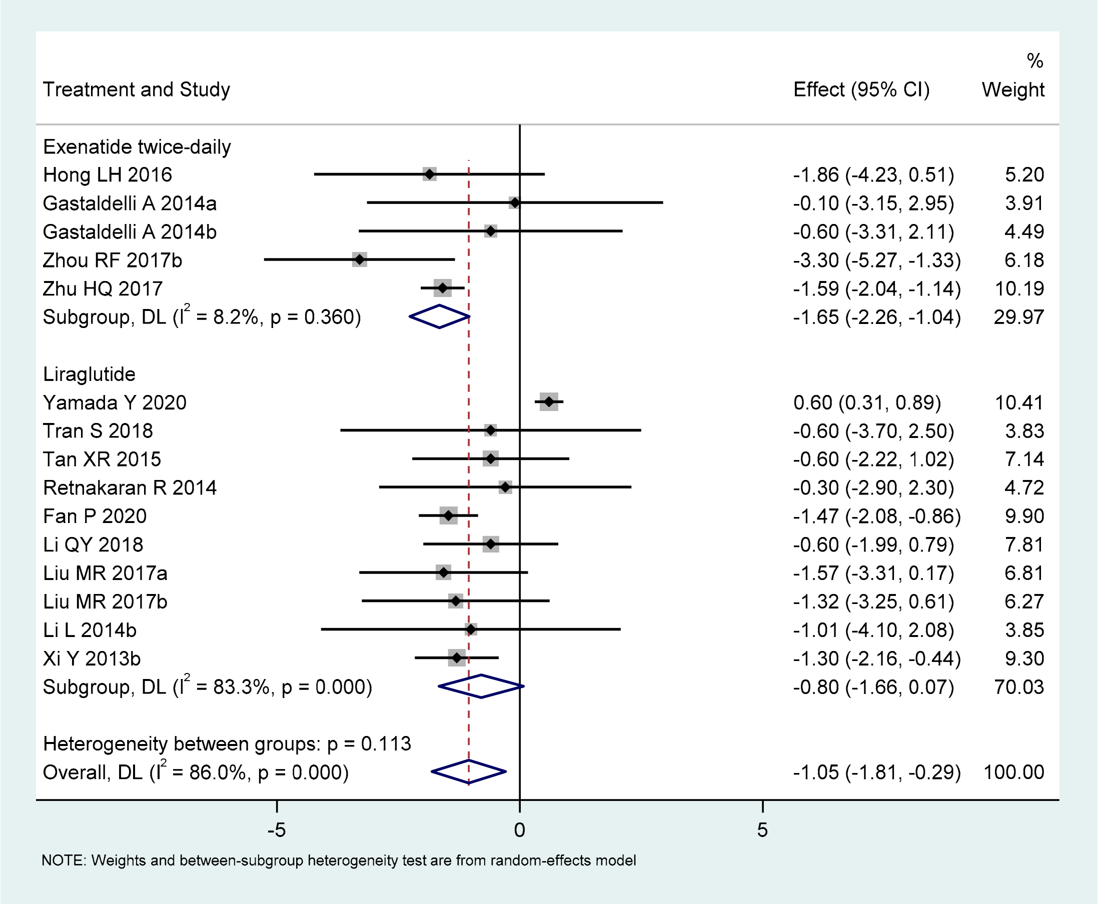

Figure S25. Meta-analysis results for change in body mass index (kg/m^2) of glucagon-like peptide-1 receptor agonists vs placebo/lifestyle intervention

【Total cholesterol, mmol/l】

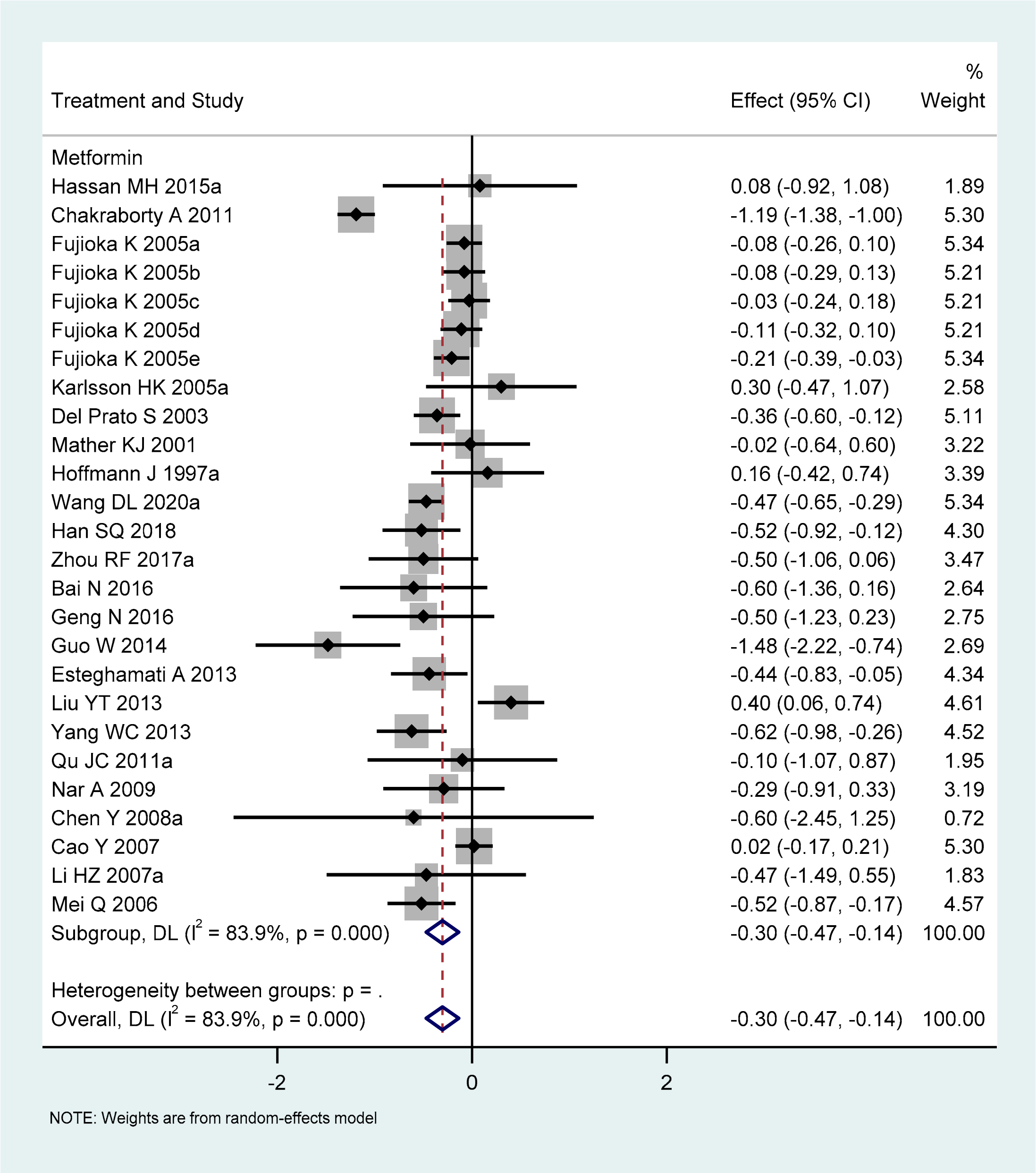

Figure S26. Meta-analysis results for change in total cholesterol (mmol/l) of metformin vs placebo/lifestyle intervention

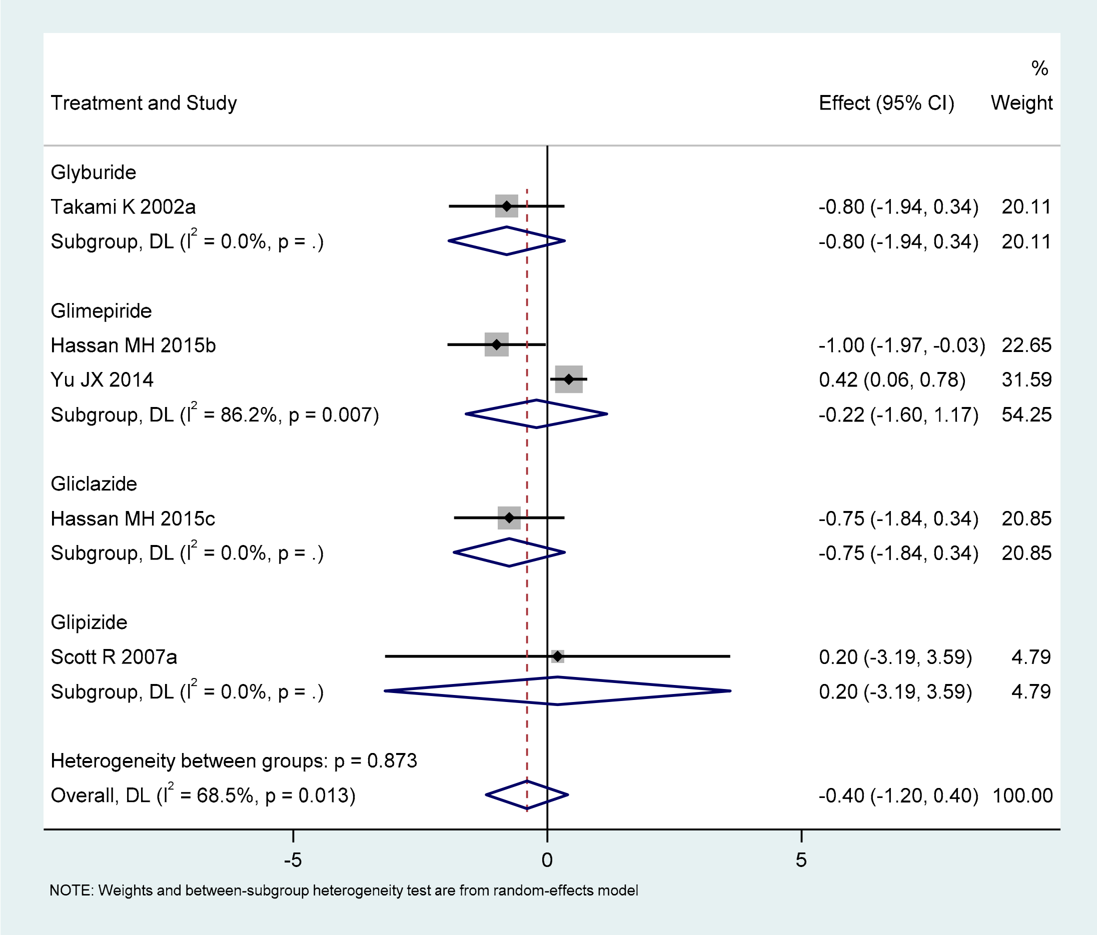

Figure S27. Meta-analysis results for change in total cholesterol (mmol/l) of sulfonylureas vs placebo/lifestyle intervention

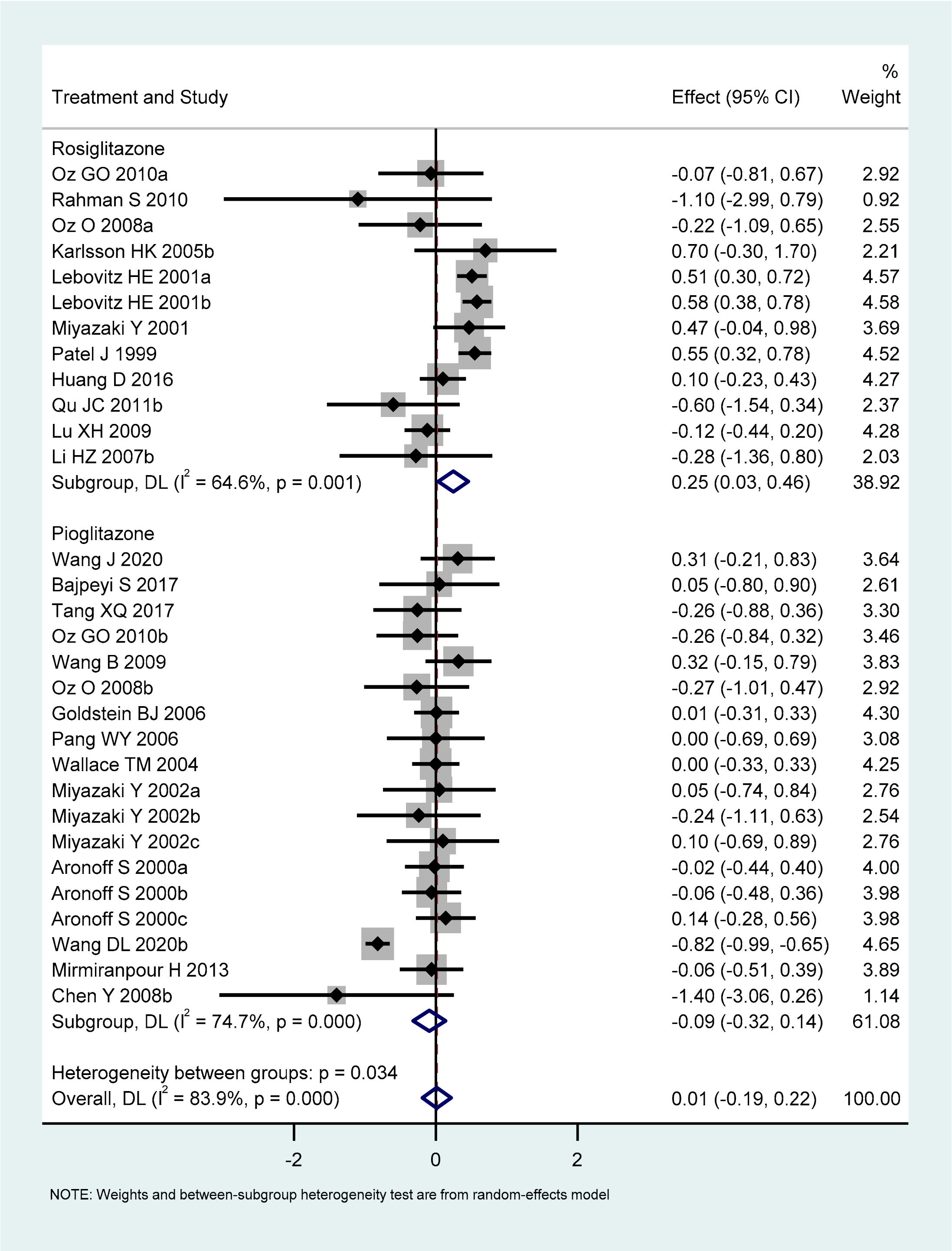

Figure S28. Meta-analysis results for change in total cholesterol (mmol/l) of thiazolidinediones vs placebo/lifestyle intervention

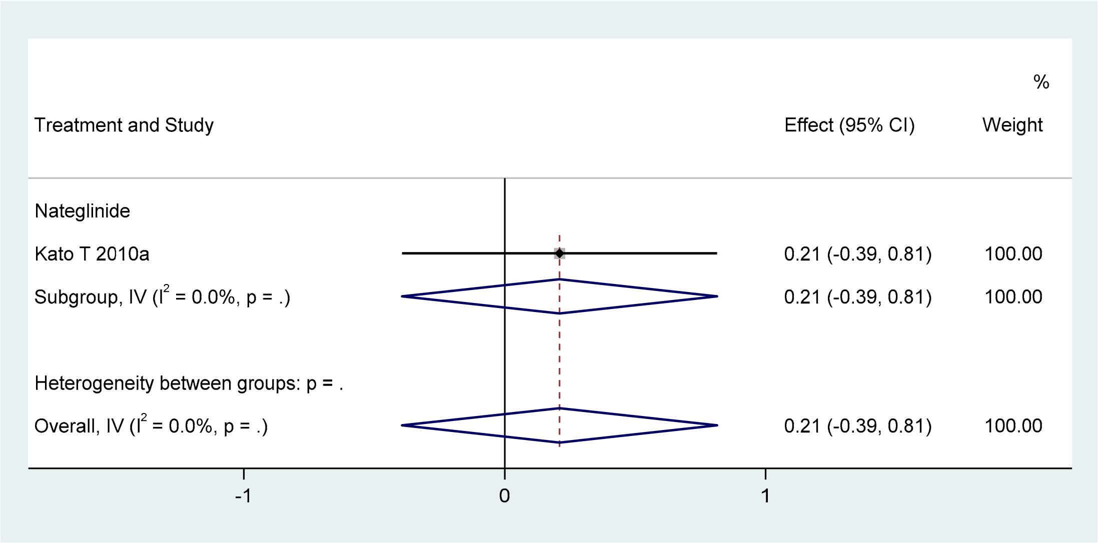

Figure S29. Meta-analysis results for change in total cholesterol (mmol/l) of glinides vs placebo/lifestyle intervention

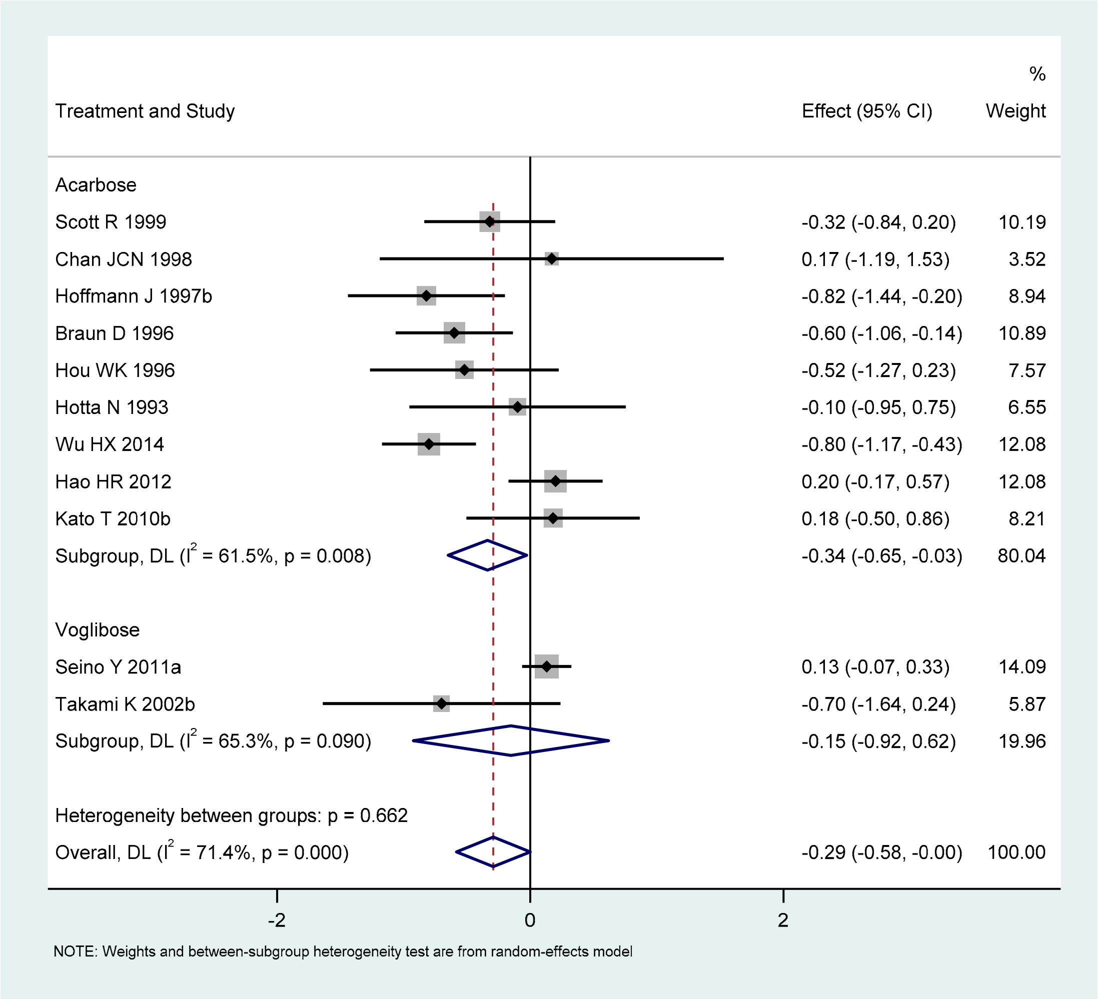

Figure S30. Meta-analysis results for change in total cholesterol (mmol/l) of α-glucosidase inhibitors vs placebo/lifestyle intervention

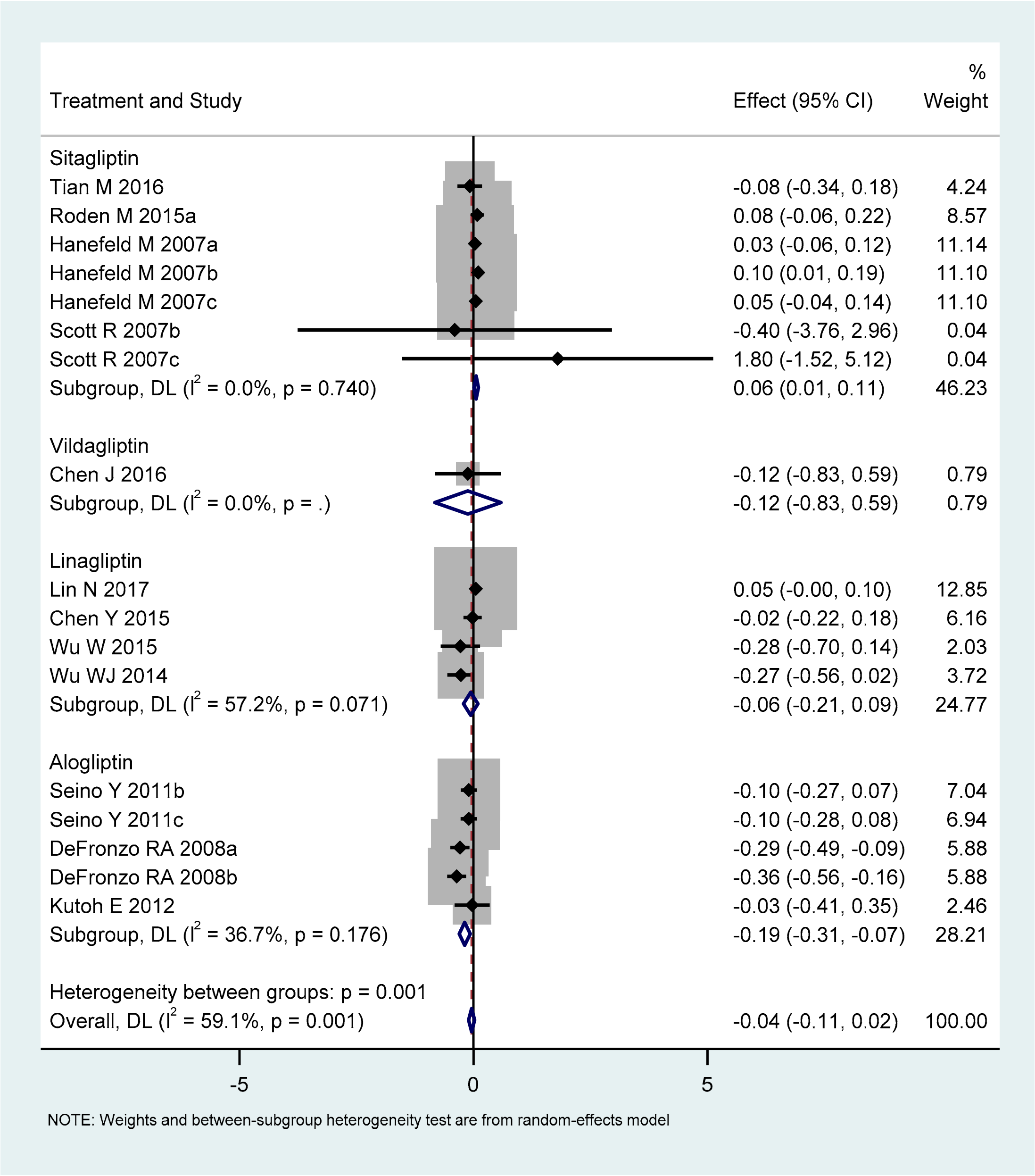

Figure S31. Meta-analysis results for change in total cholesterol (mmol/l) of dipeptidyl peptidase-4 inhibitors vs placebo/lifestyle intervention

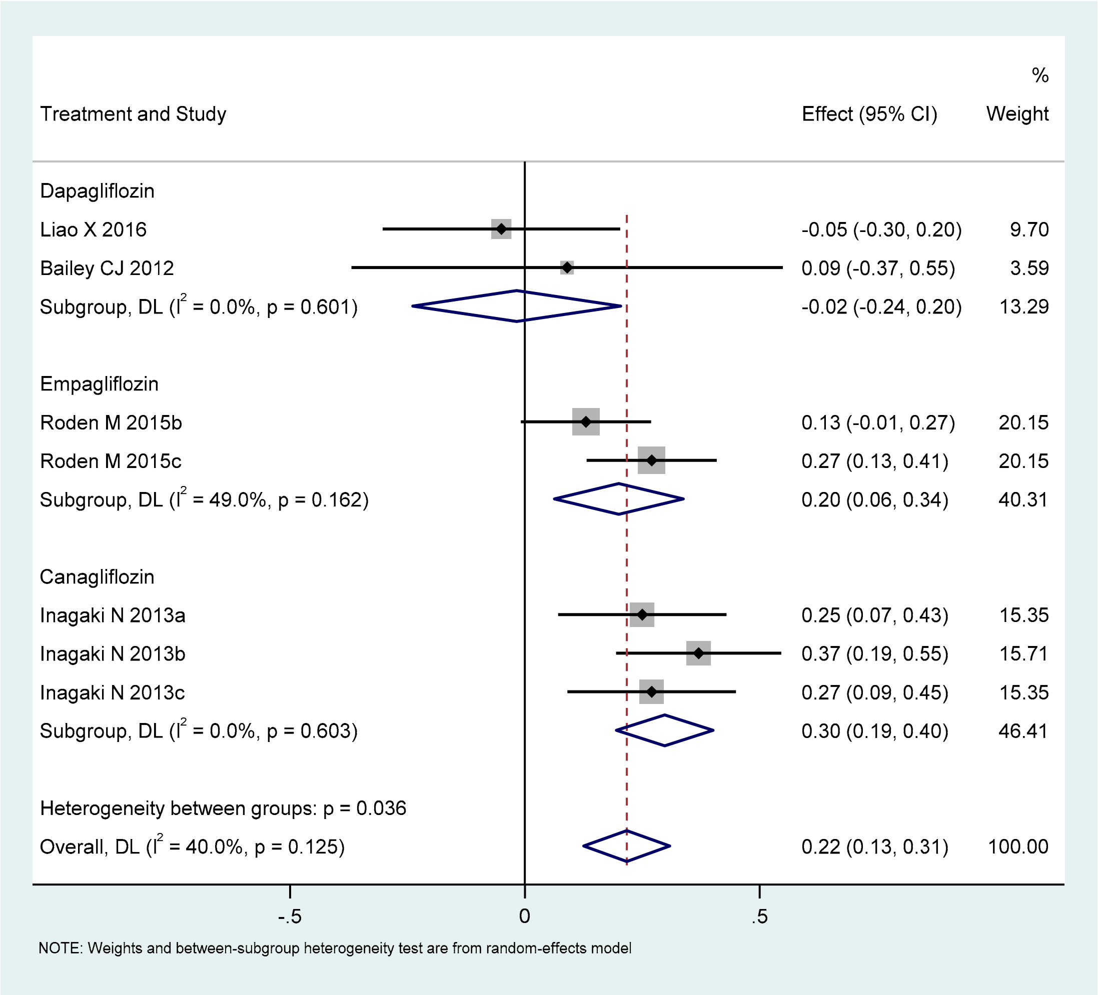

Figure S32. Meta-analysis results for change in total cholesterol (mmol/l) of sodium-glucose cotransporter-2 inhibitors vs placebo/lifestyle intervention

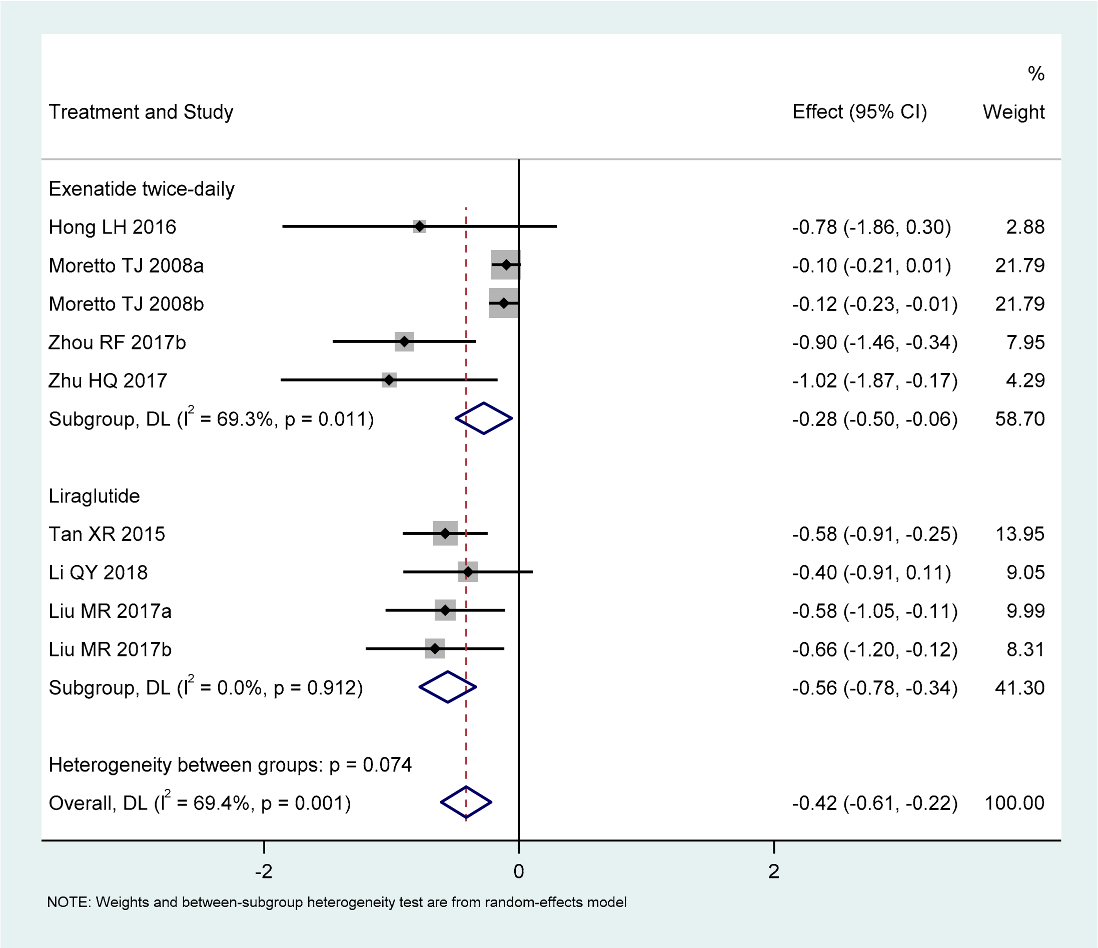

Figure S33. Meta-analysis results for change in total cholesterol (mmol/l) of glucagon-like peptide-1 receptor agonists vs placebo/lifestyle intervention

【High density lipoprotein-cholesterol, mmol/l】

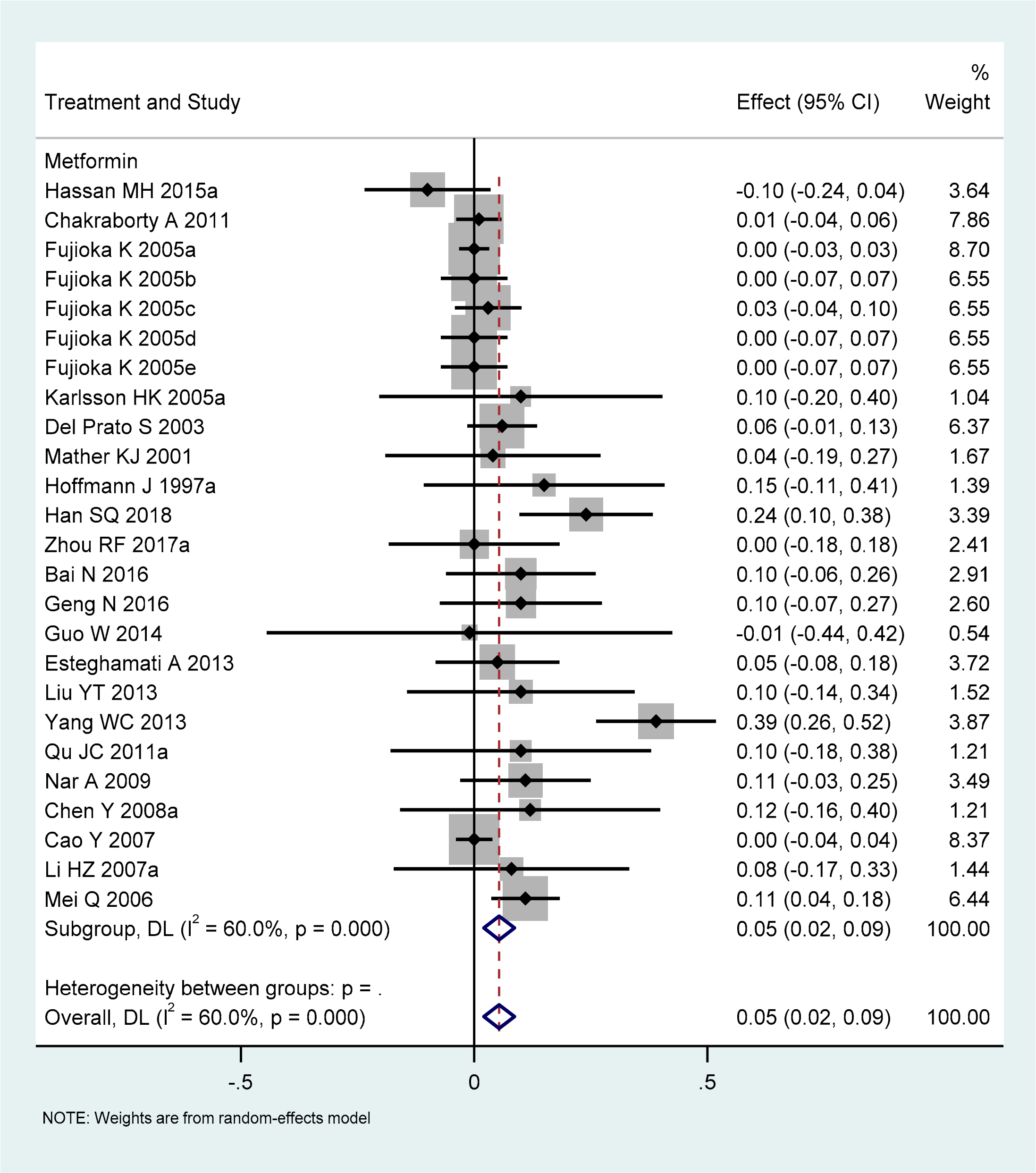

Figure S34. Meta-analysis results for change in high density lipoprotein-cholesterol (mmol/l) of metformin vs placebo/lifestyle intervention

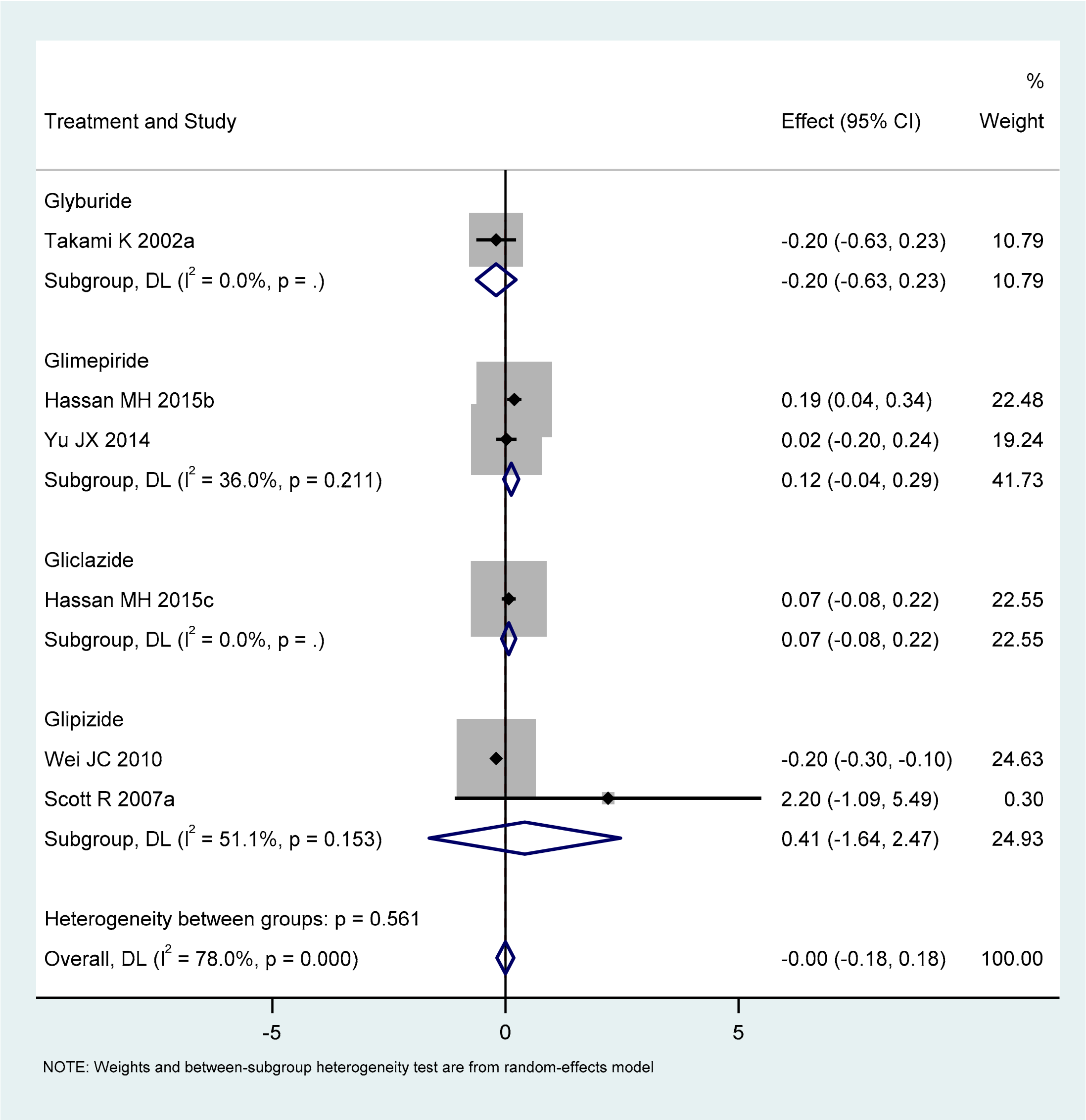

Figure S35. Meta-analysis results for change in high density lipoprotein-cholesterol (mmol/l) of sulfonylureas vs placebo/lifestyle intervention

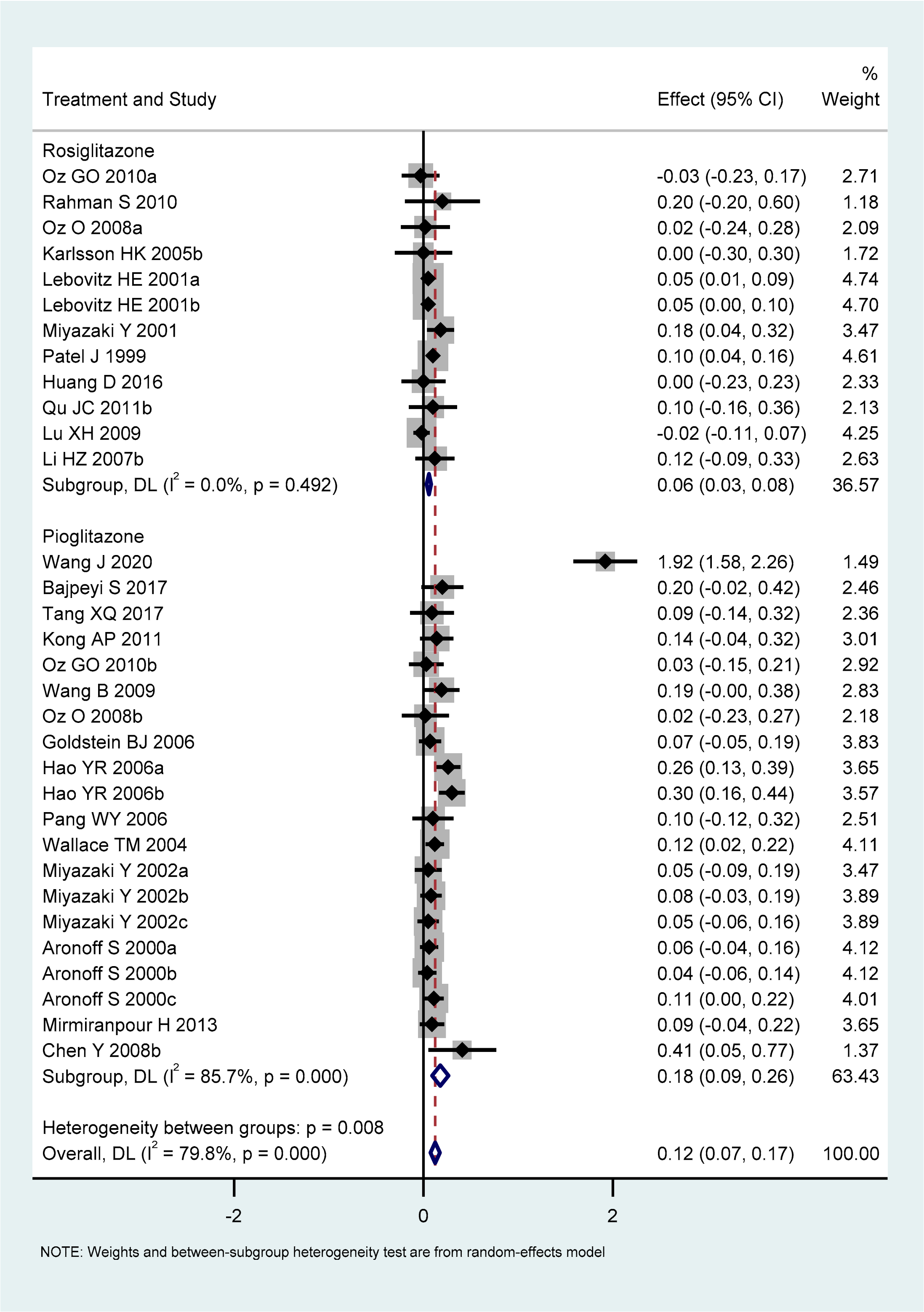

Figure S36. Meta-analysis results for change in high density lipoprotein-cholesterol (mmol/l) of thiazolidinediones vs placebo/lifestyle intervention

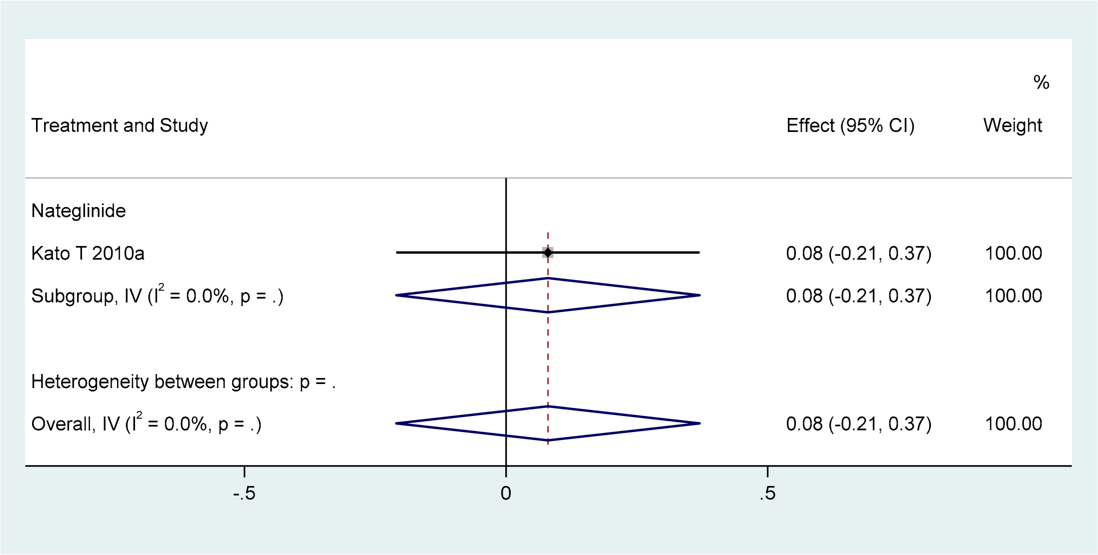

Figure S37. Meta-analysis results for change in high density lipoprotein-cholesterol (mmol/l) of glinides vs placebo/lifestyle intervention

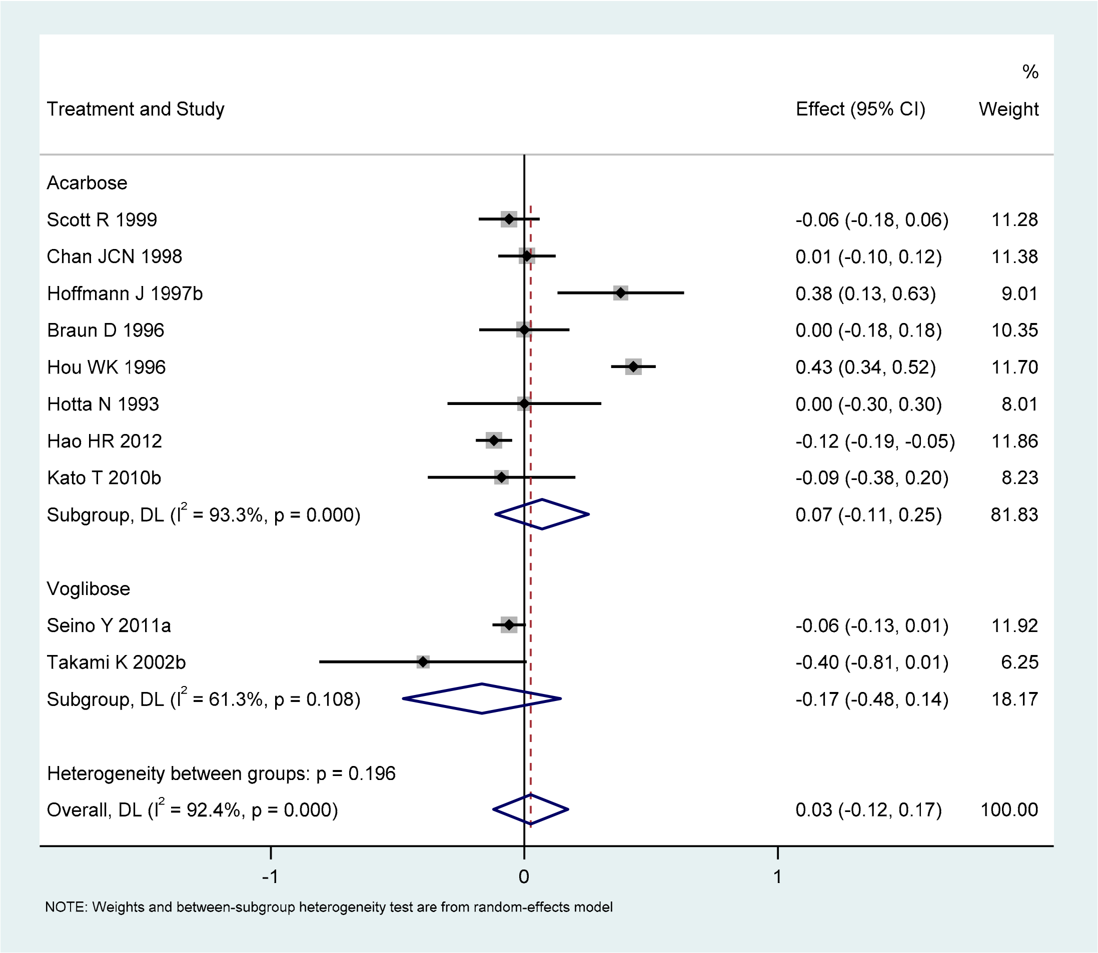

Figure S38. Meta-analysis results for change in high density lipoprotein-cholesterol (mmol/l) of α-glucosidase inhibitors vs placebo/lifestyle intervention

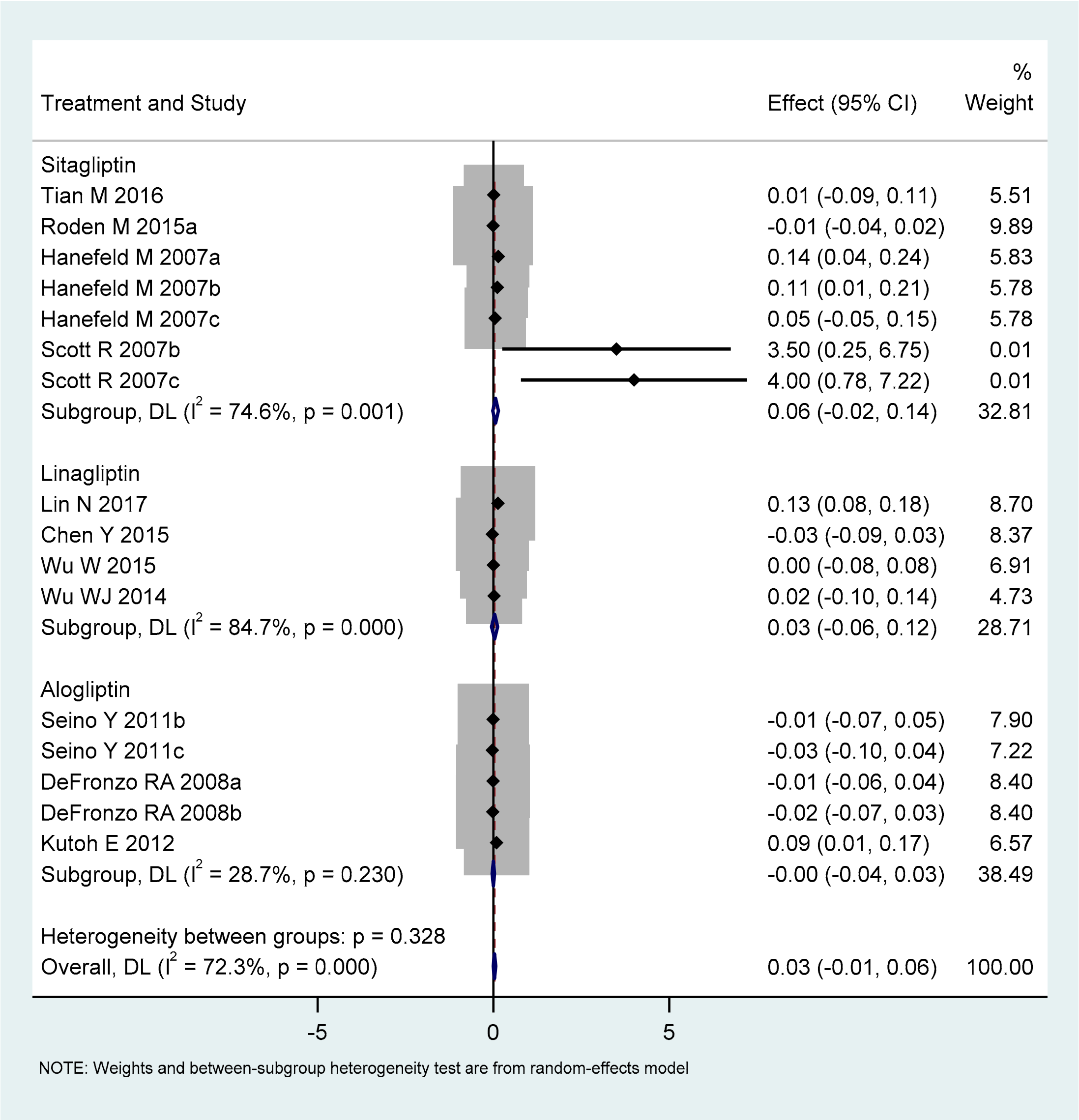

Figure S39. Meta-analysis results for change in high density lipoprotein-cholesterol (mmol/l) of dipeptidyl peptidase-4 inhibitors vs placebo/lifestyle intervention

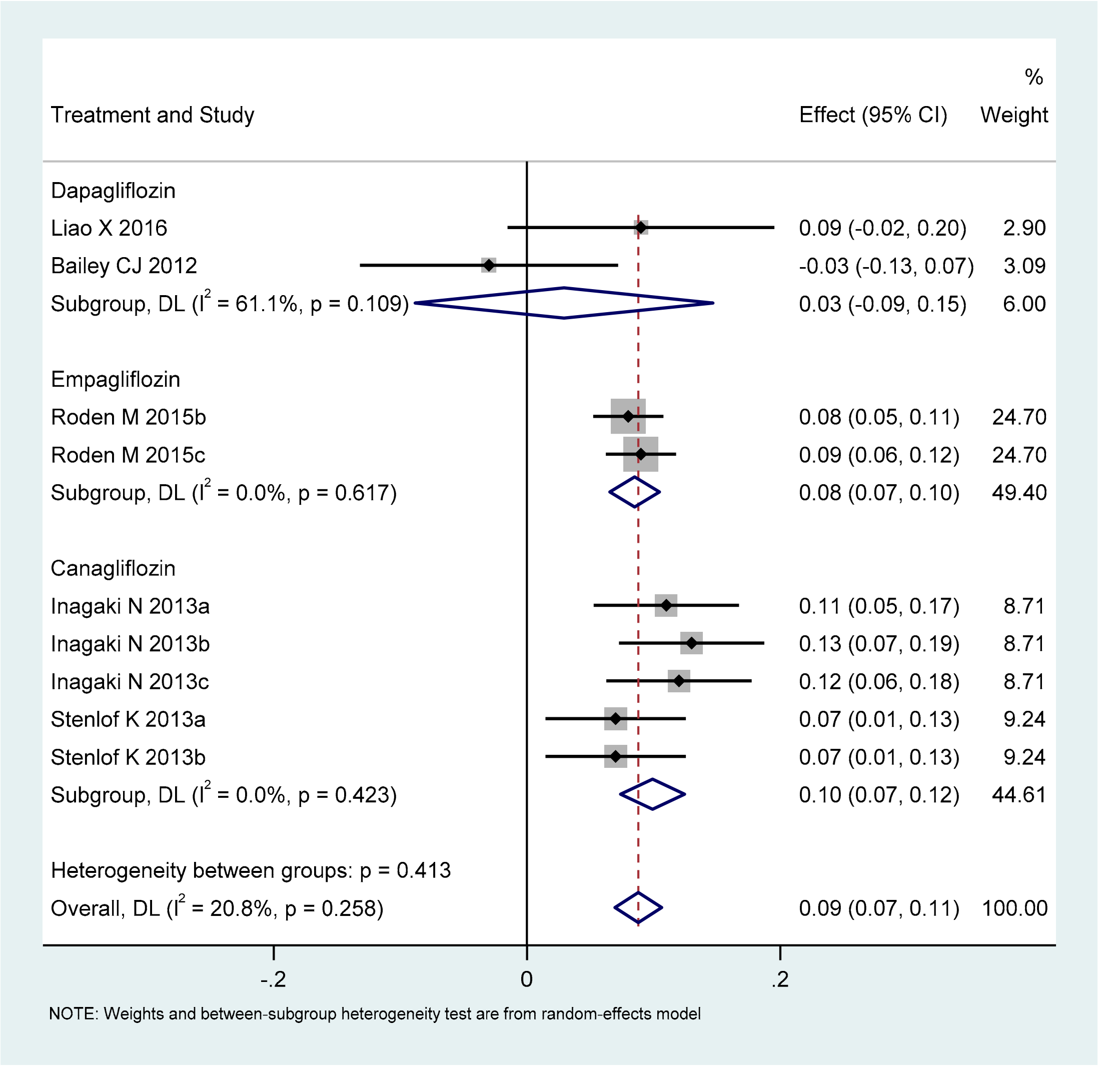

Figure S40. Meta-analysis results for change in high density lipoprotein-cholesterol (mmol/l) of sodium-glucose cotransporter-2 inhibitors vs placebo/lifestyle intervention

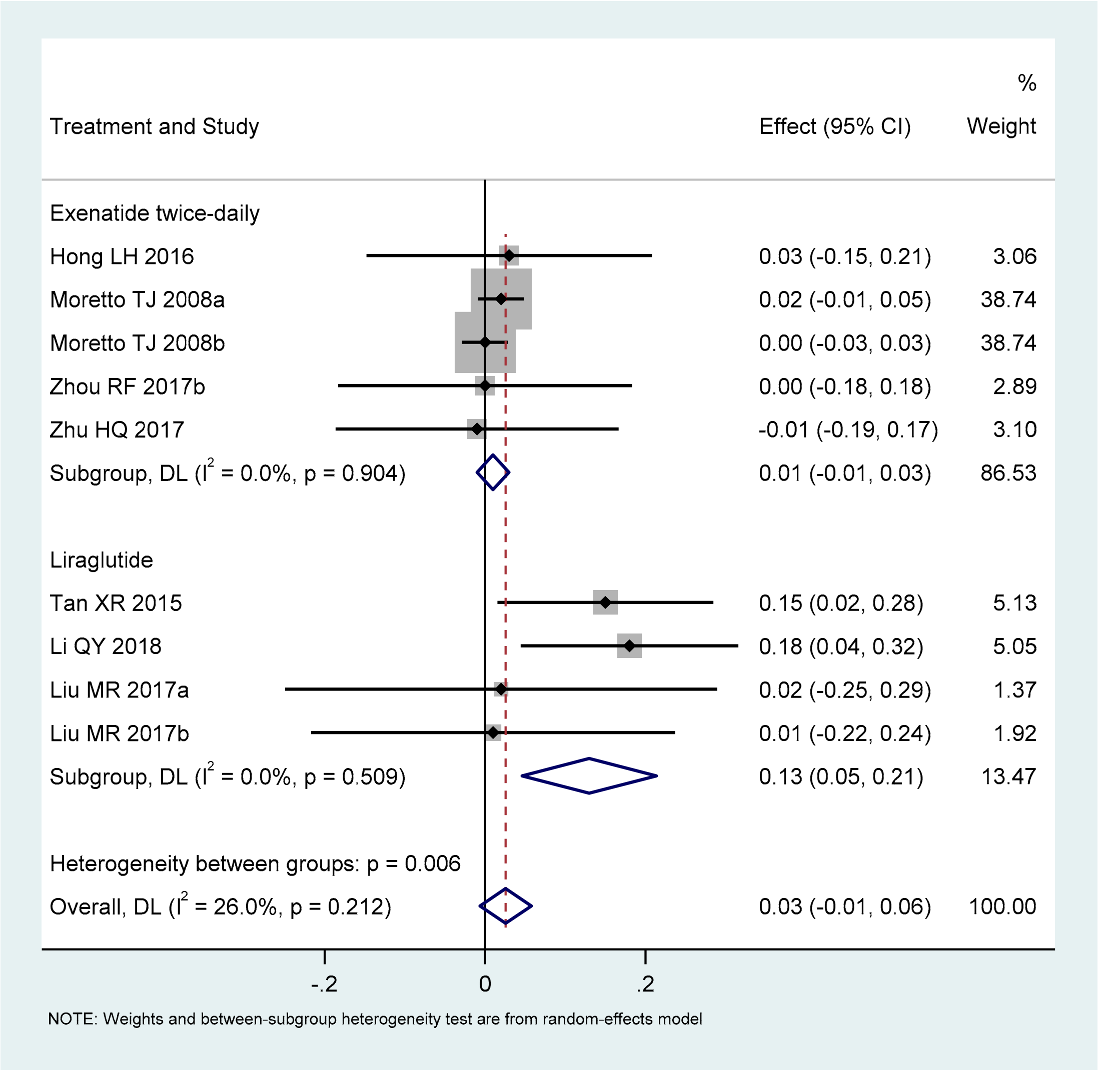

Figure S41. Meta-analysis results for change in high density lipoprotein-cholesterol (mmol/l) of glucagon-like peptide-1 receptor agonists vs placebo/lifestyle intervention

【Systolic blood pressure, mmHg】

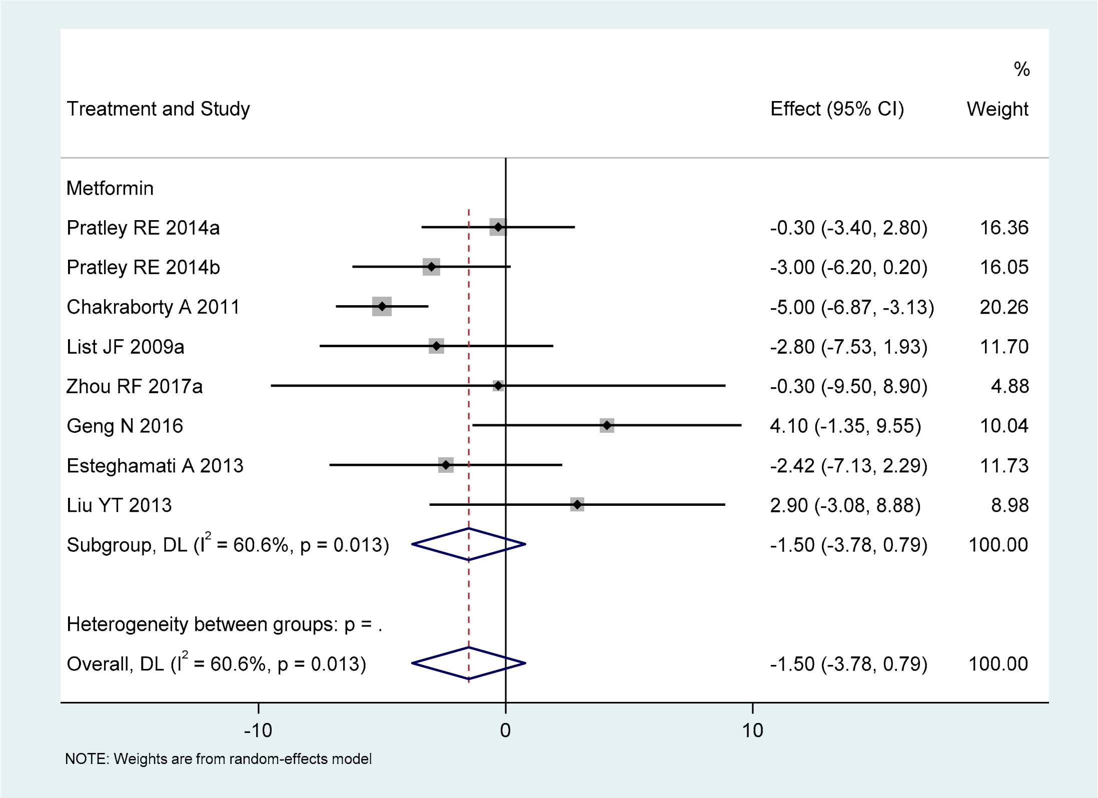

Figure S42. Meta-analysis results for change in systolic blood pressure (mmHg) of metformin vs placebo/lifestyle intervention

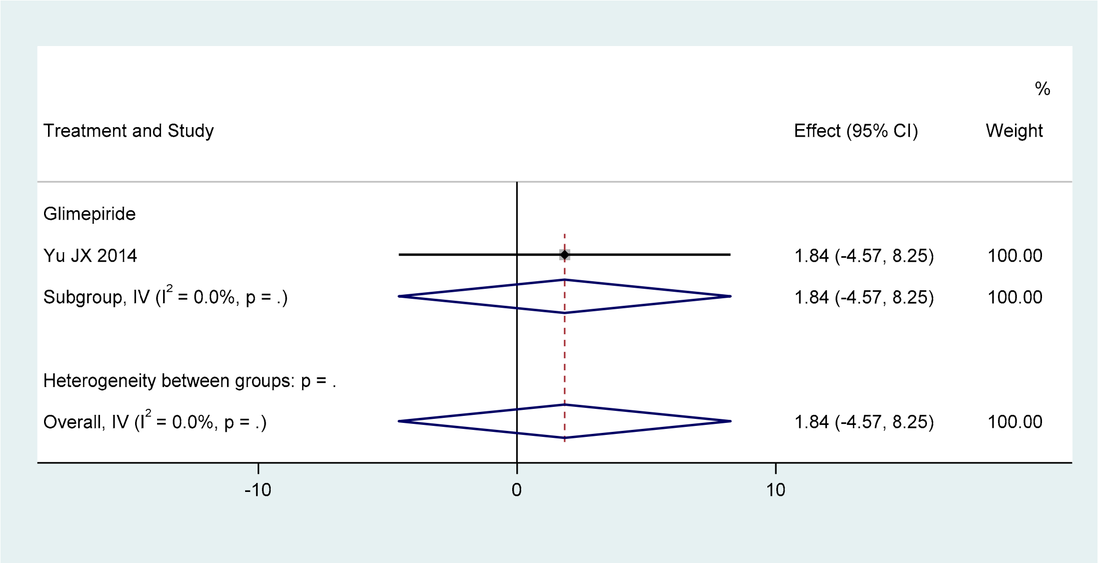

Figure S43. Meta-analysis results for change in systolic blood pressure (mmHg) of sulfonylureas vs placebo/lifestyle intervention

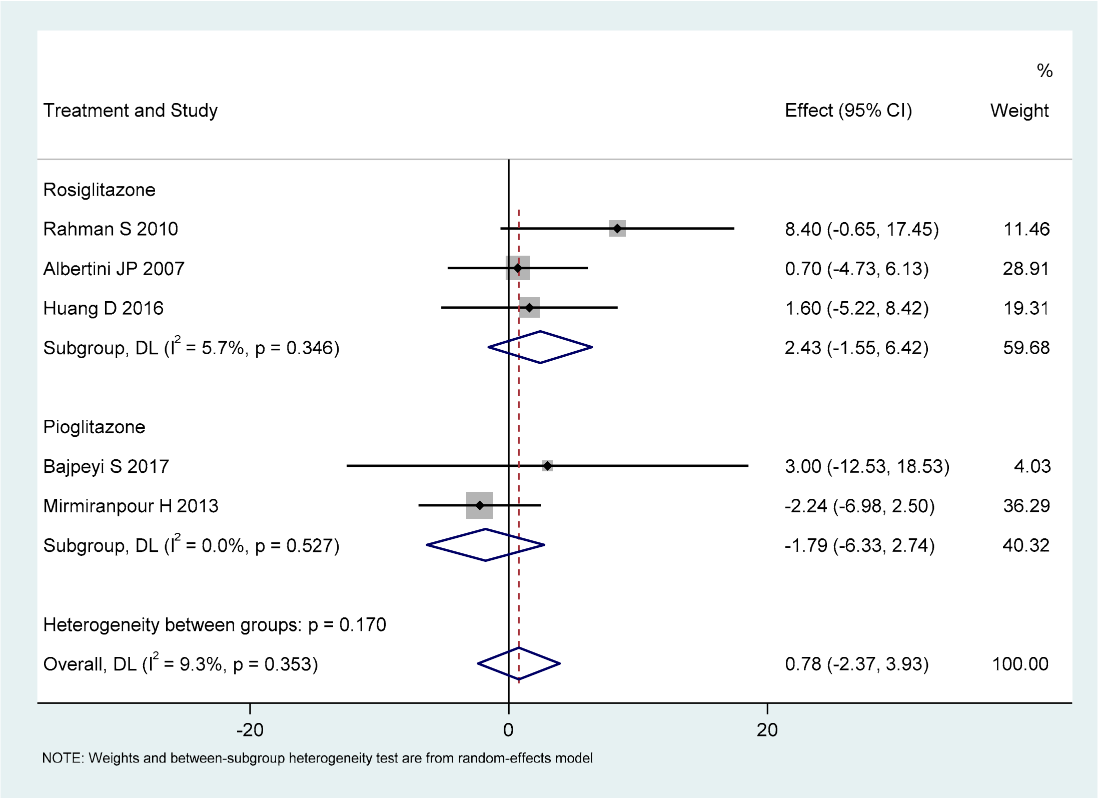

Figure S44. Meta-analysis results for change in systolic blood pressure (mmHg) of thiazolidinediones vs placebo/lifestyle intervention

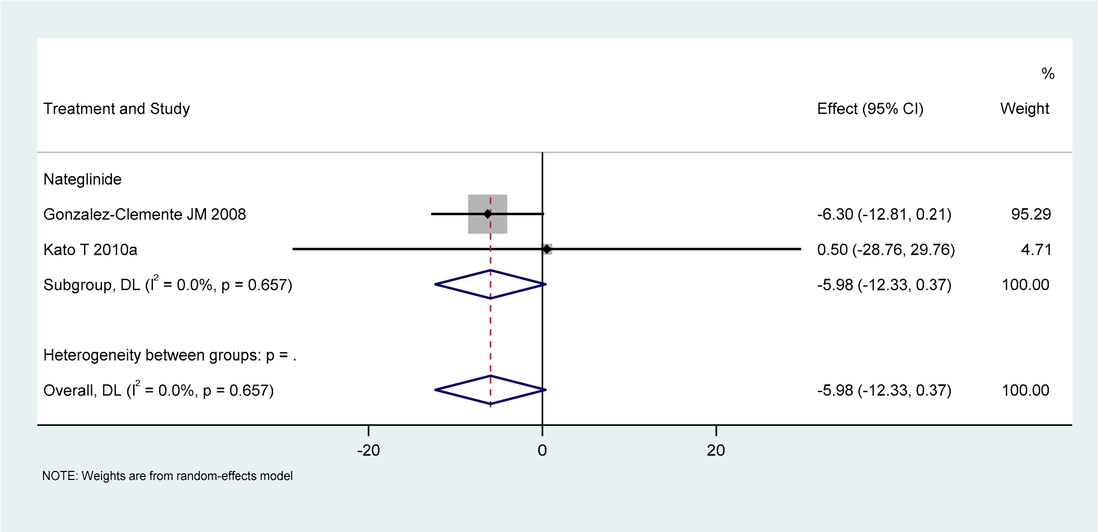

Figure S45. Meta-analysis results for change in systolic blood pressure (mmHg) of glinides vs placebo/lifestyle intervention

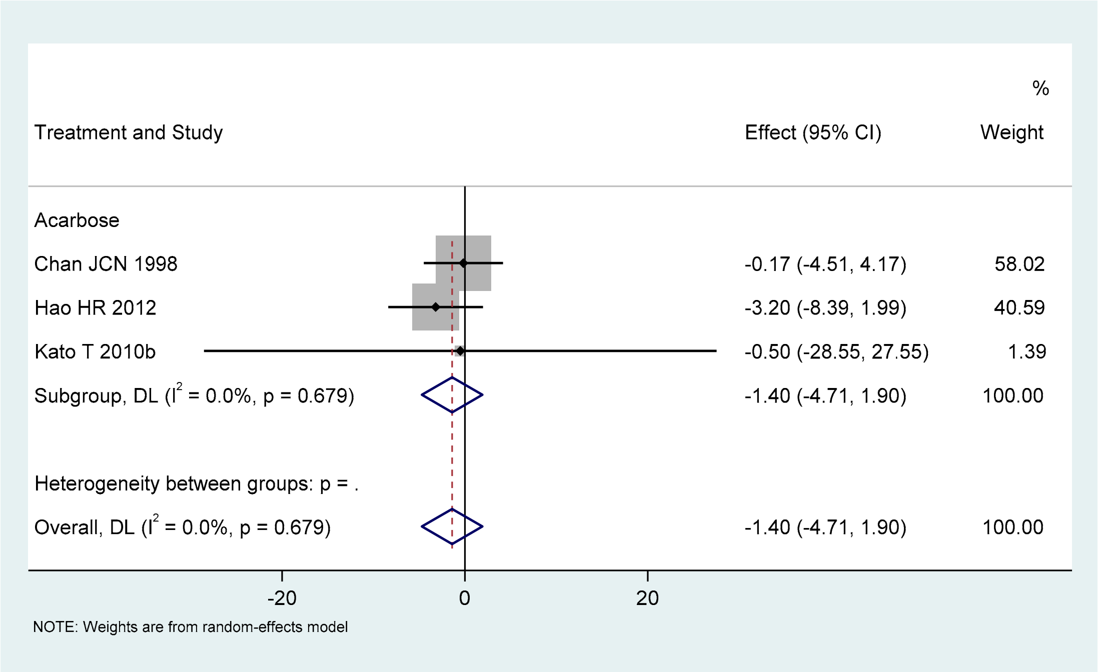

Figure S46. Meta-analysis results for change in systolic blood pressure (mmHg) of α-glucosidase inhibitors vs placebo/lifestyle intervention

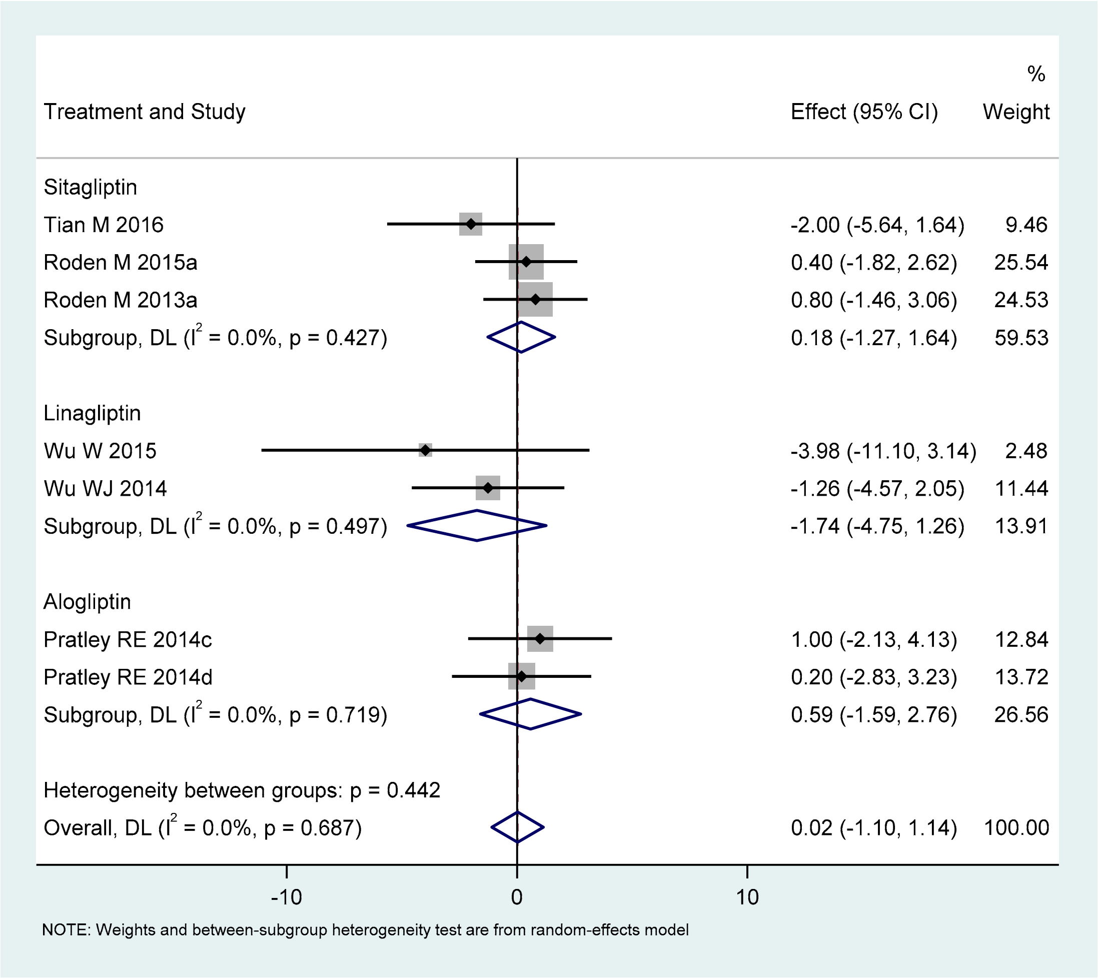

Figure S47. Meta-analysis results for change in systolic blood pressure (mmHg) of dipeptidyl peptidase-4 inhibitors vs placebo/lifestyle intervention

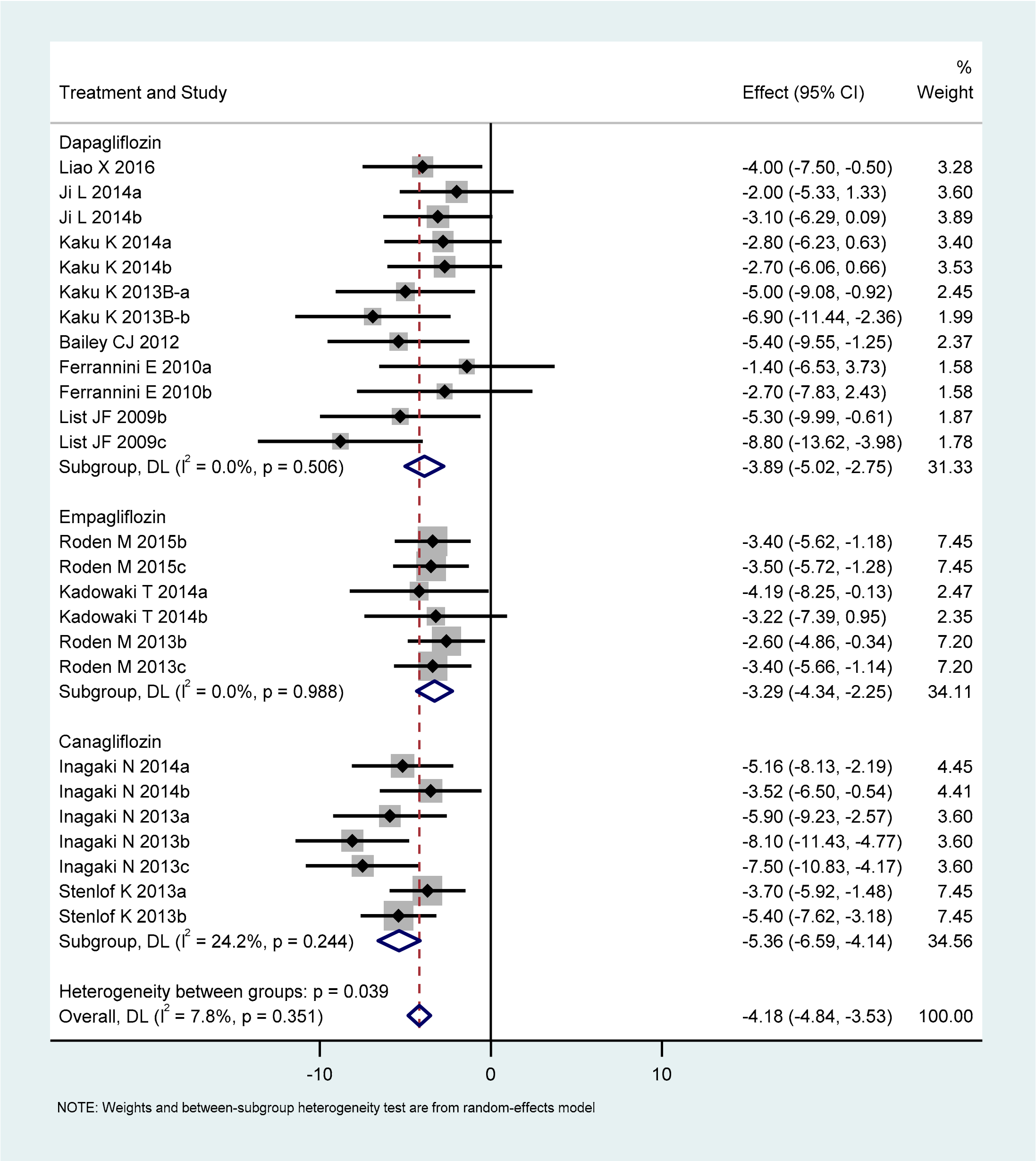

Figure S48. Meta-analysis results for change in systolic blood pressure (mmHg) of sodium-glucose cotransporter-2 inhibitors vs placebo/lifestyle intervention

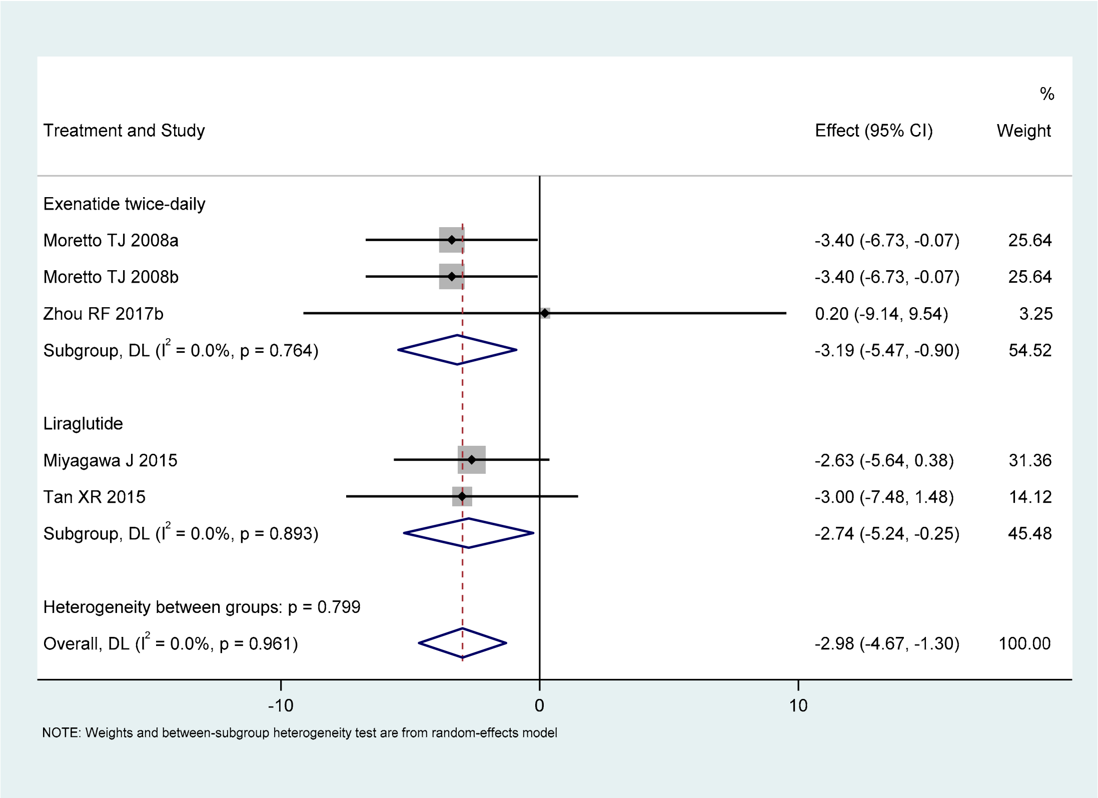

Figure S49. Meta-analysis results for change in systolic blood pressure (mmHg) of glucagon-like peptide-1 receptor agonists vs placebo/lifestyle intervention

【Hypoglycemia】

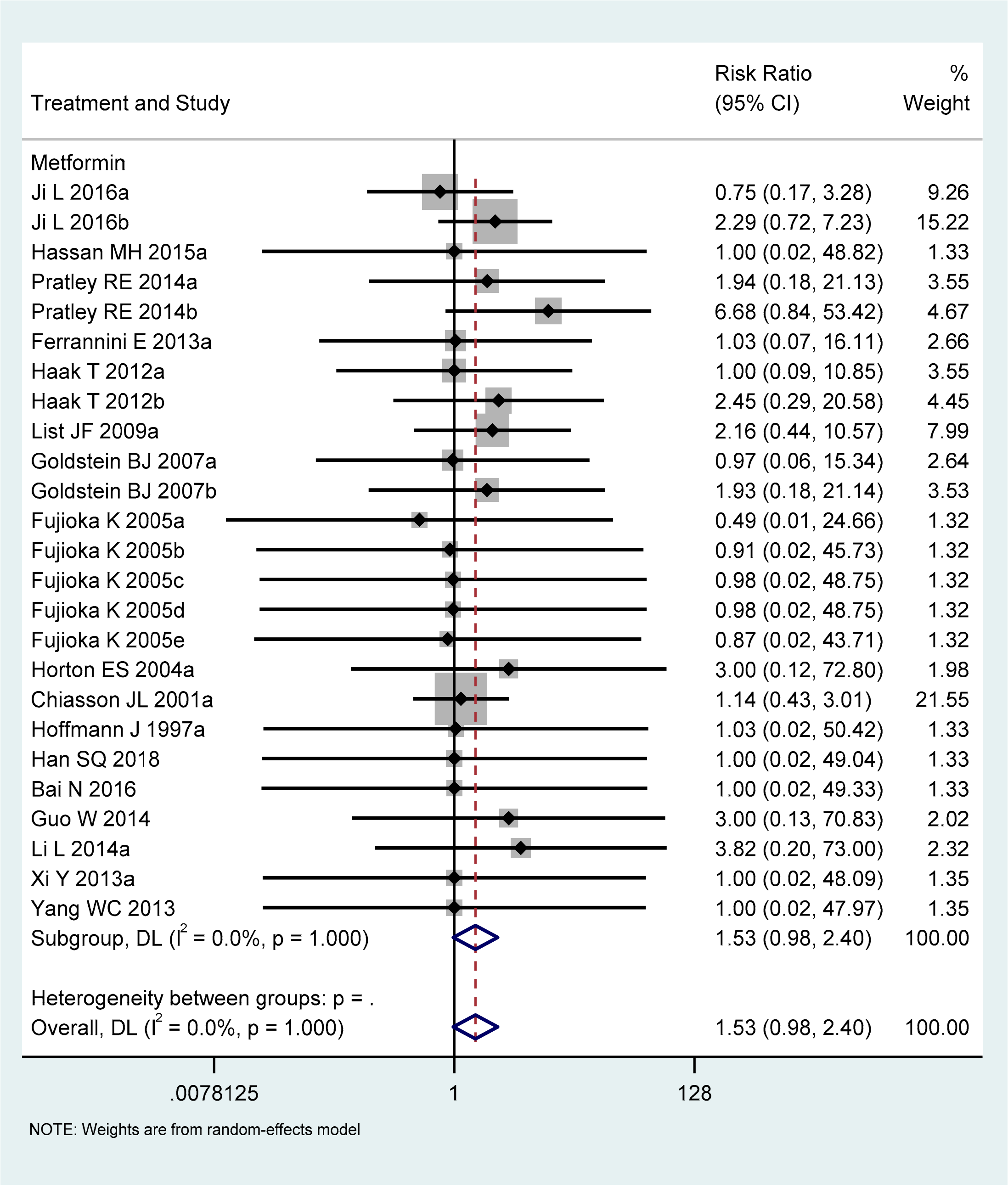

Figure S50. Meta-analysis results for incidence of hypoglycemia of metformin vs placebo/lifestyle intervention

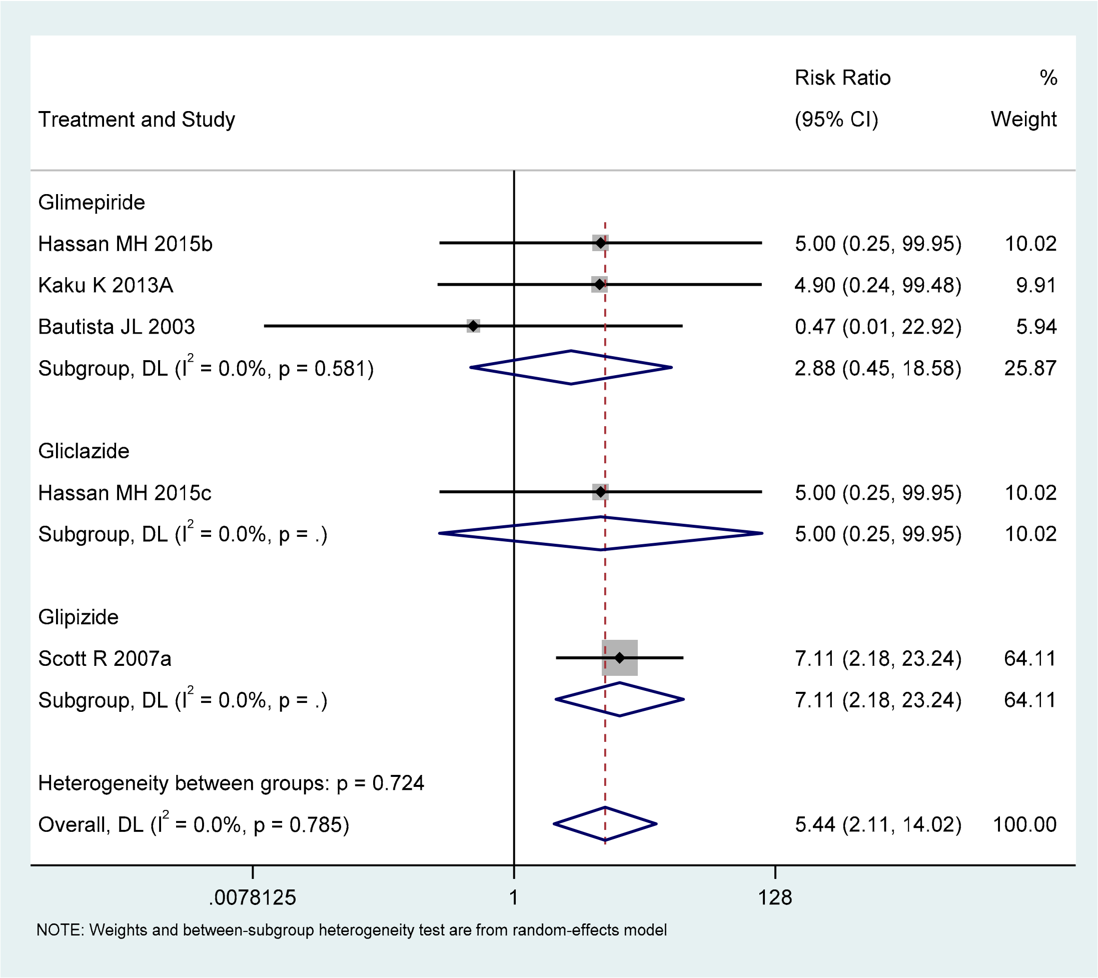

Figure S51. Meta-analysis results for incidence of hypoglycemia of sulfonylureas vs placebo/lifestyle intervention

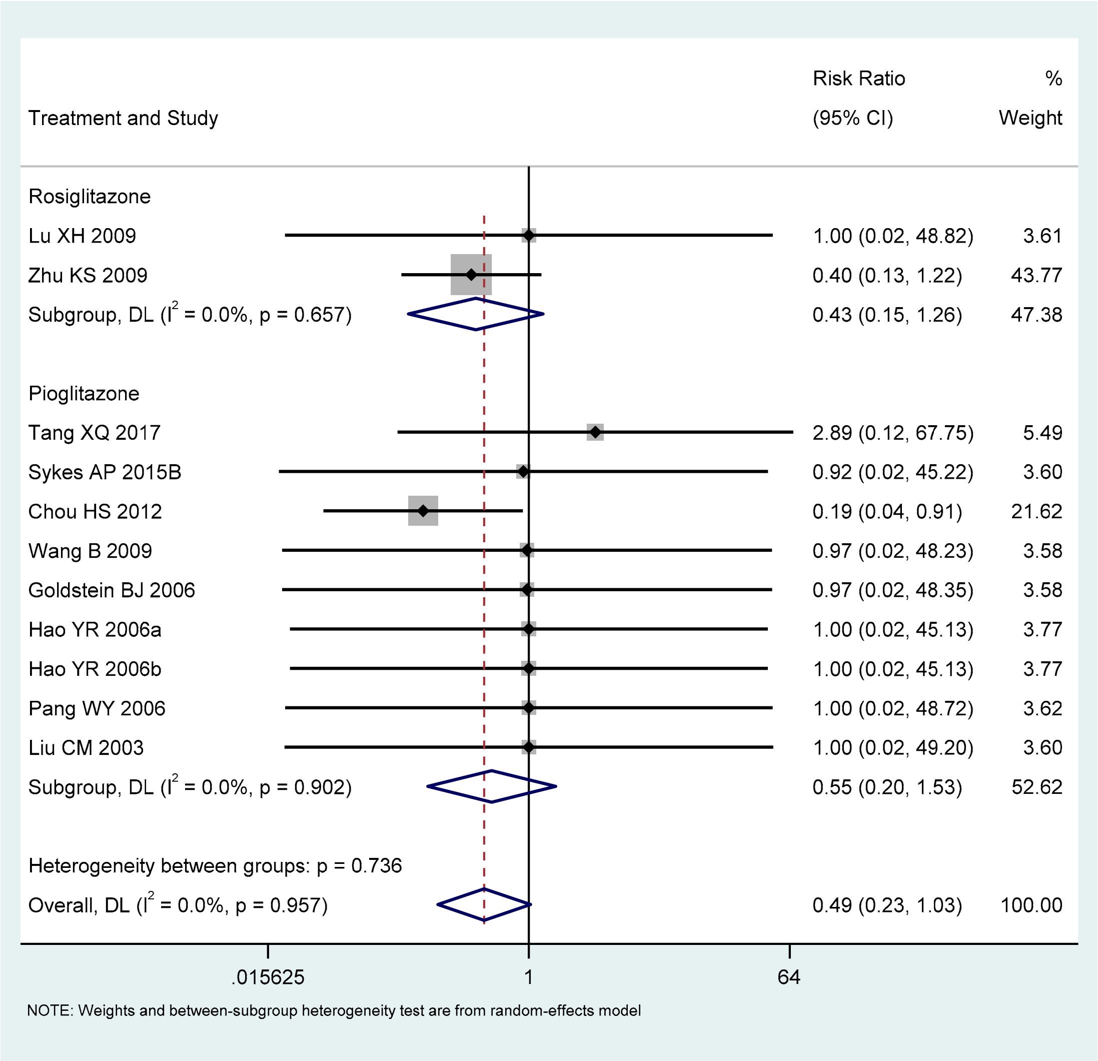

Figure S52. Meta-analysis results for incidence of hypoglycemia of thiazolidinediones vs placebo/lifestyle intervention

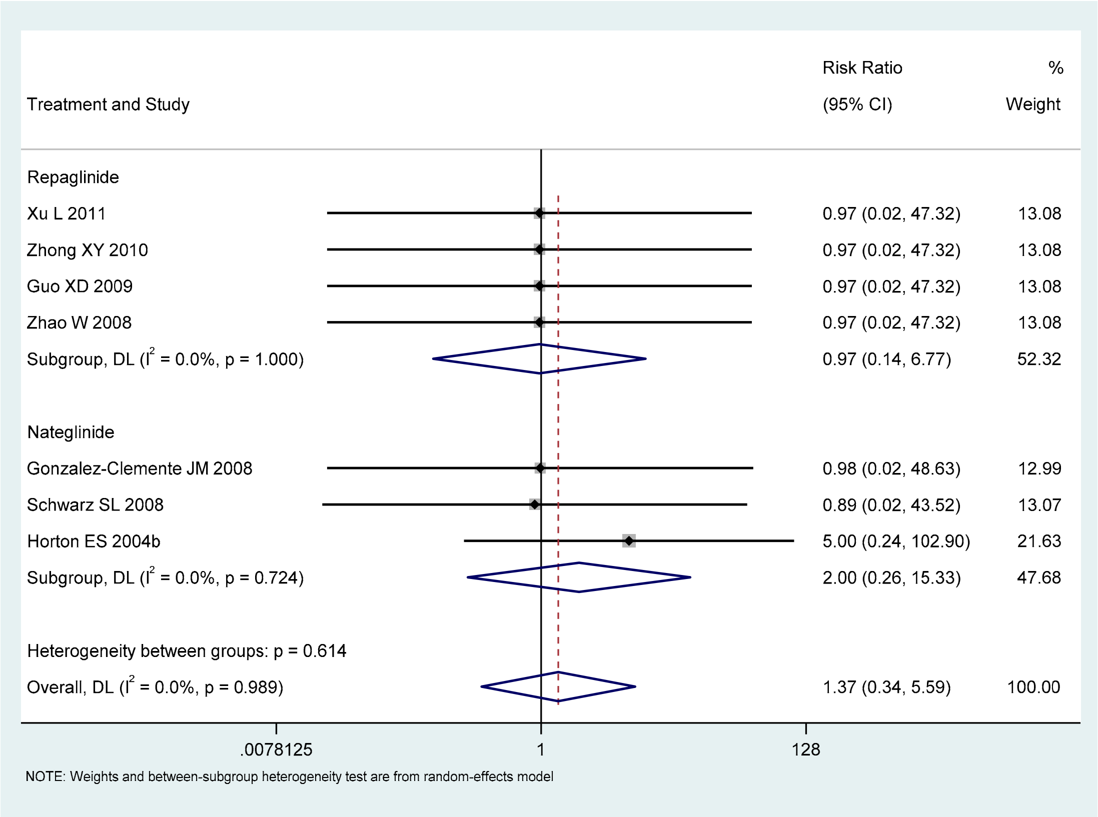

Figure S53. Meta-analysis results for incidence of hypoglycemia of glinides vs placebo/lifestyle intervention

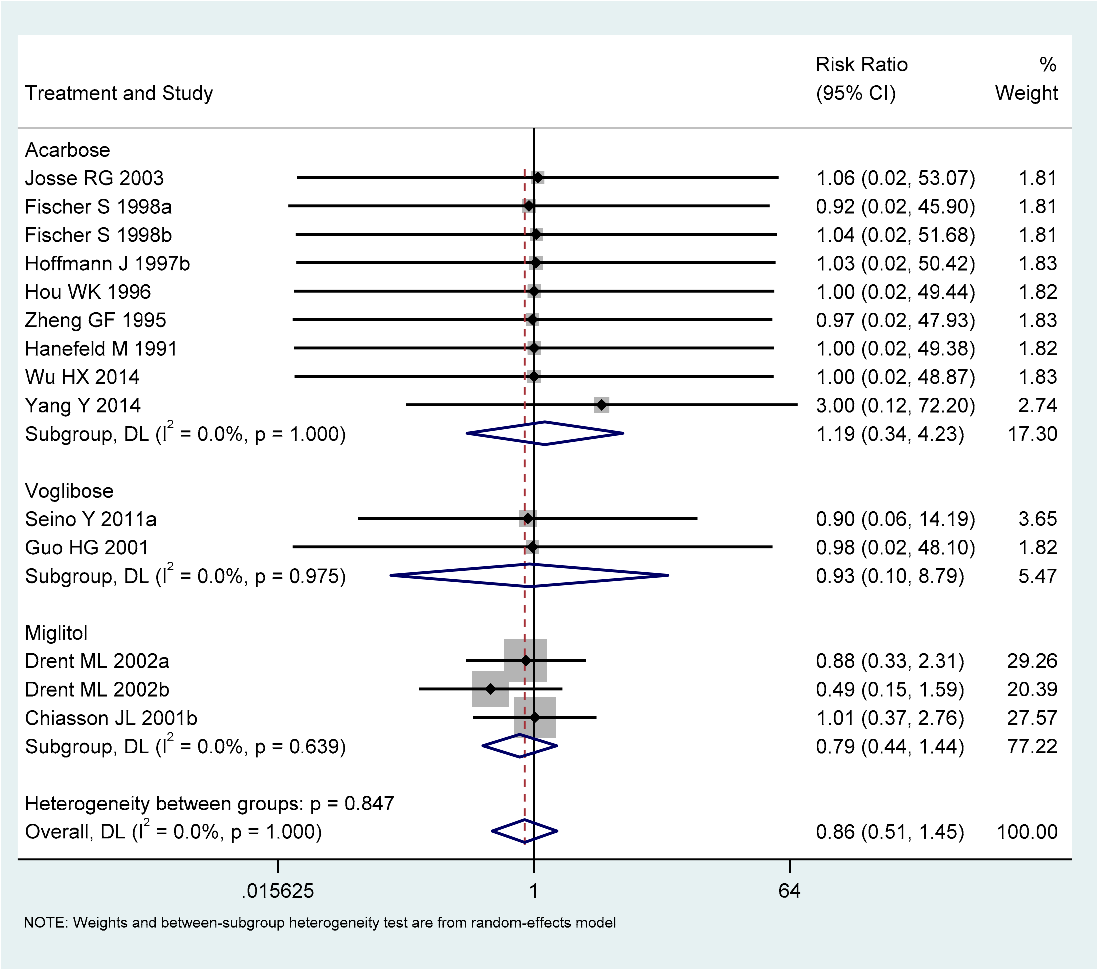

Figure S54. Meta-analysis results for incidence of hypoglycemia of α-glucosidase inhibitors vs placebo/lifestyle intervention

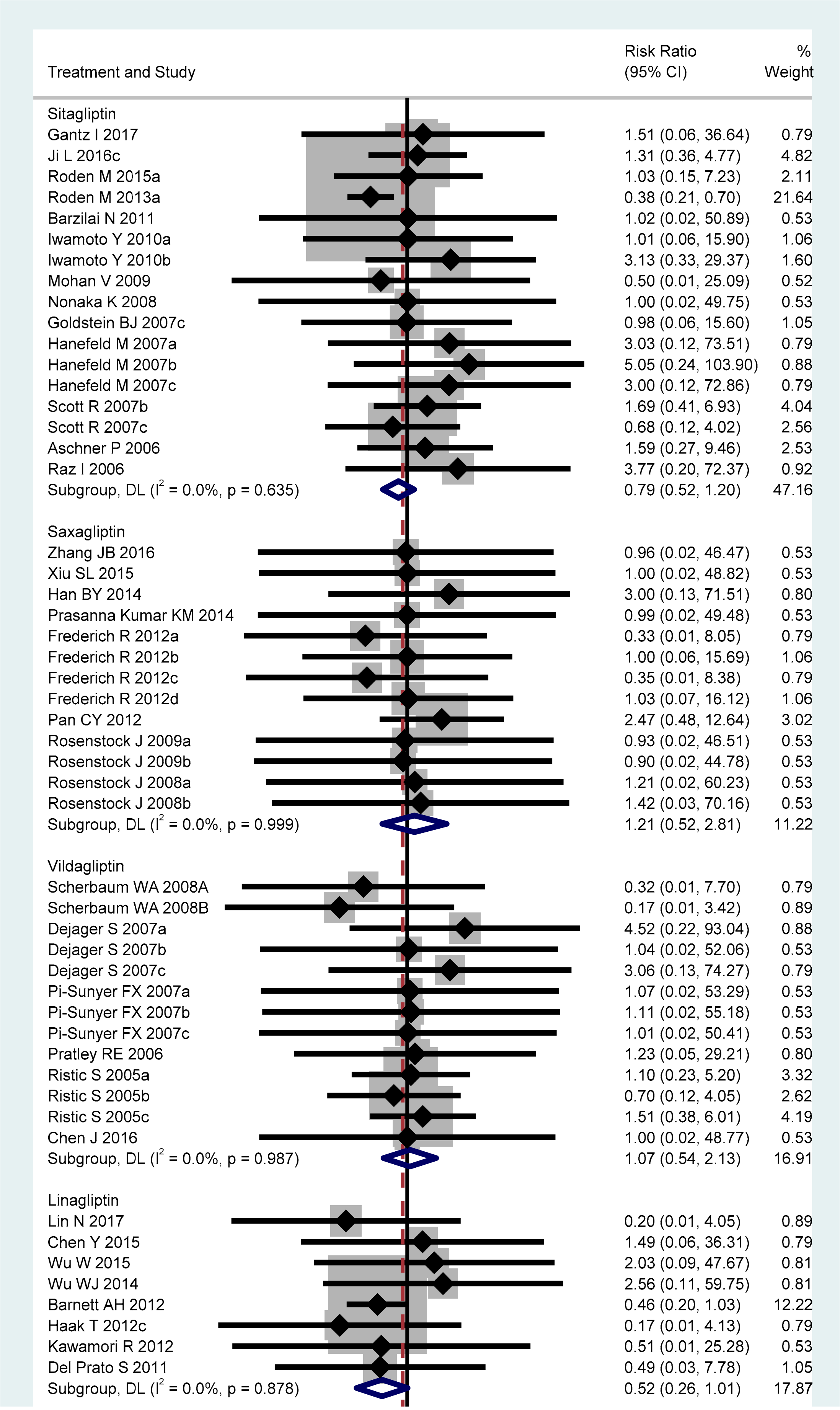
Treatment and Study
Risk Ratio (95% CI)
% Weight
Sitagliptin
Gantz I 2017 1.51 (0.06, 36.64) 0.79
Ji L 2016c 1.31 (0.36, 4.77) 4.82
Roden M 2015a 1.03 (0.15, 7.23) 2.11
Roden M 2013a 0.38 (0.21, 0.70) 21.64
Barzilai N 2011 1.02 (0.02, 50.89) 0.53
Iwamoto Y 2010a 1.01 (0.06, 15.90) 1.06
Iwamoto Y 2010b 3.13 (0.33, 29.37) 1.60
Mohan V 2009 0.50 (0.01, 25.09) 0.52
Nonaka K 2008 1.00 (0.02, 49.75) 0.53
Goldstein BJ 2007c 0.98 (0.06, 15.60) 1.05
Hanefeld M 2007a 3.03 (0.12, 73.51) 0.79
Hanefeld M 2007b 5.05 (0.24, 103.90) 0.88
Hanefeld M 2007c 3.00 (0.12, 72.86) 0.79
Scott R 2007b 1.69 (0.41, 6.93) 4.04
Scott R 2007c 0.68 (0.12, 4.02) 2.56
Aschner P 2006 1.59 (0.27, 9.46) 2.53
Raz I 2006 3.77 (0.20, 72.37) 0.92
Subgroup, DL (I2 = 0.0%, p = 0.635) 0.79 (0.52, 1.20) 47.16
Saxagliptin
Zhang JB 2016 0.96 (0.02, 46.47) 0.53
Xiu SL 2015 1.00 (0.02, 48.82) 0.53
Han BY 2014 3.00 (0.13, 71.51) 0.80
Prasanna Kumar KM 2014 0.99 (0.02, 49.48) 0.53
Frederich R 2012a 0.33 (0.01, 8.05) 0.79
Frederich R 2012b 1.00 (0.06, 15.69) 1.06
Frederich R 2012c 0.35 (0.01, 8.38) 0.79
Frederich R 2012d 1.03 (0.07, 16.12) 1.06
Pan CY 2012 2.47 (0.48, 12.64) 3.02
Rosenstock J 2009a 0.93 (0.02, 46.51) 0.53
Rosenstock J 2009b 0.90 (0.02, 44.78) 0.53
Rosenstock J 2008a 1.21 (0.02, 60.23) 0.53
Rosenstock J 2008b 1.42 (0.03, 70.16) 0.53
Subgroup, DL (I2 = 0.0%, p = 0.999) 1.21 (0.52, 2.81) 11.22
Vildagliptin
Scherbaum WA 2008A 0.32 (0.01, 7.70) 0.79
Scherbaum WA 2008B 0.17 (0.01, 3.42) 0.89
Dejager S 2007a 4.52 (0.22, 93.04) 0.88
Dejager S 2007b 1.04 (0.02, 52.06) 0.53
Dejager S 2007c 3.06 (0.13, 74.27) 0.79
Pi-Sunyer FX 2007a 1.07 (0.02, 53.29) 0.53
Pi-Sunyer FX 2007b 1.11 (0.02, 55.18) 0.53
Pi-Sunyer FX 2007c 1.01 (0.02, 50.41) 0.53
Pratley RE 2006 1.23 (0.05, 29.21) 0.80
Ristic S 2005a 1.10 (0.23, 5.20) 3.32
Ristic S 2005b 0.70 (0.12, 4.05) 2.62
Ristic S 2005c 1.51 (0.38, 6.01) 4.19
Chen J 2016 1.00 (0.02, 48.77) 0.53
Subgroup, DL (I2 = 0.0%, p = 0.987) 1.07 (0.54, 2.13) 16.91
Linagliptin
Lin N 2017 0.20 (0.01, 4.05) 0.89
Chen Y 2015 1.49 (0.06, 36.31) 0.79
Wu W 2015 2.03 (0.09, 47.67) 0.81
Wu WJ 2014 2.56 (0.11, 59.75) 0.81
Barnett AH 2012 0.46 (0.20, 1.03) 12.22
Haak T 2012c 0.17 (0.01, 4.13) 0.79
Kawamori R 2012 0.51 (0.01, 25.28) 0.53
Del Prato S 2011 0.49 (0.03, 7.78) 1.05
Subgroup, DL (I2 = 0.0%, p = 0.878) 0.52 (0.26, 1.01) 17.87

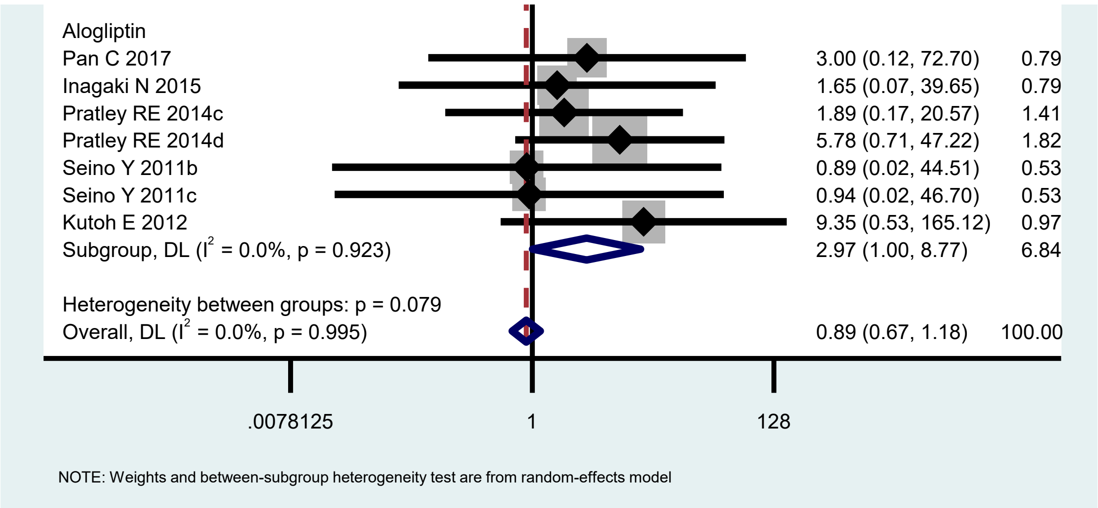

Figure S55. Meta-analysis results for incidence of hypoglycemia of dipeptidyl peptidase-4 inhibitors vs placebo/lifestyle intervention

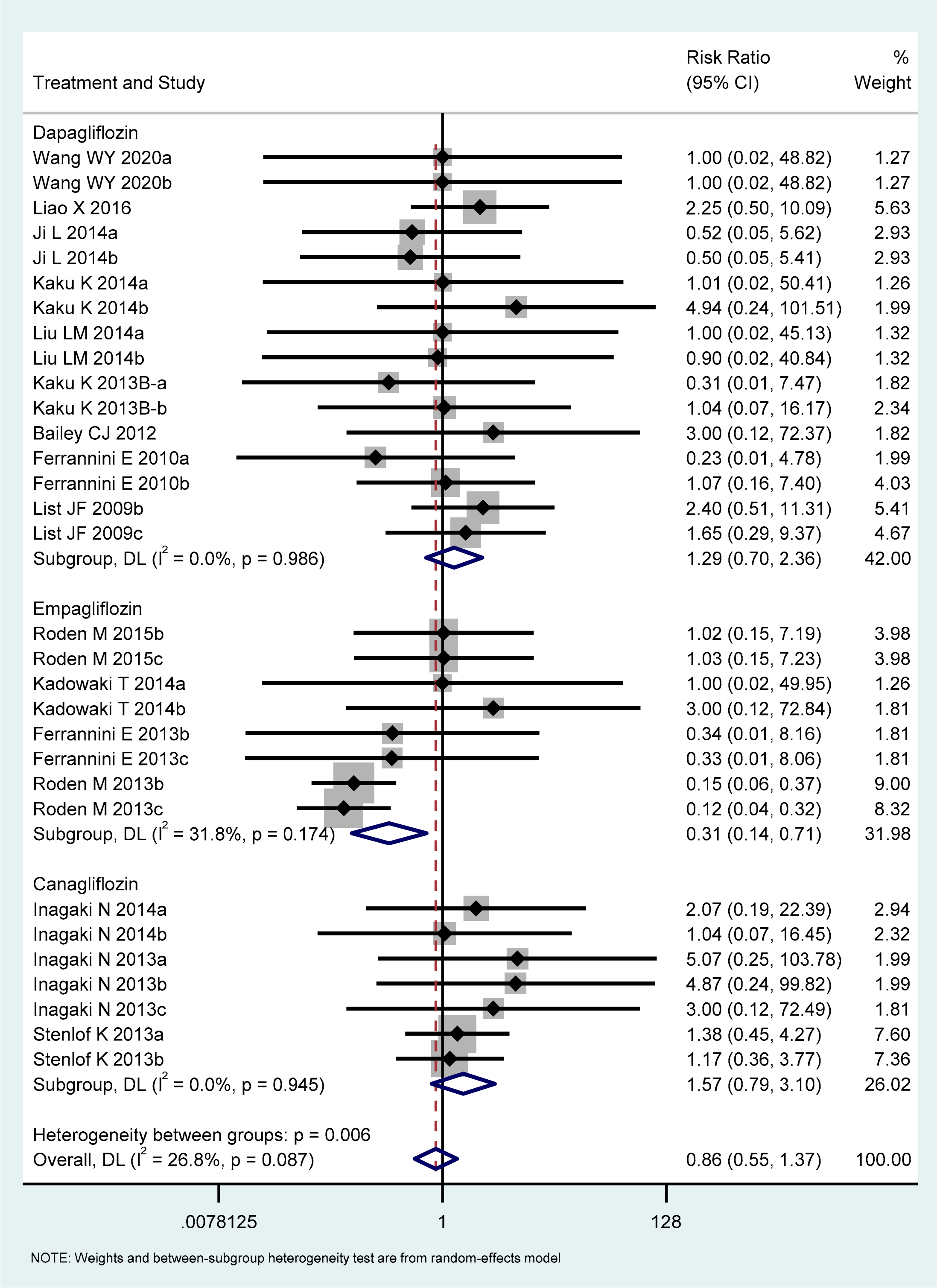

Figure S56. Meta-analysis results for incidence of hypoglycemia of sodium-glucose cotransporter-2 inhibitors vs placebo/lifestyle intervention

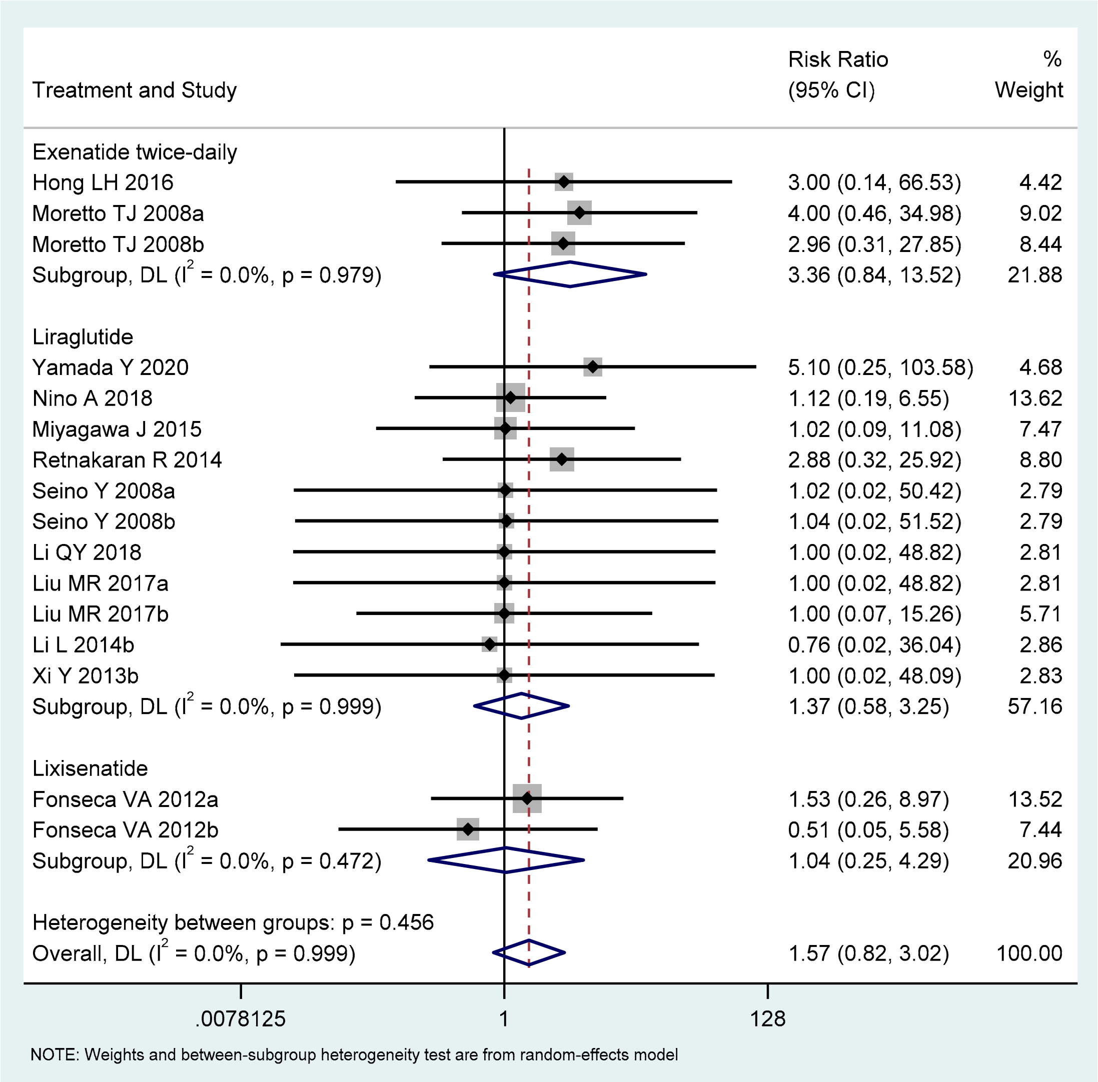

Figure S57. Meta-analysis results for incidence of hypoglycemia of glucagon-like peptide-1 receptor agonists vs placebo/lifestyle intervention

【Death】

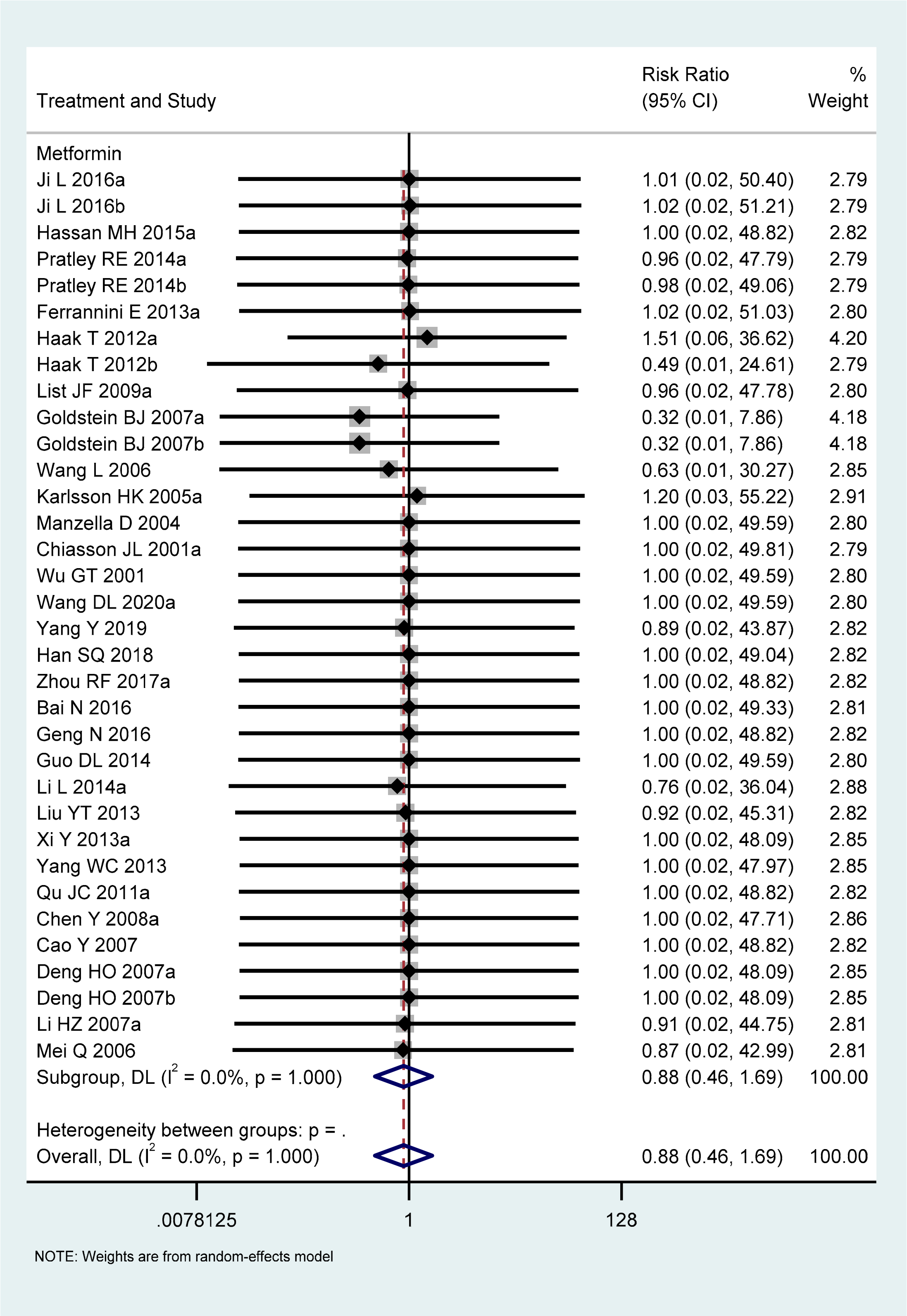

Figure S58. Meta-analysis results for incidence of death of metformin vs placebo/lifestyle intervention

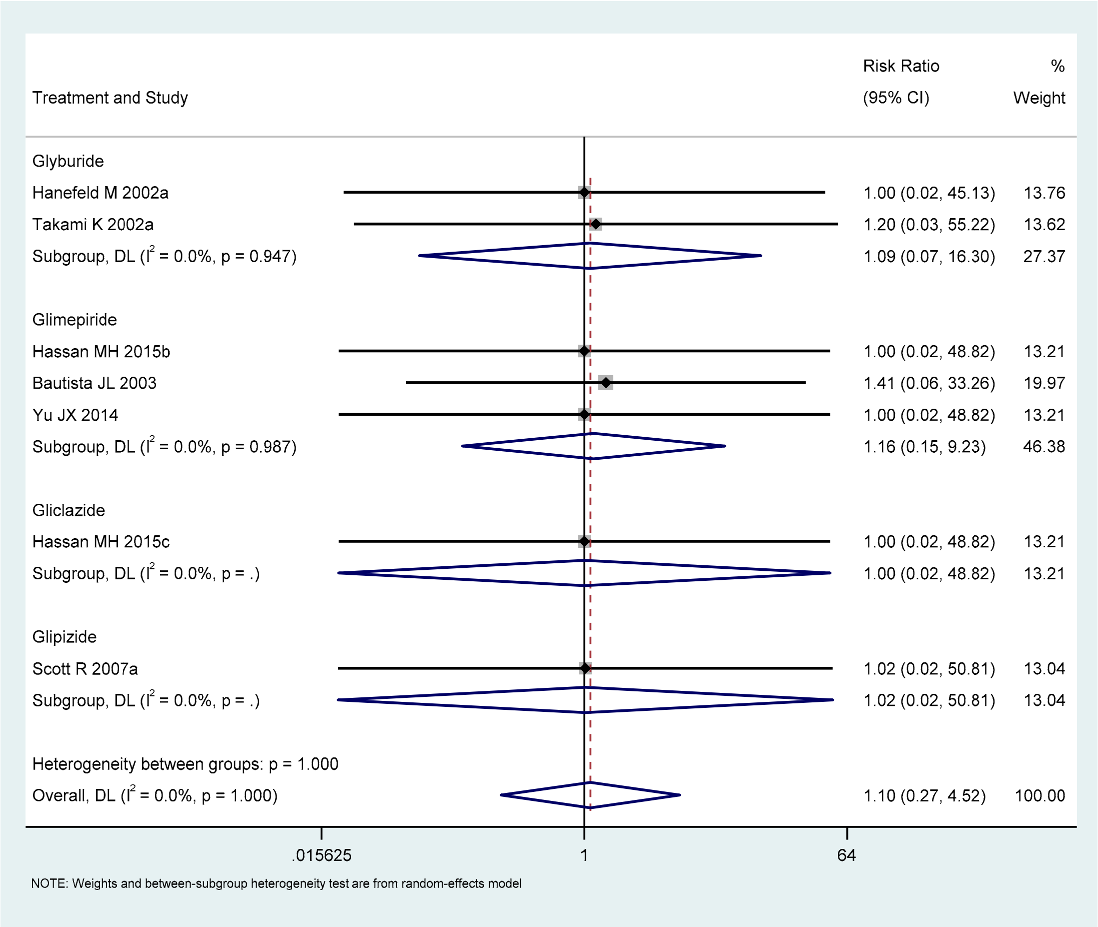

Figure S59. Meta-analysis results for incidence of death of sulfonylureas vs placebo/lifestyle intervention

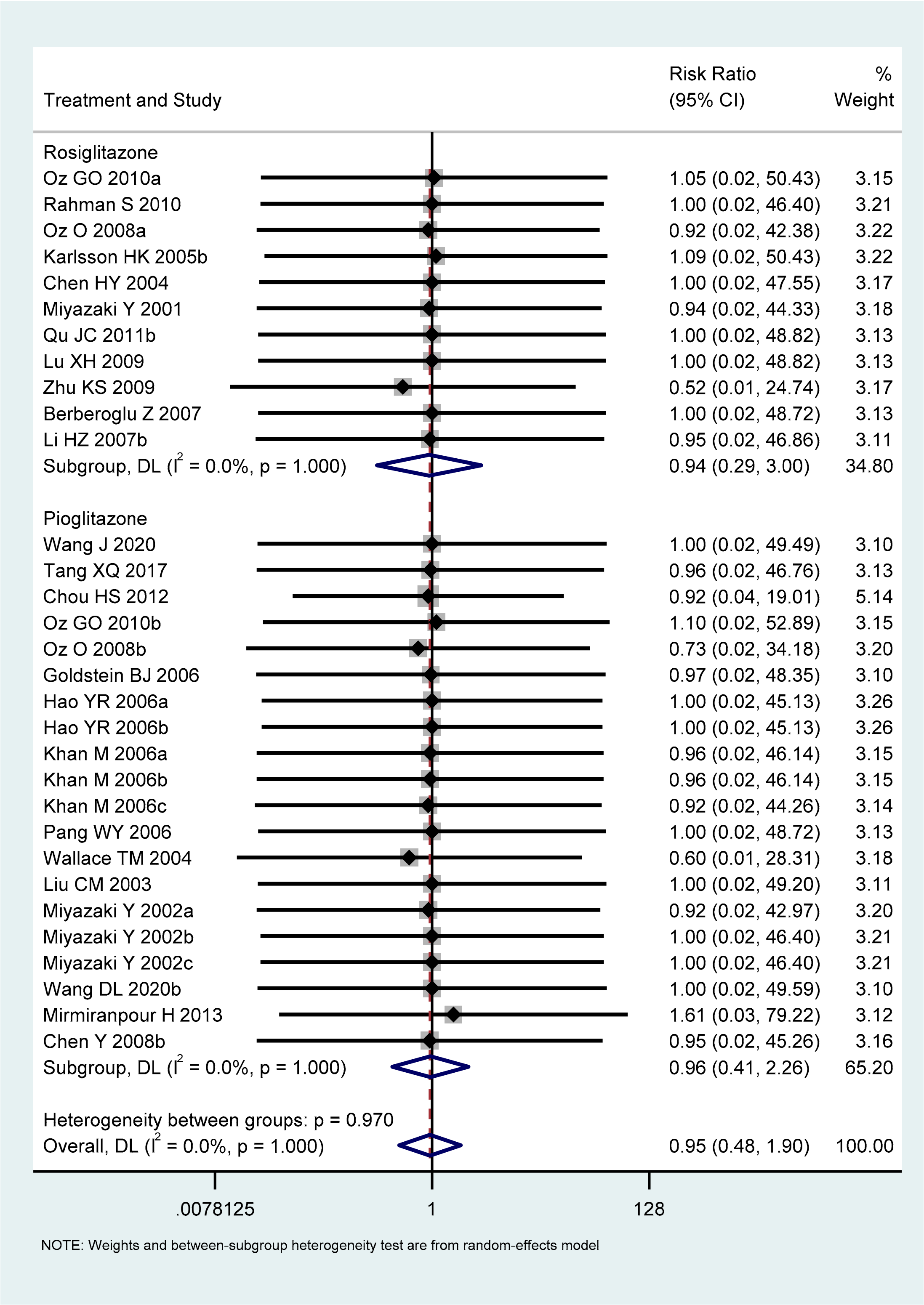

Figure S60. Meta-analysis results for incidence of death of thiazolidinediones vs placebo/lifestyle intervention

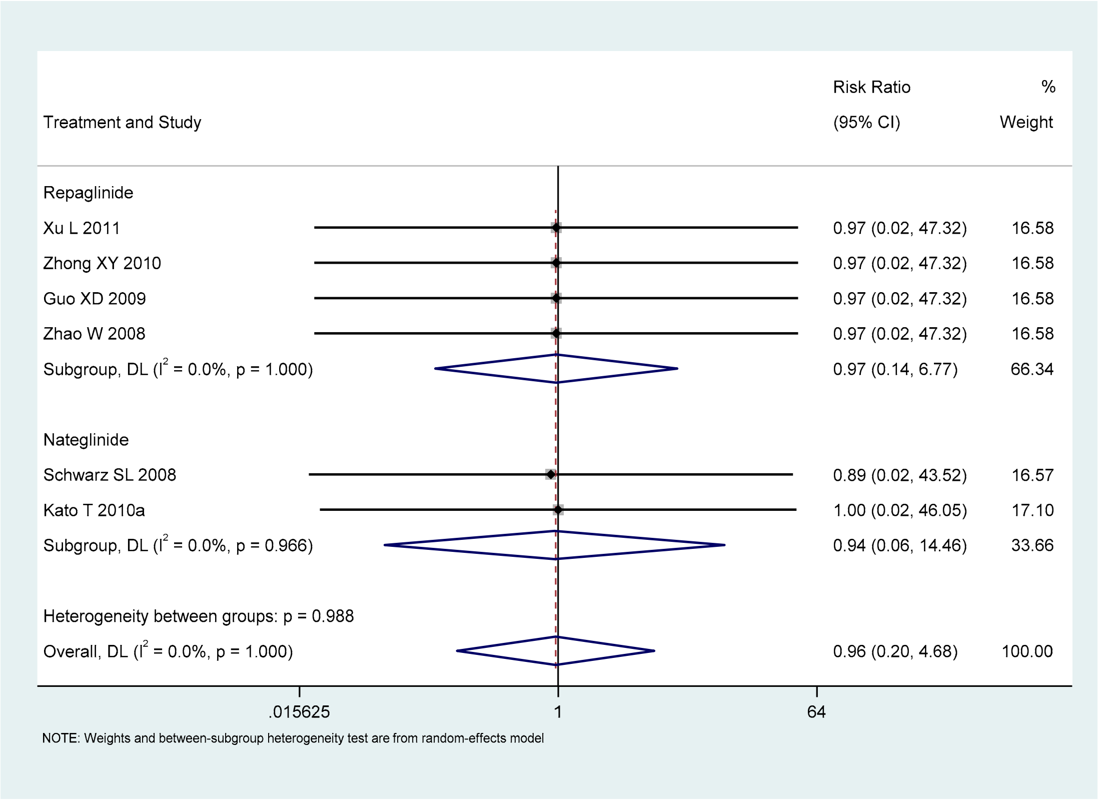

Figure S61. Meta-analysis results for incidence of death of glinides vs placebo/lifestyle intervention

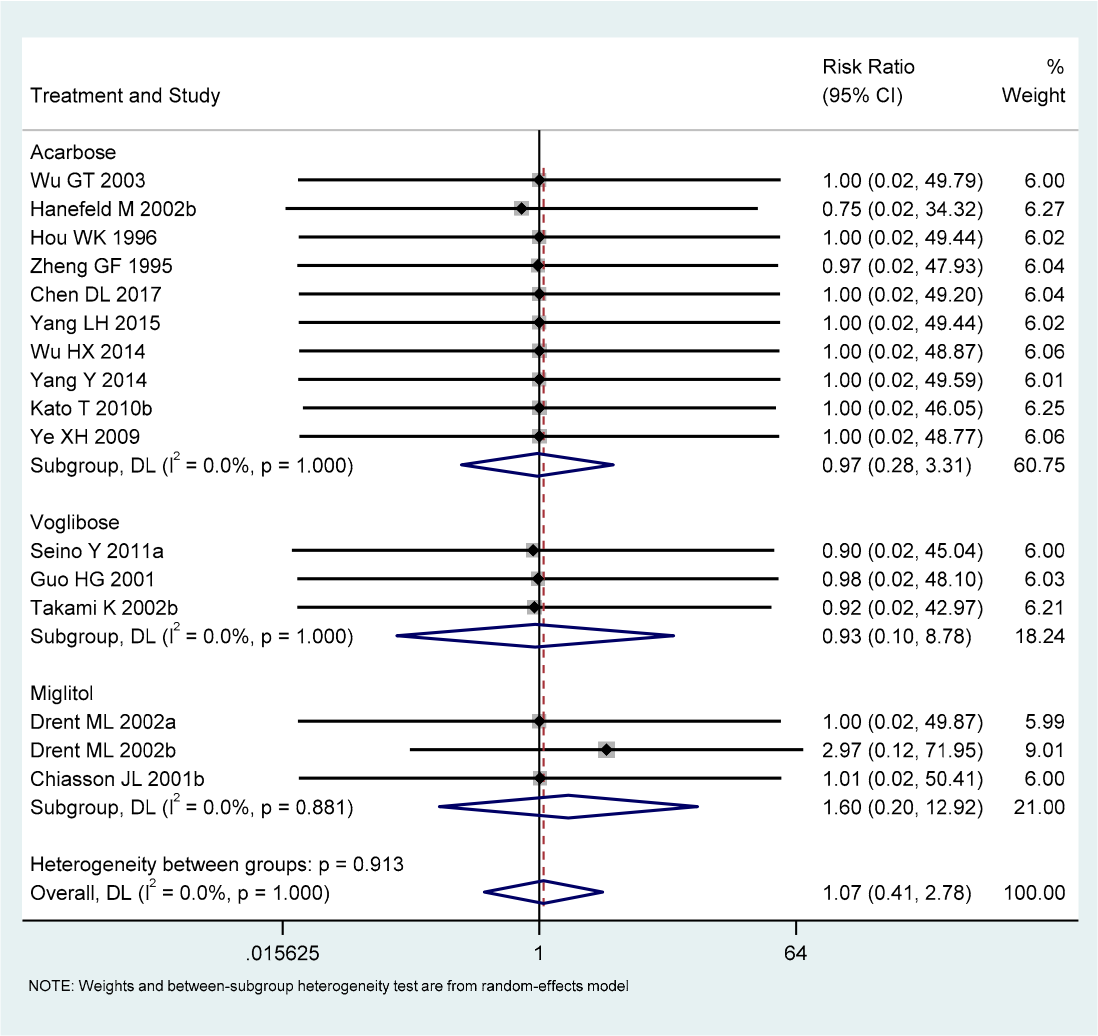

Figure S62. Meta-analysis results for incidence of death of α-glucosidase inhibitors vs placebo/lifestyle intervention

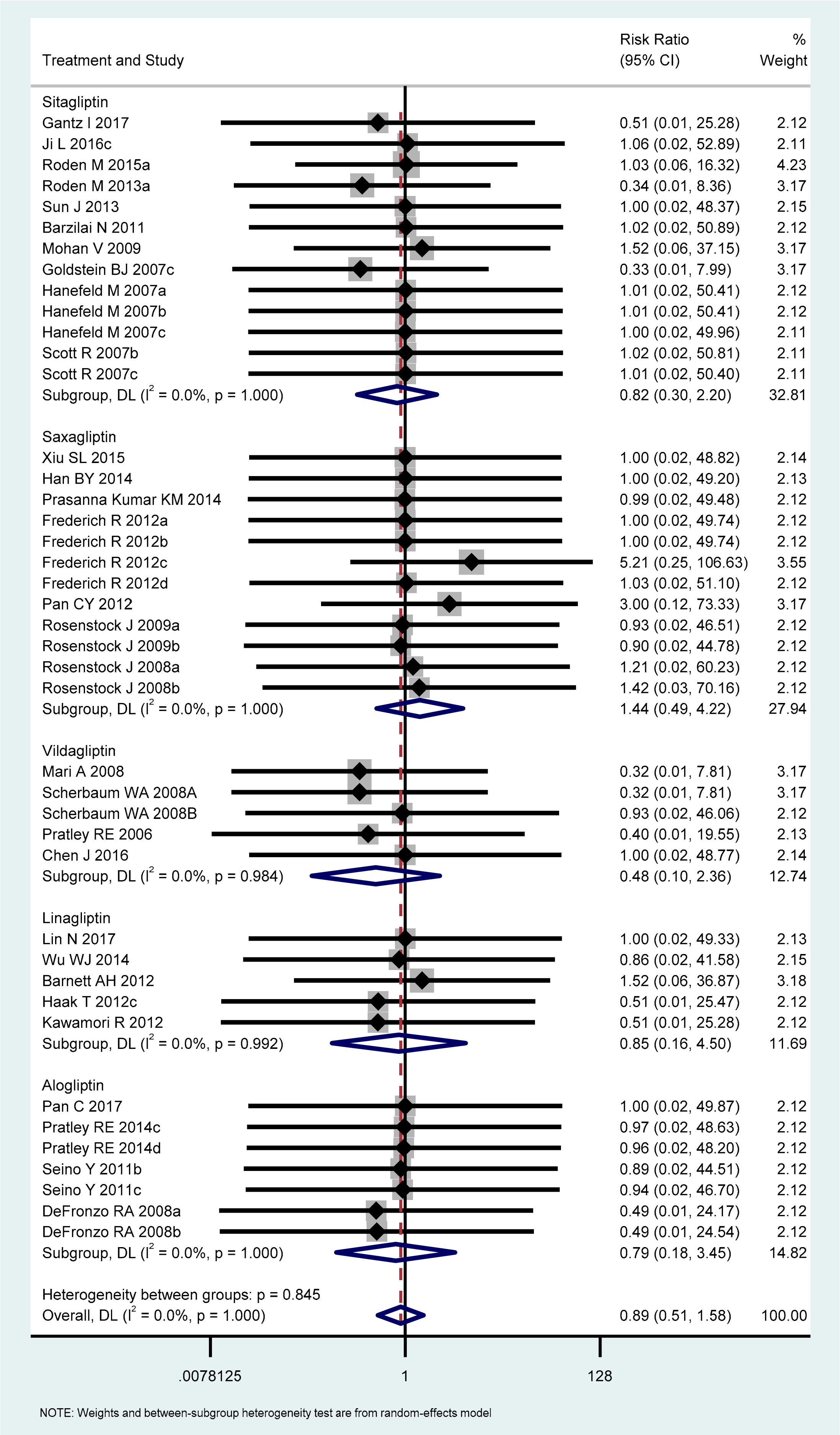

Figure S63. Meta-analysis results for incidence of death of dipeptidyl peptidase-4 inhibitors vs placebo/lifestyle intervention

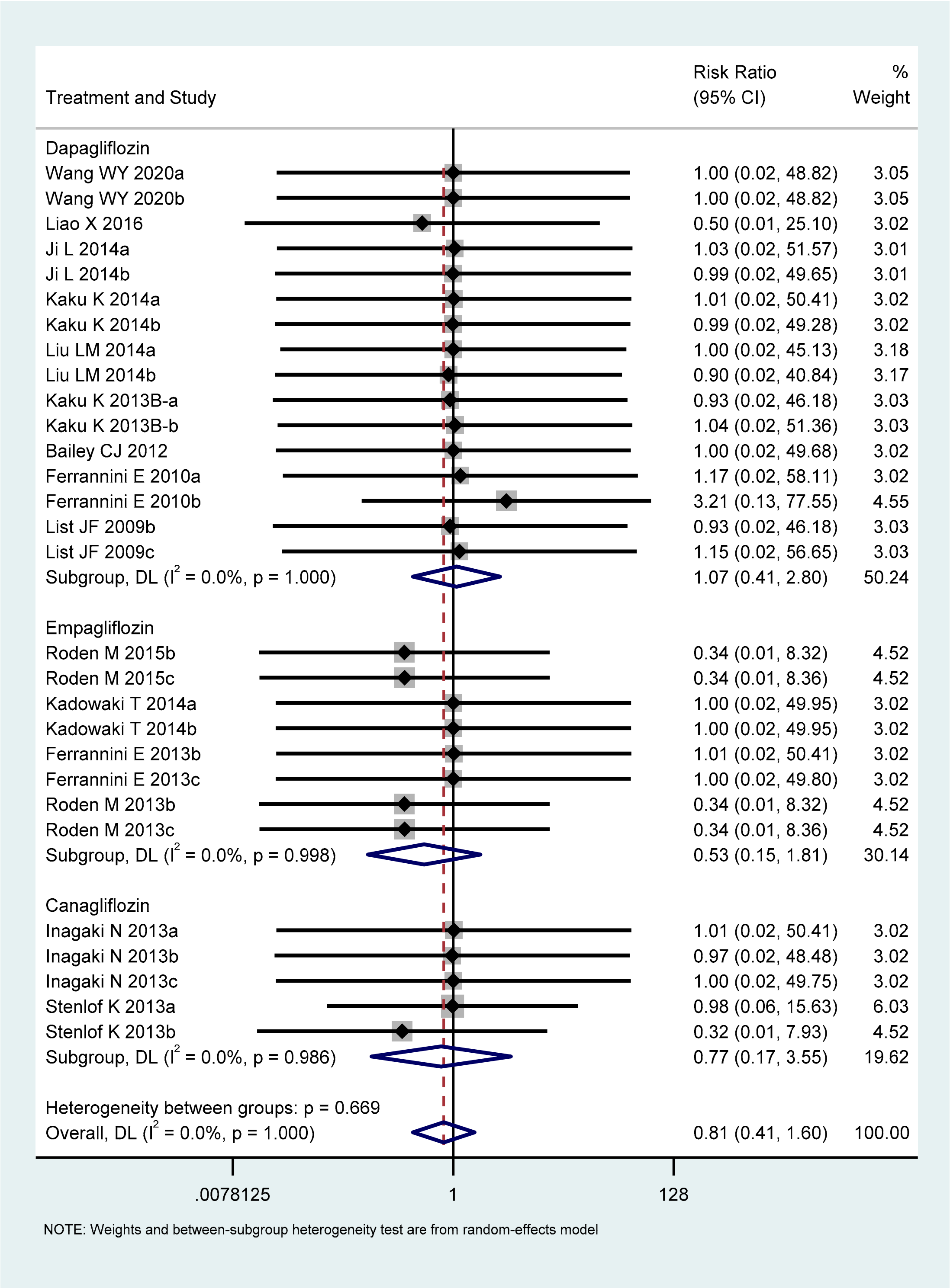

Figure S64. Meta-analysis results for incidence of death of sodium-glucose cotransporter-2 inhibitors vs placebo/lifestyle intervention

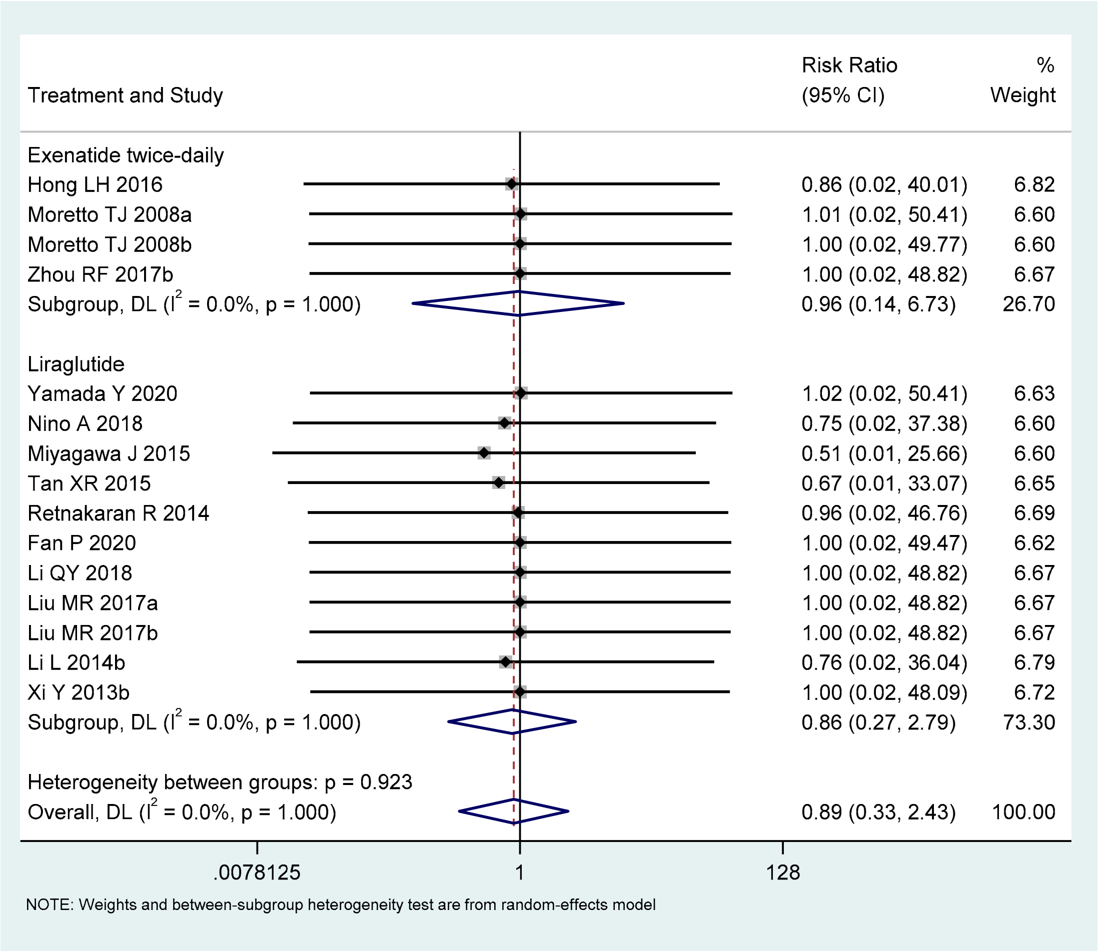

Figure S65. Meta-analysis results for incidence of death of glucagon-like peptide-1 receptor agonists vs placebo/lifestyle intervention

【Total vascular events】

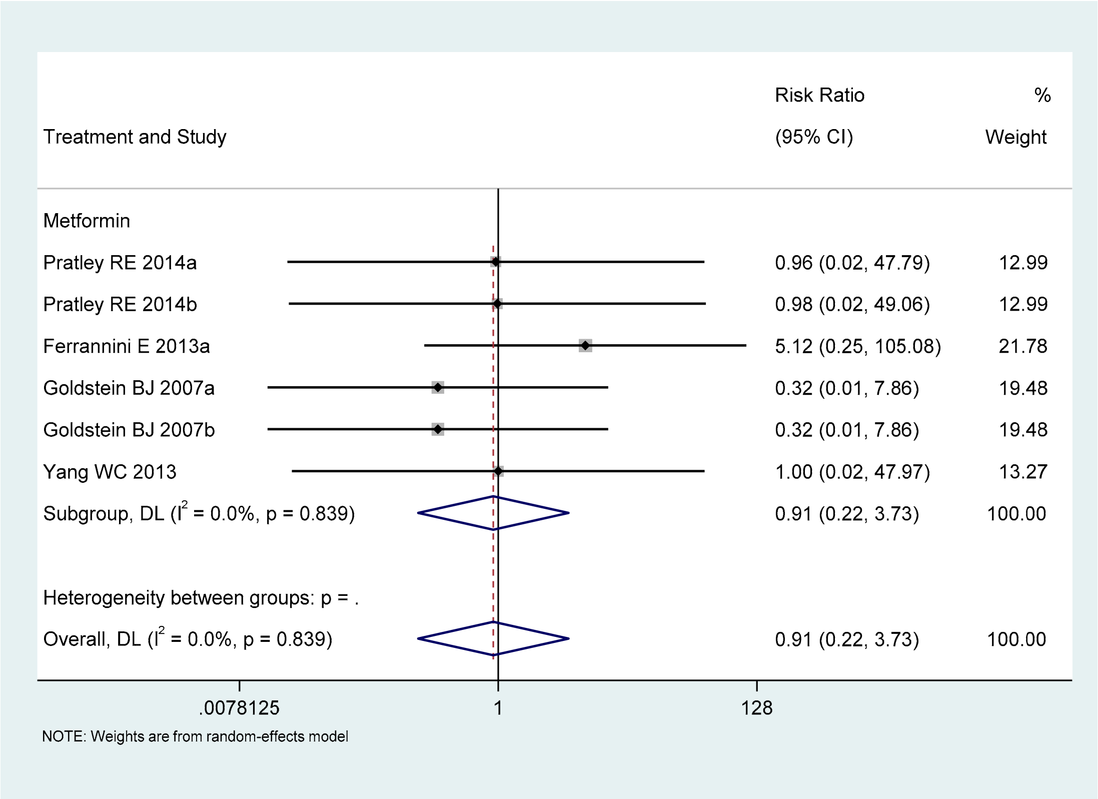

Figure S66. Meta-analysis results for incidence of total vascular events of metformin vs placebo/lifestyle intervention

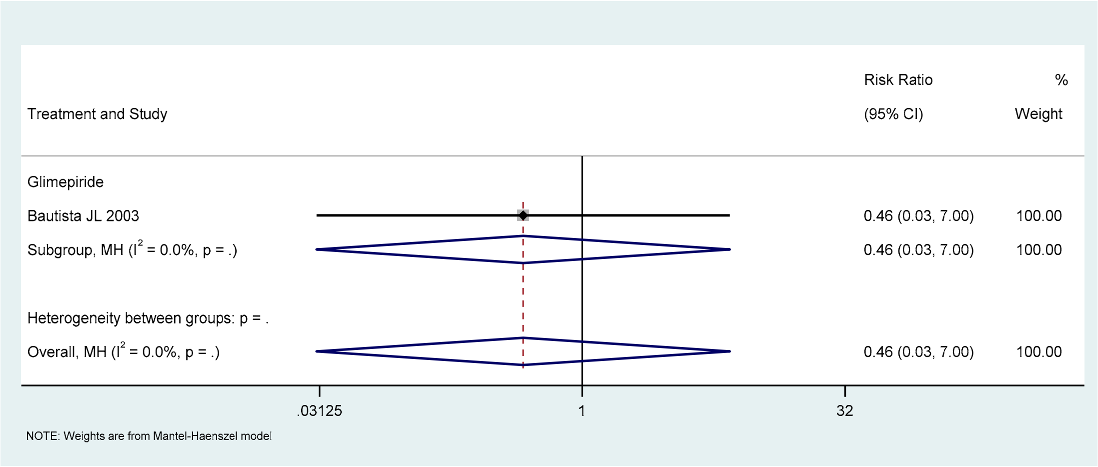

Figure S67. Meta-analysis results for incidence of total vascular events of sulfonylureas vs placebo/lifestyle intervention

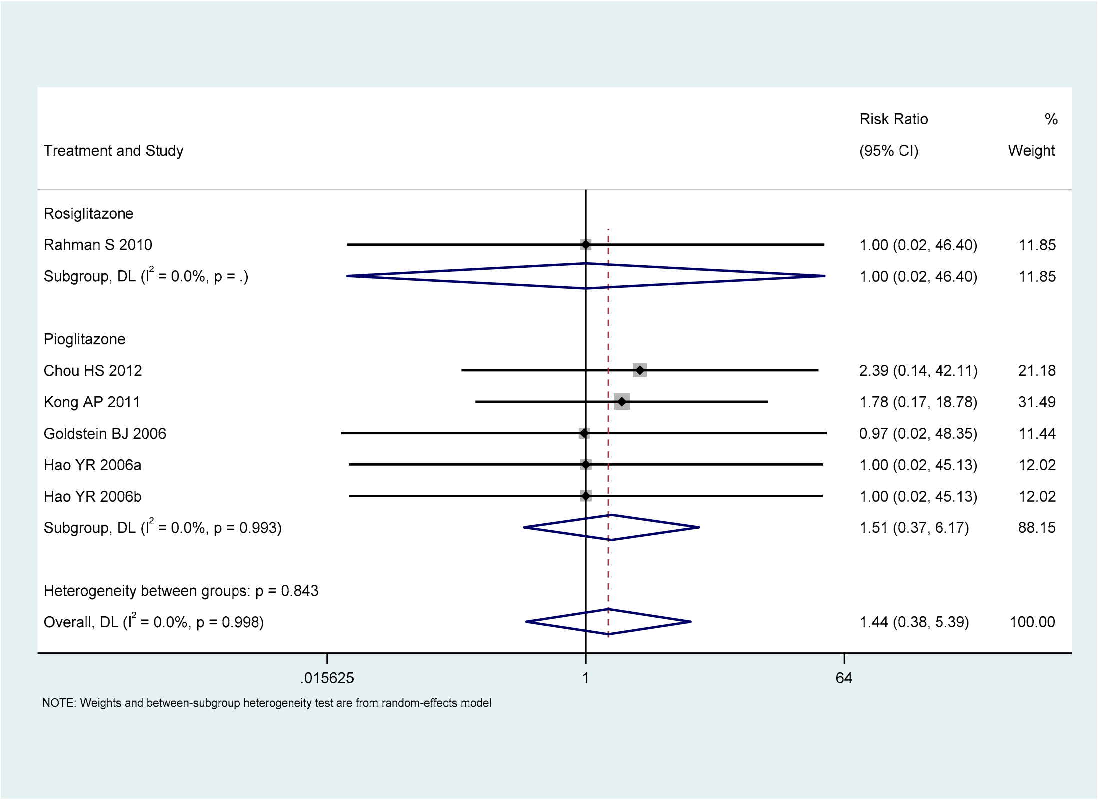

Figure S68. Meta-analysis results for incidence of total vascular events of thiazolidinediones vs placebo/lifestyle intervention

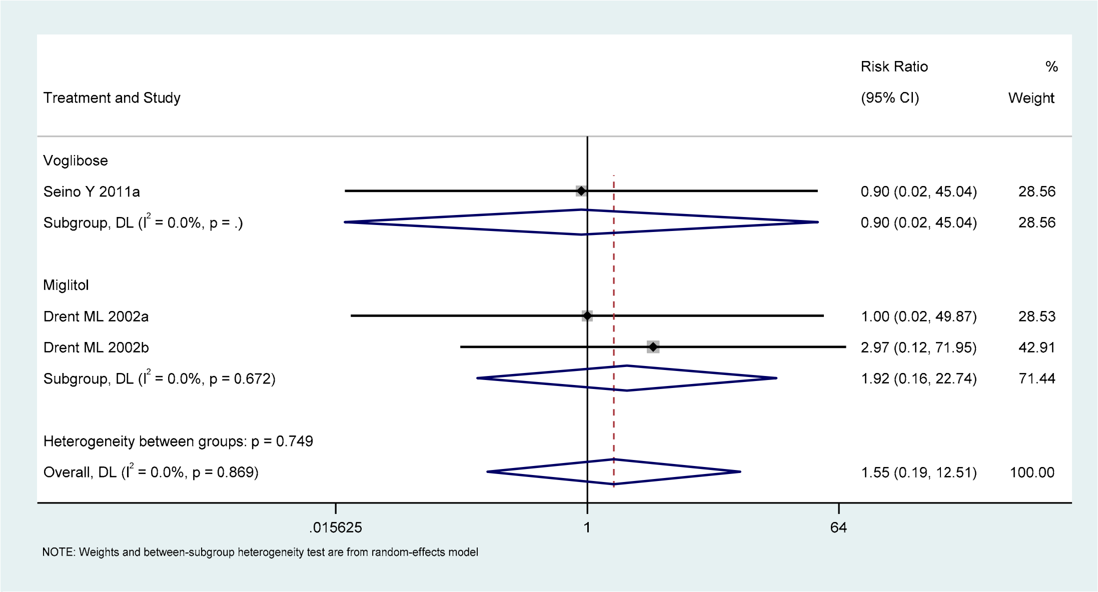

Figure S69. Meta-analysis results for incidence of total vascular events of α-glucosidase inhibitors vs placebo/lifestyle intervention

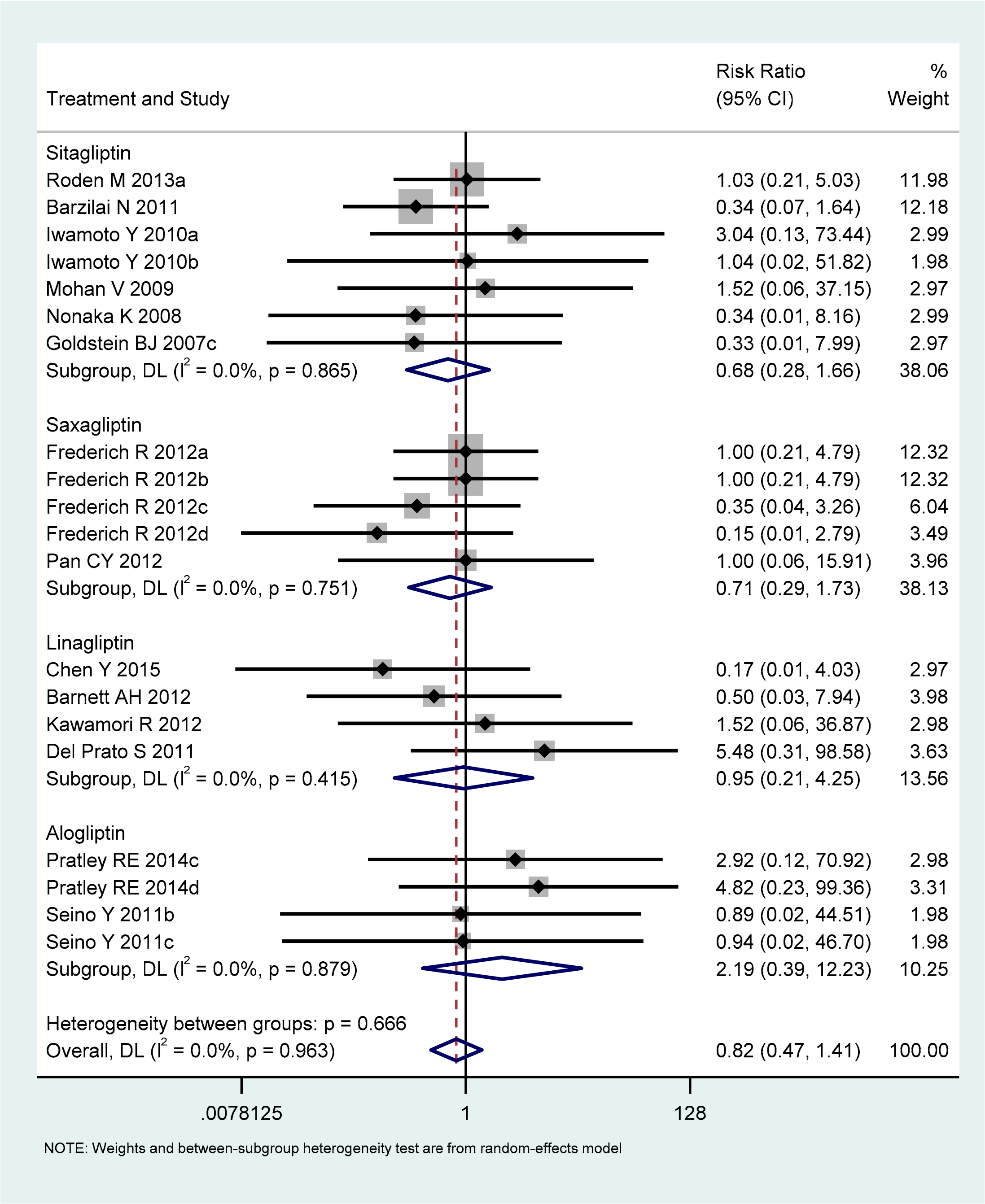

Figure S70. Meta-analysis results for incidence of total vascular events of dipeptidyl peptidase-4 inhibitors vs placebo/lifestyle intervention

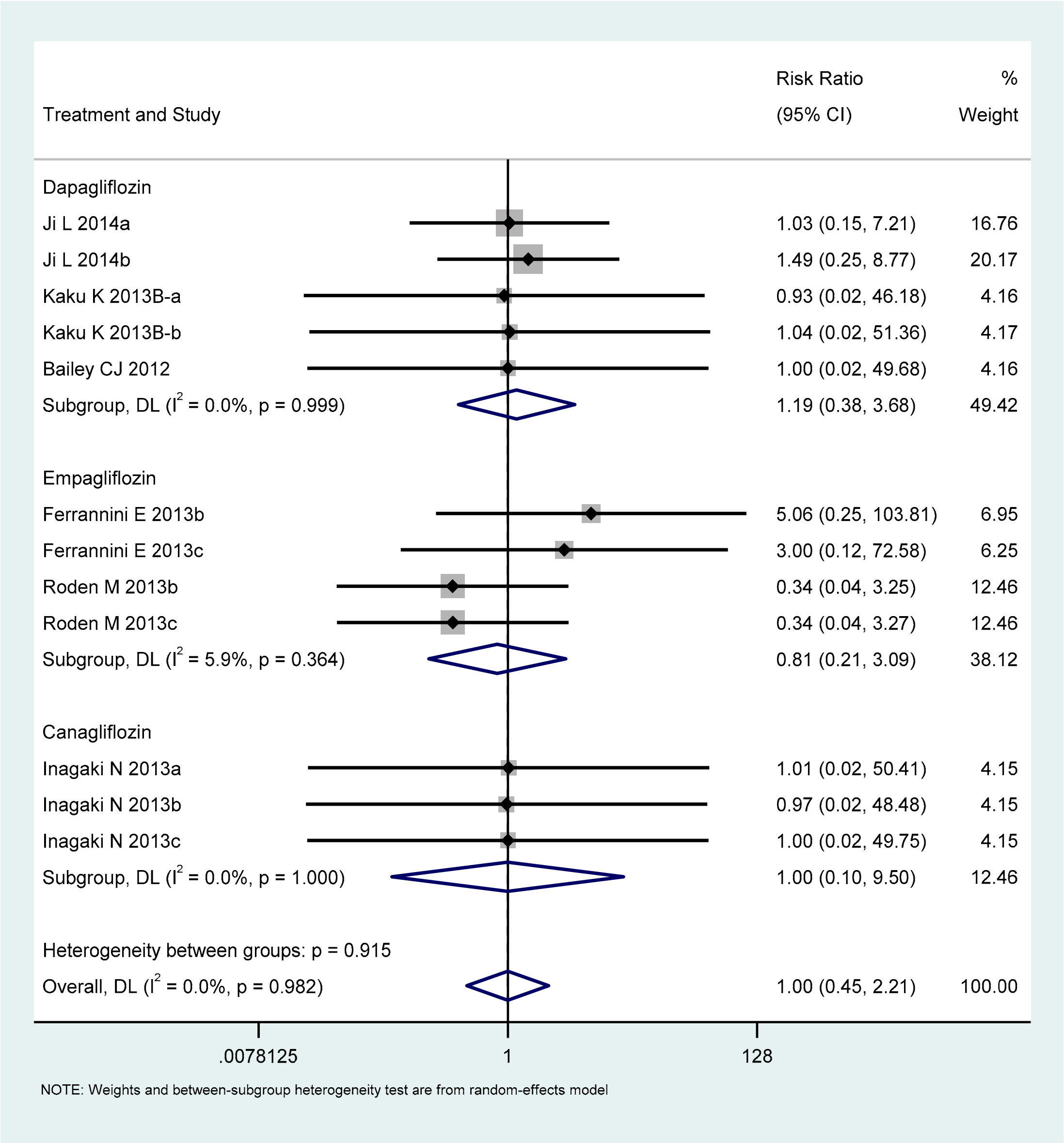

Figure S71. Meta-analysis results for incidence of total vascular events of sodium-glucose cotransporter-2 inhibitors vs placebo/lifestyle intervention

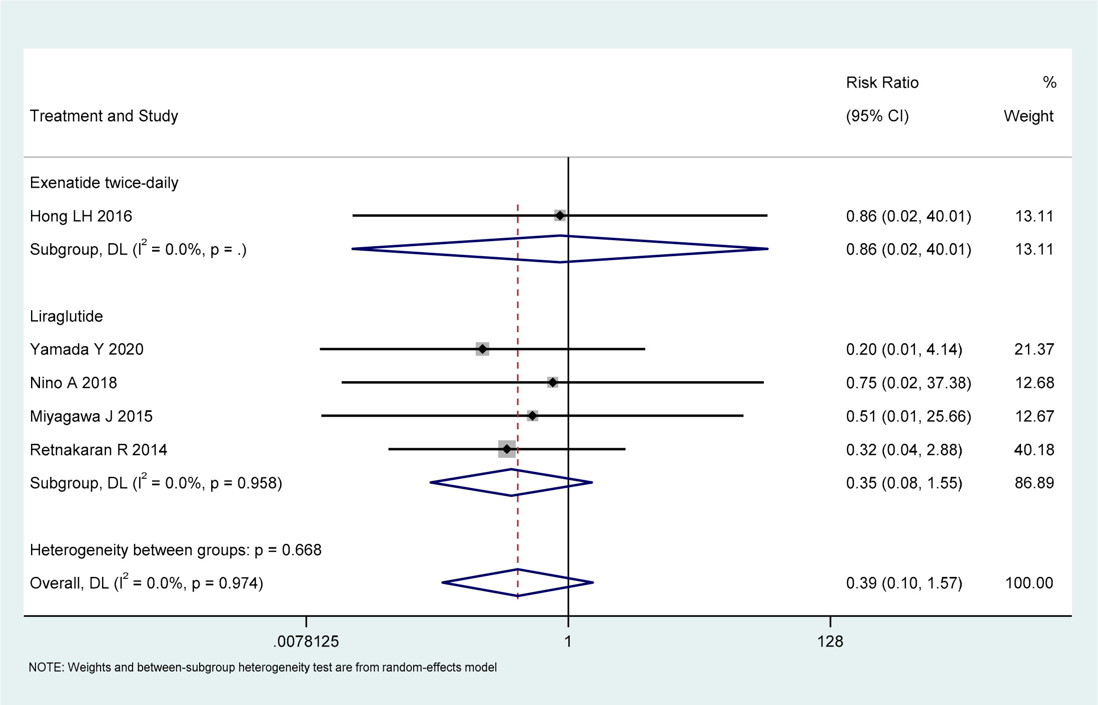

Figure S72. Meta-analysis results for incidence of total vascular events of glucagon-like peptide-1 receptor agonists vs placebo/lifestyle intervention

【Myocardial infarction】

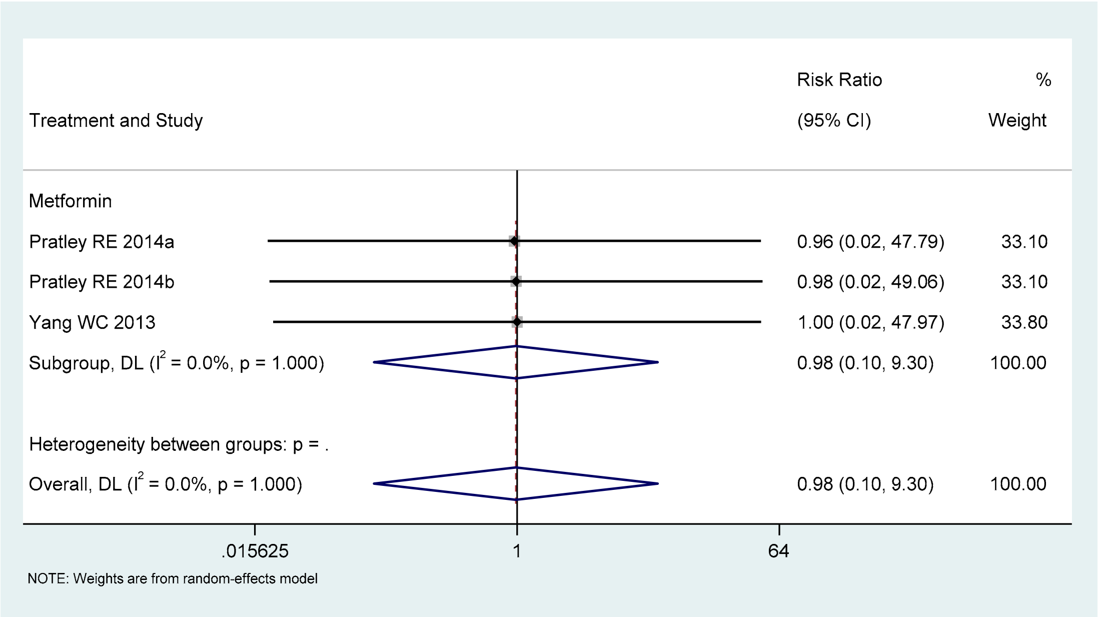

Figure S73. Meta-analysis results for incidence of myocardial infarction of metformin vs placebo/lifestyle intervention

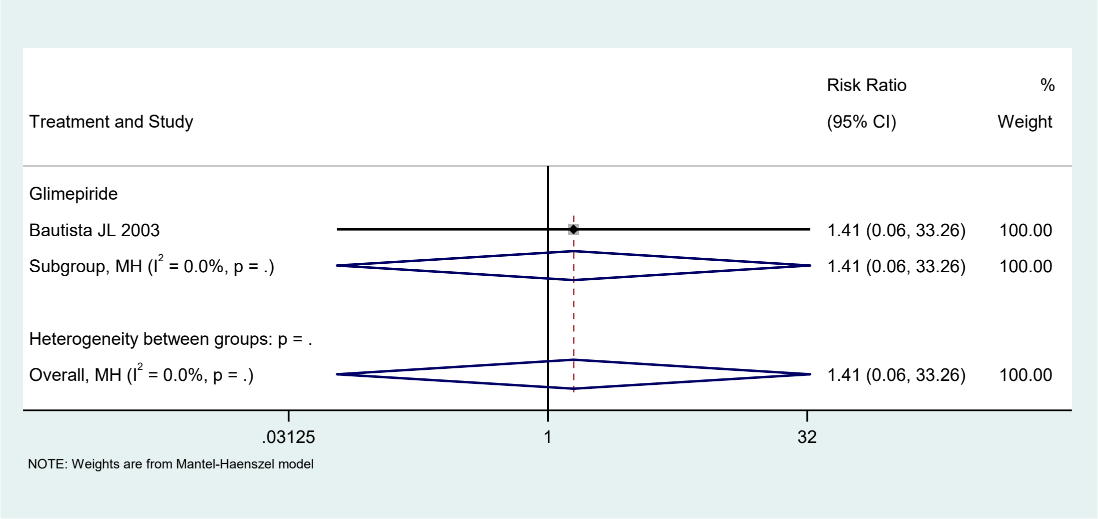

Figure S74. Meta-analysis results for incidence of myocardial infarction of sulfonylureas vs placebo/lifestyle intervention

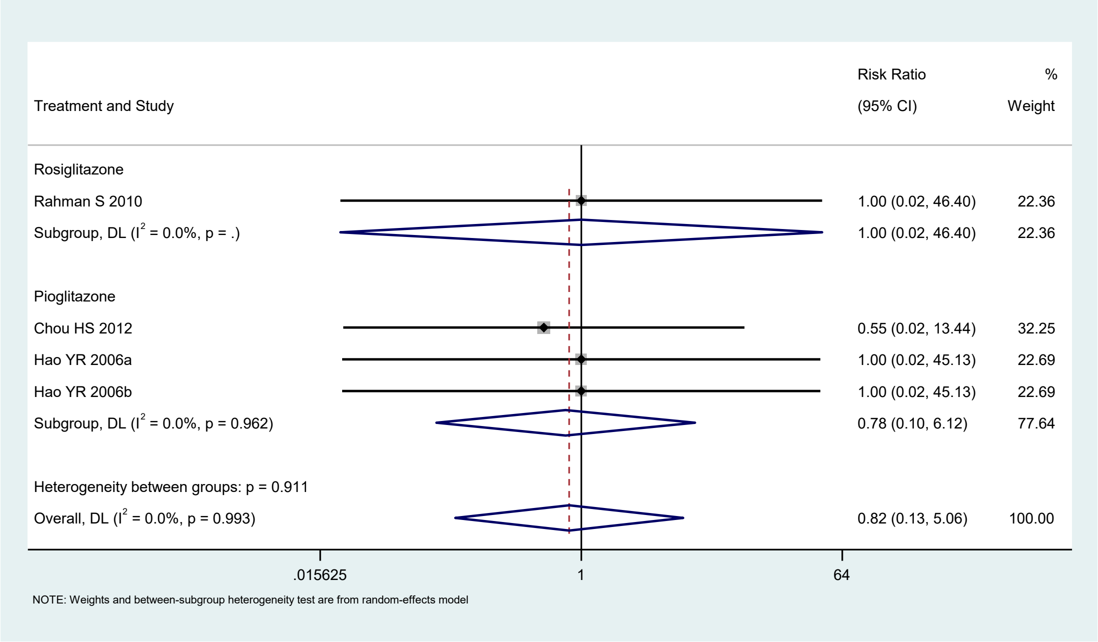

Figure S75. Meta-analysis results for incidence of myocardial infarction of thiazolidinediones vs placebo/lifestyle intervention

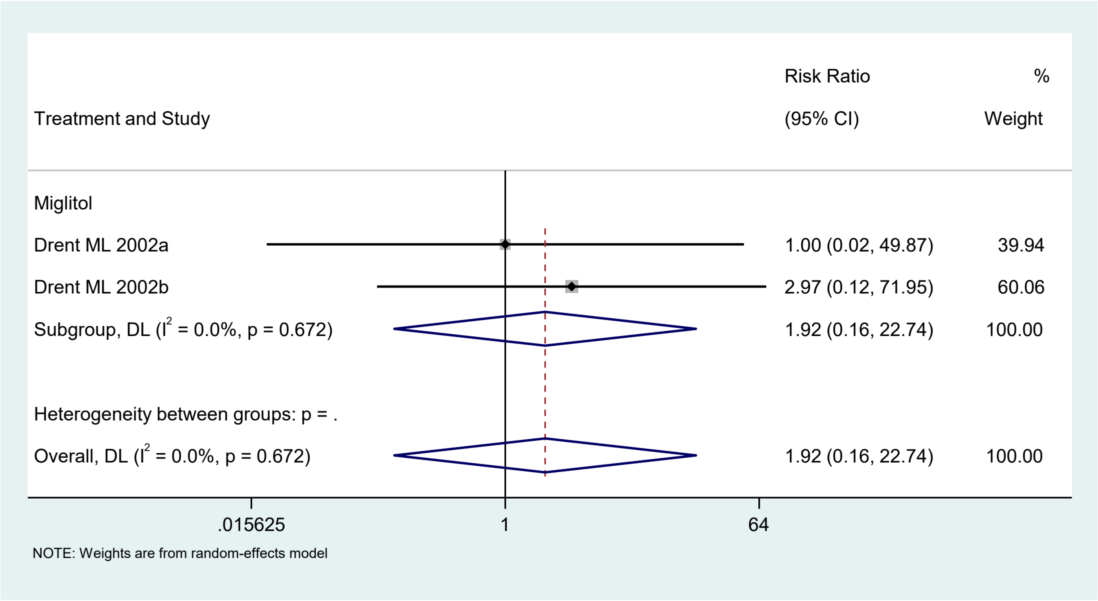

Figure S76. Meta-analysis results for incidence of myocardial infarction of α-glucosidase inhibitors vs placebo/lifestyle intervention

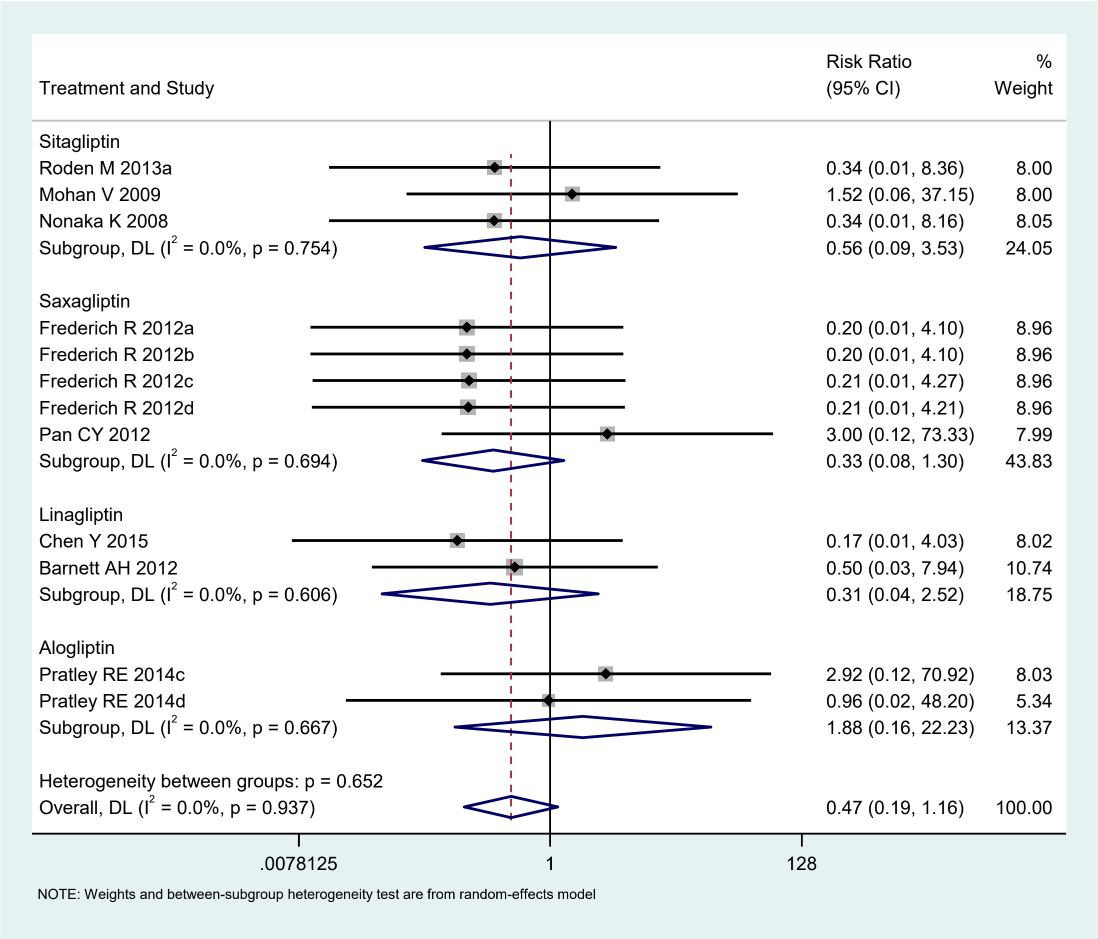

Figure S77. Meta-analysis results for incidence of myocardial infarction of dipeptidyl peptidase-4 inhibitors vs placebo/lifestyle intervention

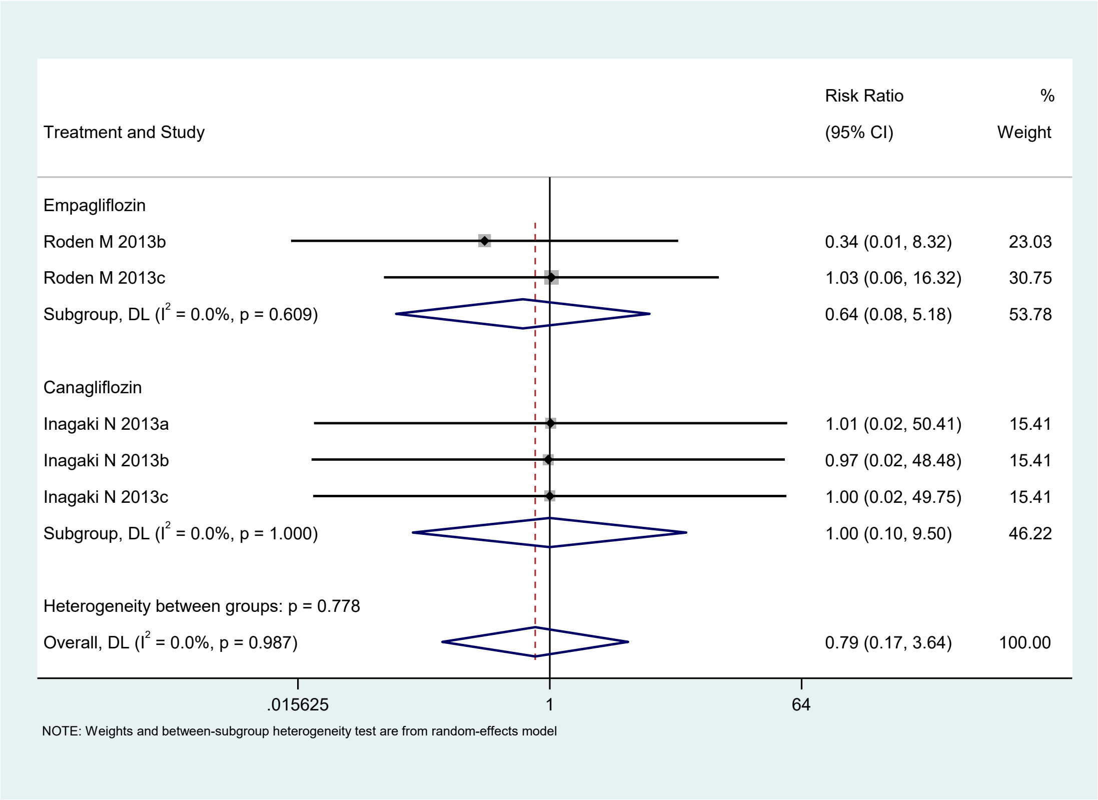

Figure S78. Meta-analysis results for incidence of myocardial infarction of sodium-glucose cotransporter-2 inhibitors vs placebo/lifestyle intervention

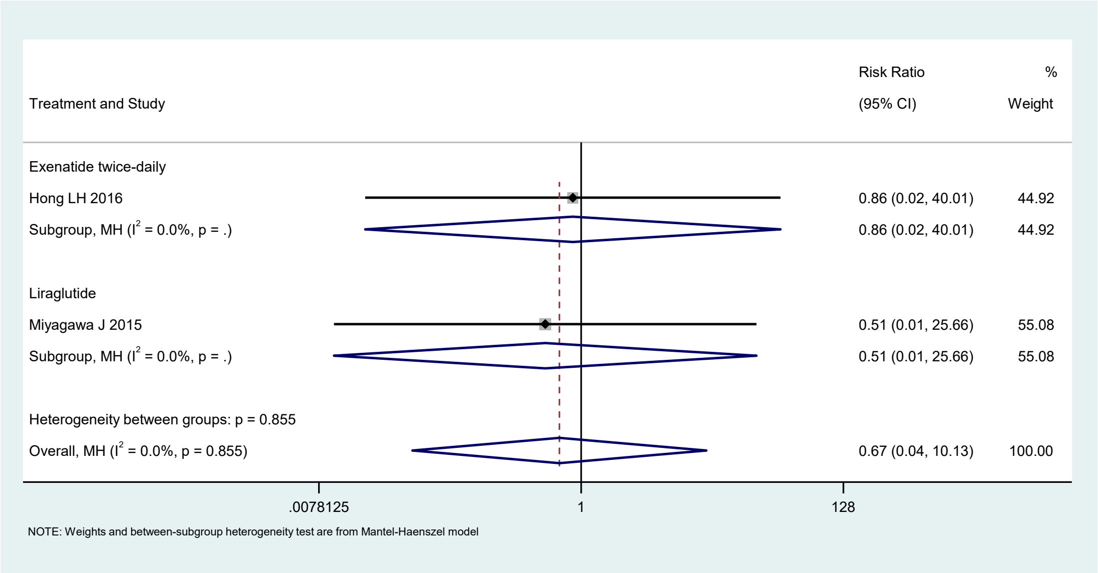

Figure S79. Meta-analysis results for incidence of myocardial infarction of glucagon-like peptide-1 receptor agonists vs placebo/lifestyle intervention

【Heart failure】

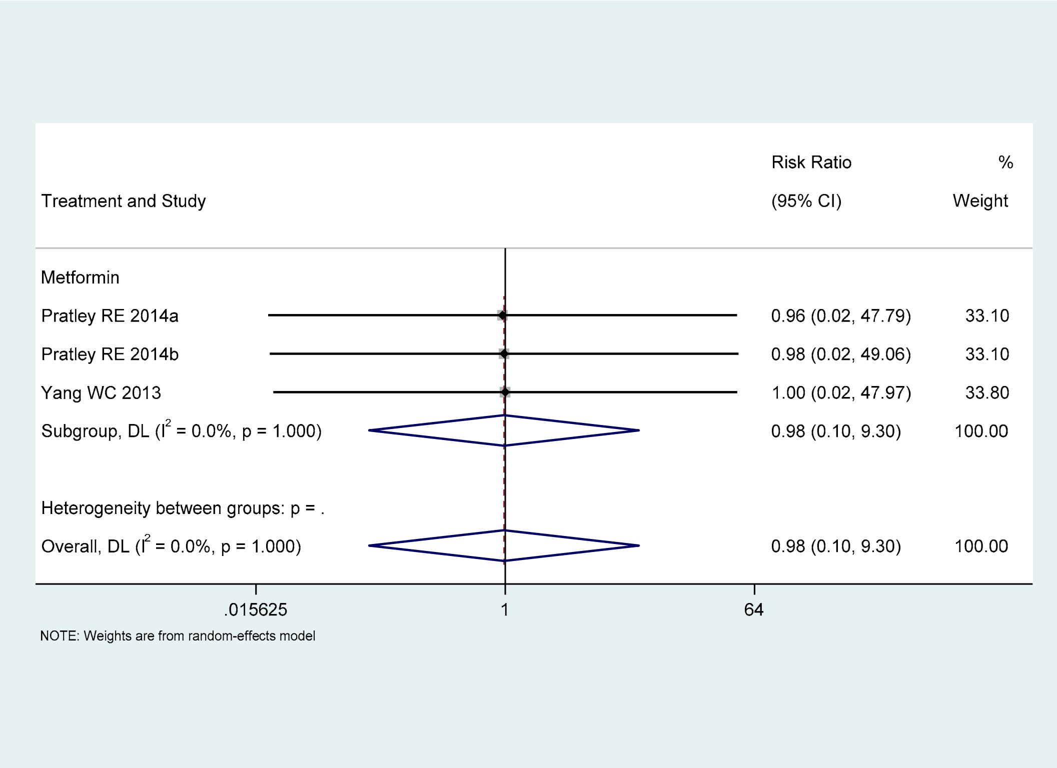

Figure S80. Meta-analysis results for incidence of heart failure of metformin vs placebo/lifestyle intervention

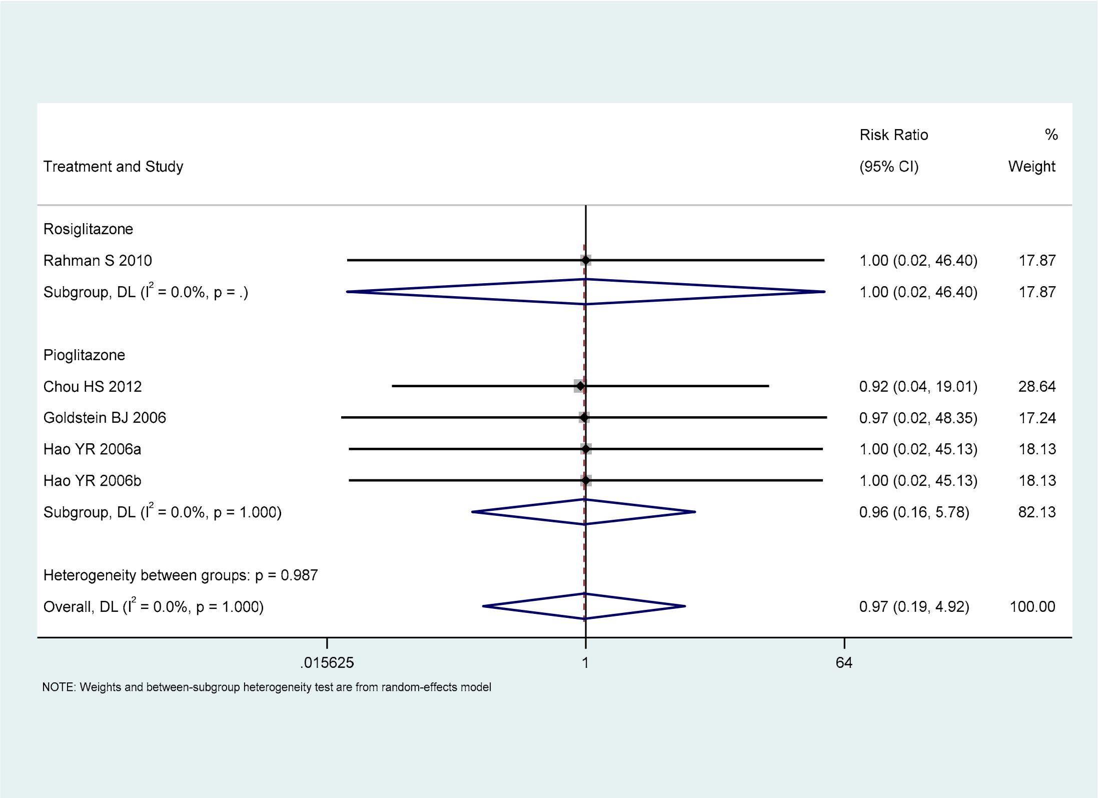

Figure S81. Meta-analysis results for incidence of heart failure of thiazolidinediones vs placebo/lifestyle intervention

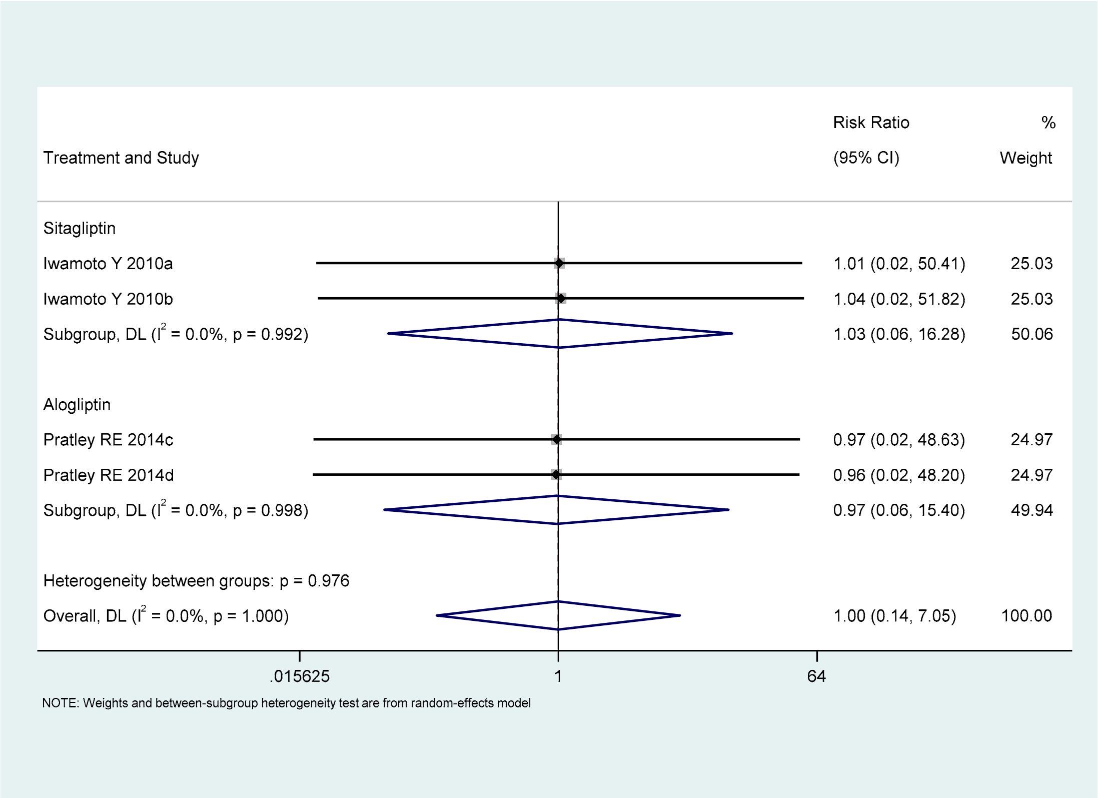

Figure S82. Meta-analysis results for incidence of heart failure of dipeptidyl peptidase-4 inhibitors vs placebo/lifestyle intervention

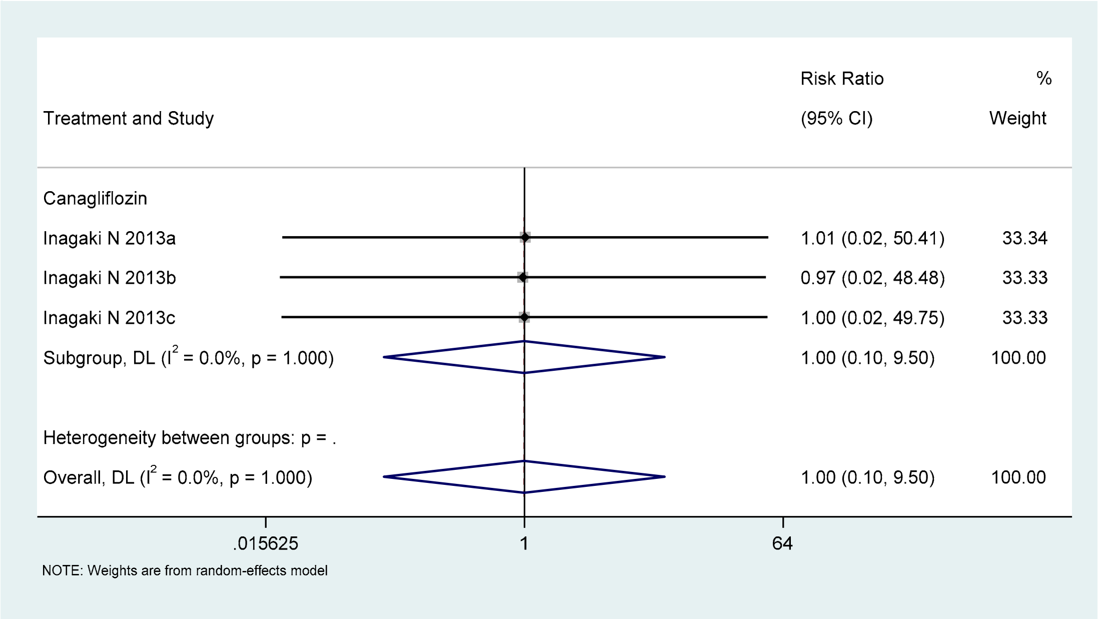

Figure S83. Meta-analysis results for incidence of heart failure of sodium-glucose cotransporter-2 inhibitors vs placebo/lifestyle intervention

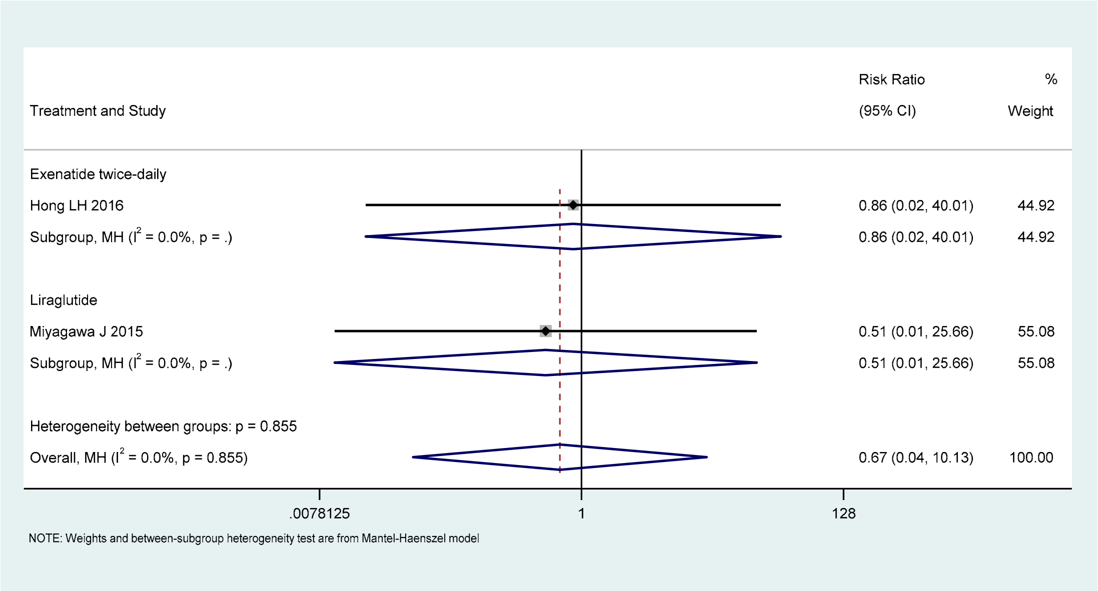

Figure S84. Meta-analysis results for incidence of heart failure of glucagon-like peptide-1 receptor agonists vs placebo/lifestyle intervention

【Stroke】

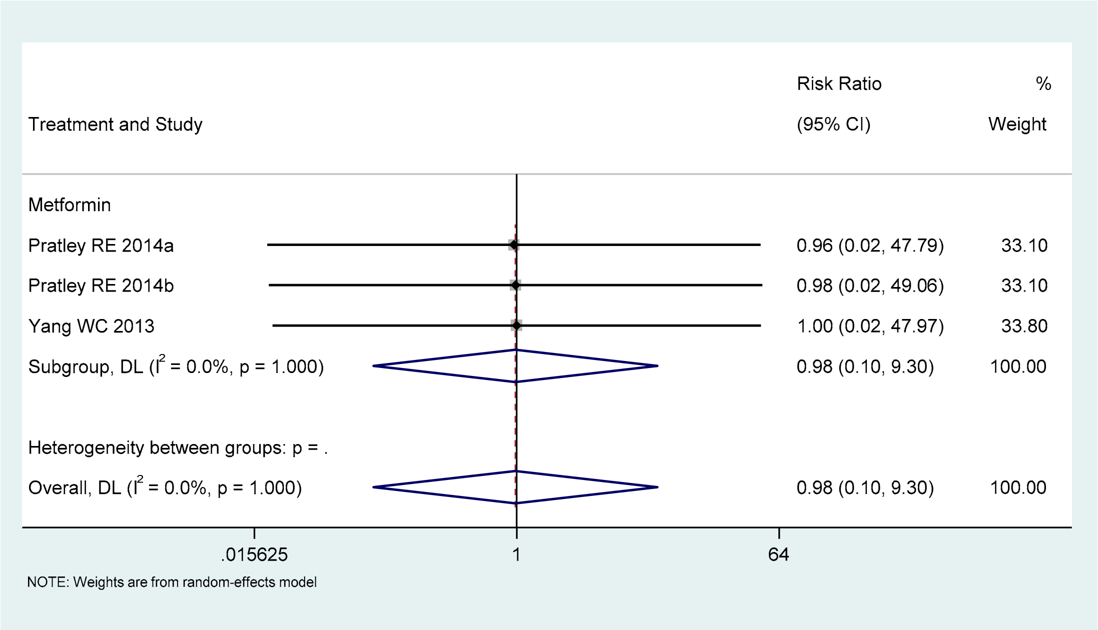

Figure S85. Meta-analysis results for incidence of stroke of metformin vs placebo/lifestyle intervention

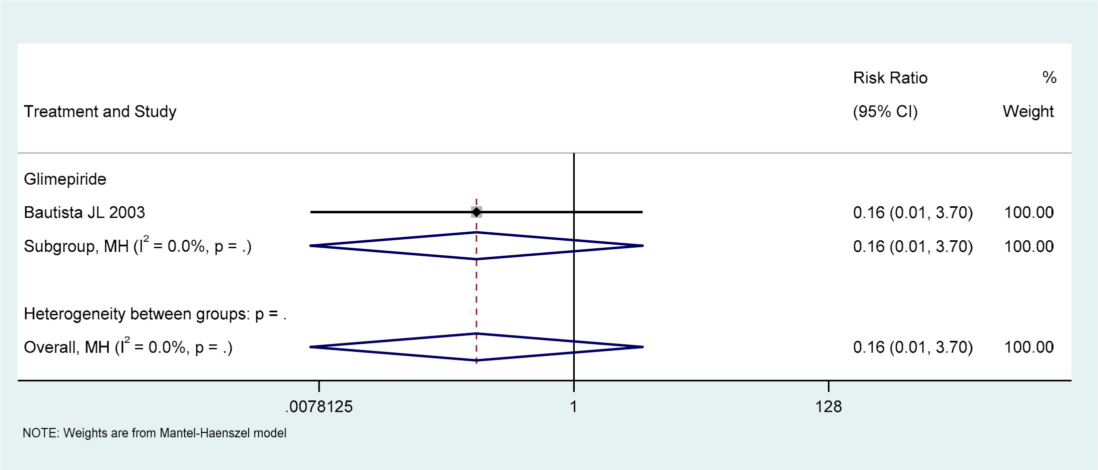

Figure S86. Meta-analysis results for incidence of stroke of sulfonylureas vs placebo/lifestyle intervention

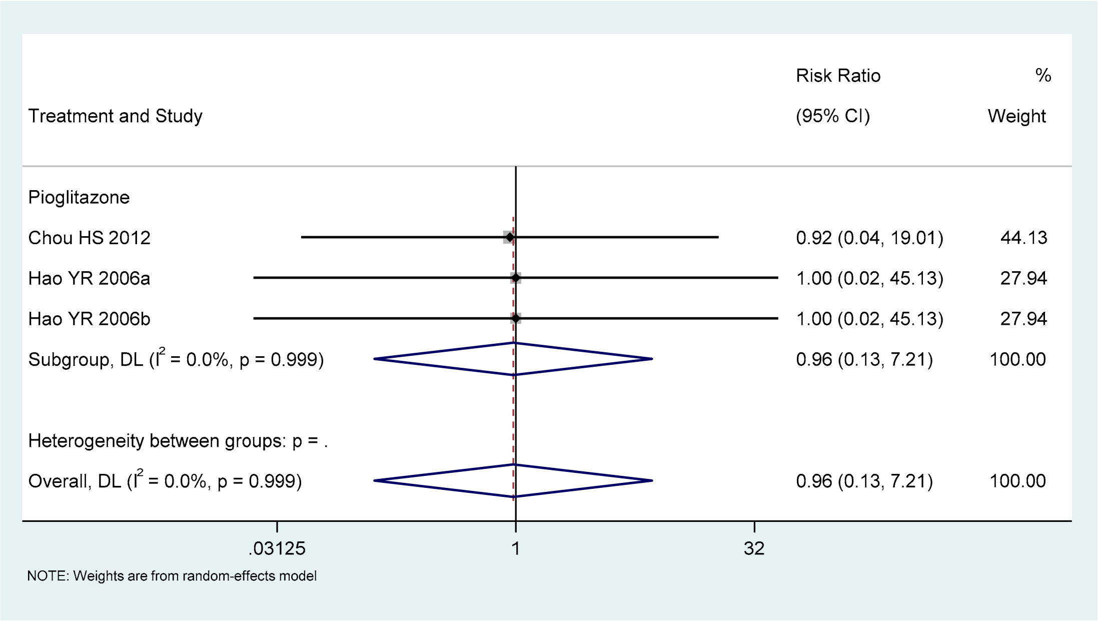

Figure S87. Meta-analysis results for incidence of stroke of thiazolidinediones vs placebo/lifestyle intervention

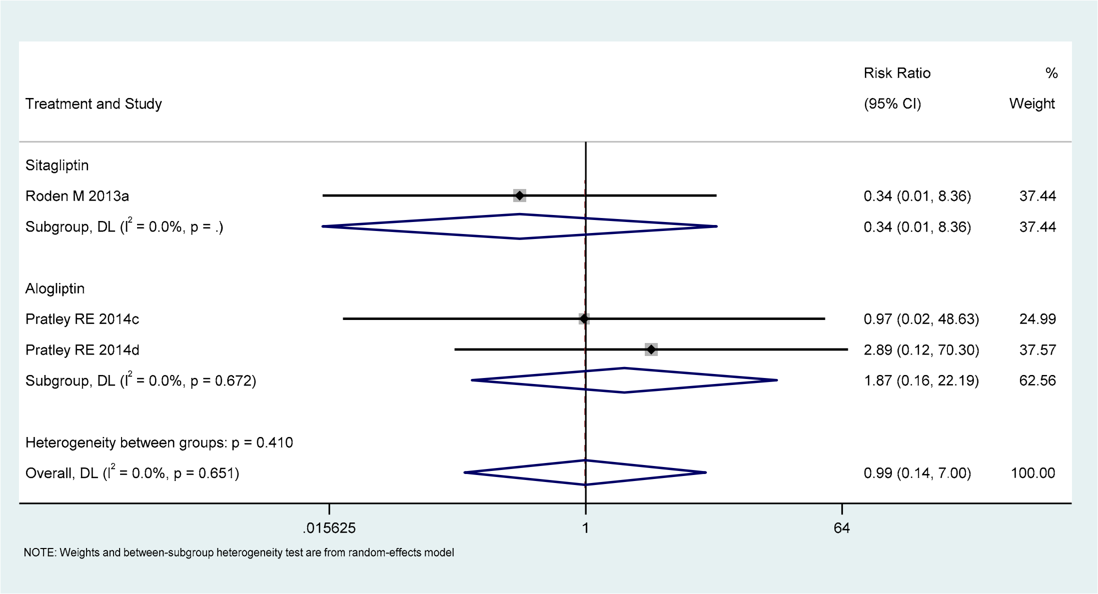

Figure S88. Meta-analysis results for incidence of stroke of dipeptidyl peptidase-4 inhibitors vs placebo/lifestyle intervention

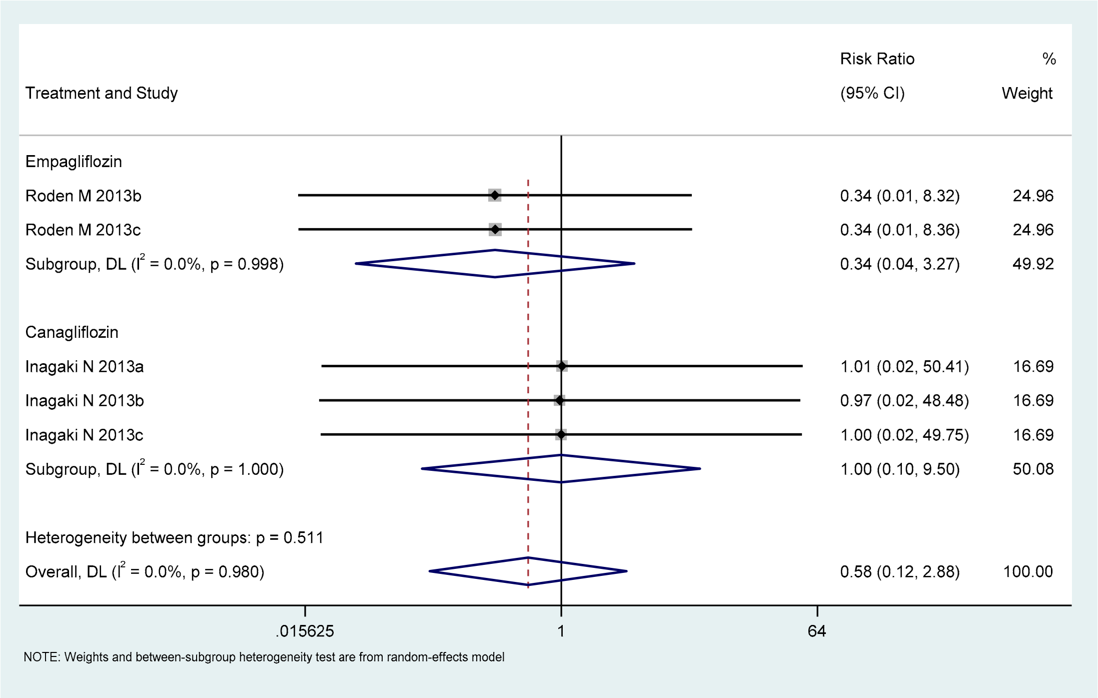

Figure S89. Meta-analysis results for incidence of stroke of sodium-glucose cotransporter-2 inhibitors vs placebo/lifestyle intervention

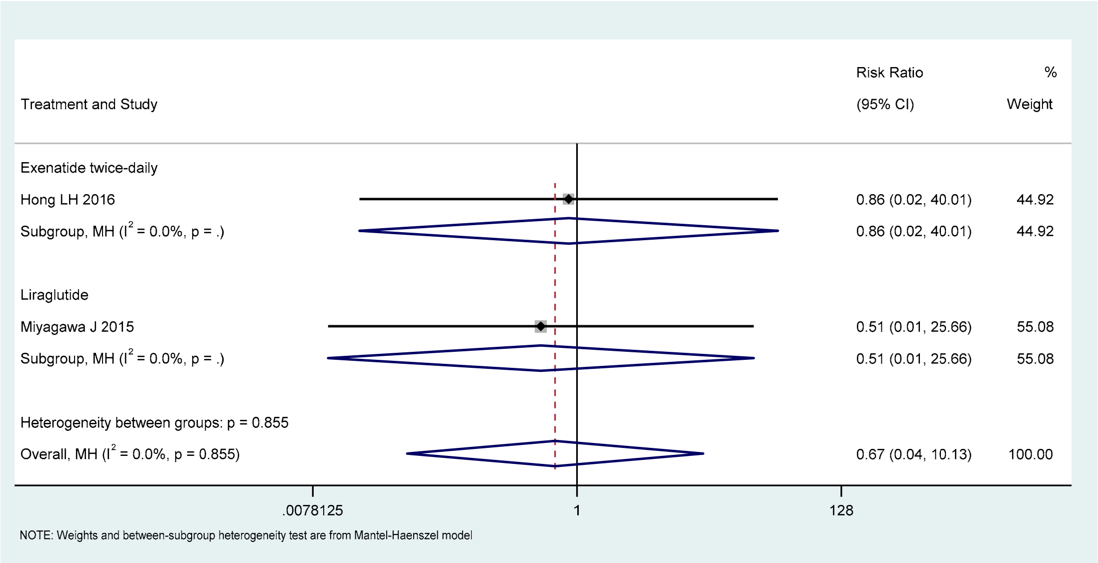

Figure S90. Meta-analysis results for incidence of stroke of glucagon-like peptide-1 receptor agonists vs placebo/lifestyle intervention

【Diabetic nephropathy】

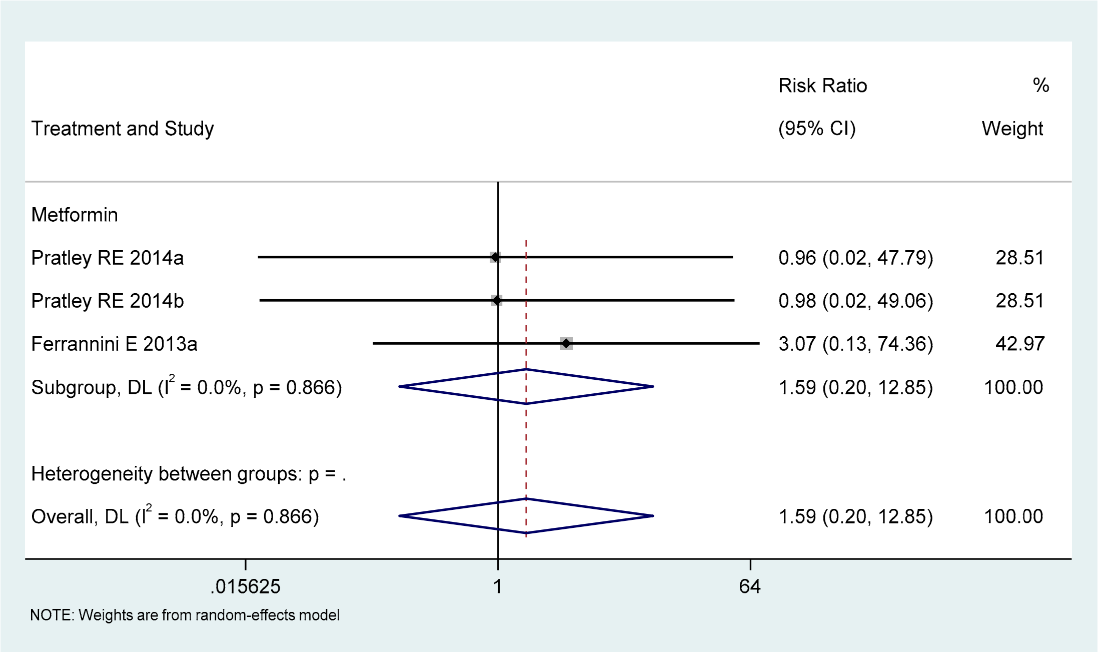

Figure S91. Meta-analysis results for incidence of diabetic nephropathy of metformin vs placebo/lifestyle intervention

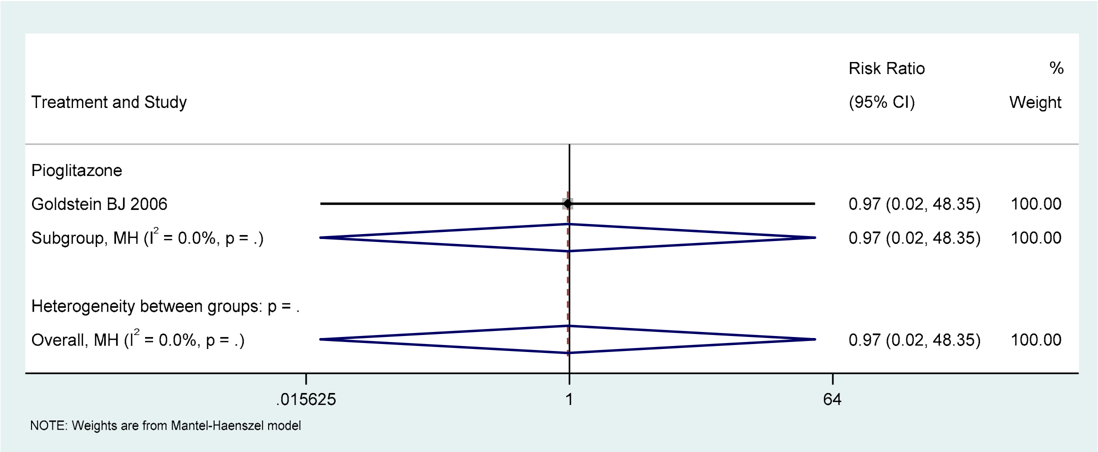

Figure S92. Meta-analysis results for incidence of diabetic nephropathy of thiazolidinediones vs placebo/lifestyle intervention

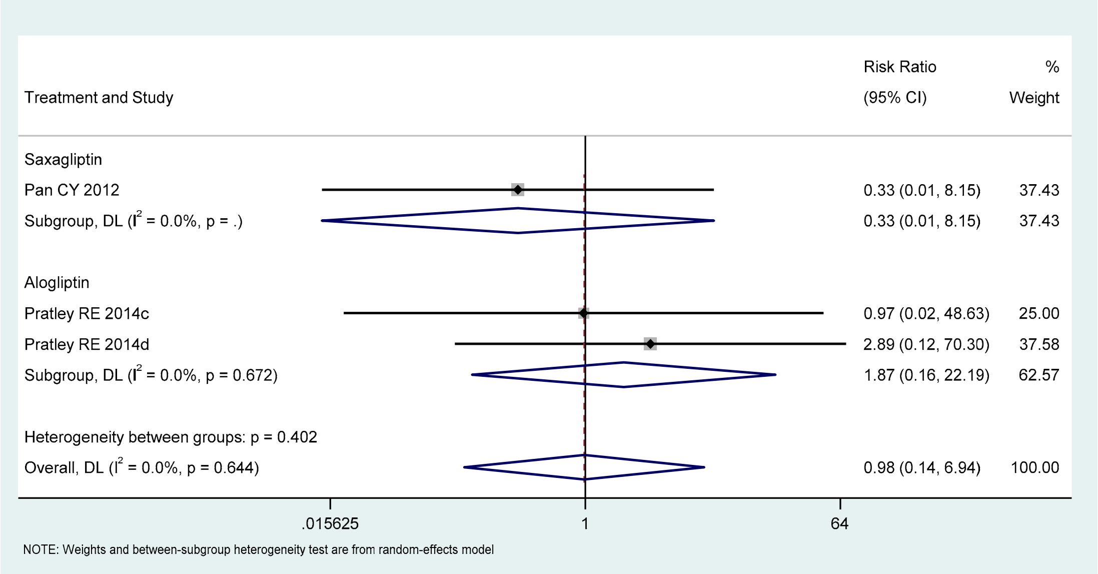

Figure S93. Meta-analysis results for incidence of diabetic nephropathy of dipeptidyl peptidase-4 inhibitors vs placebo/lifestyle intervention

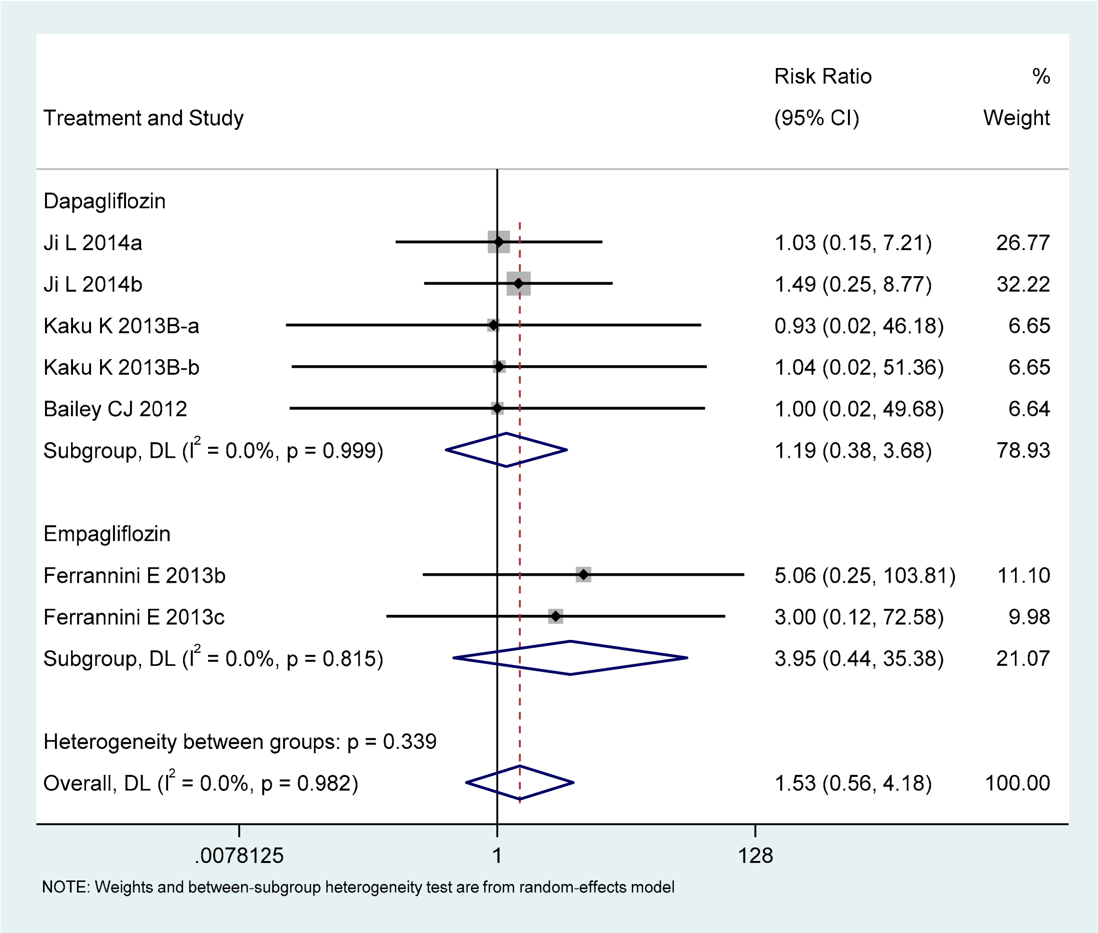

Figure S94. Meta-analysis results for incidence of diabetic nephropathy of sodium-glucose cotransporter-2 inhibitors vs placebo/lifestyle intervention

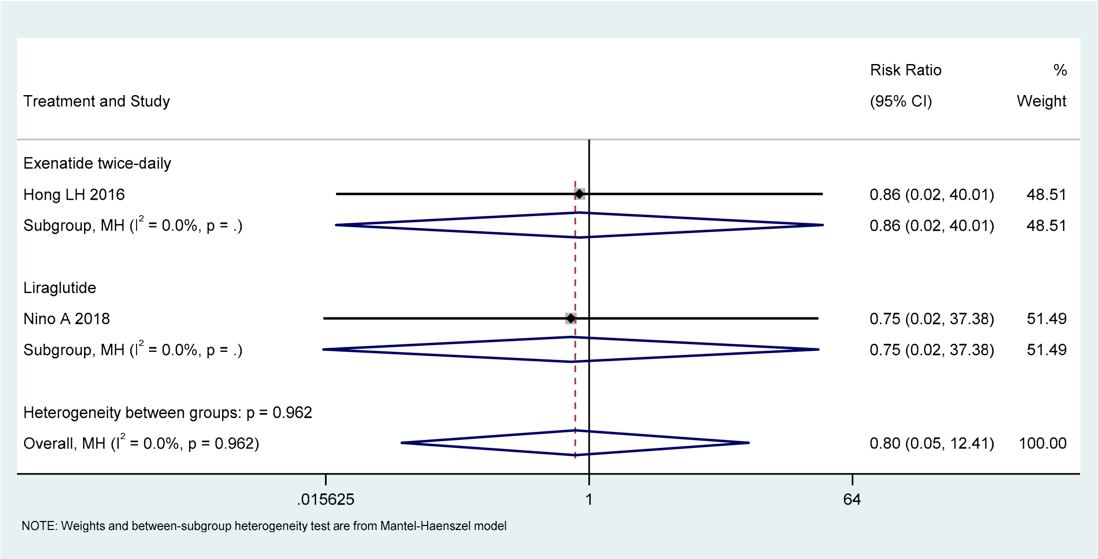

Figure S95. Meta-analysis results for incidence of diabetic nephropathy of glucagon-like peptide-1 receptor agonists vs placebo/lifestyle intervention

【AE-induced discontinuations】

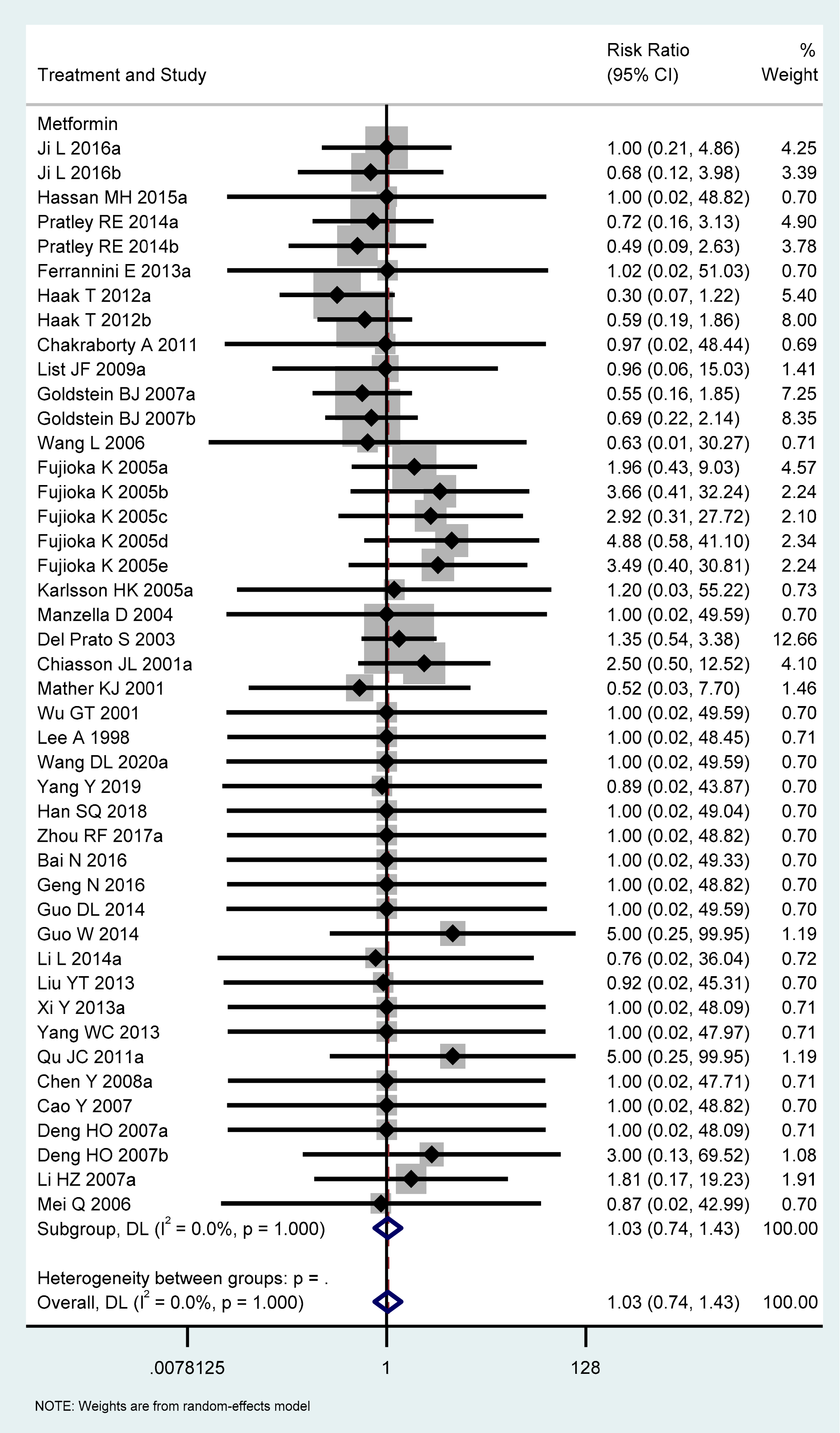

Figure S96. Meta-analysis results for incidence of AE-induced discontinuations of metformin vs placebo/lifestyle intervention

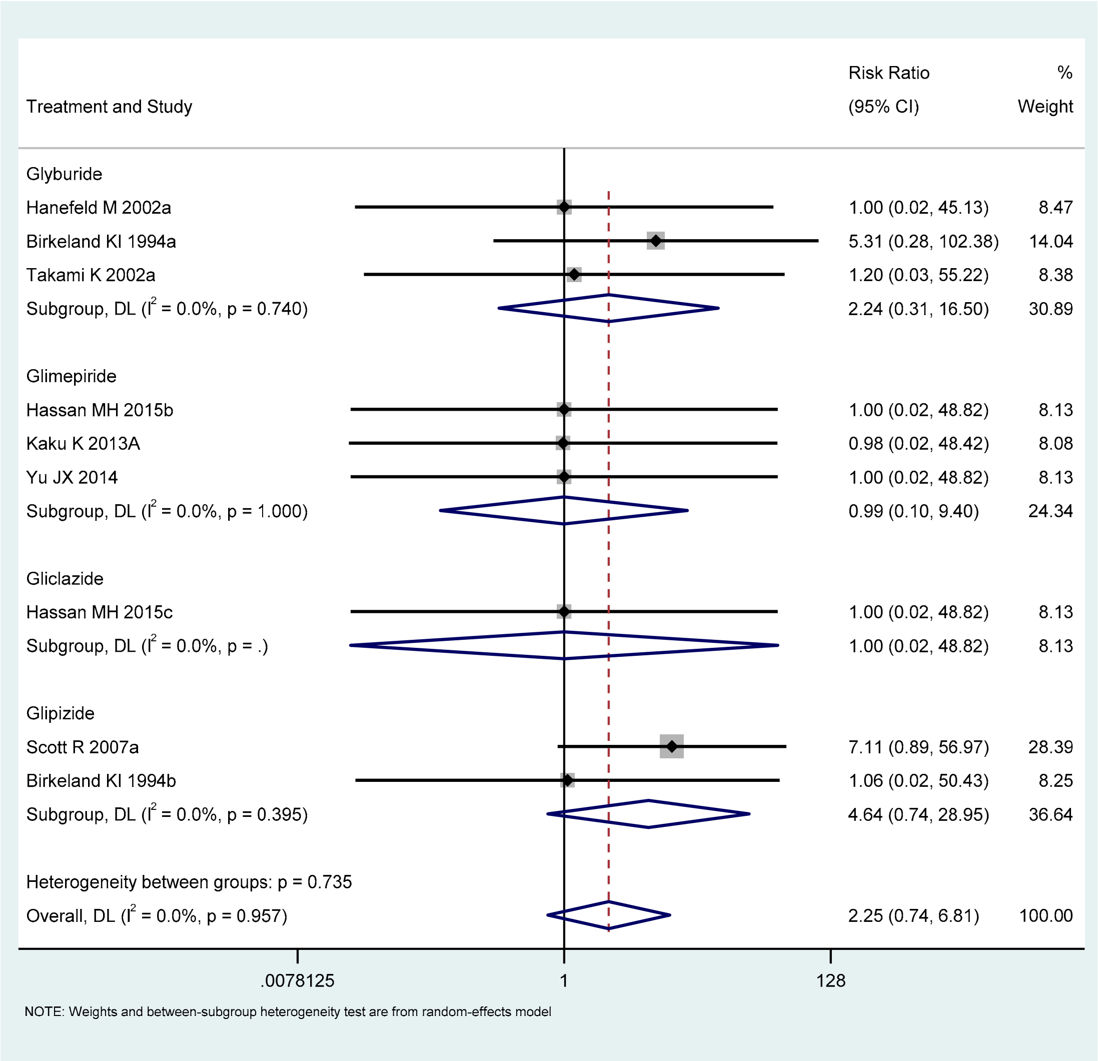

Figure S97. Meta-analysis results for incidence of AE-induced discontinuations of sulfonylureas vs placebo/lifestyle intervention

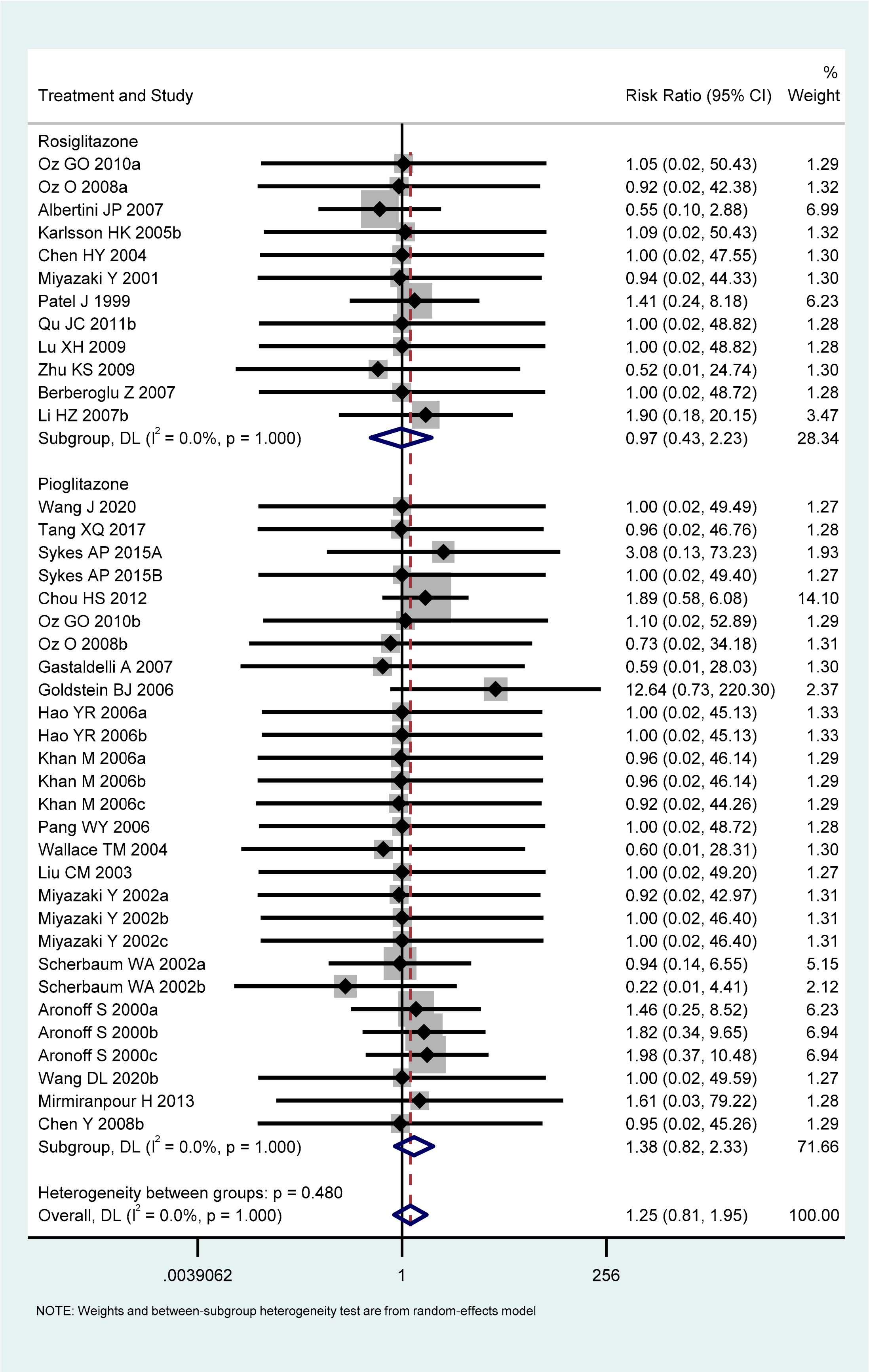

Figure S98. Meta-analysis results for incidence of AE-induced discontinuations of thiazolidinediones vs placebo/lifestyle intervention

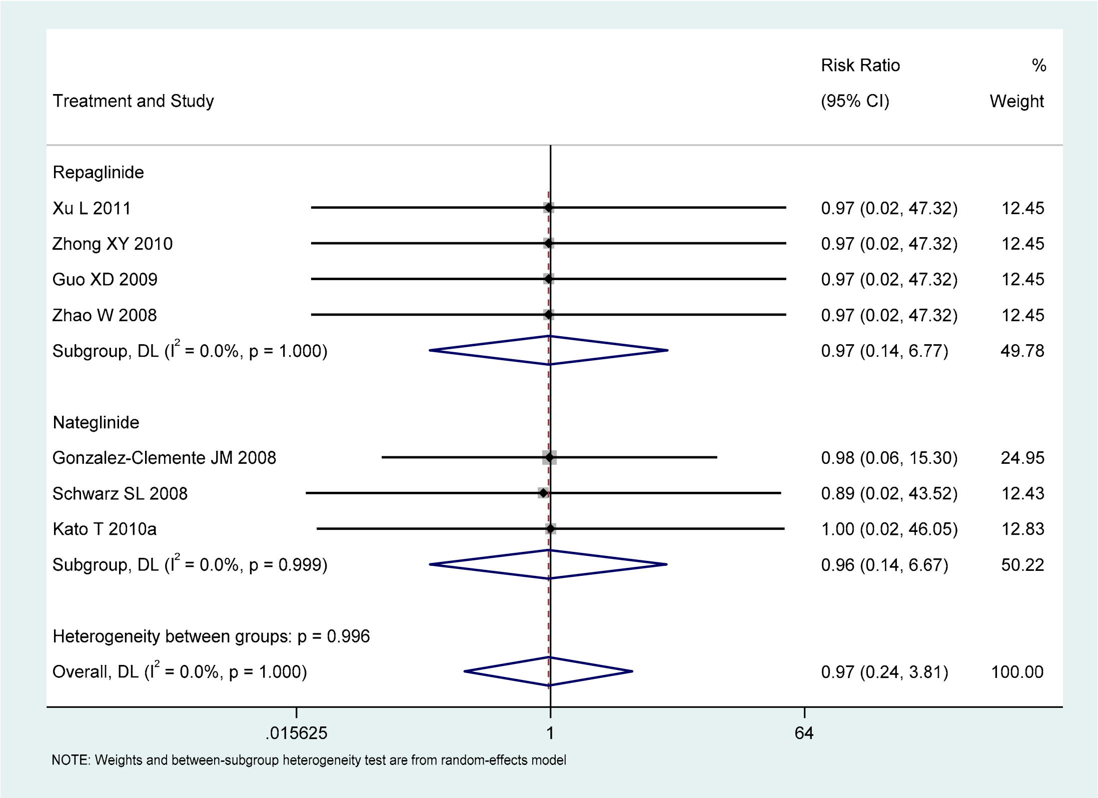

Figure S99. Meta-analysis results for incidence of AE-induced discontinuations of glinides vs placebo/lifestyle intervention

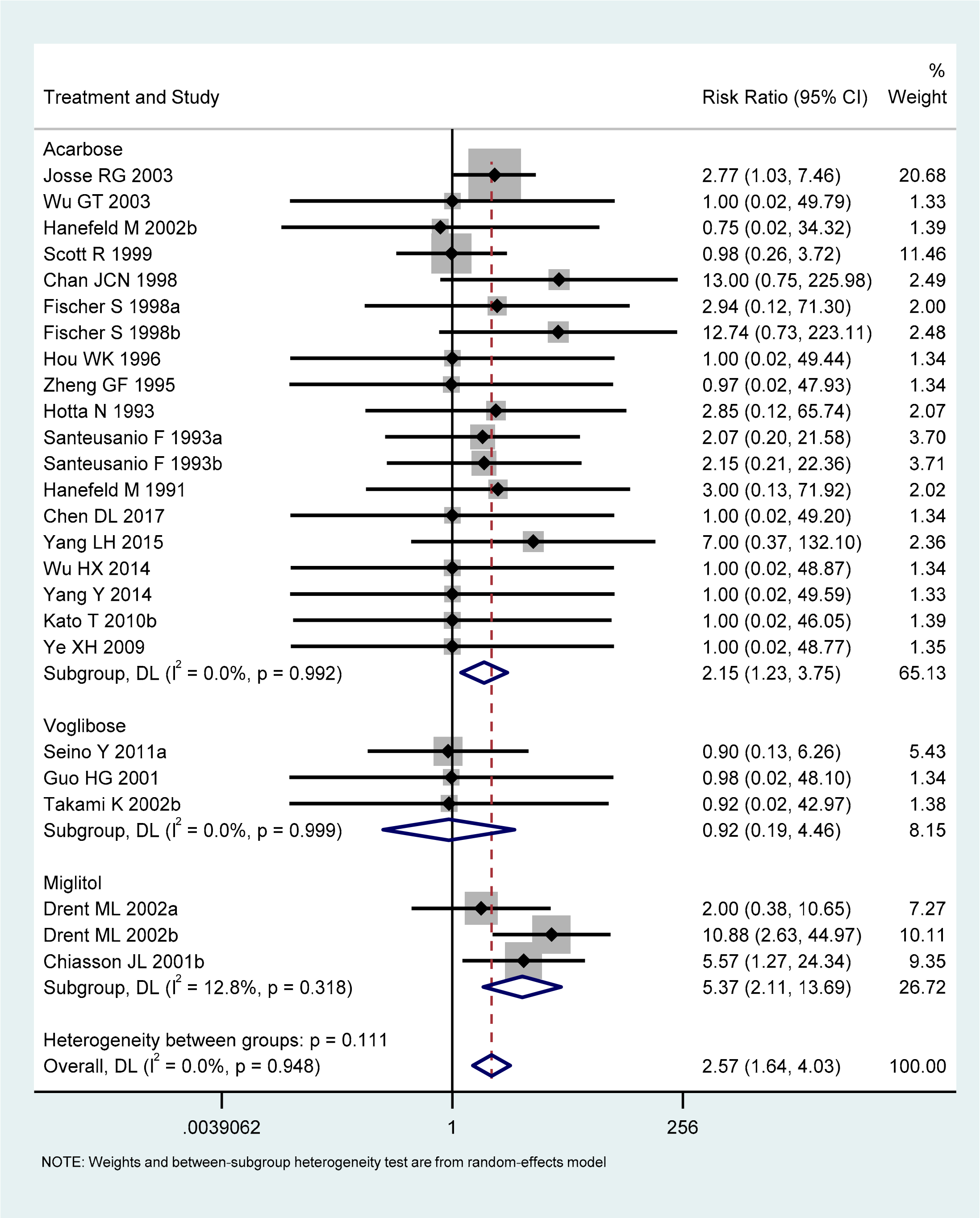

Figure S100. Meta-analysis results for incidence of AE-induced discontinuations of α-glucosidase inhibitors vs placebo/lifestyle intervention

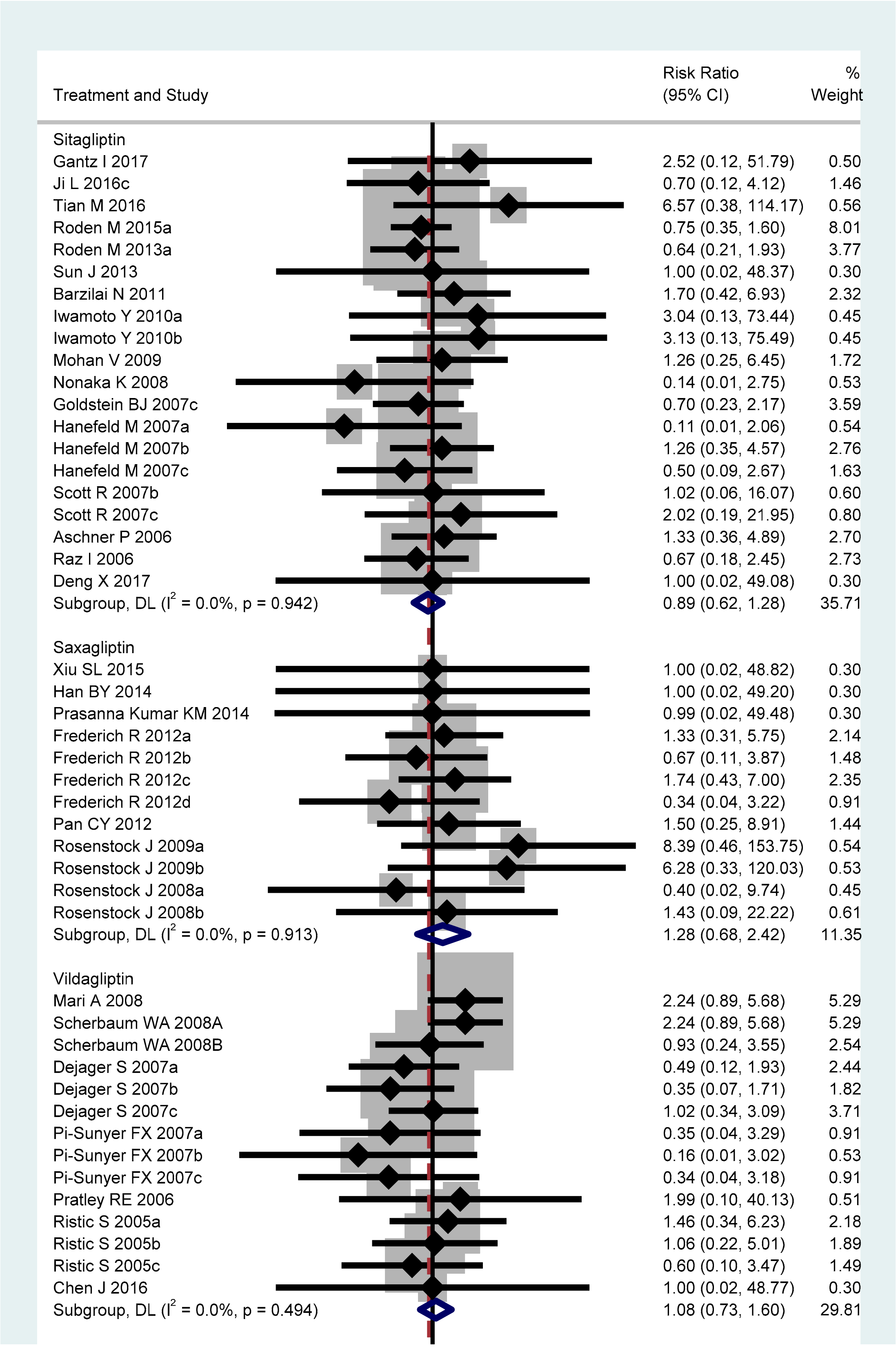
Treatment and Study
Risk Ratio (95% CI)
% Weight
Sitagliptin
Gantz I 2017 2.52 (0.12, 51.79) 0.50
Ji L 2016c 0.70 (0.12, 4.12) 1.46
Tian M 2016 6.57 (0.38, 114.17) 0.56
Roden M 2015a 0.75 (0.35, 1.60) 8.01
Roden M 2013a 0.64 (0.21, 1.93) 3.77
Sun J 2013 1.00 (0.02, 48.37) 0.30
Barzilai N 2011 1.70 (0.42, 6.93) 2.32
Iwamoto Y 2010a 3.04 (0.13, 73.44) 0.45
Iwamoto Y 2010b 3.13 (0.13, 75.49) 0.45
Mohan V 2009 1.26 (0.25, 6.45) 1.72
Nonaka K 2008 0.14 (0.01, 2.75) 0.53
Goldstein BJ 2007c 0.70 (0.23, 2.17) 3.59
Hanefeld M 2007a 0.11 (0.01, 2.06) 0.54
Hanefeld M 2007b 1.26 (0.35, 4.57) 2.76
Hanefeld M 2007c 0.50 (0.09, 2.67) 1.63
Scott R 2007b 1.02 (0.06, 16.07) 0.60
Scott R 2007c 2.02 (0.19, 21.95) 0.80
Aschner P 2006 1.33 (0.36, 4.89) 2.70
Raz I 2006 0.67 (0.18, 2.45) 2.73
Deng X 2017 1.00 (0.02, 49.08) 0.30
Subgroup, DL (I^2 = 0.0%, p = 0.942) 0.89 (0.62, 1.28) 35.71
Saxagliptin
Xiu SL 2015 1.00 (0.02, 48.82) 0.30
Han BY 2014 1.00 (0.02, 49.20) 0.30
Prasanna Kumar KM 2014 0.99 (0.02, 49.48) 0.30
Frederich R 2012a 1.33 (0.31, 5.75) 2.14
Frederich R 2012b 0.67 (0.11, 3.87) 1.48
Frederich R 2012c 1.74 (0.43, 7.00) 2.35
Frederich R 2012d 0.34 (0.04, 3.22) 0.91
Pan CY 2012 1.50 (0.25, 8.91) 1.44
Rosenstock J 2009a 8.39 (0.46, 153.75) 0.54
Rosenstock J 2009b 6.28 (0.33, 120.03) 0.53
Rosenstock J 2008a 0.40 (0.02, 9.74) 0.45
Rosenstock J 2008b 1.43 (0.09, 22.22) 0.61
Subgroup, DL (I^2 = 0.0%, p = 0.913) 1.28 (0.68, 2.42) 11.35
Vildagliptin
Mari A 2008 2.24 (0.89, 5.68) 5.29
Scherbaum WA 2008A 2.24 (0.89, 5.68) 5.29
Scherbaum WA 2008B 0.93 (0.24, 3.55) 2.54
Dejager S 2007a 0.49 (0.12, 1.93) 2.44
Dejager S 2007b 0.35 (0.07, 1.71) 1.82
Dejager S 2007c 1.02 (0.34, 3.09) 3.71
Pi-Sunyer FX 2007a 0.35 (0.04, 3.29) 0.91
Pi-Sunyer FX 2007b 0.16 (0.01, 3.02) 0.53
Pi-Sunyer FX 2007c 0.34 (0.04, 3.18) 0.91
Pratley RE 2006 1.99 (0.10, 40.13) 0.51
Ristic S 2005a 1.46 (0.34, 6.23) 2.18
Ristic S 2005b 1.06 (0.22, 5.01) 1.89
Ristic S 2005c 0.60 (0.10, 3.47) 1.49
Chen J 2016 1.00 (0.02, 48.77) 0.30
Subgroup, DL (I^2 = 0.0%, p = 0.494) 1.08 (0.73, 1.60) 29.81

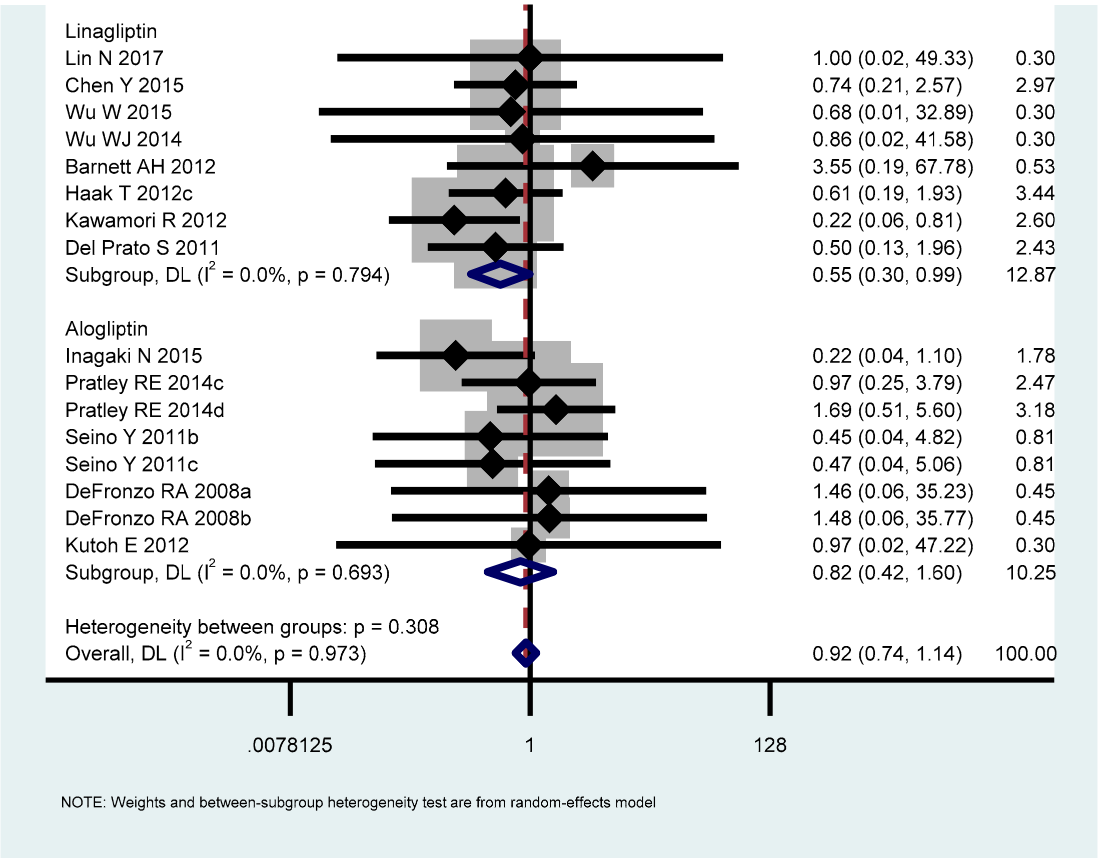

Figure S101. Meta-analysis results for incidence of AE-induced discontinuations of dipeptidyl peptidase-4 inhibitors vs placebo/lifestyle intervention

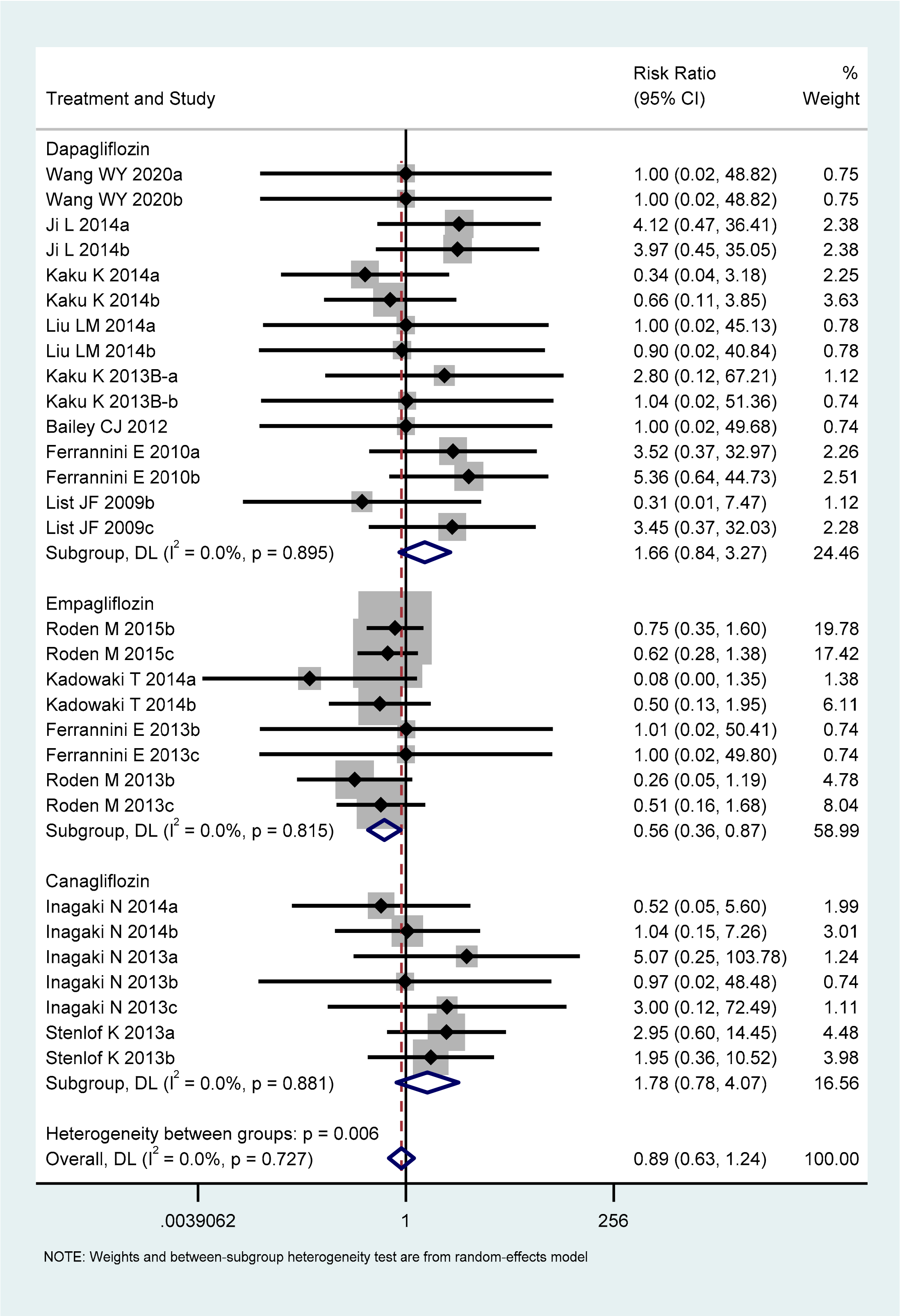

Figure S102. Meta-analysis results for incidence of AE-induced discontinuations of sodium-glucose cotransporter-2 inhibitors vs placebo/lifestyle intervention

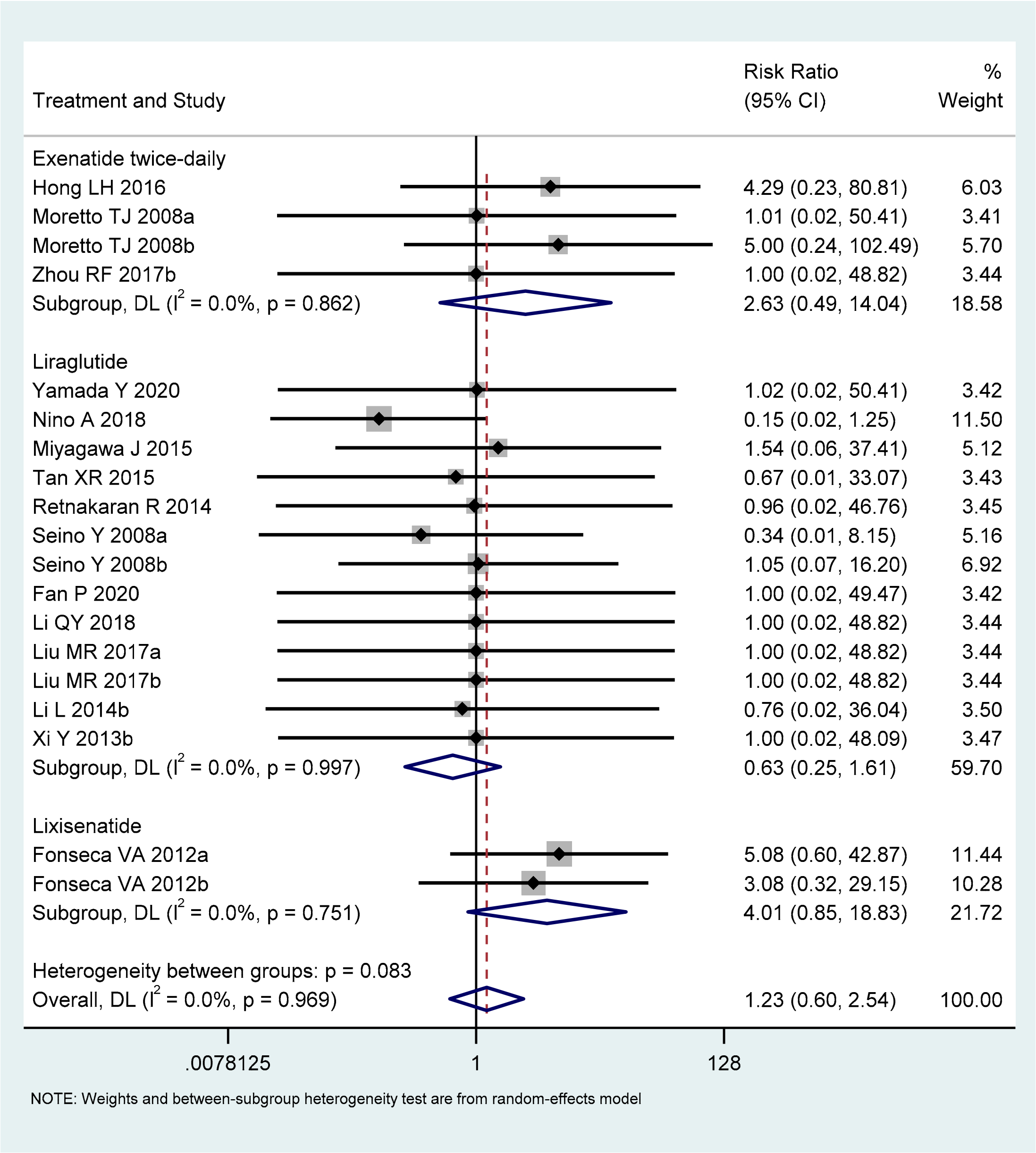

Figure S103. Meta-analysis results for incidence of AE-induced discontinuations of glucagon-like peptide-1 receptor agonists vs placebo/lifestyle intervention

【Sensitivity analysis】

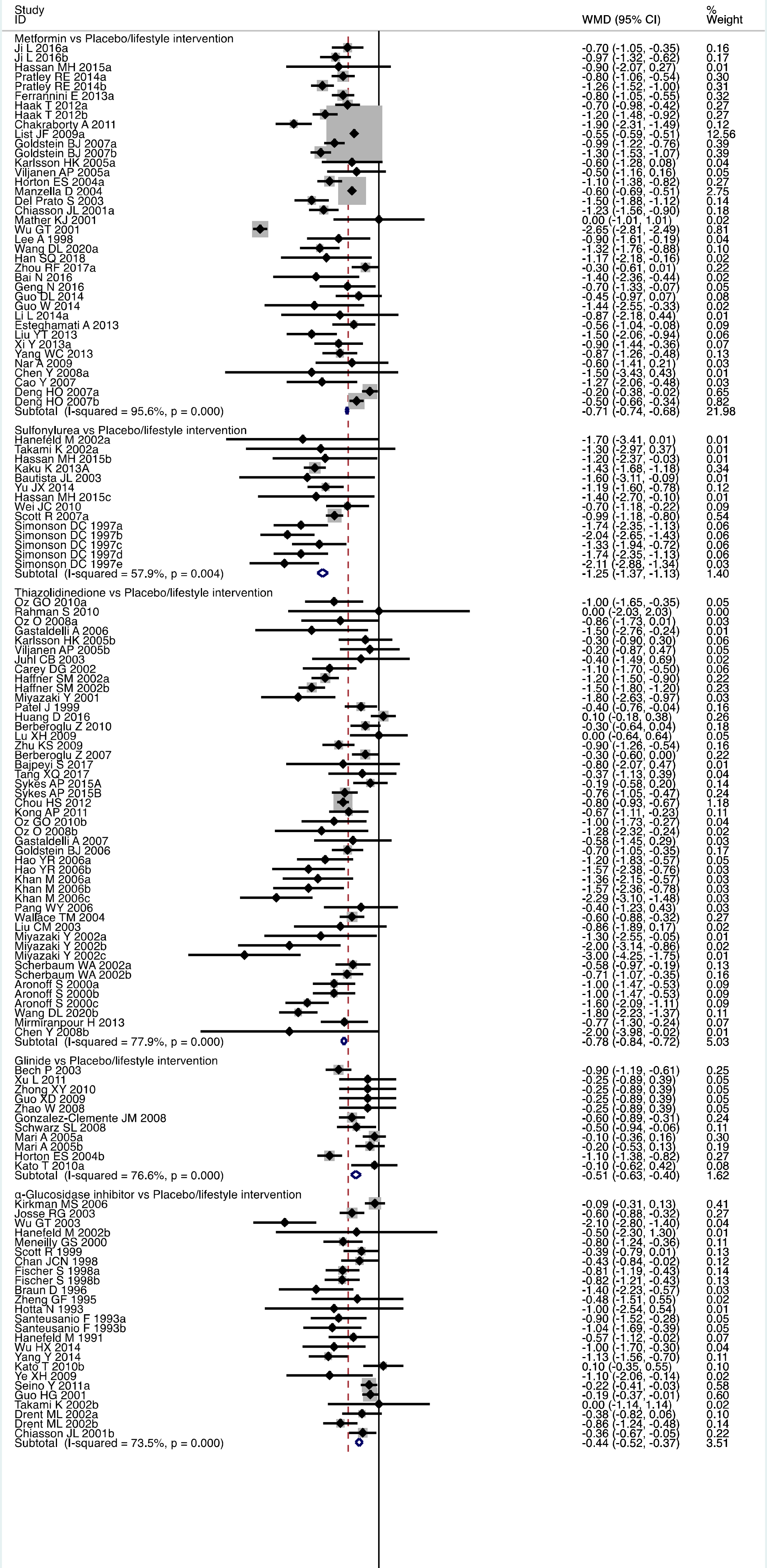

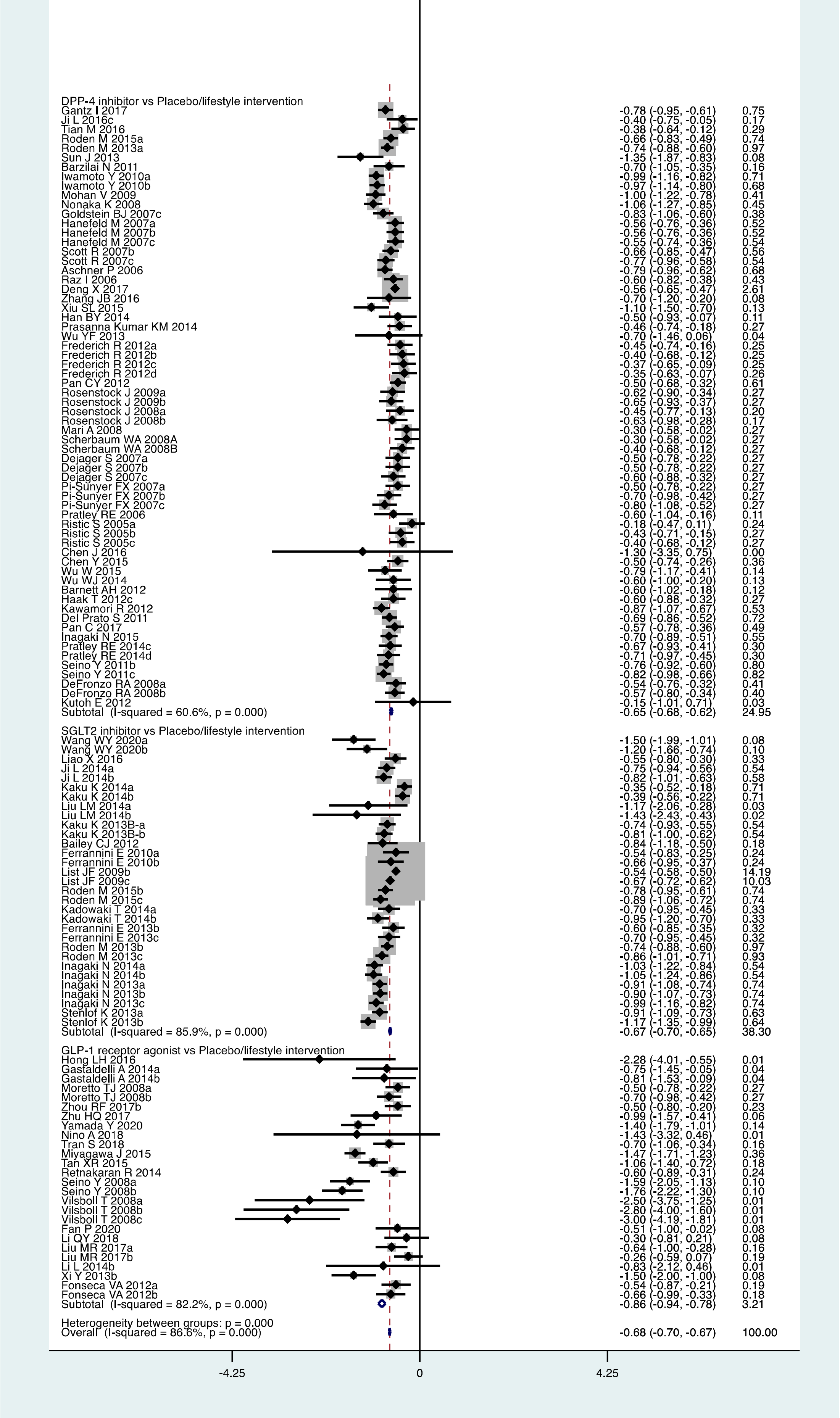

Figure S104. Sensitivity analysis results for change in hemoglobin Alc (%) of glucose-lowering drugs vs placebo/lifestyle intervention

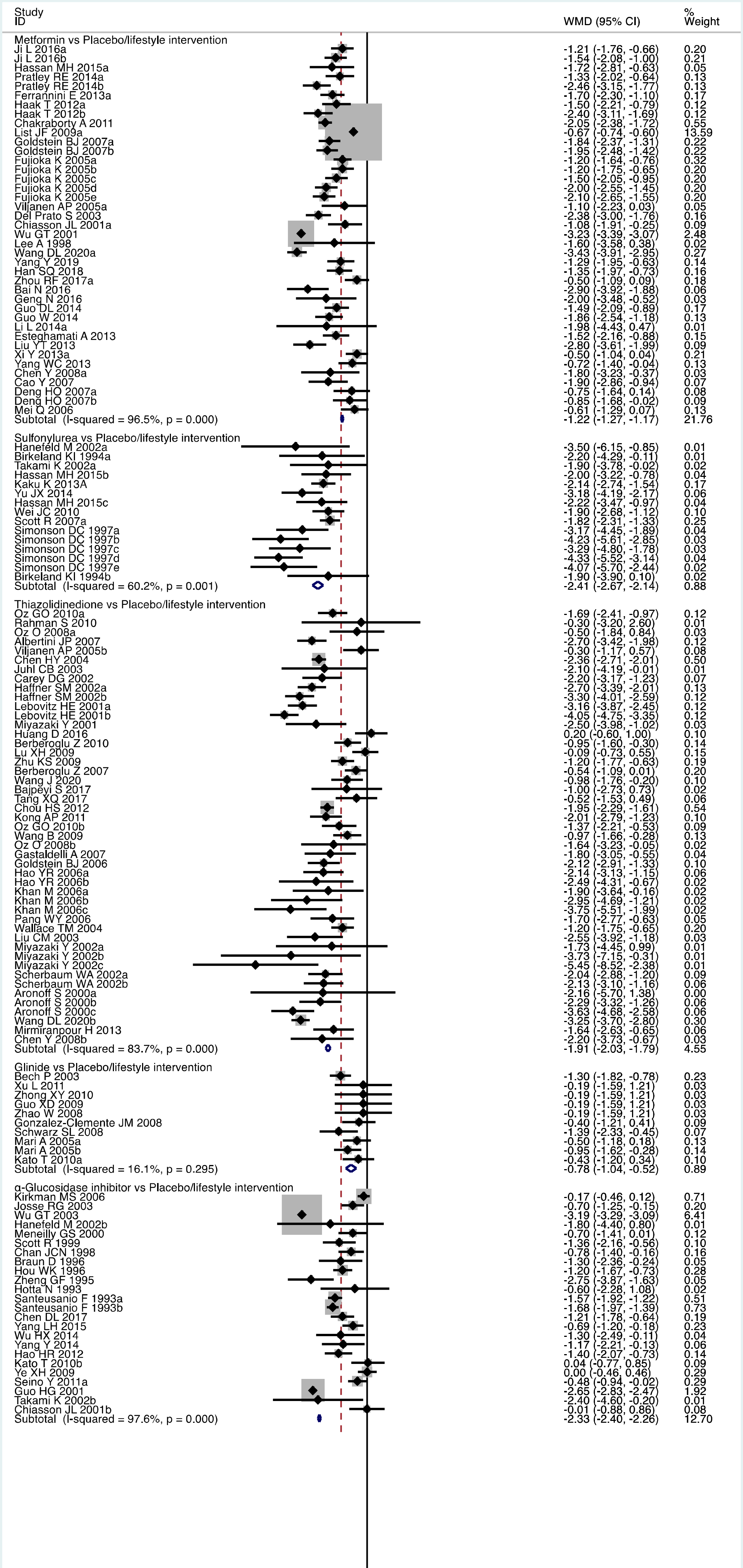
Study
ID
WMD (95% CI)
%
Weight
Metformin vs Placebo/lifestyle intervention
Ji L 2016a -1.21 (-1.76, -0.66) 0.20
Ji L 2016b -1.54 (-2.08, -1.00) 0.21
Hassan MH 2015a -1.72 (-2.81, -0.63) 0.05
Pratley RE 2014a -1.33 (-2.02, -0.64) 0.13
Pratley RE 2014b -2.46 (-3.15, -1.77) 0.13
Ferrannini E 2013a -1.70 (-2.30, -1.10) 0.17
Haak T 2012a -1.50 (-2.21, -0.79) 0.12
Haak T 2012b -2.40 (-3.11, -1.69) 0.12
Chakraborty A 2011 -2.05 (-2.38, -1.72) 0.55
List JF 2009a -0.67 (-0.74, -0.60) 13.59
Goldstein BJ 2007a -1.84 (-2.37, -1.31) 0.22
Goldstein BJ 2007b -1.95 (-2.48, -1.42) 0.22
Fujioka K 2005a -1.20 (-1.64, -0.76) 0.32
Fujioka K 2005b -1.20 (-1.75, -0.65) 0.20
Fujioka K 2005c -1.50 (-2.05, -0.95) 0.20
Fujioka K 2005d -2.00 (-2.55, -1.45) 0.20
Fujioka K 2005e -2.10 (-2.65, -1.55) 0.20
Viljanen AP 2005a -1.10 (-2.23, 0.03) 0.05
Del Prato S 2003 -2.38 (-3.00, -1.76) 0.16
Chiasson JL 2001a -1.08 (-1.91, -0.25) 0.09
Wu GT 2001 -3.23 (-3.39, -3.07) 2.48
Lee A 1998 -1.60 (-3.58, 0.38) 0.02
Wang DL 2020a -3.43 (-3.91, -2.95) 0.27
Yang Y 2019 -1.29 (-1.95, -0.63) 0.14
Han SQ 2018 -1.35 (-1.97, -0.73) 0.16
Zhou RF 2017a -0.50 (-1.09, 0.09) 0.18
Bai N 2016 -2.90 (-3.92, -1.88) 0.06
Geng N 2016 -2.00 (-3.48, -0.52) 0.03
Guo DL 2014 -1.49 (-2.09, -0.89) 0.17
Guo W 2014 -1.86 (-2.54, -1.18) 0.13
Li L 2014a -1.98 (-4.43, 0.47) 0.01
Esteghamati A 2013 -1.52 (-2.16, -0.88) 0.15
Liu YT 2013 -2.80 (-3.61, -1.99) 0.09
Xi Y 2013a -0.50 (-1.04, 0.04) 0.21
Yang WC 2013 -0.72 (-1.40, -0.04) 0.13
Chen Y 2008a -1.80 (-3.23, -0.37) 0.03
Cao Y 2007 -1.90 (-2.86, -0.94) 0.07
Deng HO 2007a -0.75 (-1.64, 0.14) 0.08
Deng HO 2007b -0.85 (-1.68, -0.02) 0.09
Mei Q 2006 -0.61 (-1.29, 0.07) 0.13
Subtotal (I-squared = 96.5%, p = 0.000) -1.22 (-1.27, -1.17) 21.76
Sulfonylurea vs Placebo/lifestyle intervention
Hanefeld M 2002a -3.50 (-6.15, -0.85) 0.01
Birkeland KI 1994a -2.20 (-4.29, -0.11) 0.01
Takami K 2002a -1.90 (-3.78, -0.02) 0.02
Hassan MH 2015b -2.00 (-3.22, -0.78) 0.04
Kaku K 2013A -2.14 (-2.74, -1.54) 0.17
Yu JX 2014 -3.18 (-4.19, -2.17) 0.06
Hassan MH 2015c -2.22 (-3.47, -0.97) 0.04
Wei JC 2010 -1.90 (-2.68, -1.12) 0.10
Scott R 2007a -1.82 (-2.31, -1.33) 0.25
Simonson DC 1997a -3.17 (-4.45, -1.89) 0.04
Simonson DC 1997b -4.23 (-5.61, -2.85) 0.03
Simonson DC 1997c -3.29 (-4.80, -1.78) 0.03
Simonson DC 1997d -4.33 (-5.52, -3.14) 0.04
Simonson DC 1997e -4.07 (-5.70, -2.44) 0.02
Birkeland KI 1994b -1.90 (-3.90, 0.10) 0.02
Subtotal (I-squared = 60.2%, p = 0.001) -2.41 (-2.67, -2.14) 0.88
Thiazolidinedione vs Placebo/lifestyle intervention
Oz GO 2010a -1.69 (-2.41, -0.97) 0.12
Rahman S 2010 -0.30 (-3.20, 2.60) 0.01
Oz O 2008a -0.50 (-1.84, 0.84) 0.03
Albertini JP 2007 -2.70 (-3.42, -1.98) 0.12
Viljanen AP 2005b -0.30 (-1.17, 0.57) 0.08
Chen HY 2004 -2.36 (-2.71, -2.01) 0.50
Juhl CB 2003 -2.10 (-4.19, -0.01) 0.01
Carey DG 2002 -2.20 (-3.17, -1.23) 0.07
Haffner SM 2002a -2.70 (-3.39, -2.01) 0.13
Haffner SM 2002b -3.30 (-4.01, -2.59) 0.12
Lebovitz HE 2001a -3.16 (-3.87, -2.45) 0.12
Lebovitz HE 2001b -4.05 (-4.75, -3.35) 0.12
Miyazaki Y 2001 -2.50 (-3.98, -1.02) 0.03
Huang D 2016 0.20 (-0.60, 1.00) 0.10
Berberoglu Z 2010 -0.95 (-1.60, -0.30) 0.14
Lu XH 2009 -0.09 (-0.73, 0.55) 0.15
Zhu KS 2009 -1.20 (-1.77, -0.63) 0.19
Berberoglu Z 2007 -0.54 (-1.09, 0.01) 0.20
Wang J 2020 -0.98 (-1.76, -0.20) 0.10
Bajpeyi S 2017 -1.00 (-2.73, 0.73) 0.02
Tang XQ 2017 -0.52 (-1.53, 0.49) 0.06
Chou HS 2012 -1.95 (-2.29, -1.61) 0.54
Kong AP 2011 -2.01 (-2.79, -1.23) 0.10
Oz GO 2010b -1.37 (-2.21, -0.53) 0.09
Wang B 2009 -0.97 (-1.66, -0.28) 0.13
Oz O 2008b -1.64 (-3.23, -0.05) 0.02
Gastaldelli A 2007 -1.80 (-3.05, -0.55) 0.04
Goldstein BJ 2006 -2.12 (-2.91, -1.33) 0.10
Hao YR 2006a -2.14 (-3.13, -1.15) 0.06
Hao YR 2006b -2.49 (-4.31, -0.67) 0.02
Khan M 2006a -1.90 (-3.64, -0.16) 0.02
Khan M 2006b -2.95 (-4.69, -1.21) 0.02
Khan M 2006c -3.75 (-5.51, -1.99) 0.02
Pang WY 2006 -1.70 (-2.77, -0.63) 0.05
Wallace TM 2004 -1.20 (-1.75, -0.65) 0.20
Liu CM 2003 -2.55 (-3.92, -1.18) 0.03
Miyazaki Y 2002a -1.73 (-4.45, 0.99) 0.01
Miyazaki Y 2002b -3.73 (-7.15, -0.31) 0.01
Miyazaki Y 2002c -5.45 (-8.52, -2.38) 0.01
Scherbaum WA 2002a -2.04 (-2.88, -1.20) 0.09
Scherbaum WA 2002b -2.13 (-3.10, -1.16) 0.06
Aronoff S 2000a -2.16 (-5.70, 1.38) 0.00
Aronoff S 2000b -2.29 (-3.32, -1.26) 0.06
Aronoff S 2000c -3.63 (-4.68, -2.58) 0.06
Wang DL 2020b -3.25 (-3.70, -2.80) 0.30
Mirmiranpour H 2013 -1.64 (-2.63, -0.65) 0.06
Chen Y 2008b -2.20 (-3.73, -0.67) 0.03
Subtotal (I-squared = 83.7%, p = 0.000) -1.91 (-2.03, -1.79) 4.55
Glinide vs Placebo/lifestyle intervention
Bech P 2003 -1.30 (-1.82, -0.78) 0.23
Xu L 2011 -0.19 (-1.59, 1.21) 0.03
Zhong XY 2010 -0.19 (-1.59, 1.21) 0.03
Guo XD 2009 -0.19 (-1.59, 1.21) 0.03
Zhao W 2008 -0.19 (-1.59, 1.21) 0.03
Gonzalez-Clemente JM 2008 -0.40 (-1.21, 0.41) 0.09
Schwarz SL 2008 -1.39 (-2.33, -0.45) 0.07
Mari A 2005a -0.50 (-1.18, 0.18) 0.13
Mari A 2005b -0.95 (-1.62, -0.28) 0.14
Kato T 2010a -0.43 (-1.20, 0.34) 0.10
Subtotal (I-squared = 16.1%, p = 0.295) -0.78 (-1.04, -0.52) 0.89
α-Glucosidase inhibitor vs Placebo/lifestyle intervention
Kirkman MS 2006 -0.17 (-0.46, 0.12) 0.71
Josse RG 2003 -0.70 (-1.25, -0.15) 0.20
Wu GT 2003 -3.19 (-3.29, -3.09) 6.41
Hanefeld M 2002b -1.80 (-4.40, 0.80) 0.01
Meneilly GS 2000 -0.70 (-1.41, 0.01) 0.12
Scott R 1999 -1.36 (-2.16, -0.56) 0.10
Chan JCN 1998 -0.78 (-1.40, -0.16) 0.16
Braun D 1996 -1.30 (-2.36, -0.24) 0.05
Hou WK 1996 -1.20 (-1.67, -0.73) 0.28
Zheng GF 1995 -2.75 (-3.87, -1.63) 0.05
Hotta N 1993 -0.60 (-2.28, 1.08) 0.02
Santeusanio F 1993a -1.57 (-1.92, -1.22) 0.51
Santeusanio F 1993b -1.68 (-1.97, -1.39) 0.73
Chen DL 2017 -1.21 (-1.78, -0.64) 0.19
Yang LH 2015 -0.69 (-1.20, -0.18) 0.23
Wu HX 2014 -1.30 (-2.49, -0.11) 0.04
Yang Y 2014 -1.17 (-2.21, -0.13) 0.06
Hao HR 2012 -1.40 (-2.07, -0.73) 0.14
Kato T 2010b 0.04 (-0.77, 0.85) 0.09
Ye XH 2009 0.00 (-0.46, 0.46) 0.29
Seino Y 2011a -0.48 (-0.94, -0.02) 0.29
Guo HG 2001 -2.65 (-2.83, -2.47) 1.92
Takami K 2002b -2.40 (-4.60, -0.20) 0.01
Chiasson JL 2001b -0.01 (-0.88, 0.86) 0.08
Subtotal (I-squared = 97.6%, p = 0.000) -2.33 (-2.40, -2.26) 12.70

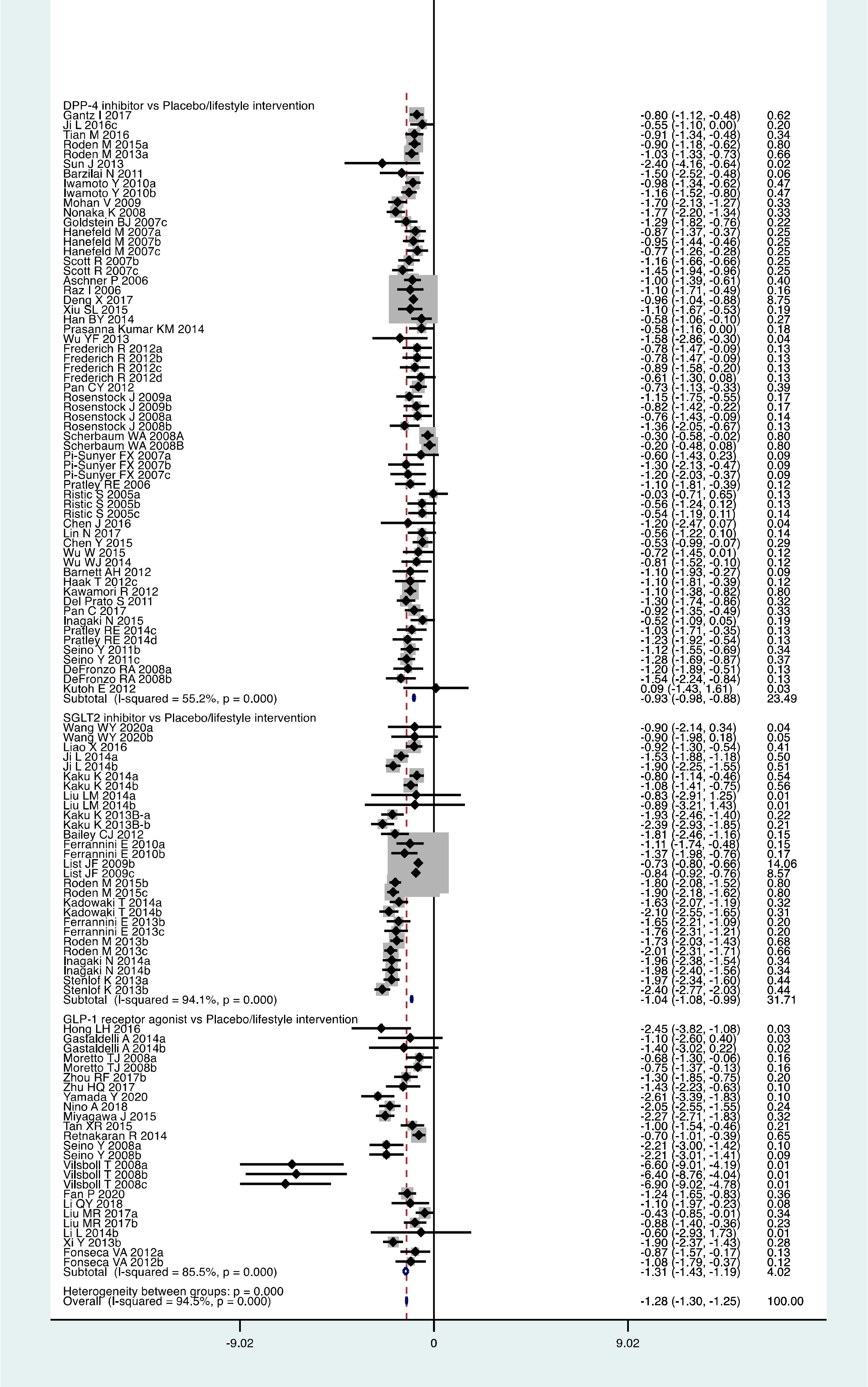

Figure S105. Sensitivity analysis results for change in fasting plasma glucose (mmol/l) of glucose-lowering drugs vs placebo/lifestyle intervention

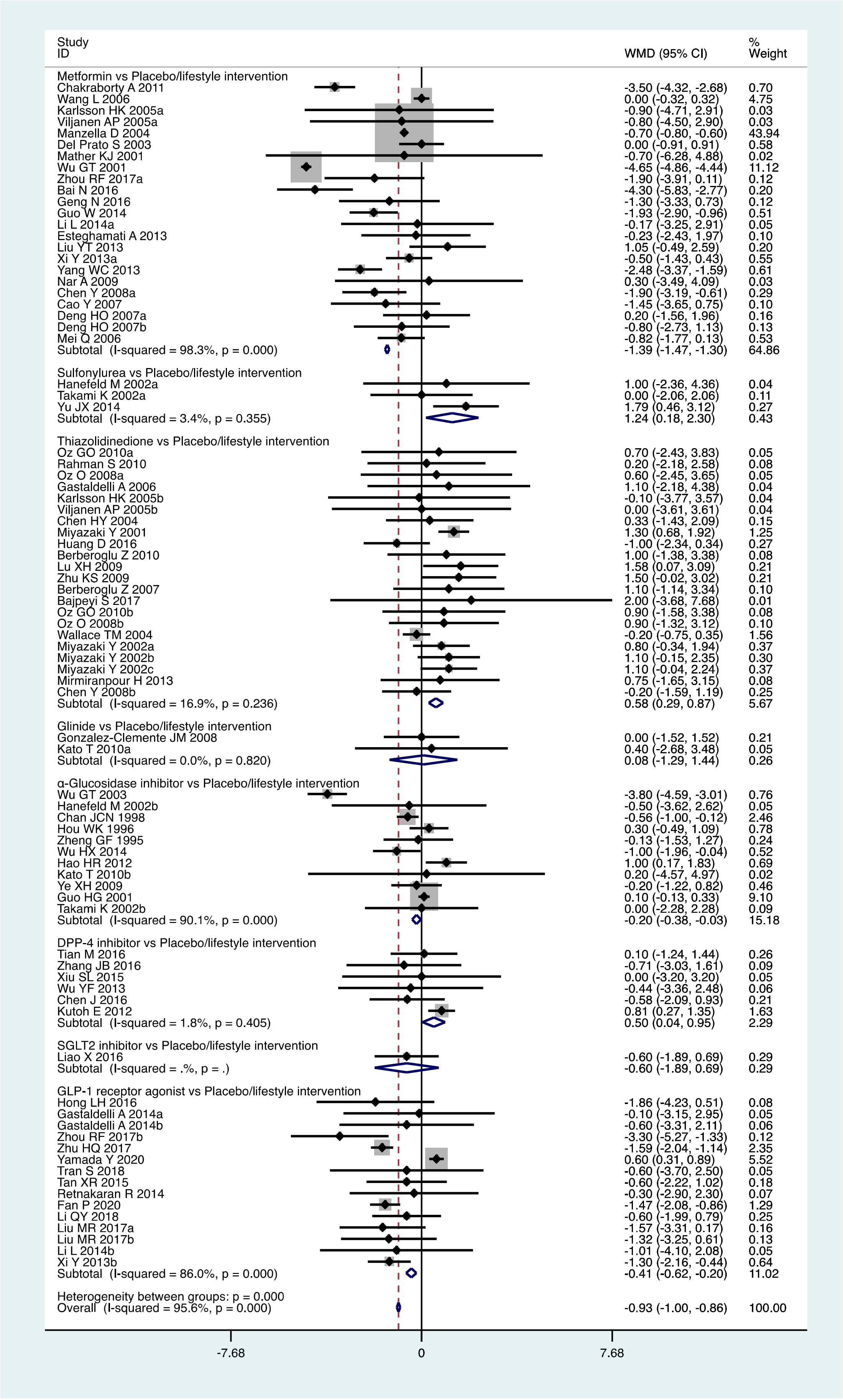

Figure S106. Sensitivity analysis results for change in body mass index (kg/m^2) of glucose-lowering drugs vs placebo/lifestyle intervention

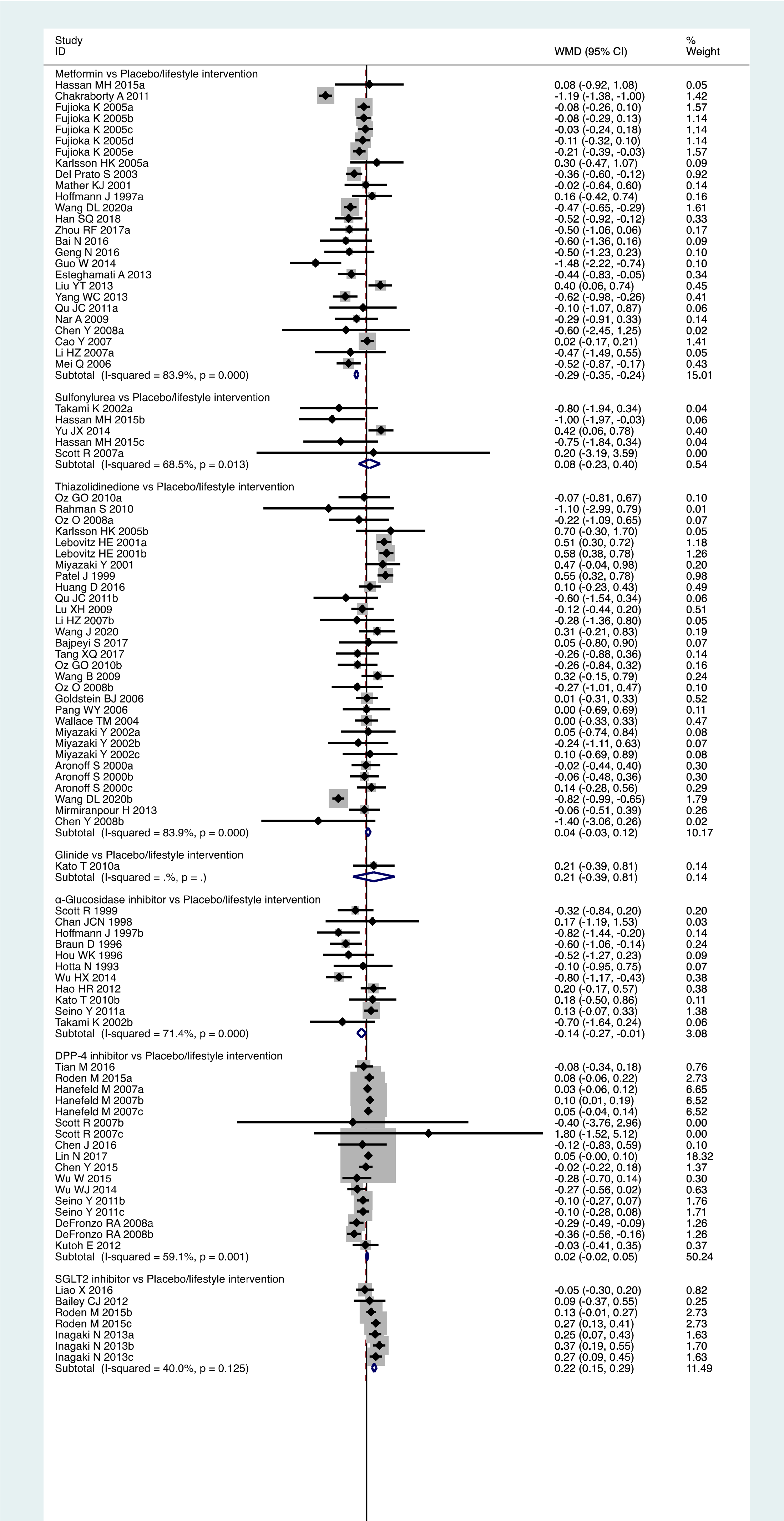
Study ID
WMD (95% CI)
% Weight
Metformin vs Placebo/lifestyle intervention
Hassan MH 2015a 0.08 (-0.92, 1.08) 0.05
Chakraborty A 2011 -1.19 (-1.38, -1.00) 1.42
Fujioka K 2005a -0.08 (-0.26, 0.10) 1.57
Fujioka K 2005b -0.08 (-0.29, 0.13) 1.14
Fujioka K 2005c -0.03 (-0.24, 0.18) 1.14
Fujioka K 2005d -0.11 (-0.32, 0.10) 1.14
Fujioka K 2005e -0.21 (-0.39, -0.03) 1.57
Karlsson HK 2005a 0.30 (-0.47, 1.07) 0.09
Del Prato S 2003 -0.36 (-0.60, -0.12) 0.92
Mather KJ 2001 -0.02 (-0.64, 0.60) 0.14
Hoffmann J 1997a 0.16 (-0.42, 0.74) 0.16
Wang DL 2020a -0.47 (-0.65, -0.29) 1.61
Han SQ 2018 -0.52 (-0.92, -0.12) 0.33
Zhou RF 2017a -0.50 (-1.06, 0.06) 0.17
Bai N 2016 -0.60 (-1.36, 0.16) 0.09
Geng N 2016 -0.50 (-1.23, 0.23) 0.10
Guo W 2014 -1.48 (-2.22, -0.74) 0.10
Esteghamati A 2013 -0.44 (-0.83, -0.05) 0.34
Liu YT 2013 0.40 (0.06, 0.74) 0.45
Yang WC 2013 -0.62 (-0.98, -0.26) 0.41
Qu JC 2011a -0.10 (-1.07, 0.87) 0.06
Nar A 2009 -0.29 (-0.91, 0.33) 0.14
Chen Y 2008a -0.60 (-2.45, 1.25) 0.02
Cao Y 2007 0.02 (-0.17, 0.21) 1.41
Li HZ 2007a -0.47 (-1.49, 0.55) 0.05
Mei Q 2006 -0.52 (-0.87, -0.17) 0.43
Subtotal (I-squared = 83.9%, p = 0.000) -0.29 (-0.35, -0.24) 15.01
Sulfonylurea vs Placebo/lifestyle intervention
Takami K 2002a -0.80 (-1.94, 0.34) 0.04
Hassan MH 2015b -1.00 (-1.97, -0.03) 0.06
Yu JX 2014 0.42 (0.06, 0.78) 0.40
Hassan MH 2015c -0.75 (-1.84, 0.34) 0.04
Scott R 2007a 0.20 (-3.19, 3.59) 0.00
Subtotal (I-squared = 68.5%, p = 0.013) 0.08 (-0.23, 0.40) 0.54
Thiazolidinedione vs Placebo/lifestyle intervention
Oz GO 2010a -0.07 (-0.81, 0.67) 0.10
Rahman S 2010 -1.10 (-2.99, 0.79) 0.01
Oz O 2008a -0.22 (-1.09, 0.65) 0.07
Karlsson HK 2005b 0.70 (-0.30, 1.70) 0.05
Lebovitz HE 2001a 0.51 (0.30, 0.72) 1.18
Lebovitz HE 2001b 0.58 (0.38, 0.78) 1.26
Miyazaki Y 2001 0.47 (-0.04, 0.98) 0.20
Patel J 1999 0.55 (0.32, 0.78) 0.98
Huang D 2016 0.10 (-0.23, 0.43) 0.49
Qu JC 2011b -0.60 (-1.54, 0.34) 0.06
Lu XH 2009 -0.12 (-0.44, 0.20) 0.51
Li HZ 2007b -0.28 (-1.36, 0.80) 0.05
Wang J 2020 0.31 (-0.21, 0.83) 0.19
Bajpeyi S 2017 0.05 (-0.80, 0.90) 0.07
Tang XQ 2017 -0.26 (-0.88, 0.36) 0.14
Oz GO 2010b -0.26 (-0.84, 0.32) 0.16
Wang B 2009 0.32 (-0.15, 0.79) 0.24
Oz O 2008b -0.27 (-1.01, 0.47) 0.10
Goldstein BJ 2006 0.01 (-0.31, 0.33) 0.52
Pang WY 2006 0.00 (-0.69, 0.69) 0.11
Wallace TM 2004 0.00 (-0.33, 0.33) 0.47
Miyazaki Y 2002a 0.05 (-0.74, 0.84) 0.08
Miyazaki Y 2002b -0.24 (-1.11, 0.63) 0.07
Miyazaki Y 2002c 0.10 (-0.69, 0.89) 0.08
Aronoff S 2000a -0.02 (-0.44, 0.40) 0.30
Aronoff S 2000b -0.06 (-0.48, 0.36) 0.30
Aronoff S 2000c 0.14 (-0.28, 0.56) 0.29
Wang DL 2020b -0.82 (-0.99, -0.65) 1.79
Mirmiranpour H 2013 -0.06 (-0.51, 0.39) 0.26
Chen Y 2008b -1.40 (-3.06, 0.26) 0.02
Subtotal (I-squared = 83.9%, p = 0.000) 0.04 (-0.03, 0.12) 10.17
Glinide vs Placebo/lifestyle intervention
Kato T 2010a 0.21 (-0.39, 0.81) 0.14
Subtotal (I-squared = .%, p = .) 0.21 (-0.39, 0.81) 0.14
α-Glucosidase inhibitor vs Placebo/lifestyle intervention
Scott R 1999 -0.32 (-0.84, 0.20) 0.20
Chan JCN 1998 0.17 (-1.19, 1.53) 0.03
Hoffmann J 1997b -0.82 (-1.44, -0.20) 0.14
Braun D 1996 -0.60 (-1.06, -0.14) 0.24
Hou WK 1996 -0.52 (-1.27, 0.23) 0.09
Hotta N 1993 -0.10 (-0.95, 0.75) 0.07
Wu HX 2014 -0.80 (-1.17, -0.43) 0.38
Hao HR 2012 0.20 (-0.17, 0.57) 0.38
Kato T 2010b 0.18 (-0.50, 0.86) 0.11
Seino Y 2011a 0.13 (-0.07, 0.33) 1.38
Takami K 2002b -0.70 (-1.64, 0.24) 0.06
Subtotal (I-squared = 71.4%, p = 0.000) -0.14 (-0.27, -0.01) 3.08
DPP-4 inhibitor vs Placebo/lifestyle intervention
Tian M 2016 -0.08 (-0.34, 0.18) 0.76
Roden M 2015a 0.08 (-0.06, 0.22) 2.73
Hanefeld M 2007a 0.03 (-0.06, 0.12) 6.65
Hanefeld M 2007b 0.10 (0.01, 0.19) 6.52
Hanefeld M 2007c 0.05 (-0.04, 0.14) 6.52
Scott R 2007b -0.40 (-3.76, 2.96) 0.00
Scott R 2007c 1.80 (-1.52, 5.12) 0.00
Chen J 2016 -0.12 (-0.83, 0.59) 0.10
Lin N 2017 0.05 (-0.00, 0.10) 18.32
Chen Y 2015 -0.02 (-0.22, 0.18) 1.37
Wu W 2015 -0.28 (-0.70, 0.14) 0.30
Wu WJ 2014 -0.27 (-0.56, 0.02) 0.63
Seino Y 2011b -0.10 (-0.27, 0.07) 1.76
Seino Y 2011c -0.10 (-0.28, 0.08) 1.71
DeFronzo RA 2008a -0.29 (-0.49, -0.09) 1.26
DeFronzo RA 2008b -0.36 (-0.56, -0.16) 1.26
Kutoh E 2012 -0.03 (-0.41, 0.35) 0.37
Subtotal (I-squared = 59.1%, p = 0.001) 0.02 (-0.02, 0.05) 50.24
SGLT2 inhibitor vs Placebo/lifestyle intervention
Liao X 2016 -0.05 (-0.30, 0.20) 0.82
Bailey CJ 2012 0.09 (-0.37, 0.55) 0.25
Roden M 2015b 0.13 (-0.01, 0.27) 2.73
Roden M 2015c 0.27 (0.13, 0.41) 2.73
Inagaki N 2013a 0.25 (0.07, 0.43) 1.63
Inagaki N 2013b 0.37 (0.19, 0.55) 1.70
Inagaki N 2013c 0.27 (0.09, 0.45) 1.63
Subtotal (I-squared = 40.0%, p = 0.125) 0.22 (0.15, 0.29) 11.49

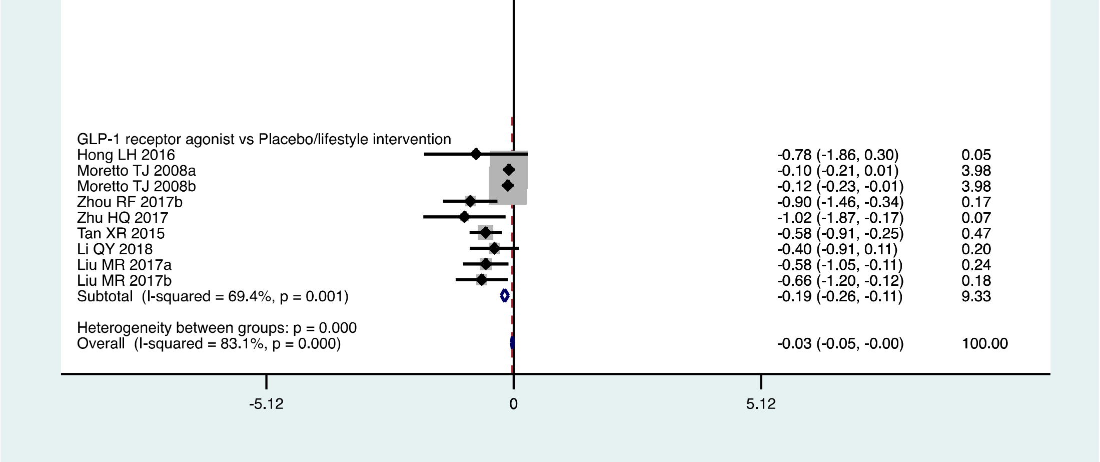

Figure S107. Sensitivity analysis results for change in total cholesterol (mmol/l) of glucose-lowering drugs vs placebo/lifestyle intervention

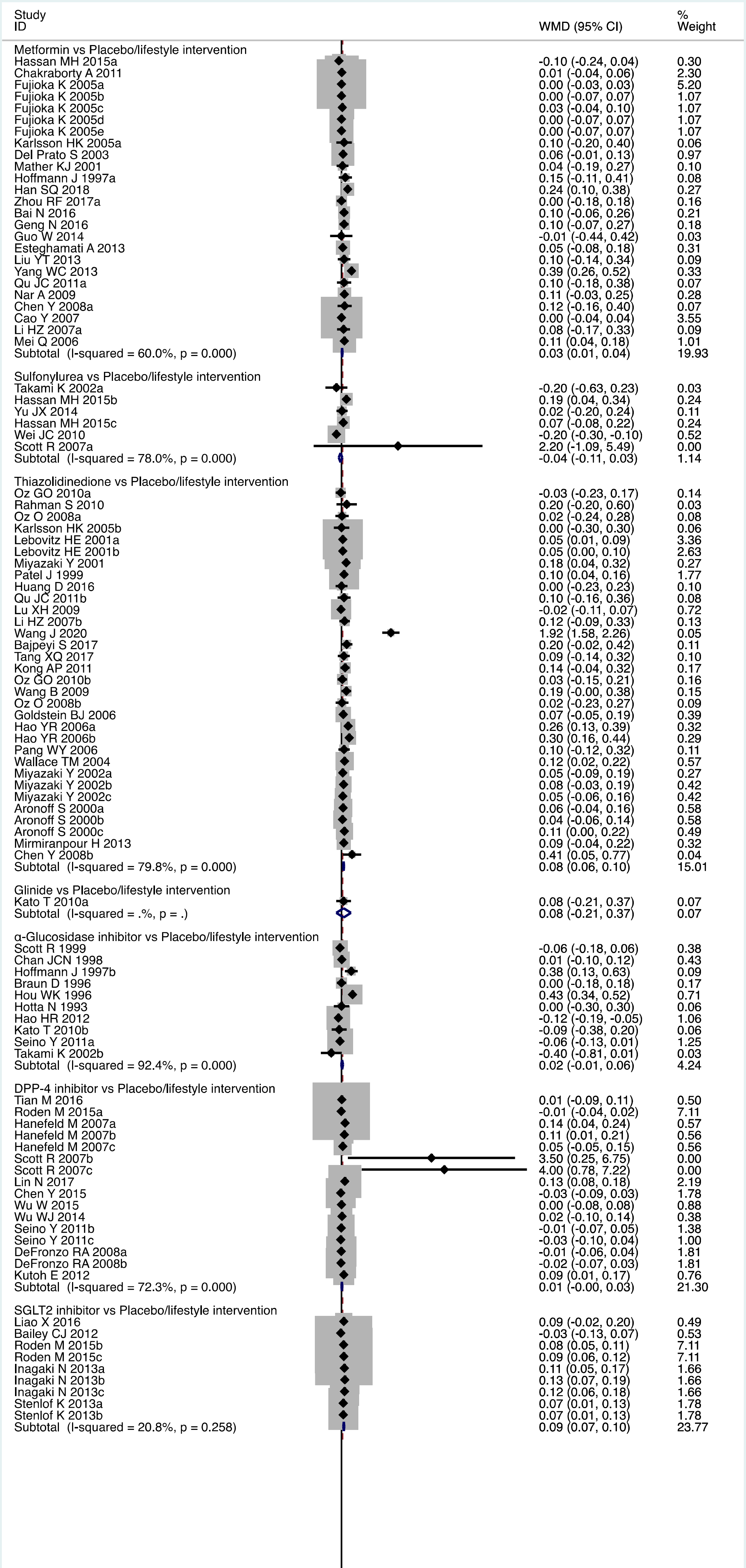
Study ID | WMD (95% CI) | % Weight
Metformin vs Placebo/lifestyle intervention
Hassan MH 2015a -0.10 (-0.24, 0.04) 0.30
Chakraborty A 2011 0.01 (-0.04, 0.06) 2.30
Fujioka K 2005a 0.00 (-0.03, 0.03) 5.20
Fujioka K 2005b 0.00 (-0.07, 0.07) 1.07
Fujioka K 2005c 0.03 (-0.04, 0.10) 1.07
Fujioka K 2005d 0.00 (-0.07, 0.07) 1.07
Fujioka K 2005e 0.00 (-0.07, 0.07) 1.07
Karlsson HK 2005a 0.10 (-0.20, 0.40) 0.06
Del Prato S 2003 0.06 (-0.01, 0.13) 0.97
Mather KJ 2001 0.04 (-0.19, 0.27) 0.10
Hoffmann J 1997a 0.15 (-0.11, 0.41) 0.08
Han SQ 2018 0.24 (0.10, 0.38) 0.27
Zhou RF 2017a 0.00 (-0.18, 0.18) 0.16
Bai N 2016 0.10 (-0.06, 0.26) 0.21
Geng N 2016 0.10 (-0.07, 0.27) 0.18
Guo W 2014 -0.01 (-0.44, 0.42) 0.03
Esteghamati A 2013 0.05 (-0.08, 0.18) 0.31
Liu YT 2013 0.10 (-0.14, 0.34) 0.09
Yang WC 2013 0.39 (0.26, 0.52) 0.33
Qu JC 2011a 0.10 (-0.18, 0.38) 0.07
Nar A 2009 0.11 (-0.03, 0.25) 0.28
Chen Y 2008a 0.12 (-0.16, 0.40) 0.07
Cao Y 2007 0.00 (-0.04, 0.04) 3.55
Li HZ 2007a 0.08 (-0.17, 0.33) 0.09
Mei Q 2006 0.11 (0.04, 0.18) 1.01
Subtotal (I-squared = 60.0%, p = 0.000) 0.03 (0.01, 0.04) 19.93
Sulfonylurea vs Placebo/lifestyle intervention
Takami K 2002a -0.20 (-0.63, 0.23) 0.03
Hassan MH 2015b 0.19 (0.04, 0.34) 0.24
Yu JX 2014 0.02 (-0.20, 0.24) 0.11
Hassan MH 2015c 0.07 (-0.08, 0.22) 0.24
Wei JC 2010 -0.20 (-0.30, -0.10) 0.52
Scott R 2007a 2.20 (-1.09, 5.49) 0.00
Subtotal (I-squared = 78.0%, p = 0.000) -0.04 (-0.11, 0.03) 1.14
Thiazolidinedione vs Placebo/lifestyle intervention
Oz GO 2010a -0.03 (-0.23, 0.17) 0.14
Rahman S 2010 0.20 (-0.20, 0.60) 0.03
Oz O 2008a 0.02 (-0.24, 0.28) 0.08
Karlsson HK 2005b 0.00 (-0.30, 0.30) 0.06
Lebovitz HE 2001a 0.05 (0.01, 0.09) 3.36
Lebovitz HE 2001b 0.05 (0.00, 0.10) 2.63
Miyazaki Y 2001 0.18 (0.04, 0.32) 0.27
Patel J 1999 0.10 (0.04, 0.16) 1.77
Huang D 2016 0.00 (-0.23, 0.23) 0.10
Qu JC 2011b 0.10 (-0.16, 0.36) 0.08
Lu XH 2009 -0.02 (-0.11, 0.07) 0.72
Li HZ 2007b 0.12 (-0.09, 0.33) 0.13
Wang J 2020 1.92 (1.58, 2.26) 0.05
Bajpeyi S 2017 0.20 (-0.02, 0.42) 0.11
Tang XQ 2017 0.09 (-0.14, 0.32) 0.10
Kong AP 2011 0.14 (-0.04, 0.32) 0.17
Oz GO 2010b 0.03 (-0.15, 0.21) 0.16
Wang B 2009 0.19 (-0.00, 0.38) 0.15
Oz O 2008b 0.02 (-0.23, 0.27) 0.09
Goldstein BJ 2006 0.07 (-0.05, 0.19) 0.39
Hao YR 2006a 0.26 (0.13, 0.39) 0.32
Hao YR 2006b 0.30 (0.16, 0.44) 0.29
Pang WY 2006 0.10 (-0.12, 0.32) 0.11
Wallace TM 2004 0.12 (0.02, 0.22) 0.57
Miyazaki Y 2002a 0.05 (-0.09, 0.19) 0.27
Miyazaki Y 2002b 0.08 (-0.03, 0.19) 0.42
Miyazaki Y 2002c 0.05 (-0.06, 0.16) 0.42
Aronoff S 2000a 0.06 (-0.04, 0.16) 0.58
Aronoff S 2000b 0.04 (-0.06, 0.14) 0.58
Aronoff S 2000c 0.11 (0.00, 0.22) 0.49
Mirmiranpour H 2013 0.09 (-0.04, 0.22) 0.32
Chen Y 2008b 0.41 (0.05, 0.77) 0.04
Subtotal (I-squared = 79.8%, p = 0.000) 0.08 (0.06, 0.10) 15.01
Glinide vs Placebo/lifestyle intervention
Kato T 2010a 0.08 (-0.21, 0.37) 0.07
Subtotal (I-squared = .%, p = .) 0.08 (-0.21, 0.37) 0.07
α-Glucosidase inhibitor vs Placebo/lifestyle intervention
Scott R 1999 -0.06 (-0.18, 0.06) 0.38
Chan JCN 1998 0.01 (-0.10, 0.12) 0.43
Hoffmann J 1997b 0.38 (0.13, 0.63) 0.09
Braun D 1996 0.00 (-0.18, 0.18) 0.17
Hou WK 1996 0.43 (0.34, 0.52) 0.71
Hotta N 1993 0.00 (-0.30, 0.30) 0.06
Hao HR 2012 -0.12 (-0.19, -0.05) 1.06
Kato T 2010b -0.09 (-0.38, 0.20) 0.06
Seino Y 2011a -0.06 (-0.13, 0.01) 1.25
Takami K 2002b -0.40 (-0.81, 0.01) 0.03
Subtotal (I-squared = 92.4%, p = 0.000) 0.02 (-0.01, 0.06) 4.24
DPP-4 inhibitor vs Placebo/lifestyle intervention
Tian M 2016 0.01 (-0.09, 0.11) 0.50
Roden M 2015a -0.01 (-0.04, 0.02) 7.11
Hanefeld M 2007a 0.14 (0.04, 0.24) 0.57
Hanefeld M 2007b 0.11 (0.01, 0.21) 0.56
Hanefeld M 2007c 0.05 (-0.05, 0.15) 0.56
Scott R 2007b 3.50 (0.25, 6.75) 0.00
Scott R 2007c 4.00 (0.78, 7.22) 0.00
Lin N 2017 0.13 (0.08, 0.18) 2.19
Chen Y 2015 -0.03 (-0.09, 0.03) 1.78
Wu W 2015 0.00 (-0.08, 0.08) 0.88
Wu WJ 2014 0.02 (-0.10, 0.14) 0.38
Seino Y 2011b -0.01 (-0.07, 0.05) 1.38
Seino Y 2011c -0.03 (-0.10, 0.04) 1.00
DeFronzo RA 2008a -0.01 (-0.06, 0.04) 1.81
DeFronzo RA 2008b -0.02 (-0.07, 0.03) 1.81
Kutoh E 2012 0.09 (0.01, 0.17) 0.76
Subtotal (I-squared = 72.3%, p = 0.000) 0.01 (-0.00, 0.03) 21.30
SGLT2 inhibitor vs Placebo/lifestyle intervention
Liao X 2016 0.09 (-0.02, 0.20) 0.49
Bailey CJ 2012 -0.03 (-0.13, 0.07) 0.53
Roden M 2015b 0.08 (0.05, 0.11) 7.11
Roden M 2015c 0.09 (0.06, 0.12) 7.11
Inagaki N 2013a 0.11 (0.05, 0.17) 1.66
Inagaki N 2013b 0.13 (0.07, 0.19) 1.66
Inagaki N 2013c 0.12 (0.06, 0.18) 1.66
Stenlof K 2013a 0.07 (0.01, 0.13) 1.78
Stenlof K 2013b 0.07 (0.01, 0.13) 1.78
Subtotal (I-squared = 20.8%, p = 0.258) 0.09 (0.07, 0.10) 23.77

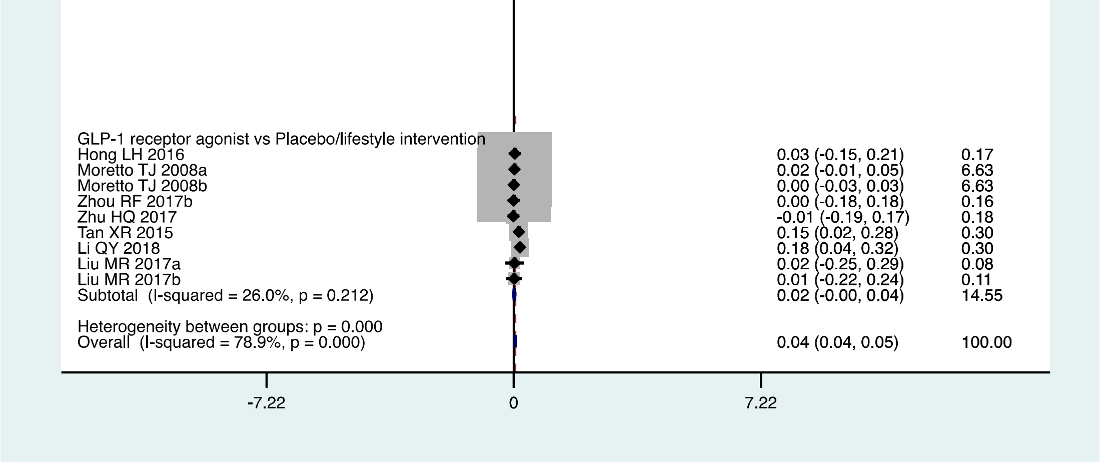

Figure S108. Sensitivity analysis results for change in high density lipoprotein-cholesterol (mmol/l) of glucose-lowering drugs vs placebo/lifestyle intervention

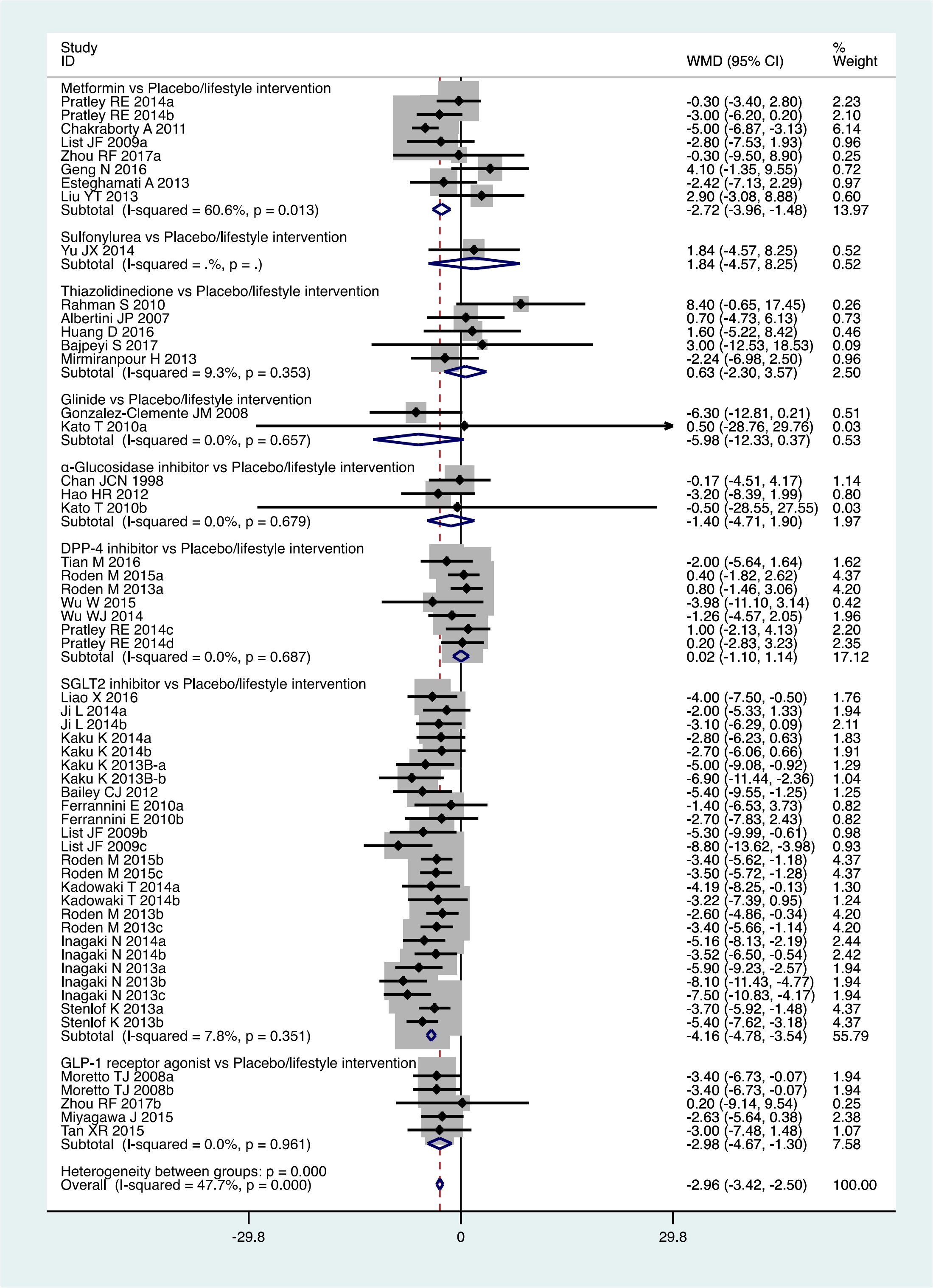

Figure S109. Sensitivity analysis results for change in systolic blood pressure (mmHg) of glucose-lowering drugs vs placebo/lifestyle intervention

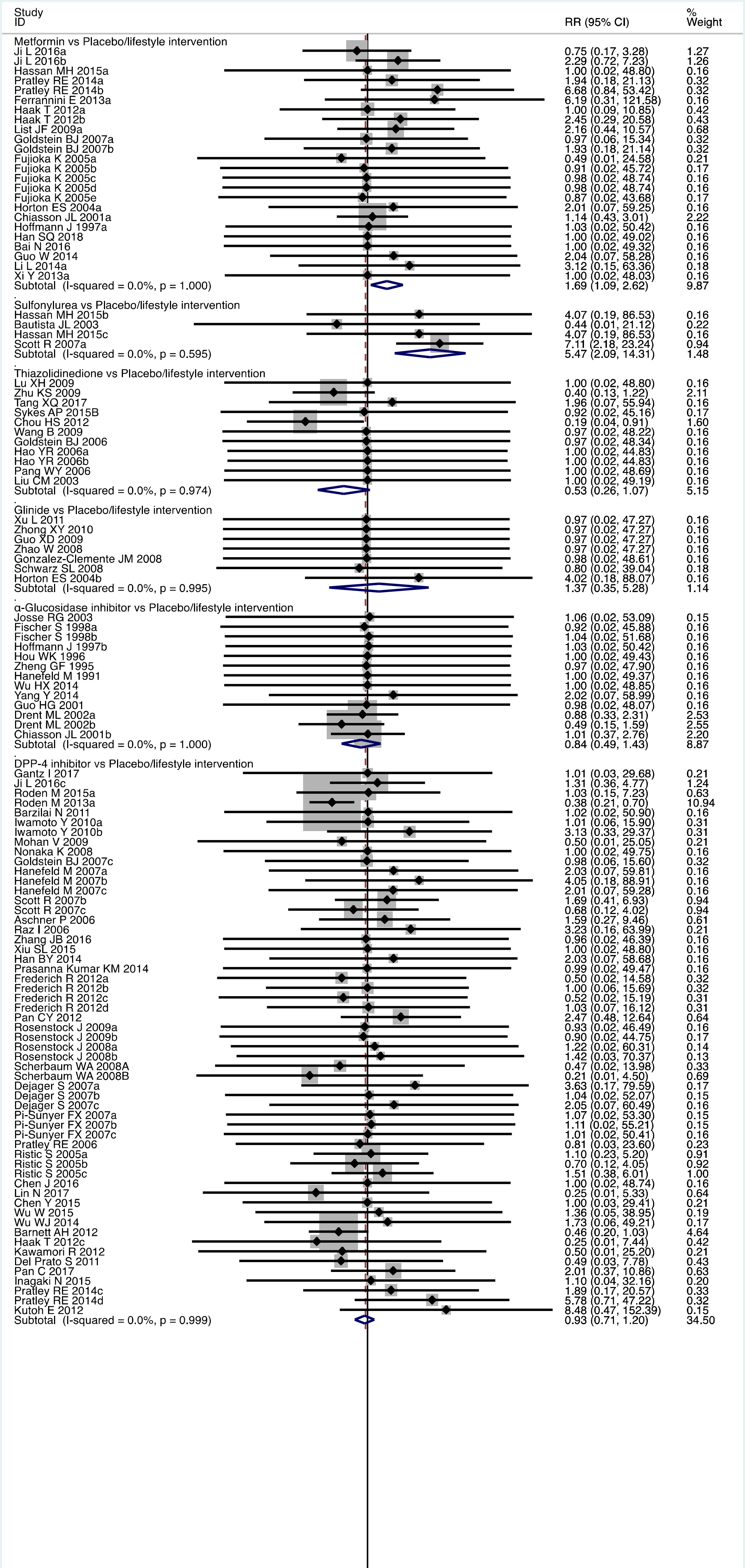
Study ID
RR (95% CI)
% Weight
Metformin vs Placebo/lifestyle intervention
Ji L 2016a 0.75 (0.17, 3.28) 1.27
Ji L 2016b 2.29 (0.72, 7.23) 1.26
Hassan MH 2015a 1.00 (0.02, 48.80) 0.16
Pratley RE 2014a 1.94 (0.18, 21.13) 0.32
Pratley RE 2014b 6.68 (0.84, 53.42) 0.32
Ferrannini E 2013a 6.19 (0.31, 121.58) 0.16
Haak T 2012a 1.00 (0.09, 10.85) 0.42
Haak T 2012b 2.45 (0.29, 20.58) 0.43
List JF 2009a 2.16 (0.44, 10.57) 0.68
Goldstein BJ 2007a 0.97 (0.06, 15.34) 0.32
Goldstein BJ 2007b 1.93 (0.18, 21.14) 0.32
Fujioka K 2005a 0.49 (0.01, 24.58) 0.21
Fujioka K 2005b 0.91 (0.02, 45.72) 0.17
Fujioka K 2005c 0.98 (0.02, 48.74) 0.16
Fujioka K 2005d 0.98 (0.02, 48.74) 0.16
Fujioka K 2005e 0.87 (0.02, 43.68) 0.17
Horton ES 2004a 2.01 (0.07, 59.25) 0.16
Chiasson JL 2001a 1.14 (0.43, 3.01) 2.22
Hoffmann J 1997a 1.03 (0.02, 50.42) 0.16
Han SQ 2018 1.00 (0.02, 49.02) 0.16
Bai N 2016 1.00 (0.02, 49.32) 0.16
Guo W 2014 2.04 (0.07, 58.28) 0.16
Li L 2014a 3.12 (0.15, 63.36) 0.18
Xi Y 2013a 1.00 (0.02, 48.03) 0.16
Subtotal (I-squared = 0.0%, p = 1.000) 1.69 (1.09, 2.62) 9.87
Sulfonylurea vs Placebo/lifestyle intervention
Hassan MH 2015b 4.07 (0.19, 86.53) 0.16
Bautista JL 2003 0.44 (0.01, 21.12) 0.22
Hassan MH 2015c 4.07 (0.19, 86.53) 0.16
Scott R 2007a 7.11 (2.18, 23.24) 0.94
Subtotal (I-squared = 0.0%, p = 0.595) 5.47 (2.09, 14.31) 1.48
Thiazolidinedione vs Placebo/lifestyle intervention
Lu XH 2009 1.00 (0.02, 48.80) 0.16
Zhu KS 2009 0.40 (0.13, 1.22) 2.11
Tang XQ 2017 1.96 (0.07, 55.94) 0.16
Sykes AP 2015B 0.92 (0.02, 45.16) 0.17
Chou HS 2012 0.19 (0.04, 0.91) 1.60
Wang B 2009 0.97 (0.02, 48.22) 0.16
Goldstein BJ 2006 0.97 (0.02, 48.34) 0.16
Hao YR 2006a 1.00 (0.02, 44.83) 0.16
Hao YR 2006b 1.00 (0.02, 44.83) 0.16
Pang WY 2006 1.00 (0.02, 48.69) 0.16
Liu CM 2003 1.00 (0.02, 49.19) 0.16
Subtotal (I-squared = 0.0%, p = 0.974) 0.53 (0.26, 1.07) 5.15
Glinide vs Placebo/lifestyle intervention
Xu L 2011 0.97 (0.02, 47.27) 0.16
Zhong XY 2010 0.97 (0.02, 47.27) 0.16
Guo XD 2009 0.97 (0.02, 47.27) 0.16
Zhao W 2008 0.97 (0.02, 47.27) 0.16
Gonzalez-Clemente JM 2008 0.98 (0.02, 48.61) 0.16
Schwarz SL 2008 0.80 (0.02, 39.04) 0.18
Horton ES 2004b 4.02 (0.18, 88.07) 0.16
Subtotal (I-squared = 0.0%, p = 0.995) 1.37 (0.35, 5.28) 1.14
α-Glucosidase inhibitor vs Placebo/lifestyle intervention
Josse RG 2003 1.06 (0.02, 53.09) 0.15
Fischer S 1998a 0.92 (0.02, 45.88) 0.16
Fischer S 1998b 1.04 (0.02, 51.68) 0.16
Hoffmann J 1997b 1.03 (0.02, 50.42) 0.16
Hou WK 1996 1.00 (0.02, 49.43) 0.16
Zheng GF 1995 0.97 (0.02, 47.90) 0.16
Hanefeld M 1991 1.00 (0.02, 49.37) 0.16
Wu HX 2014 1.00 (0.02, 48.85) 0.16
Yang Y 2014 2.02 (0.07, 58.99) 0.16
Guo HG 2001 0.98 (0.02, 48.07) 0.16
Drent ML 2002a 0.88 (0.33, 2.31) 2.53
Drent ML 2002b 0.49 (0.15, 1.59) 2.55
Chiasson JL 2001b 1.01 (0.37, 2.76) 2.20
Subtotal (I-squared = 0.0%, p = 1.000) 0.84 (0.49, 1.43) 8.87
DPP-4 inhibitor vs Placebo/lifestyle intervention
Gantz I 2017 1.01 (0.03, 29.68) 0.21
Ji L 2016c 1.31 (0.36, 4.77) 1.24
Roden M 2015a 1.03 (0.15, 7.23) 0.63
Roden M 2013a 0.38 (0.21, 0.70) 10.94
Barzilai N 2011 1.02 (0.02, 50.90) 0.16
Iwamoto Y 2010a 1.01 (0.06, 15.90) 0.31
Iwamoto Y 2010b 3.13 (0.33, 29.37) 0.31
Mohan V 2009 0.50 (0.01, 25.05) 0.21
Nonaka K 2008 1.00 (0.02, 49.75) 0.16
Goldstein BJ 2007c 0.98 (0.06, 15.60) 0.32
Hanefeld M 2007a 2.03 (0.07, 59.81) 0.16
Hanefeld M 2007b 4.05 (0.18, 88.91) 0.16
Hanefeld M 2007c 2.01 (0.07, 59.28) 0.16
Scott R 2007b 1.69 (0.41, 6.93) 0.94
Scott R 2007c 0.68 (0.12, 4.02) 0.94
Aschner P 2006 1.59 (0.27, 9.46) 0.61
Raz I 2006 3.23 (0.16, 63.99) 0.21
Zhang JB 2016 0.96 (0.02, 46.39) 0.16
Xiu SL 2015 1.00 (0.02, 48.80) 0.16
Han BY 2014 2.03 (0.07, 58.68) 0.16
Prasanna Kumar KM 2014 0.99 (0.02, 49.47) 0.16
Frederich R 2012a 0.50 (0.02, 14.58) 0.32
Frederich R 2012b 1.00 (0.06, 15.69) 0.32
Frederich R 2012c 0.52 (0.02, 15.19) 0.31
Frederich R 2012d 1.03 (0.07, 16.12) 0.31
Pan CY 2012 2.47 (0.48, 12.64) 0.64
Rosenstock J 2009a 0.93 (0.02, 46.49) 0.16
Rosenstock J 2009b 0.90 (0.02, 44.75) 0.17
Rosenstock J 2008a 1.22 (0.02, 60.31) 0.14
Rosenstock J 2008b 1.42 (0.03, 70.37) 0.13
Scherbaum WA 2008A 0.47 (0.02, 13.98) 0.33
Scherbaum WA 2008B 0.21 (0.01, 4.50) 0.69
Dejager S 2007a 3.63 (0.17, 79.59) 0.17
Dejager S 2007b 1.04 (0.02, 52.07) 0.15
Dejager S 2007c 2.05 (0.07, 60.49) 0.16
Pi-Sunyer FX 2007a 1.07 (0.02, 53.30) 0.15
Pi-Sunyer FX 2007b 1.11 (0.02, 55.21) 0.15
Pi-Sunyer FX 2007c 1.01 (0.02, 50.41) 0.16
Pratley RE 2006 0.81 (0.03, 23.60) 0.23
Ristic S 2005a 1.10 (0.23, 5.20) 0.91
Ristic S 2005b 0.70 (0.12, 4.05) 0.92
Ristic S 2005c 1.51 (0.38, 6.01) 1.00
Chen J 2016 1.00 (0.02, 48.74) 0.16
Lin N 2017 0.25 (0.01, 5.33) 0.64
Chen Y 2015 1.00 (0.03, 29.41) 0.21
Wu W 2015 1.36 (0.05, 38.95) 0.19
Wu WJ 2014 1.73 (0.06, 49.21) 0.17
Barnett AH 2012 0.46 (0.20, 1.03) 4.64
Haak T 2012c 0.25 (0.01, 7.44) 0.42
Kawamori R 2012 0.50 (0.01, 25.20) 0.21
Del Prato S 2011 0.49 (0.03, 7.78) 0.43
Pan C 2017 2.01 (0.37, 10.86) 0.63
Inagaki N 2015 1.10 (0.04, 32.16) 0.20
Pratley RE 2014c 1.89 (0.17, 20.57) 0.33
Pratley RE 2014d 5.78 (0.71, 47.22) 0.32
Kutoh E 2012 8.48 (0.47, 152.39) 0.15
Subtotal (I-squared = 0.0%, p = 0.999) 0.93 (0.71, 1.20) 34.50

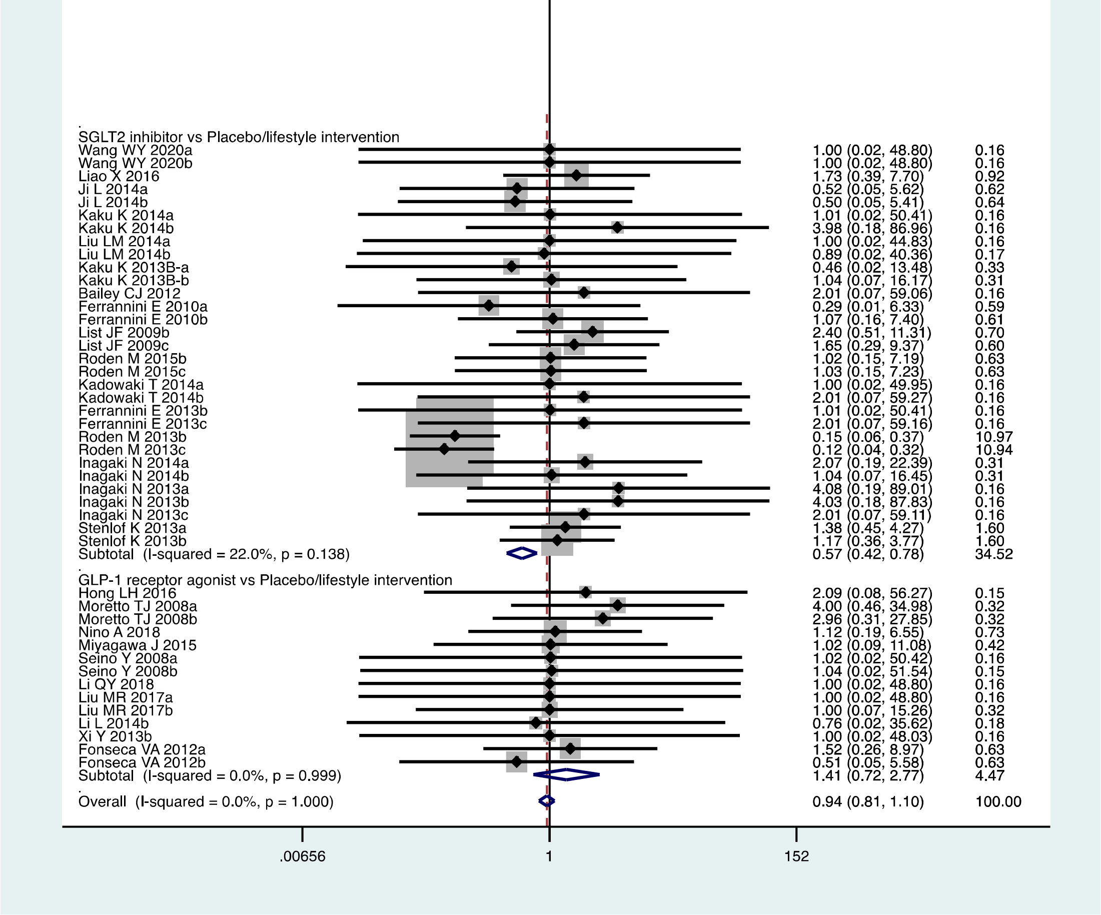

Figure S110. Sensitivity analysis results for incidence of hypoglycemia of glucose-lowering drugs vs placebo/lifestyle intervention

Reference

1. Ji L, Han P, Wang X, et al. Randomized clinical trial of the safety and efficacy of sitagliptin and metformin co-administered to Chinese patients with type 2 diabetes mellitus. *Journal of Diabetes Investigation* 2016;7(5):727-36.
2. Hassan MH, Abd-Allah GM. Effects of metformin plus gliclazide versus metformin plus glimepiride on cardiovascular risk factors in patients with type 2 diabetes mellitus. *Pak J Pharm Sci* 2015;28(5):1723-30.
3. Pratley RE, Fleck P, Wilson C. Efficacy and safety of initial combination therapy with alogliptin plus metformin versus either as monotherapy in drug-naive patients with type 2 diabetes: a randomized, double-blind, 6-month study. *Diabetes Obesity & Metabolism* 2014;16(7):613-21.
4. Ferrannini E, Seman L, Seewaldt-Becker E, et al. A Phase IIb, randomized, placebo-controlled study of the SGLT2 inhibitor empagliflozin in patients with type 2 diabetes. *Diabetes Obes Metab* 2013;15(8):721-8.
5. Haak T, Meinicke T, Jones R, et al. Initial combination of linagliptin and metformin improves glycaemic control in type 2 diabetes: a randomized, double-blind, placebo-controlled study. *Diabetes Obesity & Metabolism* 2012;14(6):565-74.
6. Chakraborty A, Chowdhury S, Bhattacharyya M. Effect of metformin on oxidative stress, nitrosative stress and inflammatory biomarkers in type 2 diabetes patients. *Diabetes Res Clin Pract* 2011;93(1):56-62.
7. List JF, Woo V, Morales E, Tang W, Fiedorek FT. Sodium-glucose cotransport inhibition with dapagliflozin in type 2 diabetes. *Diabetes Care* 2009;32(4):650-7.
8. Goldstein BJ, Feinglos MN, Lunceford JK, Johnson J, Williams-Herman DE. Effect of initial combination therapy with sitagliptin, a dipeptidyl peptidase-4 inhibitor, and metformin on glycemic control in patients with type 2 diabetes. *Diabetes Care* 2007;30(8):1979-87.
9. Wang L, Ning J. Effects of metformin on C-reactive protein and complement factor C3 in patients of obese type 2 diabetes mellitus. *Shanxi Medical Journal* 2006;35(10):888-89.
10. Fujioka K, Brazg RL, Raz I, et al. Efficacy, dose-response relationship and safety of once-daily extended-release metformin (Glucophage® XR) in type 2 diabetic patients with inadequate glycaemic control despite prior treatment with diet and exercise: Results from two double-blind, placebo-controlled studies. *Diabetes, Obesity and Metabolism* 2005;7(1):28-39.
11. Karlsson HK, Hallsten K, Bjornholm M, et al. Effects of metformin and rosiglitazone treatment on insulin signaling and glucose uptake in patients with newly diagnosed type 2 diabetes: a randomized controlled study. *Diabetes* 2005;54(5):1459-67. doi: 10.2337/diabetes.54.5.1459 [published Online First: 2005/04/28]
12. Viljanen AP, Virtanen KA, Jarvisalo MJ, et al. Rosiglitazone treatment increases subcutaneous adipose tissue glucose uptake in parallel with perfusion in patients with type 2 diabetes: a double-blind, randomized study with metformin. *J Clin Endocrinol Metab* 2005;90(12):6523-8.
13. Horton ES, Foley JE, Shen SG, Baron MA. Efficacy and tolerability of initial combination therapy with nateglinide and metformin in treatment-naive patients with type 2 diabetes. *Curr Med Res Opin* 2004;20(6):883-9.
14. Manzella D, Grella R, Esposito K, et al. Blood pressure and cardiac autonomic nervous system in obese type 2 diabetic patients: effect of metformin administration. *Am J Hypertens* 2004;17(3):223-7.
15. Del Prato S, Erkelens DW, Leutenegger M. Six-month efficacy of benfluorex vs. placebo or metformin in diet-failed type 2 diabetic patients. *Acta Diabetol* 2003;40(1):20-7.
16. Chiasson JL, Naditch L. The synergistic effect of miglitol plus metformin combination therapy in the treatment of type 2 diabetes. *Diabetes Care* 2001;24(6):989-94.
17. Mather KJ, Verma S, Anderson TJ. Improved endothelial function with metformin in type 2 diabetes mellitus. *J Am Coll Cardiol* 2001;37(5):1344-50.
18. Wu GT, Han YQ, Wang JY, et al. Effects of Glucophage on insulin resistance in type 2 diabetes mellitus patients. *Journal of Tongji University (Medical Science)* 2001;22(6):31-32+35.
19. Lee A, Morley JE. Metformin decreases food consumption and induces weight loss in subjects with obesity with type II non-insulin-dependent diabetes. *Obes Res* 1998;6(1):47-53.
20. Hoffmann J, Spengler M. Efficacy of 24-week monotherapy with acarbose, metformin, or placebo in dietary-treated NIDDM patients: the Essen-II Study. *Am J Med* 1997;103(6):483-90.
21. Wang DL, Xian B. Effect of pioglitazone and metformin on patients with incipient type 2 diabetes and nonalcoholic fatty liver disease. *Chinese Baby* 2020(4):122.
22. Yang Y. Application study of metformin combined with individualized diet and exercise therapy in elderly patients with type 2 diabetes. *Diet Health* 2019;6(7):45-46.
23. Han SQ, Liu R, Liu JX, Geng YQ. Effects of metformin on hepatic inflammation, liver fibrosis and insulin resistance in patients with type 2 diabetes mellitus complicated with nonalcoholic fatty liver disease. *Hebei Medical Journal* 2018;40(12):1769-73.
24. Zhou RF, Zhong S, Guo Y, Sun YJ, Shao ZX. Impact of exenatide combined with metformin on islet function in new-onset type 2 diabetes mellitus. *Clinical Focus* 2017;32(11):965-68.
25. Bai N. The efficacy and side effects of metformin in the treatment of type 2 diabetes mellitus complicated with nonalcoholic fatty liver disease. *China Health Standard Management* 2016;7(24):78-80.
26. Geng N, Qi H, Liu JQ. Efficacy of rosuvastatin in male patients with newly diagnosed type 2 diabetes and hyperlipidemia and its effect on serum vaspin level. *Hebei Medical Journal* 2016;38(18):2775-77,81.
27. Guo DL. Analysis of the efficacy of metformin in the treatment of type 2 diabetes. *Diabetes New World* 2014(11):1-2.
28. Guo W, Gao MS, Ye ZH, et al. Effects of metformin on the serum nesfatin-1 and liver steatosis in type 2 diabetic patients with nonalcoholic fatty liver disease. *Chinese Journal of Difficult and Complicated Cases* 2014(4):374-77.
29. Li L, Zhu KS, Qu JC, Xia AX, Zhang W. Clinical effect of liraglutide and metformin hydrochioride on overweight diabetic patients with poor glycemic control. *Clinical Medicine of China* 2014;30(1):67-69.

30. Esteghamati A, Eskandari D, Mirmiranpour H, et al. Effects of metformin on markers of oxidative stress and antioxidant reserve in patients with newly diagnosed type 2 diabetes: A randomized clinical trial. *Clinical Nutrition* 2013;32(2):179-85.
31. Liu YT, Hu B, Jian L, Li JW, Huang L. Influence of metformin on the level of serum vaspin in T2DM patients. *Chinese Journal of Diabetes* 2013;21(8):705-08.
32. Xi Y. Efficacy of liraglutide in patients with new-onset type 2 diabetes and obesity and its impact on patients' micro-inflammation. *Chinese General Practice* 2013;16(28):3339-40,45.
33. Yang WC, Dang Y, Qiao L, et al. Comparison of efficacy of exercise therapy and metformin in patients with newly diagnosed type 2 diabetes. *Journal of Henan Normal University (Natural Science Edition)* 2013;41(6):122-26.
34. Qu JC, Zhu KS, Wang T, Li L, Zhao L. Effect of rosiglitazone and metformin on lipid metabolism in patients with type 2 diabetes. *China Medicine* 2011;6(3):286-87.
35. Nar A, Gedik O. The effect of metformin on leptin in obese patients with type 2 diabetes mellitus and nonalcoholic fatty liver disease. *Acta Diabetol* 2009;46(2):113-8.
36. Chen Y. Study on the plasma visfatin level in newly diagnosed obese type 2 diabetes patients and the influence of pioglitazone on it. Guangdong Medical University, 2008.
37. Cao Y. Clinical study on multiple factor interventions in improving the insulin resistance in type 2 diabetes. Shandong University Of Traditional Chinese Medicine, 2007.
38. Deng HO, Lin K, Li DF, Li YL. Effect of metformin on serum testosterone in male patients with type 2 diabetes. *Guangdong Medical Journal* 2007;28(4):601-02.
39. Li HZ, Zhang C, Wang M, Liu XH. Effect of metformin and rosiglitazone maleate on lipid metabolism in patients with type 2 diabetes. *Journal of Clinical Research* 2007;24(2):319-21.
40. Mei Q. Therapeutic effect of metformin on type 2 diabetes mellitus complicated with nonalcoholic fatty liver disease. *Practical Pharmacy and Clinical Remedies* 2006;9(6):346-47.
41. Hanefeld M, Haffner SM, Menschikowski M, et al. Different effects of acarbose and glibenclamide on proinsulin and insulin profiles in people with type 2 diabetes. *Diabetes Res Clin Pract* 2002;55(3):221-7.
42. Birkeland KI, Furuseth K, Melander A, Mowinckel P, Vaaler S. Long-term randomized placebo-controlled double-blind therapeutic comparison of glipizide and glyburide. Glycemic control and insulin secretion during 15 months. *Diabetes Care* 1994;17(1):45-9.
43. Takami K, Takeda N, Nakashima K, et al. Effects of dietary treatment alone or diet with voglibose or glyburide on abdominal adipose tissue and metabolic abnormalities in patients with newly diagnosed type 2 diabetes. *Diabetes Care* 2002;25(4):658-62.
44. Kaku K, Araki T, Yoshinaka R. Randomized, double-blind, dose-ranging study of TAK-875, a novel GPR40 agonist, in Japanese patients with inadequately controlled type 2 diabetes. *Diabetes Care* 2013;36(2):245-50.
45. Bautista JL, Bugos C, Dirnberger G, Atherton T. Efficacy and safety profile of glimepiride in Mexican American patients with type 2 diabetes mellitus: A randomized, placebo-controlled study. *Clinical Therapeutics* 2003;25(1):194-209.
46. Yu JX, Wu Q, Zhou LL. Efficacy of simvastatin in treatment of patients with type 2 diabetes and dyslipidemia and its effect on serum vaspin level. *Chinese Journal of Gerontology* 2014(23):6588-89.
47. Wei JC, Ruan DJ, Meng XZ, Qu SH, Zheng WW. Effect of glipizide on blood glucose and blood lipids in patients with type 2 diabetes. *Progress in Modern Biomedicine* 2010;10(16):3067-69.
48. Scott R, Wu M, Sanchez M, Stein P. Efficacy and tolerability of the dipeptidyl peptidase-4 inhibitor sitagliptin as monotherapy over 12 weeks in patients with type 2 diabetes. *International Journal of Clinical Practice* 2007;61(1):171-80. doi: 10.1111/j.1742-1241.2006.01246.x
49. Simonson DC, Kourides IA, Feinglos M, et al. Efficacy, safety, and dose-response characteristics of glipizide gastrointestinal therapeutic system on glycemic control and insulin secretion in NIDDM - Results of two multicenter, randomized, placebo-controlled clinical trials. *Diabetes Care* 1997;20(4):597-606.
50. Oz GO, Tuncel E, Yilmaz Y, et al. Comparative effects of pioglitazone and rosiglitazone on plasma levels of soluble receptor for advanced glycation end products in type 2 diabetes mellitus patients. *Metabolism* 2010;59(1):64-9.
51. Rahman S, Ismail AAS, Ismail SB, Naing NN, Rahman ARA. Effect of rosiglitazone and ramipril on macrovasculopathy in patients with type 2 diabetes: Needs longer treatment and/or higher doses? *Clinical Pharmacology* 2010;2:83-87.
52. Oz O, Tuncel E, Eryilmaz S, et al. Arterial elasticity and plasma levels of adiponectin and leptin in type 2 diabetic patients treated with thiazolidinediones. *Endocrine* 2008;33(1):101-5.
53. Albertini JP, McMorn SO, Chen H, Mather RA, Valensi P. Effect of rosiglitazone on factors related to endothelial dysfunction in patients with type 2 diabetes mellitus. *Atherosclerosis* 2007;195(1):e159-66.
54. Gastaldelli A, Miyazaki Y, Pettiti M, et al. The effect of rosiglitazone on the liver: decreased gluconeogenesis in patients with type 2 diabetes. *J Clin Endocrinol Metab* 2006;91(3):806-12.
55. Chen HY, Zhang Y, Wu Y. Effects of rosiglitazone on C-reactive protein in patients with type 2 diabetes. *Journal of Jilin University (Medicine Edition)* 2004;30(3):465-67.
56. Juhl CB, Hollingdal M, Pørksen N, et al. Influence of rosiglitazone treatment on β-cell function in type 2 diabetes: Evidence of an increased ability of glucose to entrain high-frequency insulin pulsatility. *Journal of Clinical Endocrinology and Metabolism* 2003;88(8):3794-800.
57. Carey DG, Cowin GJ, Galloway GJ, et al. Effect of rosiglitazone on insulin sensitivity and body composition in type 2 diabetic patients [corrected]. *Obes Res* 2002;10(10):1008-15.
58. Haffner SM, Greenberg AS, Weston WM, et al. Effect of rosiglitazone treatment on nontraditional markers of cardiovascular disease in patients with type 2 diabetes mellitus. *Circulation* 2002;106(6):679-84.

59. Lebovitz HE, Dole JF, Patwardhan R, Rappaport EB, Freed MI. Rosiglitazone monotherapy is effective in patients with type 2 diabetes. *J Clin Endocrinol Metab* 2001;86(1):280-8.
60. Miyazaki Y, Glass L, Triplitt C, et al. Effect of rosiglitazone on glucose and non-esterified fatty acid metabolism in Type II diabetic patients. *Diabetologia* 2001;44(12):2210-9.
61. Patel J, Anderson RJ, Rappaport EB. Rosiglitazone monotherapy improves glycaemic control in patients with type 2 diabetes: a twelve-week, randomized, placebo-controlled study. *Diabetes Obesity & Metabolism* 1999;1(3):165-72.
62. Huang D, Wei X, Li ZY, et al. Effect of rosiglitazone on serum SPARC in patients with newly diagnosed type 2 diabetes mellitus. *Modern Medicine & Health* 2016;32(9):1284-86,89.
63. Berberoglu Z, Yazici AC, Demirag NG. Effects of rosiglitazone on bone mineral density and remodelling parameters in Postmenopausal diabetic women: a 2-year follow-up study. *Clin Endocrinol (Oxf)* 2010;73(3):305-12.
64. Lu XH, Feng YX, Wu XM, Bian QX, Zhang JH. Effect of aerobic exercise on blood glucose and blood lipids in obese patients with type 2 diabetes. *China & Foreign Medical Treatment* 2009;28(6):53-54.
65. Zhu KS, Wang P. Effect of rosiglitazone on type 2 diabetes with postprandial hypoglycemia as the first symptom. *Journal of Practical Diabetology* 2009;5(1):46-47.
66. Berberoglu Z, Gursoy A, Bayraktar N, et al. Rosiglitazone decreases serum bone-specific alkaline phosphatase activity in postmenopausal diabetic women. *J Clin Endocrinol Metab* 2007;92(9):3523-30. doi: 10.1210/jc.2007-0431 [published Online First: 2007/06/28]
67. Wang J. Clinical comparison of pioglitazone hydrochloride in the treatment of patients with incipient type 2 diabetes. *China Health Vision* 2020(1):100-01.
68. Bajpeyi S, Pasarica M, Conley KE, et al. Pioglitazone-induced improvements in insulin sensitivity occur without concomitant changes in muscle mitochondrial function. *Metabolism* 2017;69:24-32.
69. Tang XQ. Observation on the effect of pioglitazone hydrochloride monotherapy in the treatment of elderly patients with incipient type 2 diabetes. *Chinese Journal of Clinical Rational Drug Use* 2017;10(35):71-72.
70. Sykes AP, Kemp GL, Dobbins R, et al. Randomized efficacy and safety trial of once-daily remogliflozin etabonate for the treatment of type 2 diabetes. *Diabetes Obes Metab* 2015;17(1):98-101.
71. Sykes AP, O'Connor-Semmes R, Dobbins R, et al. Randomized trial showing efficacy and safety of twice-daily remogliflozin etabonate for the treatment of type 2 diabetes. *Diabetes Obes Metab* 2015;17(1):94-7.
72. Chou HS, Truitt KE, Moberly JB, et al. A 26-week, placebo- and pioglitazone-controlled monotherapy study of rivoglitazone in subjects with type 2 diabetes mellitus. *Diabetes Obesity & Metabolism* 2012;14(11):1000-09.
73. Kong AP, Yamasaki A, Ozaki R, et al. A randomized-controlled trial to investigate the effects of rivoglitazone, a novel PPAR gamma agonist on glucose-lipid control in type 2 diabetes. *Diabetes Obes Metab* 2011;13(9):806-13. doi: 10.1111/j.1463-1326.2011.01411.x [published Online First: 2011/04/16]
74. Wang B. Clinical observation of pioglitazone hydrochloride on 136 patients with incipient type 2 diabetes. *China Medical Herald* 2009;6(8):58-59.
75. Gastaldelli A, Casolaro A, Pettiti M, et al. Effect of pioglitazone on the metabolic and hormonal response to a mixed meal in type II diabetes. *Clin Pharmacol Ther* 2007;81(2):205-12.
76. Goldstein BJ, Rosenstock J, Anzalone D, Tou C, Ohman KP. Effect of tesaglitazar, a dual PPAR alpha/gamma agonist, on glucose and lipid abnormalities in patients with type 2 diabetes: a 12-week dose-ranging trial. *Curr Med Res Opin* 2006;22(12):2575-90. doi: 10.1185/030079906x154169 [published Online First: 2006/12/15]
77. Hao YR, Han QW, Yuan WT, He XJ. Effects of pioglitazone on elderly patients with type 2 diabetes mellitus. *Medical Journal of Wuhan University* 2006;27(1):104-07.
78. Khan M, Murray FT, Karunaratne M, Perez A. Pioglitazone and reductions in post-challenge glucose levels in patients with type 2 diabetes. *Diabetes Obes Metab* 2006;8(1):31-8.
79. Pang WY, Liu Y, Zhao CY. Pioglitazone monotherapy for elderly patients with new-onset type 2 diabetes mellitus. *Central Plains Medical Journal* 2006;33(10):6-7.
80. Wallace TM, Levy JC, Matthews DR. An increase in insulin sensitivity and basal beta-cell fuction in diabetic subjects treated with pioglitazone in a placebo-controlled randomized study. *Diabetic Medicine* 2004;21(6):568-76.
81. Liu CM. Observation of clinical effects of pioglitazone hydrochlodde in treatment of type 2 diabetic patients. *Journal of Medical Forum* 2003;24(21):5-6.
82. Miyazaki Y, Matsuda M, DeFronzo RA. Dose-response effect of pioglitazone on insulin sensitivity and insulin secretion in type 2 diabetes. *Diabetes Care* 2002;25(3):517-23.
83. Scherbaum WA, Goke B. Metabolic efficacy and safety of once-daily pioglitazone monotherapy in patients with type 2 diabetes: a double-blind, placebo-controlled study. *Horm Metab Res* 2002;34(10):589-95.
84. Aronoff S, Rosenblatt S, Braithwaite S, et al. Pioglitazone hydrochloride monotherapy improves glycemic control in the treatment of patients with type 2 diabetes - A 6-month randomized placebo-controlled dose-response study. *Diabetes Care* 2000;23(11):1605-11.
85. Mirmiranpour H, Mousavizadeh M, Noshad S, et al. Comparative effects of pioglitazone and metformin on oxidative stress markers in newly diagnosed type 2 diabetes patients: A randomized clinical trial. *Journal of Diabetes and Its Complications* 2013;27(5):501-07.
86. Bech P, Moses R, Gomis R. The effect of prandial glucose regulation with repaglinide on treatment satisfaction, wellbeing and health status in patients with pharmacotherapy-naïve type 2 diabetes: A placebo-controlled, multicentre study. *Quality of Life Research* 2003;12(4):413-25.
87. Xu L. Efficacy of repaglinide on 31 patients with type 2 diabetes. *Journal of Aerospace Medicine* 2011;22(4):458-59.
88. Zhong XY, Lv LL, Chen ZH, Xu ZH. The effect of repaglinide on 31 patients with type 2 diabetes. *Guide of China Medicine* 2010;8(14):200-01.

89. Guo XD. Efficacy of repaglinide on 31 patients with type 2 diabetes. *Guide of China Medicine* 2009;7(15):83-84.
90. Zhao W, Zhuang LF, Peng LY, Luo JL. Observation on the efficacy of repaglinide in treatment of type 2 diabetes. *World Health Digest* 2008;5(3):71-73.
91. Gonzalez-Clemente JM. Improvement of glycaemic control by nateglinide decreases systolic blood pressure in drug-naive patients with type 2 diabetes. *European Journal of Clinical Investigation* 2008;38(3):174-79.
92. Schwarz SL, Gerich JE, Marcellari A, et al. Nateglinide, alone or in combination with metformin, is effective and well tolerated in treatment-naïve elderly patients with type 2 diabetes. *Diabetes, Obesity and Metabolism* 2008;10(8):652-60.
93. Mari A, Gastaldelli A, Foley JE, Pratley RE, Ferrannini E. β-Cell function in mild type 2 diabetic patients: Effects of 6-month glucose lowering with nateglinide. *Diabetes Care* 2005;28(5):1132-38.
94. Kato T, Inoue T, Node K. Postprandial endothelial dysfunction in subjects with new-onset type 2 diabetes: an acarbose and nateglinide comparative study. *Cardiovasc Diabetol* 2010;9:12.
95. Kirkman MS, Shankar RR, Shankar S, et al. Treating postprandial hyperglycemia does not appear to delay progression of early type 2 diabetes: the Early Diabetes Intervention Program. *Diabetes Care* 2006;29(9):2095-101.
96. Josse RG, Chiasson JL, Ryan EA, et al. Acarbose in the treatment of elderly patients with type 2 diabetes. *Diabetes Res Clin Pract* 2003;59(1):37-42.
97. Wu GT, Han YQ, Yu YC, et al. Influence of acarbose on insulin resistant of patients with type 2 diabetes mellitus. *Chinese Journal of New Drugs and Clinical Remedies* 2003;22(9):535-38.
98. Meneilly GS, Ryan EA, Radziuk J, et al. Effect of acarbose on insulin sensitivity in elderly patients with diabetes. *Diabetes Care* 2000;23(8):1162-7.
99. Scott R, Lintott CJ, Zimmet P, et al. Will acarbose improve the metabolic abnormalities of insulin-resistant type 2 diabetes mellitus? *Diabetes Res Clin Pract* 1999;43(3):179-85.
100. Chan JCN, Chan KWA, Ho LLT, et al. An asian multicenter clinical trial to assess the efficacy and tolerability of acarbose compared with placebo in type 2 diabetic patients previously treated with diet. *Diabetes Care* 1998;21(7):1058-61.
101. Fischer S, Hanefeld M, Spengler M, Boehme K, Temelkova-Kurktschiev T. European study on dose-response relationship of acarbose as a first-line drug in non-insulin-dependent diabetes mellitus: efficacy and safety of low and high doses. *Acta Diabetol* 1998;35(1):34-40.
102. Braun D, Schönherr U, Mitzkat HJ. Efficacy of acarbose monotherapy in patients with type 2 diabetes: A double-blind study conducted in general practice. *Endocrinology and Metabolism, Supplement* 1996;3(4):275-80.
103. Hou WK, Zhang BZ, Xu J, Jiang B, Shi ZC. A double-blind controlled study of glucobay in the treatment of NIDDM. *Chinese Journal of Diabetes* 1996;4(1):56-59.
104. Zheng GF, Wang JP, Zhang H, et al. The clinical investigation of the effect of acarbose on non-insulin-dependent diabetics. *Chinese Journal of Endocrinology and Metabolism* 1995;11(3):163-64,92.
105. Hotta N, Kakuta H, Sano T, et al. Long-term effect of acarbose on glycaemic control in non-insulin-dependent diabetes mellitus: a placebo-controlled double-blind study. *Diabet Med* 1993;10(2):134-8.
106. Santeusanio F, Ventura MM, Contadini S, et al. Efficacy and safety of two different dosages of acarbose in non-insulin dependent diabetic patients treated by diet alone. *Diabetes, Nutrition and Metabolism - Clinical and Experimental* 1993;6(3):147-54.
107. Hanefeld M, Fischer S, Schulze J, et al. Therapeutic potentials of acarbose as first-line drug in NIDDM insufficiently treated with diet alone. *Diabetes Care* 1991;14(8):732-7.
108. Chen DL, Han CK, Zhang HJ. Evaluation of clinical effect of acarbose in the treatment of type 2 diabetes complicated with nonalcoholic fatty liver disease. *Chinese and Foreign Medical Research* 2017;15(22):123-24.
109. Yang LH. Clinical efficacy of acarbose in the treatment of patients with type 2 diabetes combined with nonalcoholic fatty liver disease. *Guide of China Medicine* 2015;13(18):17-18.
110. Wu HX, Li JX. Efficacy of acarbose on type 2 diabetes complicated with nonalcoholic fatty liver disease. *Journal of Practical Diabetology* 2014;10(2):32-33.
111. Yang Y, Xing LF, Wang AM. Clinical study for the influence of acarbose on TNF-α and IL-6 on type 2 diabetes mellitus. *Chinese Journal of Practical Medicine* 2014;41(23):8-10.
112. Hao HR, Hu W, Yu WN. Platelet CD62P expression in early type 2 diabetes and the effect of acarbose. *Shandong Medical Journal* 2012;52(45):50-52.
113. Ye XH, Yan XD, Liang S, Lao DH. Efficacy of domestic acarbose on new-onset type 2 diabetes. *Guangxi Medical Journal* 2009;31(7):963-64.
114. Seino Y, Fujita T, Hiroi S, Hirayama M, Kaku K. Efficacy and safety of alogliptin in Japanese patients with type 2 diabetes mellitus: a randomized, double-blind, dose-ranging comparison with placebo, followed by a long-term extension study. *Current Medical Research and Opinion* 2011;27(9):1781-92.
115. Guo HG, Ling HW. Clinical effect of voglibose in the treatment of type 2 diabetes mellitus. *Acta Academiae Medicinae Xuzhou* 2001;21(4):311-13.
116. Drent ML, Tollefsen ATM, van Heusden FHJA, et al. Dose-dependent efficacy of miglitol, an α-glucosidase inhibitor, in Type 2 diabetic patients on diet alone: Results of a 24-week double-blind placebo-controlled study. *Diabetes, Nutrition and Metabolism - Clinical and Experimental* 2002;15(3):152-59.
117. Gantz I, Okamoto T, Ito Y, et al. A randomized, placebo- and sitagliptin-controlled trial of the safety and efficacy of omarigliptin, a once-weekly dipeptidyl peptidase-4 inhibitor, in Japanese patients with type 2 diabetes. *Diabetes Obes Metab* 2017;19(11):1602-09.
118. Tian M, Liang Z, Liu R, et al. Effects of sitagliptin on circulating zinc-α2-glycoprotein levels in newly diagnosed type 2 diabetes patients: A randomized trial. *Eur J Endocrinol* 2016;174(2):147-55.

119. Roden M, Merker L, Christiansen AV, et al. Safety, tolerability and effects on cardiometabolic risk factors of empagliflozin monotherapy in drug-naive patients with type 2 diabetes: a double-blind extension of a Phase III randomized controlled trial. *Cardiovascular Diabetology* 2015;14:154. doi: 10.1186/s12933-015-0314-0 [published Online First: 2015/12/25]
120. Roden M, Weng J, Eilbracht J, et al. Empagliflozin monotherapy with sitagliptin as an active comparator in patients with type 2 diabetes: a randomised, double-blind, placebo-controlled, phase 3 trial. *Lancet Diabetes Endocrinol* 2013;1(3):208-19.
121. Sun J, Li W. Effect of sitagliptin on insulin resistance in patients with type 2 diabetes. *Chinese Journal of Gerontology* 2013;33(8):1886-87.
122. Barzilai N, Guo H, Mahoney EM, et al. Efficacy and tolerability of sitagliptin monotherapy in elderly patients with type 2 diabetes: a randomized, double-blind, placebo-controlled trial. *Current Medical Research and Opinion* 2011;27(5):1049-58.
123. Iwamoto Y, Taniguchi T, Nonaka K, et al. Dose-ranging efficacy of sitagliptin, a dipeptidyl peptidase-4 inhibitor, in Japanese patients with type 2 diabetes mellitus. *Endocr J* 2010;57(5):383-94.
124. Mohan V, Yang W, Son H-Y, et al. Efficacy and safety of sitagliptin in the treatment of patients with type 2 diabetes in China, India, and Korea. *Diabetes Research and Clinical Practice* 2009;83(1):106-16.
125. Nonaka K, Kakikawa T, Sato A, et al. Efficacy and safety of sitagliptin monotherapy in Japanese patients with type 2 diabetes. *Diabetes Research and Clinical Practice* 2008;79(2):291-98.
126. Hanefeld M, Herman GA, Wu M, et al. Once-daily sitagliptin, a dipeptidyl peptidase-4 inhibitor, for the treatment of patients with type 2 diabetes. *Curr Med Res Opin* 2007;23(6):1329-39.
127. Aschner P, Kipnes MS, Lunceford JK, et al. Effect of the dipeptidyl peptidase-4 inhibitor sitagliptin as monotherapy on glycemic control in patients with type 2 diabetes. *Diabetes Care* 2006;29(12):2632-7.
128. Raz I, Hanefeld M, Xu L, et al. Efficacy and safety of the dipeptidyl peptidase-4 inhibitor sitagliptin as monotherapy in patients with type 2 diabetes mellitus. *Diabetologia* 2006;49(11):2564-71.
129. Deng X, Ma R, Zhu H, Zhu J. A randomized-controlled study of sitagliptin for treating diabetes mellitus complicated by nonalcoholic fatty liver disease. *European Journal of Gastroenterology & Hepatology* 2017;29(3):297-301. doi: 10.1097/MEG.0000000000000780 [published Online First: 2016/11/11]
130. Zhang JB, Lu YZ, Fan MS. Efficacy analysis of saxagliptin treatment of new-onset type 2 diabetes. *Guide of China Medicine* 2016;14(15):9,12.
131. Xiu SL, Wang L. Efficacy and safety of saxagliptin in newly diagnosed type 2 diabetes mellitus patients. *China Medicine* 2015;10(3):352-54.
132. Han BY, Lu LM, Zhang LP, Fan ZY. Effect of DPP-4 inhibitor saxagliptin on endothelium-dependent vasodilation in patients with type 2 diabetes. *Journal of Shanxi Medical University* 2014;45(4):291-94.
133. Prasanna Kumar KM, Jain SM, Tou C, Schutzer K-M. Saxagliptin as initial therapy in treatment-naive Indian adults with type 2 diabetes mellitus inadequately controlled with diet and exercise alone: a randomized, double-blind, placebo-controlled, phase IIIb clinical study. *International Journal of Diabetes in Developing Countries* 2014;34(4):201-09.
134. Wu YF. Evaluation of the efficacy of saxagliptin monotherapy in incipient type 2 diabetes. Zhejiang University, 2013.
135. Frederich R, McNeill R, Berglind N, Fleming D, Chen R. The efficacy and safety of the dipeptidyl peptidase-4 inhibitor saxagliptin in treatment-naïve patients with type 2 diabetes mellitus: A randomized controlled trial. *Diabetology and Metabolic Syndrome* 2012;4:36.
136. Pan CY, Yang W, Tou C, Gause-Nilsson I, Zhao J. Efficacy and safety of saxagliptin in drug-naïve Asian patients with type 2 diabetes mellitus: A randomized controlled trial. *Diabetes/Metabolism Research and Reviews* 2012;28(3):268-75.
137. Rosenstock J, Aguilar-Salinas C, Klein E, et al. Effect of saxagliptin monotherapy in treatment-naïve patients with type 2 diabetes. *Current Medical Research and Opinion* 2009;25(10):2401-11.
138. Rosenstock J, Sankoh S, List JF. Glucose-lowering activity of the dipeptidyl peptidase-4 inhibitor saxagliptin in drug-naive patients with type 2 diabetes. *Diabetes Obes Metab* 2008;10(5):376-86.
139. Mari A, Scherbaum WA, Nilsson PM, et al. Characterization of the influence of vildagliptin on model-assessed -cell function in patients with type 2 diabetes and mild hyperglycemia. *J Clin Endocrinol Metab* 2008;93(1):103-9.
140. Scherbaum WA, Schweizer A, Mari A, et al. Efficacy and tolerability of vildagliptin in drug-naïve patients with type 2 diabetes and mild hyperglycaemia. *Diabetes, Obesity and Metabolism* 2008;10(8):675-82.
141. Scherbaum WA, Schweizer A, Mari A, et al. Evidence that vildagliptin attenuates deterioration of glycaemic control during 2-year treatment of patients with type 2 diabetes and mild hyperglycaemia. *Diabetes Obes Metab* 2008;10(11):1114-24.
142. Dejager S, Razac S, Foley JE, Schweizer A. Vildagliptin in drug-naïve patients with type 2 diabetes: A 24-week, double-blind, randomized, placebo-controlled, multiple-dose study. *Hormone and Metabolic Research* 2007;39(3):218-23.
143. Pi-Sunyer FX, Schweizer A, Mills D, Dejager S. Efficacy and tolerability of vildagliptin monotherapy in drug-naive patients with type 2 diabetes. *Diabetes Research and Clinical Practice* 2007;76(1):132-38.
144. Pratley RE, Jauffret-Kamel S, Galbreath E, Holmes D. Twelve-week monotherapy with the DPP-4 inhibitor vildagliptin improves glycemic control in subjects with type 2 diabetes. *Horm Metab Res* 2006;38(6):423-8.
145. Ristic S, Byiers S, Foley J, Holmes D. Improved glycaemic control with dipeptidyl peptidase-4 inhibition in patients with type 2 diabetes: vildagliptin (LAF237) dose response. *Diabetes Obes Metab* 2005;7(6):692-8.
146. Chen J, Li YC. Efficacy of vildagliptin on newly diagnosed type 2 diabetes complicated with nonalcoholic fatty liver disease. *Journal of Clinical Internal Medicine* 2016;33(10):709-10.

147. Lin N, Liu HB. Effect of linagliptin on lipid metabolism and islet resistance in patients with type 2 diabetes. *Clinical Research* 2017;25(7):77-78,80.
148. Chen Y, Ning G, Wang C, et al. Efficacy and safety of linagliptin monotherapy in Asian patients with inadequately controlled type 2 diabetes mellitus: A multinational, 24-week, randomized, clinical trial. *Journal of Diabetes Investigation* 2015;6(6):692-98.
149. Wu W, Li Y, Chen X, et al. Effect of linagliptin on glycemic control in Chinese patients with newly-diagnosed, drug-naive type 2 diabetes mellitus: A randomized controlled trial. *Med Sci Monit* 2015;21:2678-84.
150. Wu WJ, Chen X, Shen FX, et al. Effects of linagliptin treatment on hyperglycemia and islet function in newly-diagnosed type 2 diabetic patients. *Chinese Journal of New Drugs and Clinical Remedies* 2014;33(4):263-66.
151. Barnett AH, Patel S, Harper R, et al. Linagliptin monotherapy in type 2 diabetes patients for whom metformin is inappropriate: an 18-week randomized, double-blind, placebo-controlled phase III trial with a 34-week active-controlled extension. *Diabetes Obes Metab* 2012;14(12):1145-54.
152. Kawamori R, Inagaki N, Araki E, et al. Linagliptin monotherapy provides superior glycaemic control versus placebo or voglibose with comparable safety in Japanese patients with type 2 diabetes: a randomized, placebo and active comparator-controlled, double-blind study. *Diabetes Obes Metab* 2012;14(4):348-57.
153. Del Prato S, Barnett AH, Huisman H, et al. Effect of linagliptin monotherapy on glycaemic control and markers of β-cell function in patients with inadequately controlled type 2 diabetes: A randomized controlled trial. *Diabetes, Obesity and Metabolism* 2011;13(3):258-67.
154. Pan C, Han P, Ji Q, et al. Efficacy and safety of alogliptin in patients with type 2 diabetes mellitus: A multicentre randomized double-blind placebo-controlled Phase 3 study in mainland China, Taiwan, and Hong Kong. *J Diabetes* 2017;9(4):386-95.
155. Inagaki N, Onouchi H, Maezawa H, Kuroda S, Kaku K. Once-weekly trelagliptin versus daily alogliptin in Japanese patients with type 2 diabetes: A randomised, double-blind, phase 3, non-inferiority study. *The Lancet Diabetes and Endocrinology* 2015;3(3):191-97.
156. DeFronzo RA, Fleck PR, Wilson CA, Mekki Q, Group AS. Efficacy and safety of the dipeptidyl peptidase-4 inhibitor alogliptin in patients with type 2 diabetes and inadequate glycemic control: a randomized, double-blind, placebo-controlled study. *Diabetes Care* 2008;31(12):2315-7.
157. Kutoh E, Ukai Y. Alogliptin as an initial therapy in patients with newly diagnosed, drug naïve type 2 diabetes: A randomized, control trial. *Endocrine* 2012;41(3):435-41.
158. Wang WY. The clinical effect of dapagliflozin in the treatment of newly diagnosed type 2 diabetes. *Electronic Journal of Clinical Medical Literature* 2020;7(60):159-60.
159. Liao X, Wang X, Li H, et al. Sodium-glucose cotransporter 2 (SGLT2) inhibitor increases circulating zinc-alpha2-glycoprotein levels in patients with type 2 diabetes. *Sci Rep* 2016;6:32887. doi: 10.1038/srep32887 [published Online First: 2016/09/10]
160. Ji L, Ma J, Li H, et al. Dapagliflozin as monotherapy in drug-naive Asian patients with type 2 diabetes mellitus: a randomized, blinded, prospective phase III study. *Clin Ther* 2014;36(1):84-100.e9.
161. Kaku K, Kiyosue A, Inoue S, et al. Efficacy and safety of dapagliflozin monotherapy in Japanese patients with type 2 diabetes inadequately controlled by diet and exercise. *Diabetes Obesity & Metabolism* 2014;16(11):1102-10.
162. Liu LM, Yang Y, Bao MJ, et al. Therapeutic effect of dapagliflozin on type 2 diabetes mellitus. *Journal of Chinese Practical Diagnosis and Therapy* 2014;28(9):926-27,29.
163. Kaku K, Inoue S, Matsuoka O, et al. Efficacy and safety of dapagliflozin as a monotherapy for type 2 diabetes mellitus in Japanese patients with inadequate glycaemic control: a phase II multicentre, randomized, double-blind, placebo-controlled trial. *Diabetes Obes Metab* 2013;15(5):432-40.
164. Bailey CJ, Iqbal N, T'Joen C, List JF. Dapagliflozin monotherapy in drug-naive patients with diabetes: a randomized-controlled trial of low-dose range. *Diabetes Obesity & Metabolism* 2012;14(10):951-59.
165. Ferrannini E, Ramos SJ, Salsali A, Tang W, List JF. Dapagliflozin monotherapy in type 2 diabetic patients with inadequate glycemic control by diet and exercise: A randomized, double-blind, placebo-controlled, phase 3 trial. *Diabetes Care* 2010;33(10):2217-24.
166. Kadowaki T, Haneda M, Inagaki N, et al. Empagliflozin monotherapy in Japanese patients with type 2 diabetes mellitus: a randomized, 12-week, double-blind, placebo-controlled, phase II trial. *Adv Ther* 2014;31(6):621-38.
167. Inagaki N, Kondo K, Yoshinari T, et al. Efficacy and safety of canagliflozin monotherapy in Japanese patients with type 2 diabetes inadequately controlled with diet and exercise: a 24-week, randomized, double-blind, placebo-controlled, Phase III study. *Expert Opin Pharmacother* 2014;15(11):1501-15.
168. Inagaki N, Kondo K, Yoshinari T, et al. Efficacy and safety of canagliflozin in Japanese patients with type 2 diabetes: a randomized, double-blind, placebo-controlled, 12-week study. *Diabetes Obesity & Metabolism* 2013;15(12):1136-45.
169. Stenlof K, Cefalu WT, Kim KA, et al. Efficacy and safety of canagliflozin monotherapy in subjects with type 2 diabetes mellitus inadequately controlled with diet and exercise. *Diabetes Obes Metab* 2013;15(4):372-82.
170. Hong LH. Efficacy and safety of exenatide treatment in type 2 diabetes mellitus. Zhejiang University, 2016.
171. Gastaldelli A, Brodows RG, D'Alessio D. The effect of chronic twice daily exenatide treatment on β-cell function in new onset type 2 diabetes. *Clinical Endocrinology* 2014;80(4):545-53.
172. Moretto TJ, Milton DR, Ridge TD, et al. Efficacy and tolerability of exenatide monotherapy over 24 weeks in antidiabetic drug-naive patients with type 2 diabetes: A randomized, double-blind, placebo-controlled, parallel-group study. *Clinical Therapeutics* 2008;30(8):1448-60.
173. Zhu HQ. Effect of exenatide on vascular endothelial function and cardiovascular disease risk factors in patients with type 2 diabetes. Guangxi Medical University, 2017.

174. Yamada Y, Katagiri H, Hamamoto Y, et al. Dose-response, efficacy, and safety of oral semaglutide monotherapy in Japanese patients with type 2 diabetes (PIONEER 9): a 52-week, phase 2/3a, randomised, controlled trial. *Lancet Diabetes Endocrinol* 2020;8(5):377-91.
175. Nino A, Okuda I, Wilson TH, et al. Weekly glucagon-like peptide-1 receptor agonist albiglutide as monotherapy improves glycemic parameters in Japanese patients with type 2 diabetes mellitus: A randomized, double-blind, placebo-controlled study. *Journal of Diabetes Investigation* 2018;9(3):558-66.
176. Tran S, Kramer CK, Zinman B, Choi H, Retnakaran R. Effect of chronic liraglutide therapy and its withdrawal on time to postchallenge peak glucose in type 2 diabetes. *American Journal of Physiology-Endocrinology and Metabolism* 2018;314(3):E287-E95.
177. Miyagawa J, Odawara M, Takamura T, et al. Once-weekly glucagon-like peptide-1 receptor agonist dulaglutide is non-inferior to once-daily liraglutide and superior to placebo in Japanese patients with type 2 diabetes: a 26-week randomized phase III study. *Diabetes Obes Metab* 2015;17(10):974-83.
178. Tan XR, Liang ZR, Li SB, et al. Effects of GLP-1 receptor agonist on Zinc-α2-glycoprotein and its relationship with insulin resistance in patients with newly diagnosed type 2 diabetes. *Journal of Chongqing Medical University* 2015;40(11):1395-400.
179. Retnakaran R, Kramer CK, Choi H, Swaminathan B, Zinman B. Liraglutide and the preservation of pancreatic beta-cell function in early type 2 diabetes: The LIBRA Trial. *Diabetes Care* 2014;37(12):3270-78.
180. Seino Y, Rasmussen MF, Zdravkovic M, Kaku K. Dose-dependent improvement in glycemia with once-daily liraglutide without hypoglycemia or weight gain: A double-blind, randomized, controlled trial in Japanese patients with type 2 diabetes. *Diabetes Res Clin Pract* 2008;81(2):161-8.
181. Vilsboll T, Brock B, Perrild H, et al. Liraglutide, a once-daily human GLP-1 analogue, improves pancreatic B-cell function and arginine-stimulated insulin secretion during hyperglycaemia in patients with Type 2 diabetes mellitus. *Diabetic Medicine* 2008;25(2):152-56.
182. Fan P. Efficacy of long-acting GLP-1 analogue combined with low-carbohydrate diet in the treatment of obese type 2 diabetes patients. *Journal of Practical Diabetology* 2020;16(6):43-44.
183. Li QY, Liu X, Li HX, et al. Clinical effect of liraglutide on type 2 diabetes mellitus complicated with polycystic ovary syndrome. *China Medicine* 2018;13(4):549-52.
184. Liu MR. The clinical efficacy and safety observation on a low carbohydrate diet combined with liraglutide in obese patients with diabetes. University of South China, 2017.
185. Fonseca VA, Alvarado-Ruiz R, Raccah D, et al. Efficacy and safety of the once-daily GLP-1 receptor agonist lixisenatide in monotherapy: a randomized, double-blind, placebo-controlled trial in patients with type 2 diabetes (GetGoal-Mono). *Diabetes Care* 2012;35(6):1225-31.
